# Supplementary material for: Discovery of a CNS active GSK3 degrader using orthogonally reactive linker screening
Source: Nat Commun. 2025 Oct 6;16:8857. doi: 10.1038/s41467-025-63928-8 (PMC12501029; doi:10.1038/s41467-025-63928-8)
Supplement: Supplementary file 1 — Supplementary Information [file 41467_2025_63928_MOESM1_ESM.pdf]

Supporting Information

Discovery of a CNS active GSK3 degrader using orthogonally reactive linker screening

Andreas Holmqvist [1$], Nur Mehpare Kocaturk[1$], Christina Duncan[2], Jennifer Riley[2], Steven Baginski[2], Graham Marsh[3], Joel Cresser-Brown[3], Hannah Maple[3], Kristiina Juvonen[1], Gajanan Sathe[1], Nicola Morrice[4], Calum Sutherland[5], Kevin D. Read[2] and William Farnaby[1*]

1. Centre for Targeted Protein Degradation, School of Life Sciences, University of Dundee, Dundee, DD1 5JJ, U.K.
2. Wellcome Centre for Anti-Infective Research, Drug Discovery Unit, Division of Biological Chemistry and Drug Discovery, School of Life Sciences, University of Dundee, DD1 5EH, U.K.
3. Bio-Techne (Tocris), The Watkins Building, Atlantic Road, Avonmouth, Bristol BS11 9QD, U.K.
4. Division of Neuroscience, School of Medicine, IMS/ WTB Complex, University of Dundee, Dundee, DD1 5EH, UK
5. School of Medicine, University of Dundee, Ninewells Hospital & Medical School DD1 9SY, U.K.

[$]These authors contributed equally
[*]Corresponding author – for enquiries please contact w.farnaby@dundee.ac.uk

Table Of Contents

ABBREVIATIONS 3

SUPPLEMENTARY TABLES 3

SUPPLEMENTARY TABLE 1. IN VITRO ADME DATA. 3
SUPPLEMENTARY TABLE 2. IN VIVO PHARMACOKINETIC PARAMETERS FOR COMPOUNDS 22, 25 AND 26 3
SUPPLEMENTARY TABLE 3. IN VIVO PHARMACOKINETIC PARAMETERS FOR COMPOUND 24 IN FEMALE BALB/C MICE AT DOSES AND ROUTES AS INDICATED. 4
SUPPLEMENTARY TABLE 4. BRAIN AND BLOOD LEVELS FOR COMPOUND 24 5
SUPPLEMENTARY TABLE 6. HIGH THROUGHPUT ASSAY SCREENING SUMMARY. 5

SUPPLEMENTARY FIGURES 7

FIGURE S1. IMPACT OF LINKER CONJUGATION CHEMISTRY ON PHYSICOCHEMICAL PROPERTIES, REACTION OPTIMIZATION AND STRUCTURAL INSIGHTS 7
FIGURE S2. CHARACTERISATION OF HIBIT KNOCK-IN HEK293 CELLS AND FURTHER PROFILING OF SELECTED AND RESYNTHESISED HIT COMPOUNDS. 8
FIGURE S3. CELL VIABILITY TESTS 9
FIGURE S4. CELLULAR PROFILING AND MECHANISM OF ACTION STUDIES 9
FIGURE S5. FURTHER PROFILING OF GSK3 PROTACS IN CELLS AND IN VIVO 11
FIGURE S6. UNCROPPED WESTERN BLOTS AND SDS-PAGE IMAGES OF ALL BIOLOGICAL REPLICATES FOR FIGURE S2D. 12
FIGURE S7. UNCROPPED WESTERN BLOTS AND SDS-PAGE IMAGES OF ALL BIOLOGICAL REPLICATES FOR FIGURE S2E. 13
FIGURE S8. UNCROPPED WESTERN BLOTS AND SDS-PAGE IMAGES OF ALL BIOLOGICAL REPLICATES FOR FIGURE S2F. 14
FIGURE S9. UNCROPPED WESTERN BLOTS AND SDS-PAGE IMAGES OF ALL BIOLOGICAL REPLICATES FOR FIGURE S4A. 16
FIGURE S10. UNCROPPED WESTERN BLOTS AND SDS-PAGE IMAGES OF ALL BIOLOGICAL REPLICATES FOR FIGURE S4F. 17

FIGURE S11. UNCROPPED WESTERN BLOTS AND SDS-PAGE IMAGES OF ALL BIOLOGICAL REPLICATES FOR FIGURE S4I. 18
FIGURE S12. UNCROPPED WESTERN BLOTS AND SDS-PAGE IMAGES OF FIGURE S5C........ 18

SUPPLEMENTARY METHODS 19

EXPERIMENTAL DETAILS OF LINKER RELATED BUILDING BLOCKS 19
EXPERIMENTAL DETAILS OF E3 LIGASE LIGAND RELATED BUILDING BLOCKS 28
SYNTHESIS OF 1-[3-(BROMOMETHYL)PHENYL]HEXAHYDROPYRIMIDINE-2,4-DIONE (13) 28
(2S,4R)-1-((S)-2-(2-BROMOACETAMIDO)-3,3-DIMETHYLBUTANOYL)-4-HYDROXY-N-(4-(4-METHYLTHIAZOL-5-YL)BENZYL)PYRROLIDINE-2-CARBOXAMIDE (14) 29
SYNTHESIS OF 4-(BROMOMETHYL)-N-(2,6-DIOXOPIPERIDIN-3-YL)-2-FLUOROBENZAMIDE (15) .. 30
SYNTHESIS OF 1-(6-(BROMOMETHYL)-1-METHYL-1H-INDAZOL-3-YL)DIHYDROPYRIMIDINE-2,4(1H,3H)-DIONE (16) 31
SYNTHESIS OF 5-(BROMOMETHYL)-*N*-(2,6-DIOXOPIPERIDIN-3-YL)PICOLINAMIDE (17) 33
SYNTHESIS OF 1-(4-(BROMOMETHYL)PHENYL)DIHYDROPYRIMIDINE-2,4(1H,3H)-DIONE (18) 34
EXPERIMENTAL DETAILS OF GSK3 LIGAND RELATED BUILDING BLOCKS 36
N-(3-AZIDOPROPYL)-5-(3-CHLORO-4-METHOXYPHENYL)OXAZOLE-4-CARBOXAMIDE (19) 36
SYNTHESIS OF 2-(4-AZIDOPHENYL)-N-(4-MORPHOLINOPYRIDIN-3-YL)IMIDAZO[1,2-B]PYRIDAZINE-8-CARBOXAMIDE (20) 36
EXPERIMENTAL DETAILS OF NEGATIVE CONTROL RELATED BUILDING BLOCKS 39
SYNTHESIS OF 1-(6-(BROMOMETHYL)-1-METHYL-1H-INDAZOL-3-YL)-3-METHYLDIHYDROPYRIMIDINE-2,4(1H,3H)-DIONE (57) 39
GENERAL PROTOCOL FOR PLATE-BASED LIBRARY SYNTHESIS 41
EXPERIMENTAL DETAILS OF SELECTED HITS 41

NMR SPECTRA 49

Abbreviations

STAB – Sodium triacetoxyborohydride

DMSO - DimethylsuLfoxide

DCM - Dichloromethane

HATU - Hexafluorophosphate Azabenzotriazole Tetramethyl Uronium

DMF - Dimethylformamide

TFA – 2,2,2-Trifluoroacetic acid

THF – Tetrahydrofuran

TBAF - Tetra-n-butylammonium fluoride

T3P - Propanephosphonic acid anhydride

THTPA - tris-hydroxypropyltriazolylmethylamine

SCX – Strong Cation Exchange

Supplementary Tables

Supplementary Table 1. In vitro ADME data.

Compound	*Scaled Microsomal Stability (ml/min/g liver)			RealSol (μM)	Plasma Protein Binding (Fu)			Brain Tissue Binding (Fu)
	MuClint	RatClint	HuClint		Mu	Rat	Hu	
**21**	15	2.6	27	3.3	-	-	-	-
**22**	<0.53	0.71	3.6	<1	-	-	-	0.0029
**24**	2.4	0.51	0.88	2.7	0.0052	0.00064	0.0072	0.0012
**25**	17	2.4	6.4	<1	-	-	-	0.0076
**26**	4.0	1.3	1.6	2.3	0.021	0.010	0.050	0.012

*Scaled Clint values based on scaling factors of 48 (mouse), 46 (rat) and 40 (human) mg microsomal protein per g of liver.

Supplementary Table 2. In vivo pharmacokinetic parameters for compounds **22**, **25** and **26** in the female Balb/c mouse following a single intravenous bolus dose as a cassette at 0.5 mg/kg. $AUC_{0\text{-}Inf}$ not reported, as extrapolation >20%. Data represent means of independent measurements from 3 animals.

**Compound**	**$AUC_{0\text{-}last}$ (min*ng/mL)**	**Cl_obs (mL/min/kg)**	**Vss_obs (L/kg)**	**Half_Life (h)**
**22**	26616	12	2.8	2.8
**25**	18499	15	1.1	1.0
**26**	35395	8	1.9	2.7

Supplementary Table 3. In vivo pharmacokinetic parameters for compound **24** in female Balb/c mice at doses and routes as indicated. $AUC_{0\text{-}Inf}$ not reported, as extrapolation >20%. Data represent means of independent measurements from 3 animals.

**Administration Route and dose**	**AUC_{last}**	**Cl_obs**	**Vss_obs**	**Half_Life**	**Cmax**	**Tmax**	**F**
	**(min*ng/mL)**	**(mL/min/kg)**	**(L/kg)**	**(h)**	**(ng/mL)**	**(min)**	**(%)**
IV, 0.37 mg/kg	34310	10	2.0	2.3	-	-	-
Oral, 3 mg/kg	4930	-	-	-	19	120	1.6

Supplementary Table 4. Brain and blood levels for compound **24** following intravenous bolus administrations at doses and timepoints indicated to female Balb/c mouse. Data represent means of independent measurements from 3 animals.

Dose (mg/kg, i.v.)	Time (hours)	Mean Brain Concentration (nM)	Mean Blood Concentration (nM)	Brain:Blood Ratio
0.37	2	16	88	0.18
5	4	68	696	0.10

Supplementary Table 6. High Throughput assay screening summary.

Category	Parameter	Description
Assay	Type of assay: Cell-based	
	Target: GSK3β-HiBiT HEK293	https://www.uniprot.org/uniprotkb/P49841/entry
	Primary measurement: Luminescence	Lytic Detection System sensitively quantifies HiBiT-tagged proteins in cell lysates where loss of signal indicates reduction (degradation) of protein levels.
	Key reagents: HiBiT lytic assay buffer (Promega)<br>Assay protocol: https://www.promega.co.uk/resources/protocols/technical-manuals/500/nano-glo-hibit-lytic-detection-system-protocol/	
Library	Library size: 147 x bifunctional molecules targeting GSK3	crude library compounds generated from plate-based chemistry described in supplementary information
	Library composition:	
	2 x GSK3 binders<br>12 x Orthogonally reactive linkers<br>6 x E3-binders<br>1 x Benzyl bromide	Benzyl bromide analogue acts as a negative control
	Source: Generated from plate-based chemistry described in supplementary methods	
Screen	Format: 384-well plate, all wells used	
	Concentration(s) tested: assumed 10 mM of each compound in DMSO stock from plates, serially diluted into assay plate (tested at concentrations are 30μM, 10μM, 3μM, 1μM, 0.3μM, 0.1μM, 0.03μM and 0.01μM)	generated from plate-based chemistry described in supplementary information
	Plate control: 10 mM of PT-65 (Tocris) in DMSO (Sigma), serially diluted as test compounds.	positive control
	Reagent/Compound dispensing system(s)	
	MultidropTM Combi reagent dispenser	Cells are seeded on compounds and HiBiT lytic regent dispensed on into the assay plates
	Echo 500 liquid handler	Serial dilutions of compounds into assay plates
	Bravo	Transferring compounds into echo source plates
	PheraStar plate reader (BMG)	Luminescence signal measured
	Assay validation/QC:	GSK3β degradation positive control
	Correction factors:	DMSO, PT-65
	Normalization:	DMSO as background
Post-HTS analysis	Hit criteria: DC_{50} > 1 uM	Anything above that limit considered as not active
	Hit rate: 47 compounds / 147 with >50% D_{max} and 29 compounds / 147 with <100nM DC_{50}	

Additional assay(s):

<u>Confirmation of hit purity, structure and potency of compounds:</u> Compounds are repurified, analyzed then re-tested in HiBiT lytic degradation assays.

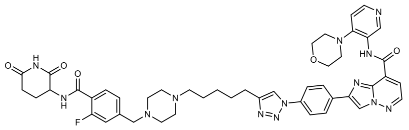

C7 from plate 2 re-synthesized, purified and analyzed by LCMS, HRMS and NMR.

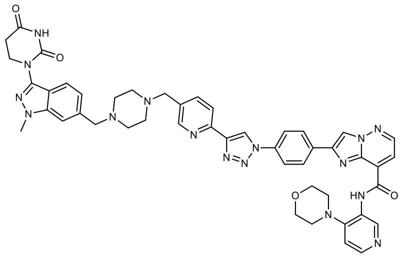

D2 from plate 2 re-synthesized, purified and analyzed by LCMS, HRMS and NMR.

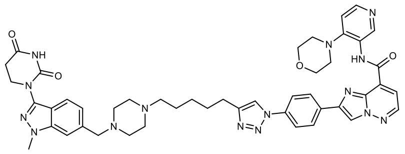

D7 from plate 2 re-synthesized, purified and analyzed by LCMS, HRMS and NMR.

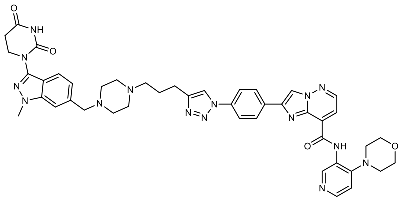

D12 from plate 2 re-synthesized, purified and analyzed by LCMS, HRMS and NMR.

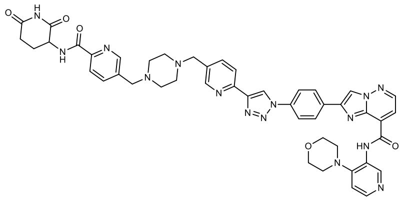

F2 from plate 2 re-synthesized, purified and analyzed by LCMS, HRMS and NMR.

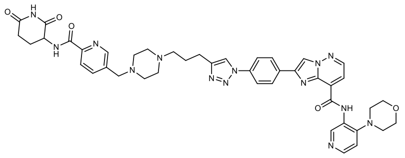

F12 from plate 2 re-synthesized, purified and analyzed by LCMS, HRMS and NMR.

Western blotting: Quantitation of degradation of wild-type endogenous GSK3α and β paralogs for hit validation in HEK293, HAP1, HAP1 CRBN KO, MEF and differentiated SH-SY5Y cells.

Mode of action study: Competition assays using 1 µM MG132 (Merck, 474790-5mg), 1 µM MLN4924 (Merck, 5054770001) or 1 µM GSK3 inhibitor (compound 29) to validate dependency on neddylation, proteasome activity and GSK3 engagement respectively.

Supplementary Figures

Figure S1. Impact of linker conjugation chemistry on physicochemical properties, reaction optimization and structural insights**.** (**A**) Analysis of various linker conjugation chemistries and their effects on cLogP and hydrogen bond donors (HBD). (**B**) Screening of S_N2 reaction conditions (**C**). X-ray co-crystal structure of PF-367 (green sticks) bound to the kinase domain of GSK3β (PDB ID: 5K5N), highlighting residue Arg141 and its measured distance to the triazole group of PF-367. (**D**) X-ray co-crystal structure of CMP-47 (purple sticks) bound to the kinase domain of GSK3β (PDB ID: 8DJC), highlighting residue Arg141 and its measured distance to the triazole attachment point. (**E**) 2D Chemical structure of PF-367. (**F**) 2D Chemical structure of CMP-47.

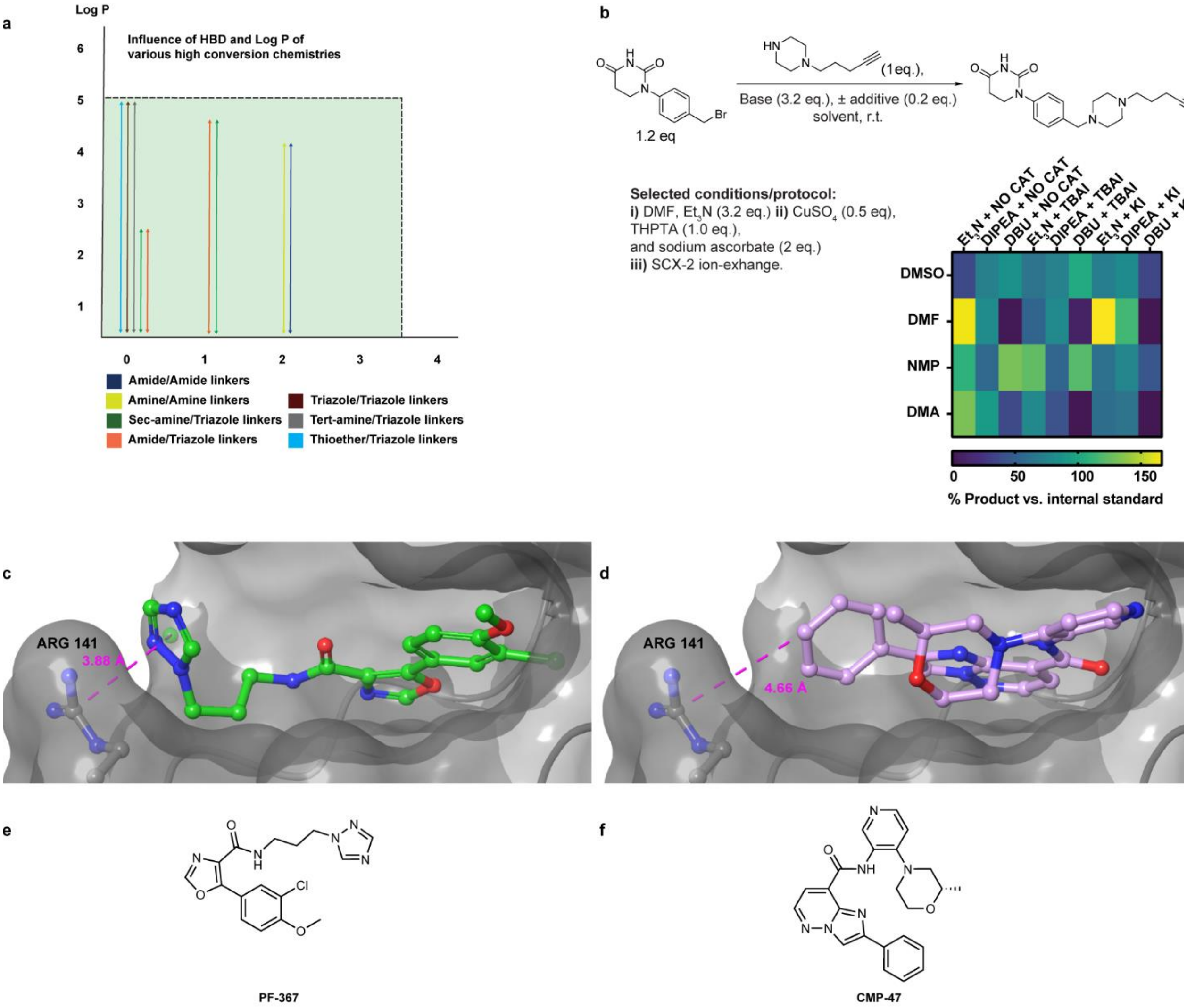

Figure S2. Characterisation of HiBiT Knock-in HEK293 cells and further profiling of selected and resynthesised hit compounds. (**A-C**) Sequencing of the GSK3β-HiBiT KI HEK293 clone utilised for high-throughput screening. (**D**) Immunoblotting-based HiBiT expression detection and half-life determination of GSK3β-HiBiT vs WT up to 48 hours (representative of n=3 biological replicates). (**E**) Immunoblotting-based half-life determination of GSK3α (WT) up to 48 hours (representative of n=2 biological replicates). (**F**) Immunoblotting based degradation profile of selected compounds (representative of n= 3 biological replicates, HEK293, 10 nM or 100 nM, 24 hours) (**G**) Immunoblots shown in **F** were quantified, normalised to relevant internal controls and plotted for changes in GSK3β and GSK3α levels for compounds **21** (orange), **24** (green), **25** (teal), **26** (blue), **22** (pink) and **23** (purple) with shading intensity corresponding to concentration (lightest = 10 nM, darkest = 100 nM) (n=3 biological replicates, mean and ±SD). (**H-K**) Kinetic live cell degradation of GSK3β with compounds **21** (orange), **22** (pink), **23** (purple) and **25** (blue)**,** monitored in LgBit overexpressing GSK3β-HiBiT KI HEK293 cells where shading intensity reflects compound concentration, with darkest colour indicating highest tested concentration (3 µM) and lightest colour indicating lowest tested concentration (10 nM) (n=3 biological repeats, and data representatives of 1 biological repeat per compound). (**L**) Kinetic degradation rate assessment of compounds **21** (orange), **22** (pink), **24** (green) and **25** (blue) in LgBit overexpressing GSK3β-HiBiT cells for selected compounds. Plot shows calculated degradation rates for tested concentrations using Michaelis-Menten analysis (GraphPad), n=3 biological replicates, error bars represent mean and ±SD.

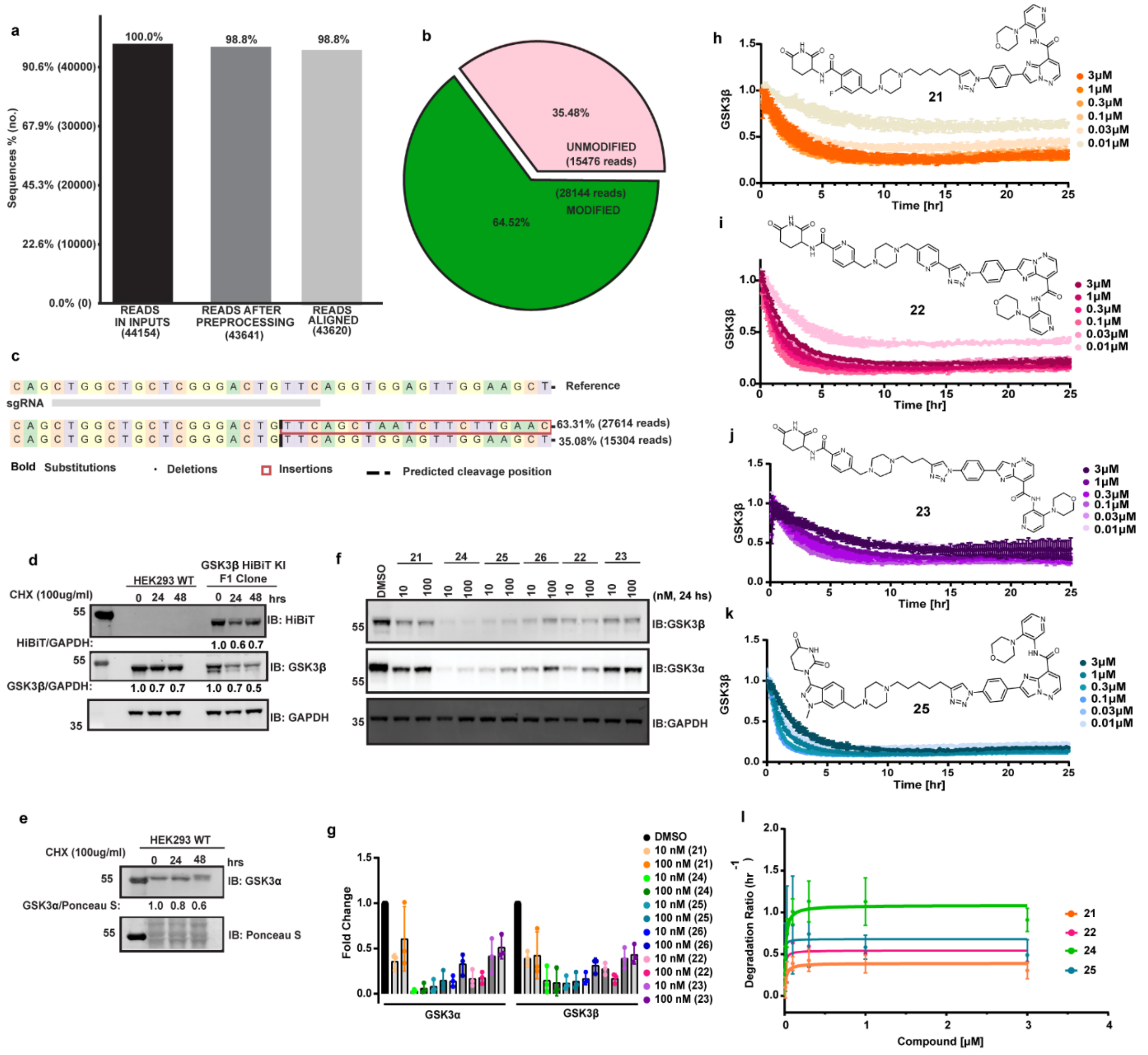

Figure S3. Cell viability tests following compound 24 (Green) mediated GSK3 degradation and negative control 27 (Grey). (**A-C**) Luminescence based quantification of GSK3β degradation following treatment of GSK3β-HiBiT HEK293 cells at doses and timepoints indicated. (n>3 biological replicates, individual dots represent each technical measurements in all biological repeats and error bars represent mean and ±SD, *p* values are calculated as p<0.0001 as indicated, two-tailed unpaired *t*-test (Graphpad)). (**D-F**) Fluorescence based quantification of cell viability, multiplexed with luminescent read-out of HiBiT signal/GSK3β degradation in the same conditions as in panels A-C**.** Live cells retain active protease capable of cleaving substrate and inducing fluorescence normalised to DMSO conditions (n>3 biological replicates, individual dots represent each technical measurements in all biological repeats with mean and ±SD indicated, *p* values are calculated as non-significant (ns) as indicated, two-tailed unpaired *t*-test (GraphPad)).

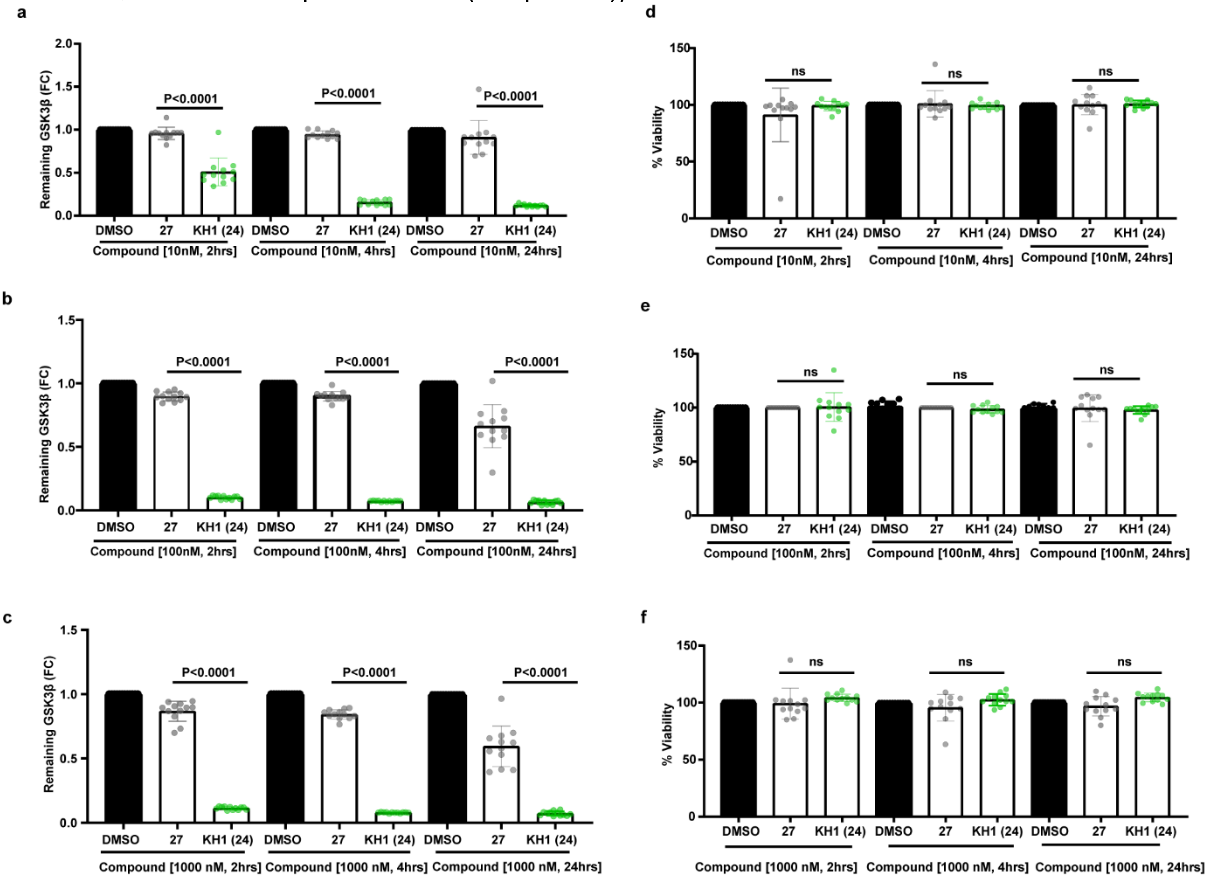

Figure S4. Cellular profiling and mechanism of action studies for compounds **24**, **25** and **26**. (**A**) Immunoblotting of endogenous GSK3α and GSK3β in HAP1 cells following treatment with **24**, **25** and **26** for 4 hours with indicated concentrations (representative of n=4 biological replicates). The samples derive from the same experiment, gels and blots were processed in parallel. (**B**) and (**C**) Relative quantification of GSK3α and GSK3β levels normalised to GAPDH in HAP1 cells following treatment with **24** (green), **25** (teal) and **26** (blue) with shading intensity corresponding to concentration (lightest = 10 nM, darkest = 100 nM) (n=4 biological replicates,

with error bars representing mean, ±SD). (**D**) and (**E**) Chemical structures of negative control compounds **27** and **28**, featuring methylated dihydrouracil rings which abrogate CRBN engagement. (**F**) Assessment of CRBN dependency on GSK3α and GSK3β degradation in either WT or CRBN KO HAP1 cells following treatment with compounds **24** or **26** for 2 hours (Representative blot of n=3 biological replicates). The samples derive from the same experiment, gels and blots were processed in parallel. (**G**) and (**H**) Quantification of relative GSK3α and GSK3β levels normalised to GAPDH in either WT or CRBN KO HAP1 cells following treatment with compounds **24** (green) or **26** (blue) from panel **G** compared to corresponding negative control **27** (light grey) and **28** (dark grey) (n=3 biological replicates, error bars represent mean, ±SD). (**I**) Immunoblotting of endogenous total GSK3 in differentiated SH-SY5Y cells after 24 hours treatment with compounds **24** and **27** with indicated concentrations (Representative blot of n=3 biological replicates, error bars represent mean, ±SD). (**J**) Proteome wide compound selectivity of compound KH2 (**26**) (blue) vs **28** (grey) normalised to DMSO, following 10 nM treatment for 2 hours in HEK293 cells (n=3 biological replicates, error bars represent mean, ±SD). (**K**) Proteome wide compound selectivity for compound **24** (green) vs **27** (grey) normalised to DMSO, following 10 nM treatment for 4 hours in HEK293 cells (n=3 biological replicates, error bars represent mean, ±SD). N.B. GSK3α and GSK3β points overlap. (**L**) Phosphoproteomic analysis of compound **24** (purple) and **27** (grey) normalised to DMSO following 10 nM treatment for 4 hours (n=3 biological replicates). Two-tailed t-test performed with 250 randomisations to assess the statistical significance between groups using Perseus software.

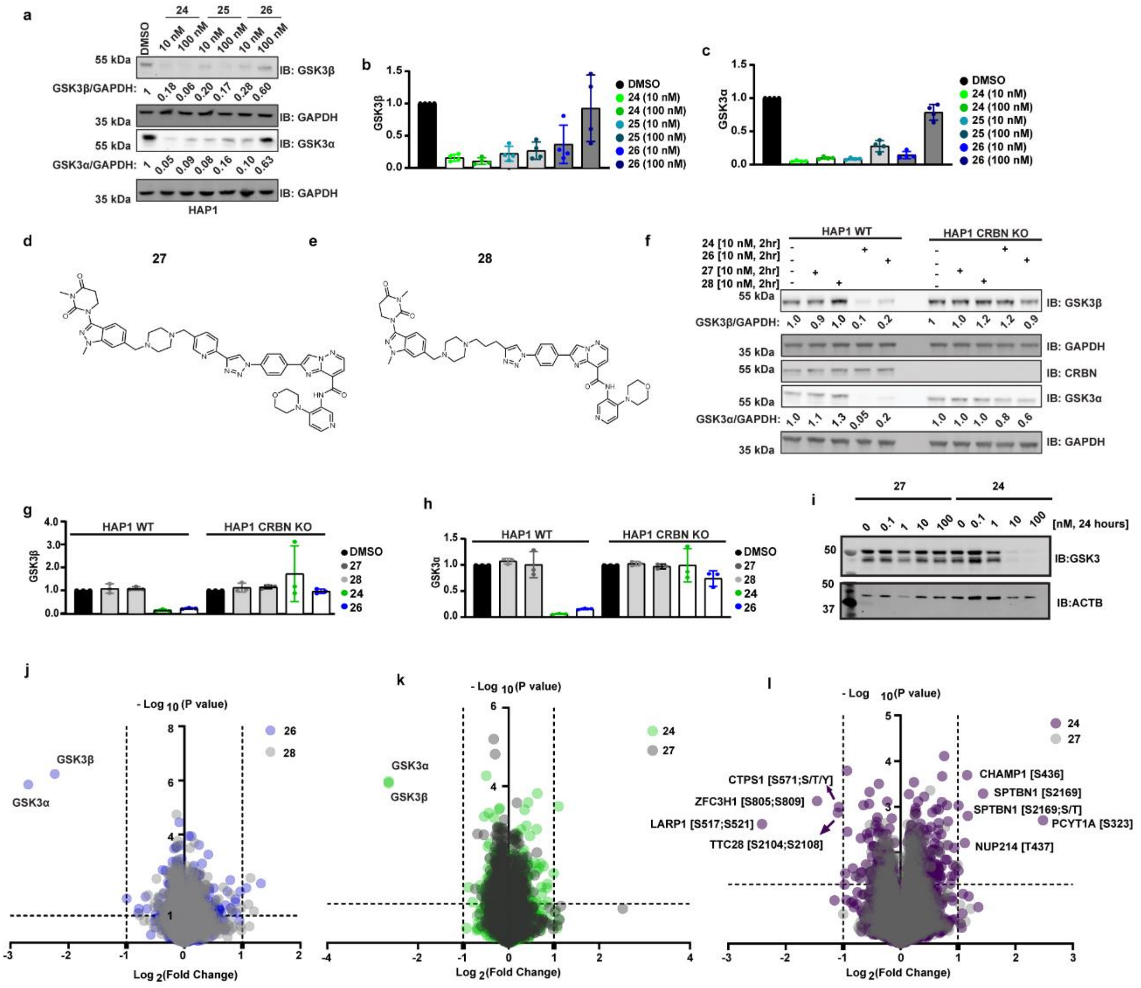

Figure S5. Further profiling of GSK3 PROTACs in cells and in vivo**.** (**A**) Pharmacokinetic profile of compounds **22** (pink), **25** (teal) and KH2 (**26**) (blue) in mouse following single cassette dosing of 0.5 mg/kg i.v. Data represent means of independent measurements from 3 animals showing whole blood concentration-time profile. (**B**) Serum shift assay. Degradation of GSK3β-HiBiT by KH2 (**26**) in GSK3β-HiBiT KI HEK293 cells in the presence of 10% fetal bovine serum (FBS) (blue) or mouse serum (MS) (black) (24 hours, n=3 biological replicates, error bars represent mean, ± SD). (**C**) Assessment of GSK3β downstream substrates in liver and brain following single dose of KH1 (**24**) 5 mg/kg in mice after 4 hours, data represent samples from 3 animals. The samples derive from the same experiment, gels and blots were processed in parallel. (**D**) Quantification of relative β-catenin and phospho-CRMP2 (Thr509-514) levels in brain and liver following single dose of KH1 (**24**) (green dots) compared to control (CNT) (black dots) from panel C (Data represent means of independent measurements of 3 animals ± SD, two-tailed unpaired *t*-test).

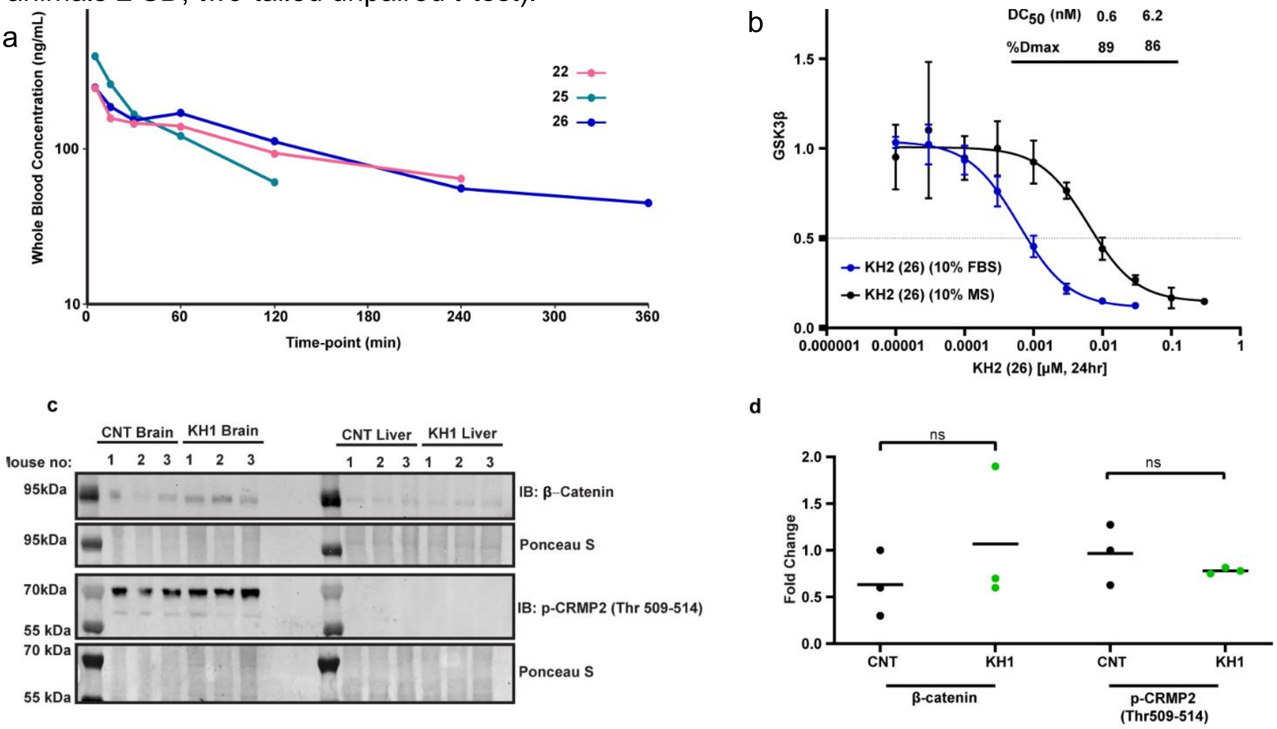

Figure S6. Uncropped Western blots and SDS-PAGE images of all biological replicates for Figure S2d. Boxes indicate the regions shown in the figure. Same samples for the replicates run through parallel gels.

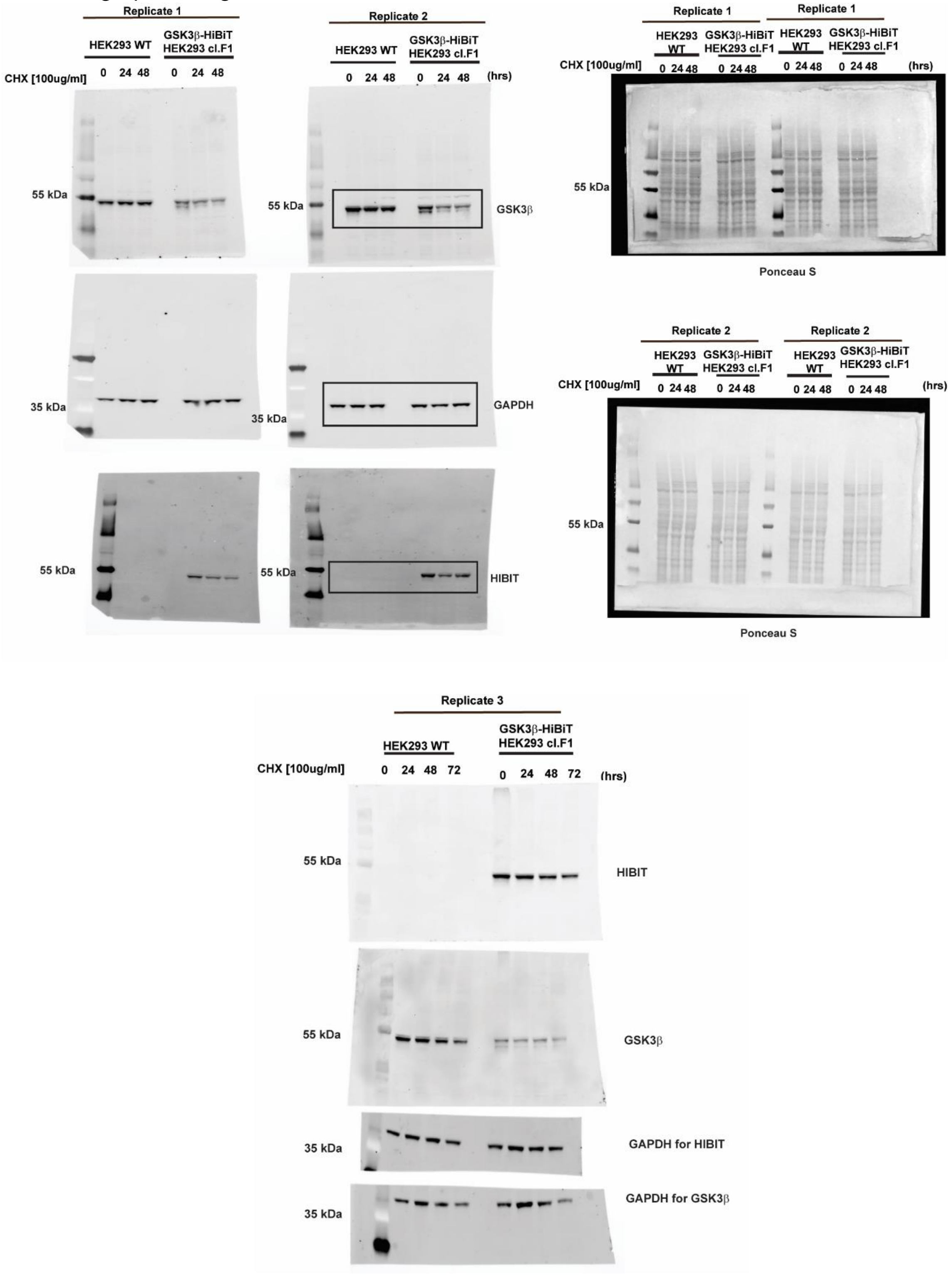

Figure S7. Uncropped Western blots and SDS-PAGE images of all biological replicates for Figure S2e. Boxes indicate the regions shown in the figure. Same samples for the replicates run through parallel gels.

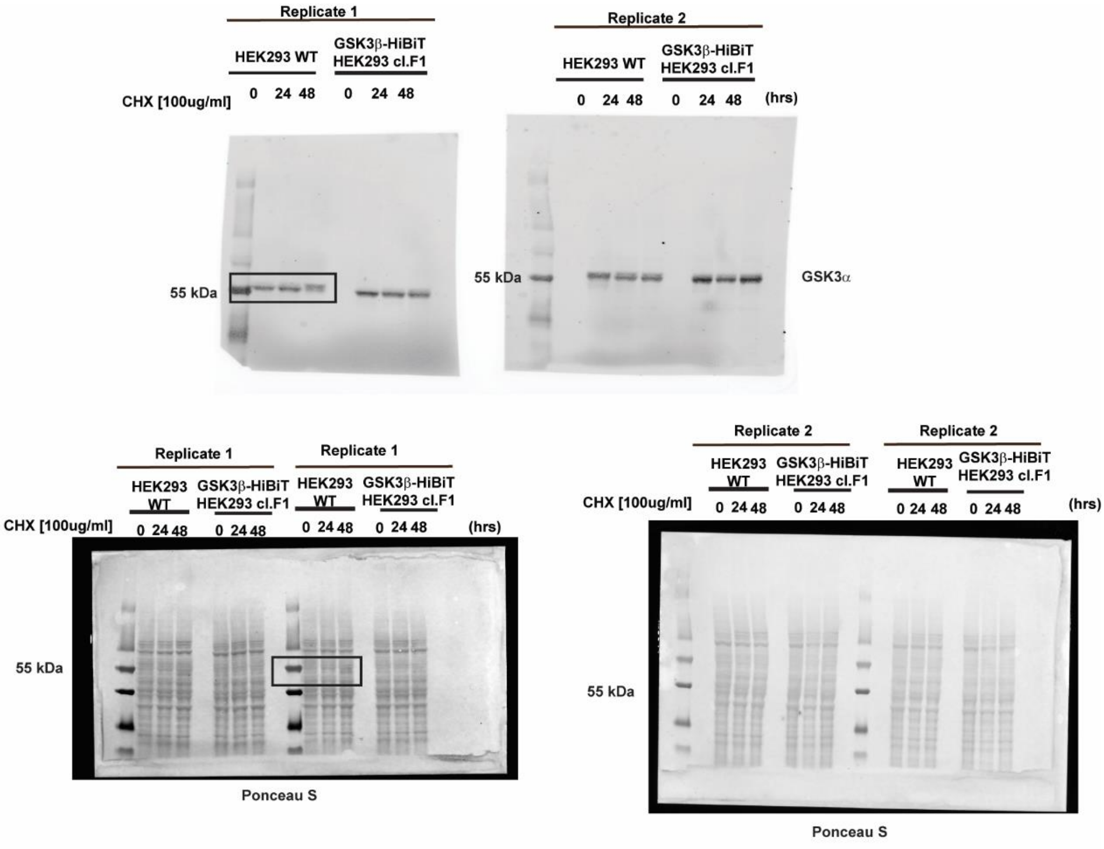

Figure S8. Uncropped Western blots and SDS-PAGE images of all biological replicates for Figure S2f. Boxes indicate the regions shown in the figure. Same samples for the replicate run through parallel gels.

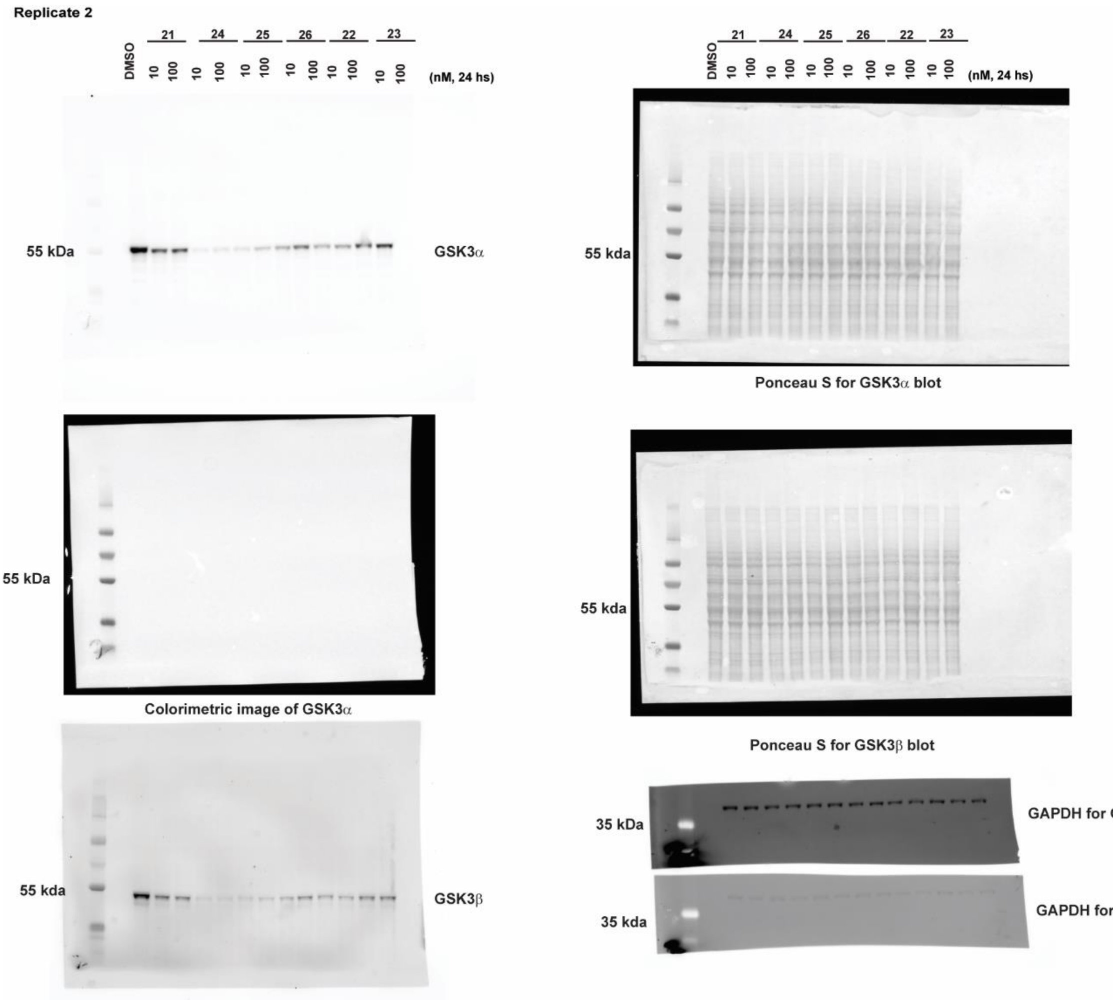

Replicate 1

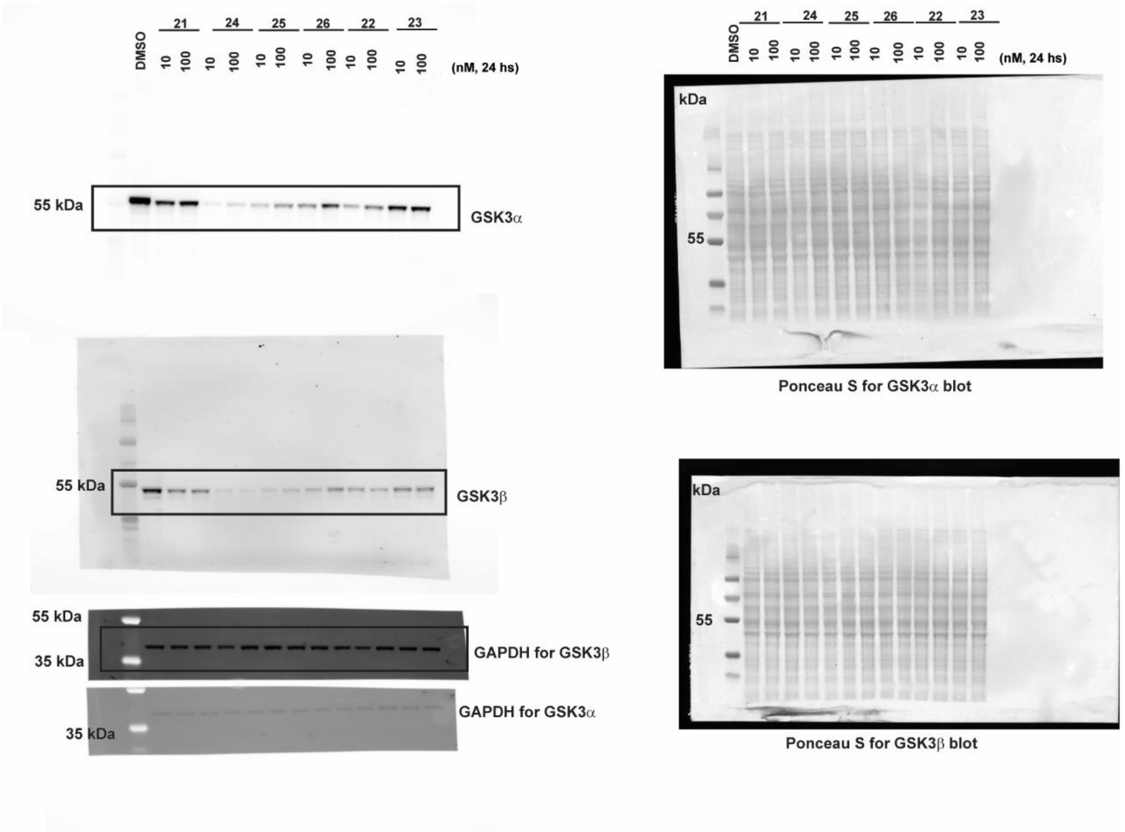

Replicate 3

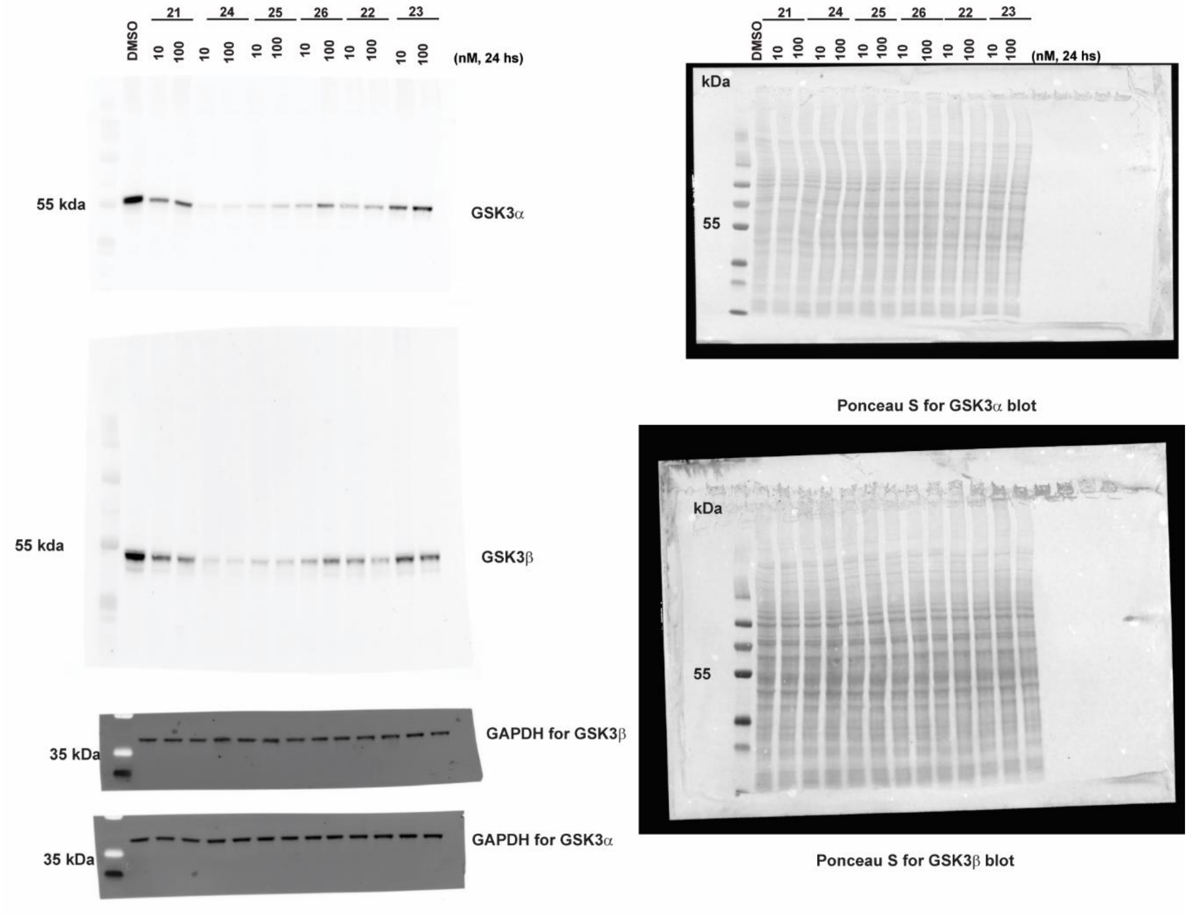

Figure S9. Uncropped Western blots and SDS-PAGE images of all biological replicates for Figure S4a. Boxes indicate the regions shown in the figure. Same samples for the replicate run through parallel gels.

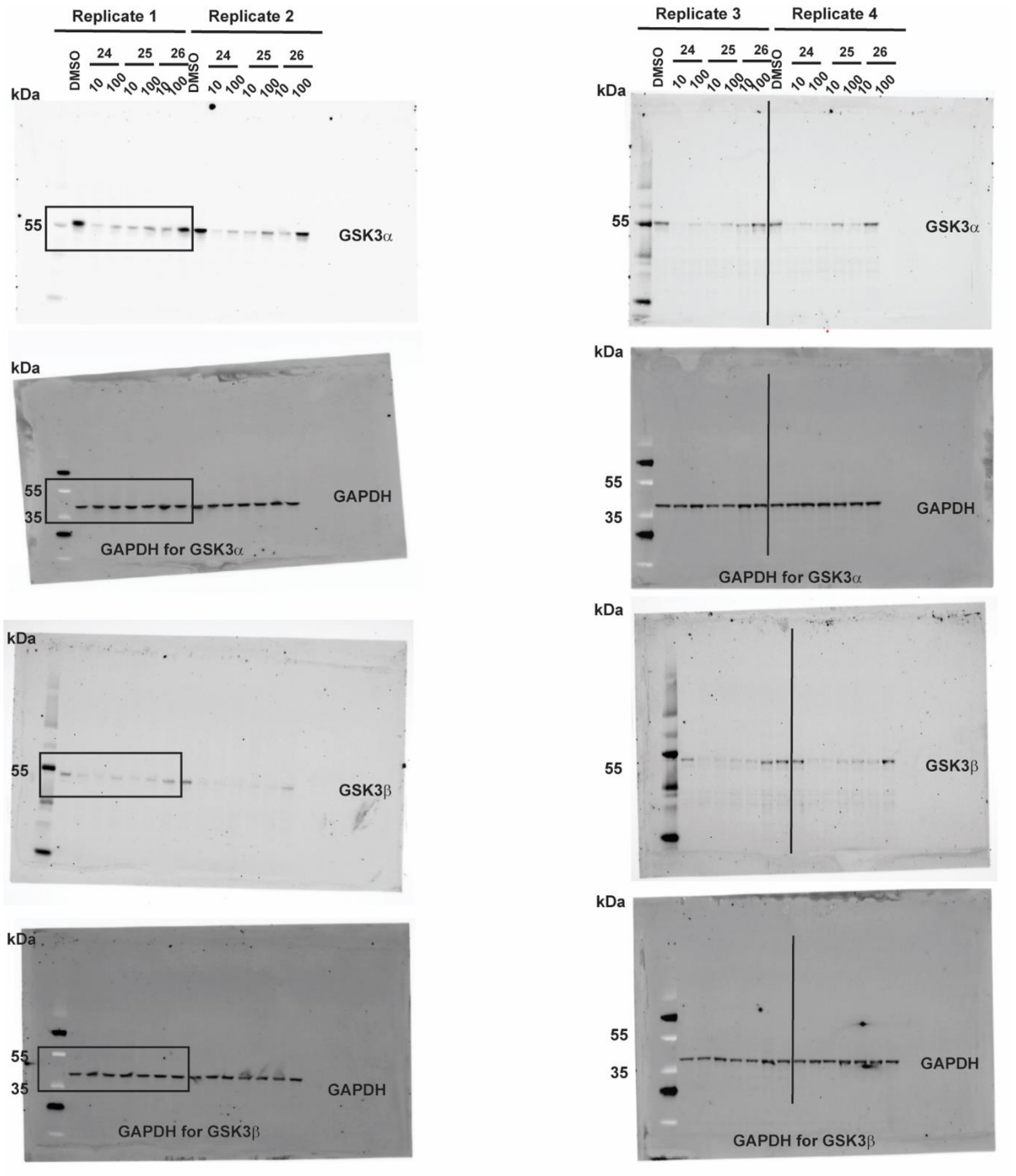

Figure S10. Uncropped Western blots and SDS-PAGE images of all biological replicates for Figure S4f. Boxes indicate the regions shown in the figure. Same samples for the replicate run through parallel gels.

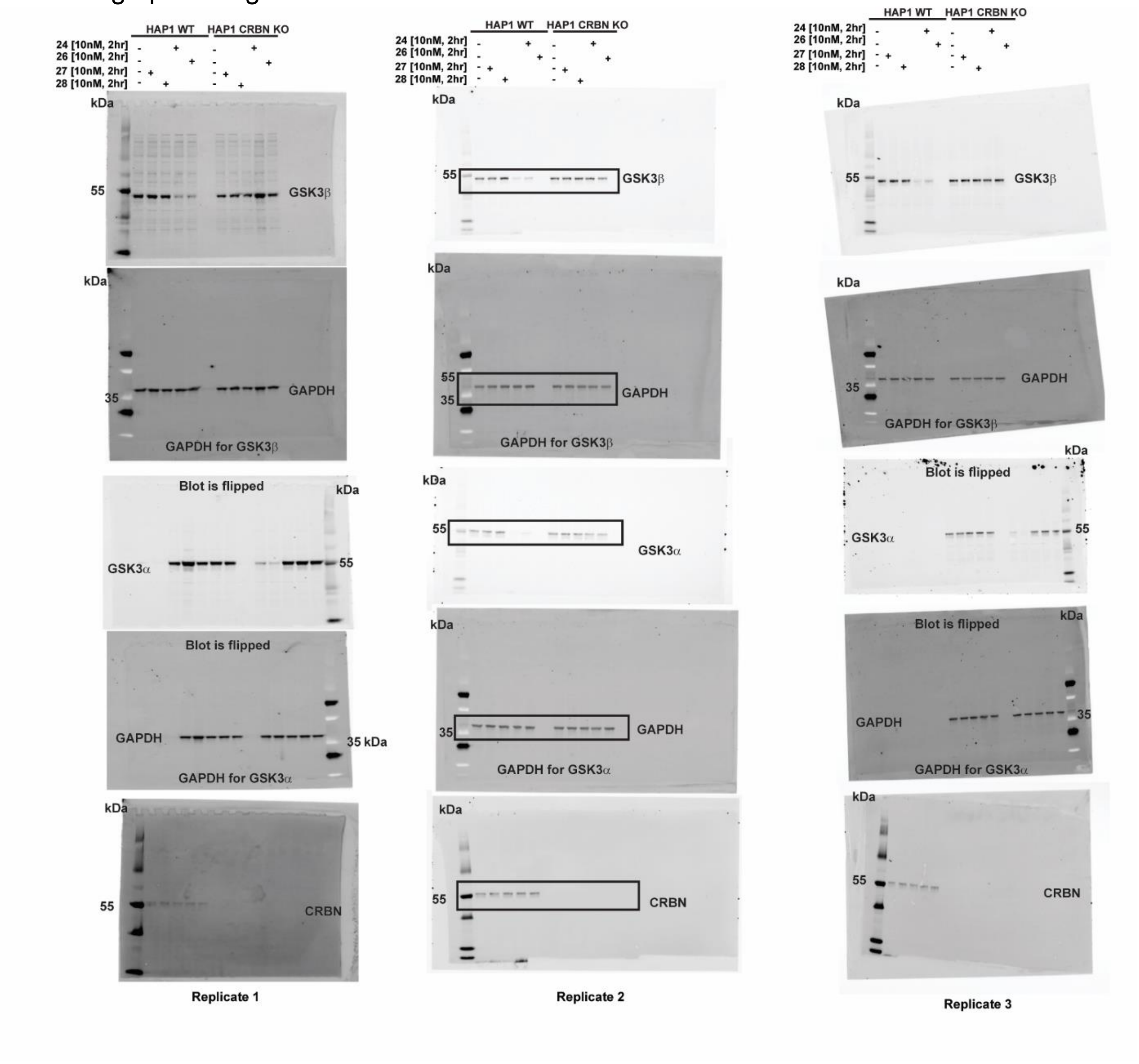

Figure S11. Uncropped Western blots and SDS-PAGE images of all biological replicates for Figure S4i. Boxes indicate the regions shown in the figure.

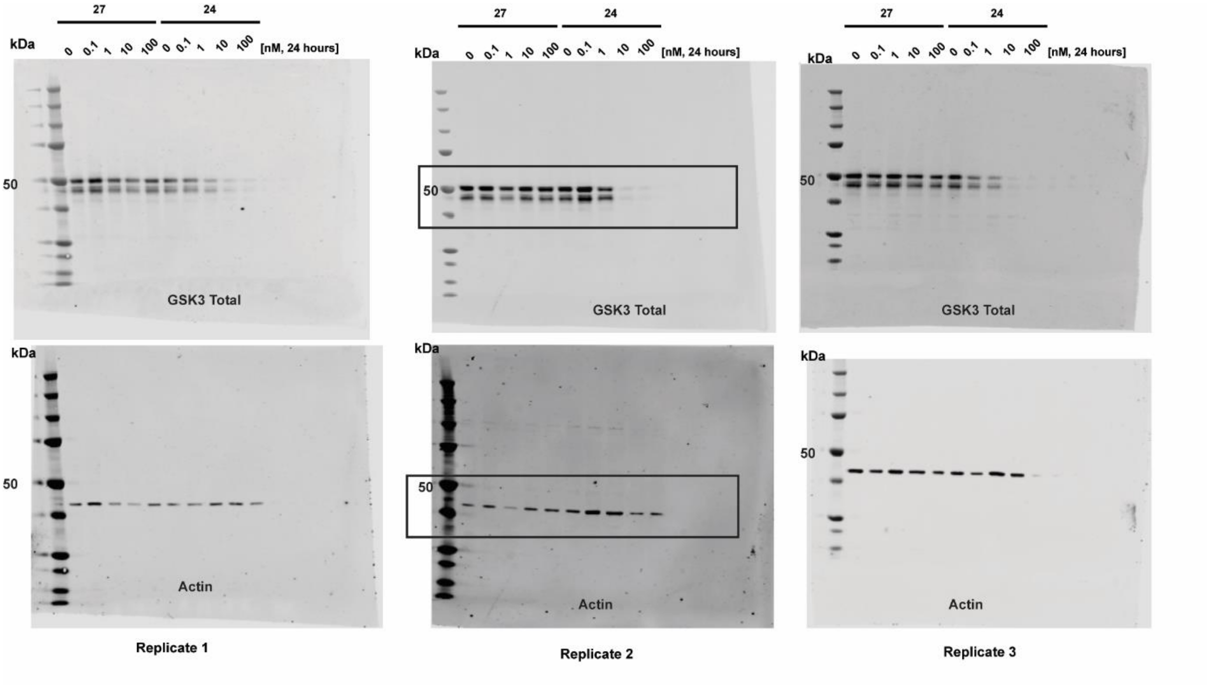

Figure S12. Uncropped Western blots and SDS-PAGE images of Figure S5c. Boxes indicate the regions shown in the figure. Same samples for the replicate run through parallel gels.

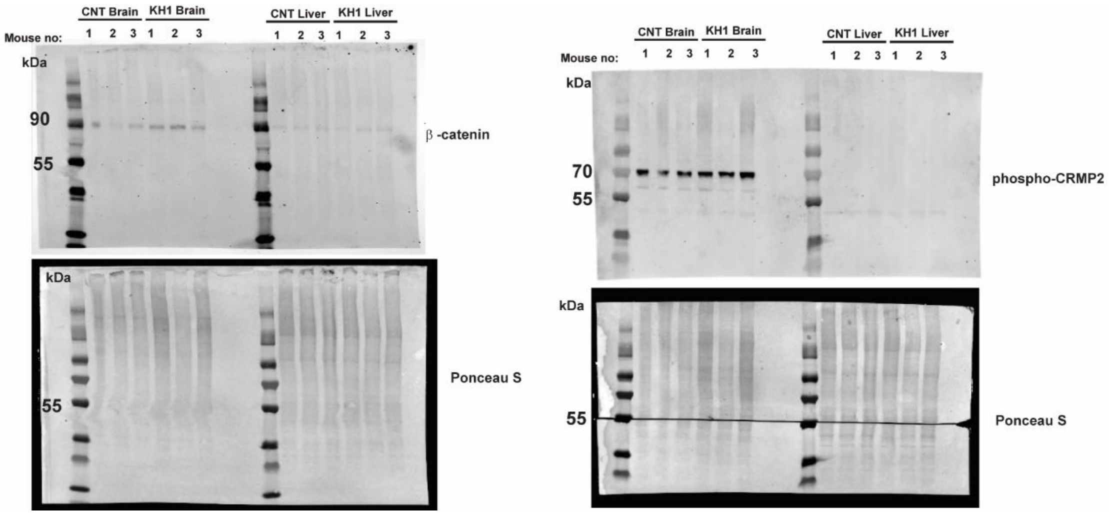

Supplementary Methods

Experimental Details of Linker Related Building Blocks

Reagents and starting materials that are commercially available were purchased from biotechne, Apollo Scientific, Sigma Aldrich, Fluorochem, TCI and ChemBridge at reagent grade and were used as received. Anhydrous DCM, DMF, dioxane, MeOH, and THF, purchased from Thermo Scientific, were used for synthesis. Solvents for work-up, purification and preparative HPLC were HPLC grade. Thin layer chromatography (TLC) was performed on pre-coated plates (Silica gel 60 F254, Merck) and visualised via UV light (UV 254 and/or 365 nm). Flash column chromatography was performed using a Teledyne Isco Combiflash Rf with prepacked Redisep RF Normal phase disposable columns (230 – 400 mesh, 40 – 63 mm: SiliCycle). All ^{1}H and ^{13}C NMR spectra were recorded at ambient temperature on Bruker Ascend 500 MHz spectrometers. Chemical shifts (δ/ppm) were referenced to the residual solvent peak in ^{1}H (7.26 ppm for $CDCl_3$ and 2.50 ppm for d-DMSO) and ^{13}C spectra (77.16 ppm for $CDCl_3$ and 39.52 ppm for d-DMSO). Coupling constants (J) are given in Hz. Signal splitting patterns are described as singlet (s), doublet (d), triplet (t), quartet (q), doublet of doublets (dd), doublet of triplets (dt), triplet of doublets (td), muLtiplet (m), broad (br) or a combination thereof. All standard NMR experiments were performed at 298 K and recorded on 500 MHz Bruker Avance NEO with a BBFO SmartProbe. High-resolution mass spectrometry (HR-MS) was performed on a Bruker MicroTOF II focus ESI mass spectrometer connected in parallel to a Dionex uLtimate 3000 RSLC system with a diode array detector and a Waters XBridge C18 column (50 mm × 2.1 mm, 3.5 µm particle size). Samples were eluted with a 6 min gradient of 5–95% acetonitrile: water containing 0.1% formic acid at a flow rate of 0.6 mL/min. Liquid chromatography-mass spectrometry (LC-MS) was carried out on a Shimadzu HPLC/MS 2020 equipped with a Hypersil Gold column (1.9 µm particle size, 50 × 2.1 mm), photodiode array detector and ESI detector. Samples were eluted with either a 3 min or 5 min gradient of 5–95% acetonitrile:water containing 0.1% formic acid at a flow rate of 0.7 mL/min.

All compounds are >95% pure by HPLC unless stated otherwise.

General procedures for linker synthesis.

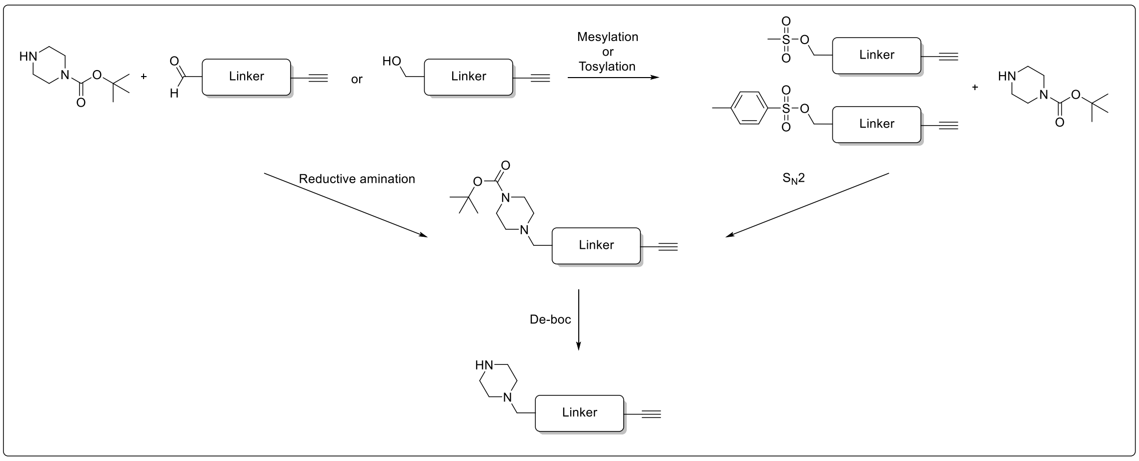

Figure S13: General procedures for linker synthesis.

1-(Hex-5-yn-1-yl)piperazine (1)

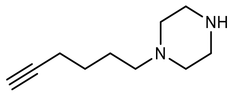

Synthesised according to the published procedure (Nature Chemistry (2024), 16(2), 183-192).

(ES+): 167 $[M+H^+]^+$.

^{1}H NMR (500 MHz, $CDCl_3$) δ 2.89 (t, *J* = 4.9 Hz, 4H), 2.40 (s, 4H), 2.35 – 2.30 (m, 2H), 2.21 (td, *J* = 6.9, 2.7 Hz, 2H), 1.94 (t, *J* = 2.6 Hz, 1H), 1.65 – 1.59 (m, 2H), 1.55 (m, 2H).

^{13}C NMR (126 MHz, $CDCl_3$) δ 84.3, 68.3, 58.6, 54.6, 46.1, 26.4, 25.6, 18.3.

1-((6-Ethynylpyridin-3-yl)methyl)piperazine: (2)

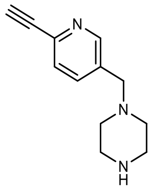

Synthesised according to the published procedure (CN113683611 A).

^{1}H NMR (500 MHz, $CDCl_3$) δ 8.67 (d, *J* = 2.1 Hz, 1H), 7.74 (dd, *J* = 8.1, 2.1 Hz, 1H), 7.40 (d, *J* = 8.0 Hz, 1H), 3.65 (s, 2H), 3.19 (s, 1H), 2.97 – 2.90 (m, 4H), 2.53 – 2.44 (m, 4H).

^{13}C NMR (126 MHz, $CDCl_3$) δ 158.9, 152.7, 139.9, 122.9, 117.9, 80.9, 80.6, 65.3, 54.8, 46.3.

HRMS (ESI+) (*m/z*): calculated for $[(C12H15N3)+H^+]^+$: 202.13388, found: 202.13336

***tert*-Butyl 4-(4-(prop-2-yn-1-yloxy)benzyl)piperazine-1-carboxylate (30)**

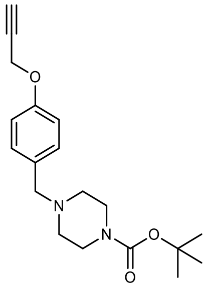

To a stirred solution of 4-(pent-4-yn-1-yloxy)benzaldehyde (430 mg, 2.7 mmol) in DCM (5.4 mL) at 0 °C was added *tert*-butyl piperazine-1-carboxylate (547 mg, 2.9 mmol) and acetic acid (0.2 mL, 2.9 mmol). The reaction was then stirred at 0 °C for 1 hour. To the solution was then added STAB (1.7 g, 8.0 mmol) in several portions over 30 minutes. The reaction mixture was ambient temperature overnight. The reaction was quenched by addition of saturated aqueous sodium hydrogen carbonate solution, and the organic phase was separated. The aqueous component was extracted with DCM, and the combined organic extracts were dried over anhydrous magnesium sulfate and concentrated under reduced pressure. Purification by flash column chromatography, eluting with 0-40% EtOAc in heptane, afforded the title compound as a crude and taken directly forward to the next step.

(ES+): 331 $[M+H^+]^+$.

1-[(4-Prop-2-ynoxyphenyl)methyl]piperazine (3)

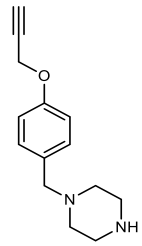

To a stirred solution of **30** (880 mg, 2.7 mmol) from previous reaction in DCM (3.3 mL) at 0 °C was added TFA (1.5 mL, 20.1 mmol) and the reaction was stirred overnight before being concentrated under reduced pressure. The residue was taken up in MeCN/Water (4 mL; 1:1), and purification by reverse-phase flash column chromatography, eluting with 0-60% aq 0.1% formic acid in MeCN, afforded the title compound as a white solid as a white solid (430 mg, 70%).

^{1}H NMR (500 MHz, $CDCl_3$) δ 7.24 (d, *J* = 8.7 Hz, 2H), 6.92 (d, *J* = 8.7 Hz, 2H), 4.68 (d, *J* = 2.4 Hz, 2H), 3.43 (s, 2H), 2.88 (t, *J* = 4.9 Hz, 4H), 2.51 (t, *J* = 2.4 Hz, 1H), 2.40 (br, 4H).

^{13}C NMR (126 MHz, $CDCl_3$) δ 156.8, 130.4, 115.2, 114.7, 78.7, 75.5, 62.8, 55.9, 53.6, 45.6.

HRMS (ESI+) (*m/z*): calculated for $[(C14H18N2O)+H^+]^+$: 231.14919, found: 231.14940

***tert*-Butyl 4-(4-(pent-4-yn-1-yloxy)benzyl)piperazine-1-carboxylate (31)**

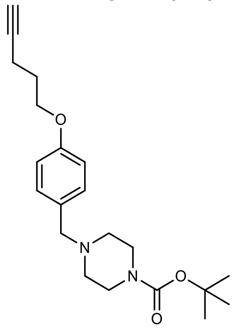

To a stirred solution of 4-(pent-4-yn-1-yloxy)benzaldehyde (940 mg, 5.0 mmol) in DCM (10.0 mL) at 0 °C was added *tert*-butyl piperazine-1-carboxylate (1.0 g, 5.5 mmol) and acetic acid (0.3 mL, 5.5 mmol). The reaction was then stirred at 0 °C for 1 hour. To the solution was then added STAB (3.2 g, 15.0 mmol) in several portions over 30 minutes. The reaction mixture was ambient temperature overnight. The reaction was quenched by addition of saturated aqueous sodium hydrogen carbonate solution and the organic phase was separated. The aqueous component was extracted with DCM, and the combined organic extracts were dried over anhydrous magnesium sulfate and concentrated under reduced pressure. Purification by flash column chromatography, eluting with 0-40% EtOAc in heptane, afforded the title compound as a colourless oil (558 mg, 31%).

^{1}H NMR (500 MHz, CDCl3) δ 7.20 (d, J = 8.5 Hz, 2H), 6.85 (d, J = 8.6 Hz, 2H), 4.05 (t, J = 6.1 Hz, 2H), 3.47 (s, 2H), 3.42 (t, J = 5.1 Hz, 4H), 2.43 – 2.37 (m, 6H), 2.03 – 1.98 (m, 2H), 1.96 (t, J = 2.7 Hz, 1H), 1.45 (s, 9H).

^{13}C NMR (126 MHz, $CDCl_3$) δ 175.0, 158.4, 154.9, 130.7, 129.4, 114.4, 83.6, 79.8, 69.0, 66.3, 62.3, 52.7, 28.6, 28.3, 15.3.

HRMS (ESI+) (*m/z*): calculated for $[(C21H30N2O3)+H^+]^+$: 359.23292, found: 359.23348.

1-[(4-Pent-4-ynoxyphenyl)methyl]piperazine (4)

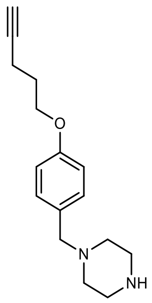

To a stirred solution of **31** from previous reaction (560 mg, 1.6 mmol) in DCM (3.9 mL) at ambient temperature was added HCl (3.9 mL, 15.6 mmol, 4M solution in 1,4-dioxane) and the reaction was stirred overnight before being concentrated under reduced pressure. The residue was taken up in MeCN/Water (4 mL; 1:1), and purification by reverse-phase flash column chromatography, eluting with 0-60% aq. 0.1% formic acid in MeCN, afforded the title compound as a white solid as a white solid (340 mg, 85%).

^{1}H NMR (500 MHz, CDCl3) δ 7.18 (d, *J* = 8.4 Hz, 2H), 6.85 (d, *J* = 8.4 Hz, 2H), 4.05 (t, *J* = 6.1 Hz, 2H), 3.49 (s, 2H), 3.17 (t, *J* = 5.1 Hz, 4H), 2.71 (t, *J* = 5.1 Hz, 4H), 2.40 (td, *J* = 7.0, 2.7 Hz, 2H), 2.00 (q, *J* = 6.5 Hz, 2H), 1.96 (t, *J* = 2.7 Hz, 1H).

^{13}C NMR (126 MHz, CDCl3) δ 158.8, 130.7, 114.9, 83.9, 69.3, 66.6, 62.2, 50.2, 44.2, 28.6, 15.6. N.B. 1x remaining quaternary carbon observed in HMBC at 129.44. (See Supplementary spectrum 17).

HRMS (ESI+) (*m/z*): calculated for [(C16H22N2O)+H$^+$]$^+$: 259.18049, found: 259.18094

tert-Butyl 4-(prop-2-yn-1-yloxy)piperidine-1-carboxylate (32)

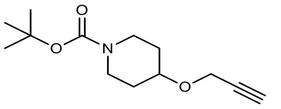

To a stirred solution of *tert*-butyl 4-hydroxypiperidine-1-carboxylate (500 mg, 2.5 mmol) at 0 °C in DMF (1.0 mL) was added sodium hydride (109 mg, 2.7 mmol, 60% dispersion in mineral oil) and stirred for 10 minutes. To the solution was then added propargyl bromide (0.2 mL, 2.0 mmol) at 0 °C dropwise. The reaction was quenched by addition of saturated aqueous ammonium chloride solution, and the organic phase was separated. The aqueous component was extracted with diethyl ether, and the combined organic extracts were dried over anhydrous magnesium sulfate and concentrated under reduced pressure. Purification by flash column chromatography, eluting with 0-50% EtOAc in heptane, afforded the title compound as a yellow oil (400 mg, 67%).

^{1}H NMR (500 MHz, $CDCl_3$) δ 4.19 (d, *J* = 2.4 Hz, 2H), 3.80 – 3.73 (m, 2H), 3.73 – 3.67 (m, 1H), 3.10 (m, 2H), 2.41 (t, *J* = 2.4 Hz, 1H), 1.88 – 1.82 (m, 2H), 1.56 – 1.50 (m, 2H), 1.45 (s, 9H).

4-(Prop-2-yn-1-yloxy)piperidine (5)

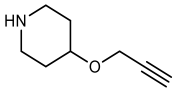

To a stirred solution of **32** (400 mg, 1.7 mmol) at 0 °C in DCM (8.4 mL) was added HCl (4.2 mL, 16.7 mmol, 4M solution in 1,4-dioxane) and the reaction was stirred overnight before being concentrated under reduced pressure to afford the title compound (220 mg, 94%).

^{1}H NMR (500 MHz, $CDCl_3$) δ 9.52 (s, 2H), 4.17 (d, *J* = 2.4 Hz, 2H), 3.94 – 3.88 (m, 1H), 3.35 – 3.24 (m, 2H), 3.24 – 3.14 (m, 2H), 2.43 (t, *J* = 2.4 Hz, 1H), 2.20 – 2.10 (m, 2H), 2.07 – 1.98 (m, 2H).

^{13}C NMR (126 MHz, $CDCl_3$) δ 79.8, 75.1, 69.4, 56.2, 40.2, 27.3.

HRMS (ESI+) (*m/z*): calculated for [(C8H13NO)+H$^+$]$^+$: 140.10699, found: 140.10718

***tert*-Butyl 6-(4-(prop-2-yn-1-yloxy)benzyl)-2,6-diazaspiro[3.4]octane-2-carboxylate (33)**

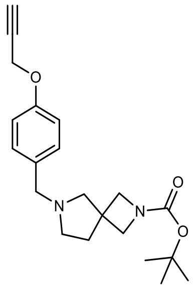

To a stirred solution of 4-(prop-2-yn-1-yloxy)benzaldehyde (1.0 g, 6.2 mmol) in DCM (12.5 mL) at 0 °C was added *tert*-butyl 2,6-diazaspiro[3.4]octane-2-carboxylate (1.5 g, 6.9 mmol) and acetic acid (0.4 mL, 6.9 mmol). The reaction was then stirred at 0 °C for 1 hour. To the solution was then added STAB (4.0 g, 18.9 mmol mmol) in several portions over 30 minutes, and the reaction mixture was stirred at ambient temperature overnight. The reaction was quenched by addition of saturated aqueous sodium hydrogen carbonate solution and the organic phase was separated. The aqueous component was extracted with DCM, and the combined organic extracts were dried over anhydrous magnesium sulfate and concentrated under reduced pressure. Purification by flash column chromatography, eluting with 0-40% EtOAc in heptane, afforded the title compound as a colourless oil (800 mg, 35%).

^{1}H NMR (500 MHz, $CDCl_3$) δ 7.22 (d, *J* = 8.6 Hz, 2H), 6.92 (d, *J* = 8.6 Hz, 2H), 4.68 (d, *J* = 2.4 Hz, 2H), 3.83 – 3.79 (m, 4H), 3.52 (s, 2H), 2.65 (s, 2H), 2.56 (t, *J* = 7.1 Hz, 2H), 2.52 (t, *J* = 2.4 Hz, 1H), 2.03 (t, *J* = 7.1 Hz, 2H), 1.42 (s, 9H).

^{13}C NMR (126 MHz, $CDCl_3$) δ 157.0, 156.7, 132.4, 130.2, 115.1, 79.7, 79.1, 75.8, 65.7, 62.1, 59.9, 56.2, 54.0, 39.6, 37.6, 28.8.

HRMS (ESI+) (*m/z*): calculated for [(C21H28N2O3)+H$^+$]$^+$: 357.21727, found: 357.21785

6-(4-(Prop-2-yn-1-yloxy)benzyl)-2,6-diazaspiro[3.4]octane (6)

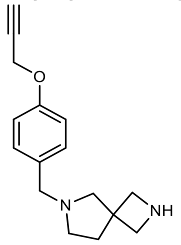

To a stirred solution of **33** (580 mg, 1.6 mmol) in DCM (2.0 mL) at ambient temperature was added TFA (0.9 mL, 12.0 mmol), and the reaction was stirred overnight before being concentrated under reduced pressure. The residue was taken up in MeCN/Water (4 mL; 1:1), and purification by reverse-phase flash column chromatography, eluting with 0-60% aq 0.1% formic acid in MeCN, afforded the title compound as a white solid (210 mg, 51%).

^{1}H NMR (500 MHz, $CDCl_3$) δ 7.22 (d, *J* = 8.5 Hz, 2H), 6.92 (d, *J* = 8.5 Hz, 2H), 4.67 (d, *J* = 2.4 Hz, 2H), 3.64 – 3.55 (m, 4H), 3.52 (s, 2H), 2.72 (s, 2H), 2.54 – 2.48 (m, 3H), 2.04 (t, *J* = 7.0 Hz, 2H).

^{13}C NMR (126 MHz, $CDCl_3$) δ 156.6, 132.1, 130.0, 114.7, 78.7, 75.4, 65.6, 59.9, 59.7, 55.9, 53.7, 44.6, 37.5.

HRMS (ESI+) (*m/z*): calculated for [(C16H20N2O)+H$^+$]$^+$: 257.16484, found: 257.16513

***tert*-Butyl 4-(hept-6-yn-1-yl)piperazine-1-carboxylate (34)**

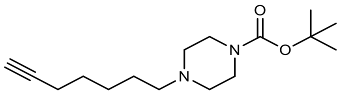

To a stirred solution of hept-6-yn-1-yl methanesulfonate (950 mg, 5.0 mmol) in MeCN (20.0 mL) was added *tert*-butyl piperazine-1-carboxylate (1.1 g, 6.0 mmol) and Et_3N (1.4 mL, 10.0 mmol) and the reaction was heated to 90 °C and stirred overnight before being concentrated under reduced pressure. Purification by flash column chromatography, eluting with 0-50% EtOAc in heptane, afforded the title compound as a crude and taken directly forward to the next step.

^1H NMR (500 MHz, $CDCl_3$) δ 3.43 (t, *J* = 5.1 Hz, 4H), 2.43 – 2.30 (m, 6H), 2.18 (td, *J* = 7.1, 2.7 Hz, 2H), 1.93 (t, *J* = 2.6 Hz, 1H), 1.58 – 1.48 (m, 4H), 1.45 (s, 9H), 1.44 – 1.38 (m, 2H).

1-(Hept-6-yn-1-yl)piperazine (7)

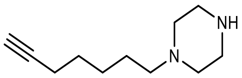

To a stirred solution of **34** (800 mg, 2.9 mmol) from previous reaction in DCM (3.6 mL) at 0 °C was added HCl (7.1 mL, 29.0 mmol, 4M solution in 1,4-dioxane) and the reaction was stirred overnight before being concentrated *in vacuo* to afford the title compound as a white solid (620 mg, 99%).
^1H NMR (500 MHz, $CDCl_3$) δ 2.90 – 2.87 (m, 4H), 2.45 – 2.34 (m, 4H), 2.33 – 2.28 (m, 2H), 2.18 (td, *J* = 7.0, 2.6 Hz, 2H), 1.93 (t, *J* = 2.6 Hz, 1H), 1.66 – 1.61 (m, 1H), 1.57 – 1.47 (m, 4H), 1.44 – 1.37 (m, 2H).

^{13}C NMR (126 MHz, $CDCl_3$) δ 84.7, 68.3, 59.4, 54.8, 46.3, 28.6, 26.9, 26.3, 18.5.

HRMS (ESI+) (*m/z*): calculated for [(C11H20N2)+H$^+$]$^+$: 181.16993, found: 181.16986

2-(Chloromethyl)-6-ethynylpyridine (35)

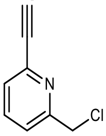

To stirred a solution of (6-ethynylpyridin-2-yl)methanol (450 mg, 3.0 mmol) in DCM (7.5 mL) at -78 °C under nitrogen atmosphere added methanesulfonyl chloride (0.8 mL, 10.0 mmol) and stirred for 1 hour. The reaction was quenched by addition of saturated aqueous sodium hydrogen carbonate solution (5 mL) and the organic phase was separated. The aqueous component was extracted with DCM, and the combined organic extracts were dried over anhydrous magnesium sulfate and concentrated under reduced pressure. Purification by flash column chromatography, eluting with 0-100% EtOAc in heptane, afforded the title compound as a dark red oil (230 mg, 32%).

(ES+): 152 [M+H$^+$]$^+$.

^1H NMR (500 MHz, $CDCl_3$) δ 7.72 (t, *J* = 7.5 Hz, 1H), 7.50 (d, *J* = 7.5 Hz, 1H), 7.43 (d, *J* = 7.5 Hz, 1H), 4.67 (s, 2H), 3.18 (s, 1H).

^{13}C NMR (126 MHz, $CDCl_3$) δ 157.4, 141.9, 137.5, 126.8, 122.7, 82.5, 77.8, 46.4.

***tert*-Butyl 4-((6-ethynylpyridin-2-yl)methyl)piperazine-1-carboxylate (36)**

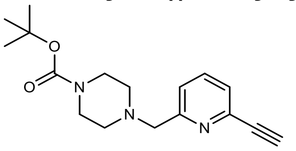

To a stirred solution of **35** (230 mg, 1.1 mmol) in DMF (2.2 mL) was added potassium iodide (36 mg, 0.2 mmol) and *tert*-butyl piperazine-1-carboxylate (407 mg, 2.2 mmol) and the reaction was heated to 80 °C and stirred overnight. The reaction was taken up in EtOAc (50 mL) and the organics washed with water (2 x 40 mL) and once with brine (40 mL) and the combined organic extracts were dried over anhydrous magnesium sulfate and concentrated under under reduced pressure. Purification by flash column chromatography, eluting with 0-100% EtOAc in heptane, afforded the title as a dark solid (190 mg, 58%).

(ES+): 302 $[M+H^+]^+$.

^1H NMR (500 MHz, $CDCl_3$) δ 7.64 (t, *J* = 7.7 Hz, 1H), 7.47 (d, *J* = 7.7 Hz, 1H), 7.38 (d, *J* = 7.7 Hz, 1H), 3.68 (s, 2H), 3.47 – 3.41 (m, 4H), 3.14 (s, 1H), 2.48 – 2.40 (m, 4H), 1.45 (s, 9H).

^{13}C NMR (126 MHz, $CDCl_3$) δ 159.7, 155.2, 142.0, 137.0, 126.3, 123.3, 83.4, 80.0, 77.6, 64.7, 60.8, 53.4, 28.9.

1-((6-Ethynylpyridin-2-yl)methyl)piperazine (8)

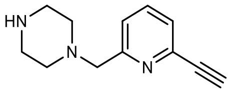

To a stirred solution of **36** (190 mg, 0.6 mmol) at ambient temperature in DCM (3.2 mL) was added HCl (4.7 mL, 19.0 mmol, 4M solution in 1,4-dioxane) and stirred for 3 hours before concentrated under reduced pressure. Purification by strong cation exchange (SCX) cartridge eluting with 3.5 N NH_3 in MeOH, afforded the title compound as a white solid (120 mg, 95%).

^1H NMR (500 MHz, $CDCl_3$) δ 7.63 (t, *J* = 7.8 Hz, 1H), 7.48 (d, *J* = 7.8 Hz, 1H), 7.36 (d, *J* = 7.8 Hz, 1H), 3.66 (s, 2H), 3.13 (s, 1H), 2.90 (t, *J* = 4.9 Hz, 4H), 2.48 (t, *J* = 4.7 Hz, 4H).

^{13}C NMR (126 MHz, $CDCl_3$) δ 159.9, 141.6, 136.7, 125.9, 123.0, 83.1, 77.2, 65.1, 54.8, 46.3.

HRMS (ESI+) (*m/z*): calculated for $[(C12H15N3)+H^+]^+$: 202.13388, found: 202.12694

***tert*-Butyl 4-hept-6-ynyl-1,4-diazepane-1-carboxylate (37)**

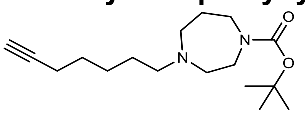

To a stirred solution of hept-6-yn-1-yl methanesulfonate (1.0 g, 5.3 mmol) in dry MeCN (21.0 mL) was added *tert*-butyl 1,4-diazepane-1-carboxylate (1.2 mL, 6.3 mmol) and Et_3N (1.5 mL, 10.5 mmol) and the reaction was heated to 90 °C and stirred overnight before being concentrated *in vacuo*. Filtration through a plug of silica and purification by flash column chromatography, eluting with 0-10% MeOH/DCM, afforded the crude title compound which was used directly in the next stage without further purification.

1-(Hept-6-yn-1-yl)-1,4-diazepane (9)

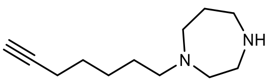

To a stirred solution of **37** (500 mg, 1.7 mmol) from previous reaction in DCM (4.0 mL) was added HCl (4.2 mL, 17.0 mmol, 4M solution in 1,4-dioxane) and the reaction was stirred overnight before being concentrated under reduced pressure. Purification by Strong cation exchange (SCX) cartridge eluting with 3.5 N NH_3 in MeOH, afforded the title compound as a colourless oil (120 mg, 95%).

^{1}H NMR (500 MHz, $CDCl_3$) δ 2.95 – 2.88 (m, 4H), 2.70 – 2.62 (m, 4H), 2.51 – 2.45 (m, 2H), 2.19 (td, *J* = 7.1, 2.7 Hz, 2H), 1.93 (t, *J* = 2.7 Hz, 1H), 1.76 (p, *J* = 6.0 Hz, 2H), 1.54 (dt, *J* = 14.4, 7.1 Hz, 2H), 1.51 – 1.45 (m, 2H), 1.44 – 1.35 (m, 2H).

^{13}C NMR (126 MHz, $CDCl_3$) δ 84.5, 68.1, 58.2, 58.2, 54.4, 48.7, 47.2, 30.3, 28.3, 27.0, 26.6, 18.3.

HRMS (ESI+) (*m/z*): calculated for [(C12H22N2)+H$^+$]$^+$: 195.18558, found: 195.18554

tert-Butyl 4-(2-(2-(prop-2-yn-1-yloxy)ethoxy)ethyl)piperazine-1-carboxylate (38)

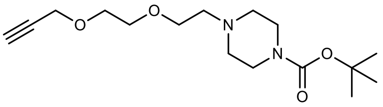

Synthesised according to the published procedure and used directly in the following stage without isolation. (WO2021067606 A1).

^{1}H NMR (500 MHz, $CDCl_3$) δ 4.17 (d, *J* = 2.5 Hz, 2H), 3.68 – 3.64 (m, 2H), 3.64 – 3.57 (m, 4H), 3.39 (t, *J* = 6.8 Hz, 5H), 2.58 (t, *J* = 5.8 Hz, 2H), 2.44 – 2.39 (m, 4H), 1.43 (s, 9H).

1-[2-(2-Prop-2-ynoxyethoxy)ethyl]piperazine (10)

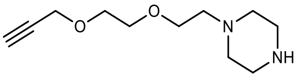

To a stirred solution of **38** (500 mg, 1.6 mmol) in DCM (2.0 mL) at ambient temperature was added TFA (1.8 mL, 24.0 mmol), and the reaction was stirred overnight before being concentrated under reduced pressure. The residue was taken up in MeCN/Water (4 mL; 1:1) and purification by reverse-phase flash column chromatography, eluting with 5-95% aq 0.1% formic acid in MeCN, afforded the title compound as a colourless oil (332 mg, 97%).

^{1}H NMR (500 MHz, $CDCl_3$) δ 4.17 (d, *J* = 2.4 Hz, 2H), 3.67 – 3.65 (m, 2H), 3.62 – 3.58 (m, 4H), 2.86 (t, *J* = 4.9 Hz, 4H), 2.55 (t, *J* = 6.0 Hz, 2H), 2.49 – 2.41 (m, 4H), 2.40 (t, *J* = 2.4 Hz, 1H), 2.01 (s, 1H).

^{13}C NMR (126 MHz, $CDCl_3$) δ 79.7, 74.6, 70.3, 69.2, 68.9, 58.5, 58.4, 55.0, 46.0.

tert-Butyl 6-(4-(prop-2-yn-1-yloxy)benzyl)-2,6-diazaspiro[3.3]heptane-2-carboxylate (39)

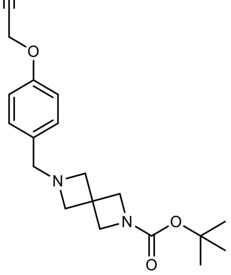

To a stirred solution of 4-(prop-2-yn-1-yloxy)benzaldehyde (1.0 g, 6.2 mmol) in DCM (12.5 mL) at 0 °C was added 2,6-diaza-spiro[3.3]heptane-2-carboxylic acid *tert*-butyl ester oxalate salt

(2.0 g, 6.9 mmol) and acetic acid (0.4 mL, 6.9 mmol). The reaction was then stirred at 0 °C for 1 hour. To the solution was added STAB (4.0 g, 18.7 mmol) in several portions over 30 minutes. The reaction mixture was ambient temperature overnight. The reaction was quenched by addition of saturated aqueous sodium hydrogen carbonate solution and the organic phase was separated. The aqueous component was extracted with DCM, and the combined organic extracts were dried over anhydrous magnesium sulfate and concentrated under reduced pressure. Purification by flash column chromatography, eluting with 0-100% EtOAc in heptane, afforded the title compound as a colourless oil (780 mg, 36%).
^{1}H NMR (500 MHz, $CDCl_3$) δ 7.17 (d, *J* = 8.6 Hz, 2H), 6.92 (d, *J* = 8.6 Hz, 2H), 4.67 (d, *J* = 2.4 Hz, 2H), 3.97 (s, 4H), 3.48 (s, 2H), 3.29 (s, 4H), 2.51 (t, *J* = 2.4 Hz, 1H), 1.42 (s, 9H).

^{13}C NMR (126 MHz, $CDCl_3$) δ 156.6, 156.0, 130.8, 129.6, 114.7, 79.3, 78.5, 75.3, 64.0, 62.8, 59.1 (br), 55.7, 33.3, 28.3.

HRMS (ESI+) (*m/z*): calculated for [(C20H26N2O3)+H$^+$]$^+$: 343.20162, found: 343.20227

2-(4-(Prop-2-yn-1-yloxy)benzyl)-2,6-diazaspiro[3.3]heptane (11)

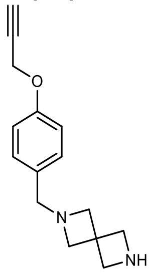

To a stirred solution of **39** (519 mg, 1.5 mmol) in DCM (1.9 mL) at ambient temperature was added TFA (0.9 mL, 11.4 mmol) and the reaction was stirred overnight before being concentrated under reduced pressure. The residue was taken up in MeCN/Water (4 mL; 1:1), and purification by reverse-phase flash column chromatography, eluting with 0-60% aq 0.1% formic acid in MeCN, afforded the title compound as a white solid as a white solid (230 mg, 63%).

^{1}H NMR (500 MHz, $CDCl_3$) δ 7.18 (d, *J* = 8.5 Hz, 2H), 6.91 (d, *J* = 8.5 Hz, 2H), 4.67 (d, *J* = 2.4 Hz, 2H), 3.73 (s, 4H), 3.48 (s, 2H), 3.30 (s, 4H), 2.51 (t, *J* = 2.5 Hz, 1H).

^{13}C NMR (126 MHz, $CDCl_3$) δ 156.7, 131.1, 129.6, 114.8, 78.7, 75.4, 64.7, 63.0, 57.5, 55.9, 38.7.

HRMS (ESI+) (*m/z*): calculated for [(C15H18N2O)+H$^+$]$^+$: 243.14919, found: 243.14925.

1-(Pent-4-yn-1-yl)piperazine (12)

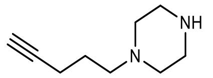

Synthesised according to the published procedure (WO2006105372 A2).

^{1}H NMR (500 MHz, $CDCl_3$) δ 2.91 – 2.87 (m, 4H), 2.46 – 2.39 (m, 6H), 2.22 (td, *J* = 7.2, 2.7 Hz, 2H), 1.93 (t, *J* = 2.7 Hz, 1H), 1.70 (p, *J* = 7.2 Hz, 2H).

^{13}C NMR (126 MHz, $CDCl_3$) δ 84.2, 68.4, 57.9, 54.3, 46.0, 25.6, 16.4.

Experimental Details of E3 Ligase Ligand Related Building Blocks

Synthesis of 1-[3-(Bromomethyl)phenyl]hexahydropyrimidine-2,4-dione (13)

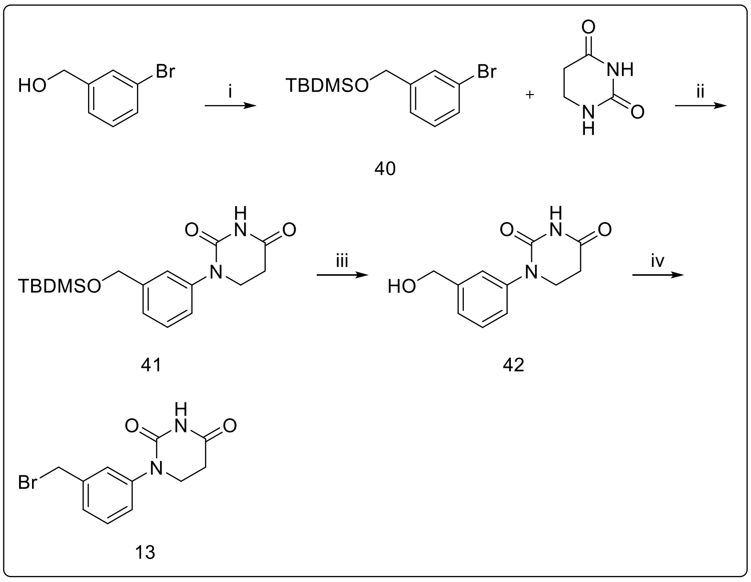

Figure S14: Synthetic route for 1-[3-(Bromomethyl)phenyl]hexahydropyrimidine-2,4-dione (13): (i) TBDMSCl, Imidazole, DMF; (ii) BrettPhos Pd G3, BrettPhos, Cs_2CO_3, 1,4-Dioxane, 100 °C; (iii) TBAF, DCM, 0 °C; (iv) HBr in water 90 °C

((3-Bromobenzyl)oxy)(*tert*-butyl)dimethylsilane (40)

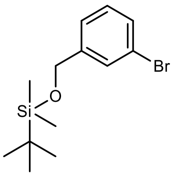

Synthesised according to the published procedure (CN117343081 A).

^{1}H NMR (500 MHz, $CDCl_3$) δ 7.48 (t, J = 1.7 Hz, 1H), 7.39 – 7.35 (m, 1H), 7.26 – 7.23 (m, 1H), 7.19 (t, J = 7.7 Hz, 1H), 4.71 (s, 2H), 0.95 (s, 9H), 0.11 (s, 6H).

^{13}C NMR (126 MHz, $CDCl_3$) δ 143.7, 129.8, 129.7, 128.9, 124.4, 122.3, 64.1, 25.8, 18.3, -5.4.

1-(3-(((*tert*-Butyldimethylsilyl)oxy)methyl)phenyl)dihydropyrimidine-2,4(1H,3H)-dione (41)

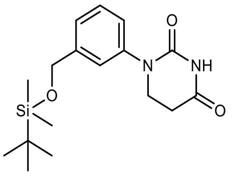

To a stirred solution of **40** (500 mg, 1.7 mmol) in dry 1,4-Dioxane (16.6 mL) at ambient temperature was added dihydropyrimidine-2,4(1H,3H)-dione (852 mg, 7.5 mmol), Cs_2CO_3 (1.6 mg, 5.0 mmol). The reaction vessel was then evacuated and backfilled with nitrogen three times. To the reaction was added BrettPhos Pd G3 (eq, 150 mg, 0.2 mmol) and BrettPhos (89 mg, 0.2 mmol) and the reaction vessel was sealed and left under inert atmosphere. The reaction was then stirred at 100 °C overnight before being filtered through cotton and filtrate was taken up in water (15 mL) and extracted with EtOAc, and the combined organic phase was separated. The aqueous component was extracted with EtOAc, and the combined organic extracts were dried over anhydrous magnesium sulfate and concentrated under reduced

pressure. Purification by flash column chromatography, eluting with 0-100% EtOAc in heptane, afforded the title compound as a colourless oil (335 mg, 60%).

^{1}H NMR (500 MHz, $CDCl_3$) δ 7.40 – 7.35 (m, 2H), 7.24 (d, *J* = 7.6 Hz, 1H), 7.21 – 7.16 (m, 1H), 4.75 (s, 2H), 3.88 (t, *J* = 6.6 Hz, 2H), 2.83 (t, *J* = 6.6 Hz, 2H), 0.95 (s, 9H), 0.11 (s, 6H).

^{13}C NMR (126 MHz, DMSO) δ 170.6, 152.1, 142.0, 141.8, 128.4, 123.7, 123.4, 122.8, 64.0, 44.5, 31.1., 25.8, 18.0, -5.3.

1-(3-(Hydroxymethyl)phenyl)dihydropyrimidine-2,4(1H,3H)-dione (42)

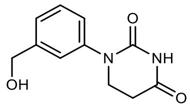

To a stirred solution of **41** (335 mg, 1.0 mmol) in dry THF (6.3 mL) at ambient temperature was added TBAF (1.7 mL, 1.7 mmol). The reaction was then stirred for 1 hour before concentrated under reduced pressure. Purification by flash column chromatography, eluting with 0-20% MeOH in DCM, afforded the title compound as a light yellow solid (197 mg, 89%).

^{1}H NMR (500 MHz, DMSO) δ 10.34 (s, 1H), 7.33 (t, *J* = 7.7 Hz, 1H), 7.27 (s, 1H), 7.20 – 7.16 (m, 2H), 5.24 (t, *J* = 5.7 Hz, 1H), 4.50 (d, *J* = 5.7 Hz, 2H), 3.77 (t, *J* = 6.6 Hz, 2H), 2.70 (t, *J* = 6.6 Hz, 2H).

HRMS (ESI+) (*m/z*): calculated for $[(C11H12N2O3)+H^+]^+$: 221.09207, found: 221.09175

1-[3-(Bromomethyl)phenyl]hexahydropyrimidine-2,4-dione (13)

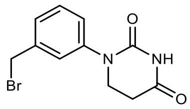

To a stirred solution of solution of 48% wt. hydrobromic acid in water (4.8 mL) was added **42** (150 mg, 0.7 mmol) at ambient temperature. The reaction was then stirred at 90 °C for 1 hour. The reaction was then taken up in water (10 mL) and extracted with EtOAc, and the combined organic extracts were dried over anhydrous magnesium sulfate and concentrated under reduced pressure to afford title compound as a brown solid (125 mg, 65%).

^{1}H NMR (500 MHz, DMSO) δ 10.41 (s, 1H), 7.42 (s, 1H), 7.38 (t, *J* = 7.8 Hz, 1H), 7.33 – 7.26 (m, 2H), 4.71 (s, 2H), 3.79 (t, *J* = 6.6 Hz, 2H), 2.71 (t, *J* = 6.6 Hz, 2H).

^{13}C NMR (126 MHz, DMSO) δ 170.6, 152.1, 142.2, 138.6, 129.0, 126.7, 125.9, 125.2, 44.5, 34.1, 31.1.

HRMS (ESI+) (*m/z*): calculated for $[(C11H11BrN2O)+H^+]^+$: 283.00767, found: 283.00807

(2S,4R)-1-((S)-2-(2-Bromoacetamido)-3,3-dimethylbutanoyl)-4-hydroxy-N-(4-(4-methylthiazol-5-yl)benzyl)pyrrolidine-2-carboxamide (14)

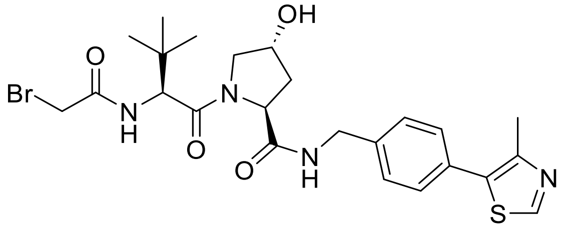

Synthesised according to the published procedure (WO2022125804 A1).

LCMS: Mass: 551.5 [M+H$^+$]: 552.

Synthesis of 4-(Bromomethyl)-N-(2,6-dioxopiperidin-3-yl)-2-fluorobenzamide (15)

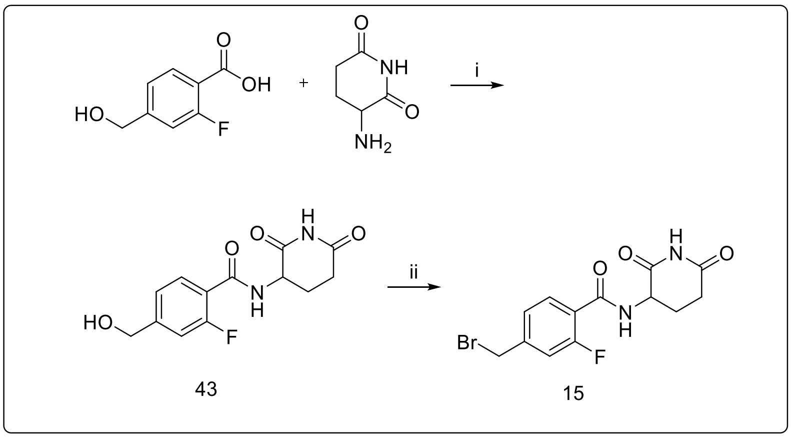

Figure S15: Synthetic route for 4-(Bromomethyl)-N-(2,6-dioxopiperidin-3-yl)-2-fluorobenzamide (15): (i) HATU, DIPEA, DMF; (ii) PBr_3, DCM, 0 °C

N-(2,6-Dioxopiperidin-3-yl)-2-fluoro-4-(hydroxymethyl)benzamide (43)

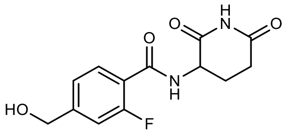

To a stirred solution of 2-fluoro-4-(hydroxymethyl)benzoic acid (400 mg, 2.4 mmol) in DMF (15.7 mL) at 0 °C was added DIPEA (1.6 mL, 9.4 mmol) dropwise and stirred for 5 minutes before addition of HATU (1.3 g, 3.6 mmol) and 3-aminopiperidine-2,6-dione hydrochloride (390 mg, 2.4 mmol) at ambient temperature. The reaction was then stirred overnight before concentrated under reduced pressure. Purification by reverse-phase flash column chromatography, eluting with 5-60% aq 0.1% formic acid in MeCN, afforded the title compound as an off-white solid (260 mg, 39%).

^{1}H NMR (500 MHz, DMSO) δ 10.85 (s, 1H), 8.48 (dd, *J* = 8.2, 3.5 Hz, 1H), 7.64 (t, *J* = 7.9 Hz, 1H), 7.26 – 7.17 (m, 2H), 5.43 (t, *J* = 5.8 Hz, 1H), 4.79 – 4.71 (m, 1H), 4.55 (d, *J* = 5.8 Hz, 2H), 2.82 – 2.72 (m, 1H), 2.56 – 2.52 (m, 1H), 2.15 – 2.04 (m, 1H), 2.03 – 1.95 (m, 1H).

^{13}C NMR (126 MHz, DMSO) δ 173.4, 172.4, 163.9, 160.9, 149.2, 130.6, 130.6, 122.4, 114.0, 113.8, 62.3, 50.1, 31.4, 24.5.

HRMS (ESI+) (*m/z*): calculated for [(C13H13FN2O4)+H$^+$]$^+$: 281.09322, found: 281.09354

4-(Bromomethyl)-N-(2,6-dioxopiperidin-3-yl)-2-fluorobenzamide (15)

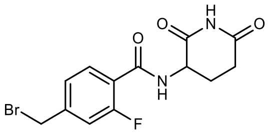

To a stirred solution of **43** (200 mg, 0.7 mmol) in dry DCM (4.8 mL) at 0 °C was added PBr_3 (140 µL, 1.4 mmol). The reaction was then stirred at ambient temperature overnight. The reaction was quenched by addition of saturated aqueous sodium hydrogen carbonate solution and the organic phase was separated. The aqueous component was extracted with DCM, and the combined organic extracts were dried over anhydrous magnesium sulfate and concentrated under reduced pressure to afford title compound as a white solid (190 mg, 78%).

^{1}H NMR (500 MHz, DMSO) δ 10.88 (s, 1H), 8.63 (dd, *J* = 8.3, 2.5 Hz, 1H), 7.65 (t, *J* = 7.7 Hz, 1H), 7.43 (dd, *J* = 11.2, 1.6 Hz, 1H), 7.39 (dd, *J* = 7.9, 1.6 Hz, 1H), 4.80 – 4.75 (m, 1H), 4.74 (s, 2H), 2.84 – 2.73 (m, 1H), 2.55 (t, *J* = 3.8 Hz, 1H), 2.15 – 2.04 (m, 1H), 2.05 – 1.97 (m, 1H).

^{13}C NMR (126 MHz, DMSO) δ 173.1, 172.0, 163.4, 160.1, 143.4, 130.8, 125.6, 123.3, 117.1, 116.9, 49.8, 32.5, 31.1, 24.2.

HRMS (ESI+) (*m/z*): calculated for [(C13H12BrFN2O3)+H$^+$]$^+$: 343.00881, found: 343.00949

Synthesis of 1-(6-(Bromomethyl)-1-methyl-1H-indazol-3-yl)dihydropyrimidine-2,4(1H,3H)-dione (16)

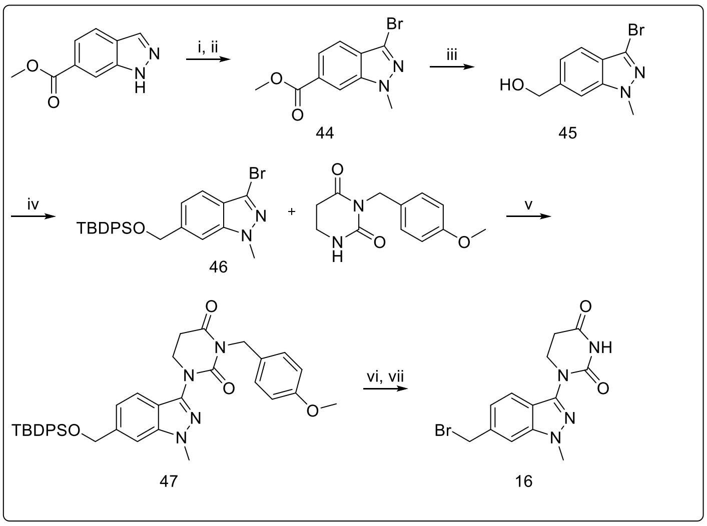

Figure S16: Synthetic route for 1-(6-(Bromomethyl)-1-methyl-1H-indazol-3-yl)dihydropyrimidine-2,4(1H,3H)-dione (16): (i) NBS, DMF; (ii) NaH, MeI, THF, 0°C; (iii) $LiAlH_4$, THF, -78°C; (iv) TBDPSCl, Imidazole, DCM, 0°C; (v) Cs_2CO_3, CuI, trans-N,N'-Dimethylcyclohexane-1,2-diamine, 1,4-Dioxane, 80° C; (vi) Triflic acid, TFA, 70°C; (vii) HBr in water, 90°C

Methyl 3-bromo-1-methyl-1H-indazole-6-carboxylate (44)

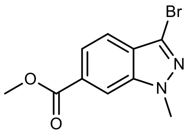

Synthesised according to the published procedure (CN115260180 A).

^{1}H NMR (500 MHz, $CDCl_3$) δ 8.16 (t, *J* = 1.1 Hz, 1H), 7.87 (dd, *J* = 8.5, 1.2 Hz, 1H), 7.66 (dd, *J* = 8.5, 1.2 Hz, 1H), 4.12 (s, 3H), 3.99 (s, 3H).

LCMS: Mass: 269.1 [M+H$^+$]: 271.

(3-Bromo-1-methyl-1H-indazol-6-yl)methanol (45)

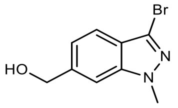

To a stirred solution of **44** (4.5 g, 16.5 mmol) in THF (82.7 mL) at -78 °C was added $LiAlH_4$ (33.1 mL, 33.1 mmol) slowly. The reaction was then stirred at -78 °C for 30 minutes before warming up to 0 °C. The reaction was quenched by adding Glauber's salt, then filtered and concentrated under reduced pressure. Purification by flash column chromatography, eluting with 0-60% EtOAc in heptane, afforded the title compound as a white solid (2.5 g, 62%).

(ES+): 241 [M+H$^+$]$^+$.

^{1}H NMR (500 MHz, $CDCl_3$) δ 7.54 (d, *J* = 8.4 Hz, 1H), 7.38 (s, 1H), 7.14 (dd, *J* = 8.4, 1.2 Hz, 1H), 4.85 (d, *J* = 5.8 Hz, 2H), 4.00 (s, 3H), 2.22 (t, *J* = 5.8 Hz, 1H).

^{13}C NMR (126 MHz, $CDCl_3$) δ 141.2, 140.9, 123.0, 120.4, 120.3, 119.6, 106.6, 65.0, 35.8.

3-bromo-6-(((*tert*-Butyldiphenylsilyl)oxy)methyl)-1-methyl-1H-indazole (46)

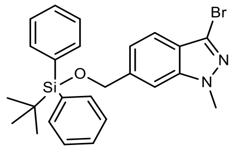

To a stirred solution of **45** (2.5 g, 10.2 mmol) in dry DCM (51.1 mL) at 0 °C was added imidazole (1.5 g, 22.5 mmol) and stirred for 5 minutes. To the stirred reaction was added
tert-butyldiphenylchlorosilane (2.9 mL, 11.2 mmol) dropwise and stirred at 0 °C for 1 hour before being concentrated under reduced pressure. The residue was taken up in EtOAc (30 mL) and washed once with water (20 mL x 2) and brine (20 mL x 1), and organic extracts were dried over anhydrous magnesium sulfate and concentrated under reduced pressure to afford afforded the title compound as a white solid (4.8 g, 99%).

^{1}H NMR (500 MHz, $CDCl_3$) δ 7.72 – 7.68 (m, 4H), 7.53 (d, *J* = 8.4 Hz, 1H), 7.46 – 7.41 (m, 2H), 7.40 – 7.36 (m, 5H), 7.09 (d, *J* = 8.4 Hz, 1H), 4.92 (s, 2H), 4.02 (s, 3H), 1.13 (s, 9H).

^{13}C NMR (126 MHz, $CDCl_3$) δ 141.2, 135.5, 134.7, 133.2, 129.7, 127.7, 122.7, 119.9, 119.9, 119.6, 105.9, 65.5, 35.7, 26.8, 19.2.

HRMS (ESI+) (*m/z*): calculated for [(C25H27BrN2OSi)+H$^+$]$^+$: 479.1149, found: 479.1154

1-(6-(((*tert*-Butyldiphenylsilyl)oxy)methyl)-1-methyl-1H-indazol-3-yl)-3-(4-methoxybenzyl)dihydropyrimidine-2,4(1H,3H)-dione (47)

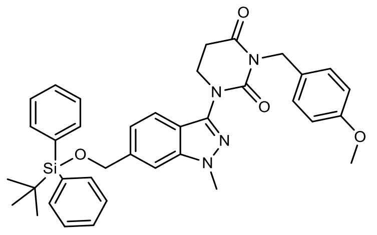

To a stirred solution of **46** (1.35 g, 2.8 mmol) in 1,4-Dioxane (11.7 mL) at ambient temperature was added 3-(4-methoxybenzyl)dihydropyrimidine-2,4(1H,3H)-dione (550 mg, 2.4 mmol), Cs_2CO_3 (1.5 g, 4.7 mmol), CuI (89 mg, 0.5 mmol) and trans-*N,N'*-dimethylcyclohexane-1,2-diamine (74 µL, 0.5 mmol). The reaction vessel was then evacuated and backfilled with nitrogen three times. The reaction was then stirred at 80 °C under inert atmosphere overnight. The reaction was filtered through cotton and concentrated under reduced pressure. Purification by flash column chromatography, eluting with 0-100% EtOAc in heptane, afforded the title compound as a colourless oil (1.2 g, 81%).

^{1}H NMR (500 MHz, $CDCl_3$) δ 7.73 – 7.69 (m, 4H), 7.56 (d, *J* = 8.4 Hz, 1H), 7.47 – 7.41 (m, 4H), 7.41 – 7.35 (m, 5H), 7.06 (dd, *J* = 8.4, 1.3 Hz, 1H), 6.85 – 6.81 (m, 2H), 5.02 (s, 2H), 4.90 (s, 2H), 4.00 (t, *J* = 6.7 Hz, 2H), 3.97 (s, 3H), 3.78 (s, 3H), 2.93 (t, *J* = 6.7 Hz, 2H), 1.12 (s, 9H).

^{13}C NMR (126 MHz, $CDCl_3$) δ 169.3, 159.1, 152.7, 142.2, 141.6, 140.9, 135.7, 133.5, 130.8, 130.0, 129.9, 127.9, 121.5, 119.6, 116.8, 113.9, 106.1, 65.8, 55.4, 43.7, 43.5, 35.5, 32.3, 27.0, 19.5.

HRMS (ESI+) (*m/z*): calculated for [(C37H40N4O4Si)+H^+]$^+$: 633.28916, found: 633.29022

1-(6-(Bromomethyl)-1-methyl-1H-indazol-3-yl)dihydropyrimidine-2,4(1H,3H)-dione (16)

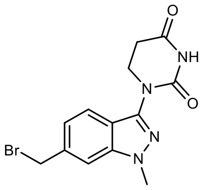

To a stirred solution of **47** (2.4 g, 3.8 mmol) in TFA (54.2 mL) at ambient temperature was added trifluoromethanesulfonic acid (5.0 mL, 56.9 mmol) slowly and heated to 60 °C for 5 hours before concentrated under reduced pressure. Purification by flash column chromatography, eluting with 0-15% MeOH in DCM. The collected residue was taken up in a solution of 48% wt. hydrobromic acid in water (17.0 mL, 666.0 mmol) and stirred at 90 °C for 30 minutes. The reaction was then taken up in water (10 mL) and extracted with EtOAc, and the combined organic extracts were dried over anhydrous magnesium sulfate and concentrated under reduced pressure. Purification by reverse-phase flash column chromatography, eluting with 5-95% aq 0.1% formic acid in MeCN, afforded the title compound as a light brown solid (234 mg, 18%).
^1H NMR (500 MHz, DMSO) δ 10.58 (s, 1H), 7.72 (s, 1H), 7.64 (d, J = 8.4 Hz, 1H), 7.18 (d, J = 8.4 Hz, 1H), 4.85 (s, 2H), 3.98 (s, 3H), 3.92 (t, J = 6.6 Hz, 2H), 2.76 (t, J = 6.6 Hz, 2H).

13C NMR (126 MHz, DMSO) δ 170.6, 151.9, 141.8, 140.5, 136.6, 122.2, 121.7, 116.8, 110.2, 44.0, 35.4, 35.1, 31.0.

HRMS (ESI+) (*m/z*): calculated for [(C13H13BrN4O2)+H^+]$^+$: 337.02947, found: 337.03022

Synthesis of 5-(Bromomethyl)-*N*-(2,6-dioxopiperidin-3-yl)picolinamide (17)

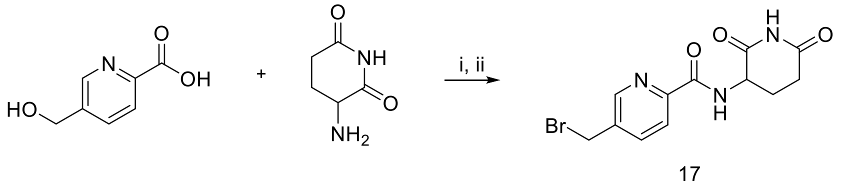

Figure S17: Synthetic route for 5-(Bromomethyl)-*N*-(2,6-dioxopiperidin-3-yl)picolinamide (17): (i) HATU, DIPEA, DMF; (ii) CBr_4, PPh_3, THF

5-(Bromomethyl)-*N*-(2,6-dioxopiperidin-3-yl)picolinamide (17)

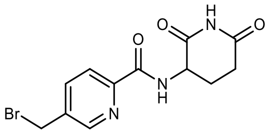

To a stirred solution of 3-aminopiperidine-2,6-dione hydrochloride (1.3 g, 8.2 mmol) in dry DMF (40.81 mL) at 0 °C was added DIPEA (5.7 mL, 32.7 mmol) dropwise and stirred for 5 minutes before addition of HATU (4.4 g, 11.4 mmol) and 5-(hydroxymethyl)picolinic acid (1.0 g, 6.5 mmol) at ambient temperature. The reaction was then stirred overnight before concentrated under reduced pressure and loaded on to an SCX-cartridge eluting with 3.5 N NH_3 in MeOH and the elute was concentrated under reduced pressure. Purification by reverse-

phase flash column chromatography, eluting with 5-60% aq 0.1% formic acid in MeCN, and the collected residue was taken up in THF (75.97 mL) and stirred. To this stirred solution was added CBr_4 (3.0 g, 9.1 mmol) and PPh_3 (1.6 g, 6.1 mmol) at ambient temperature. The reaction was then stirred for 30 minutes before being concentrated under reduced pressure. The residue was taken up in ice-cold EtOAc and filtered and the filter-cake was dried under vacuum. Purification by flash column chromatography, eluting with 0-30% Acetone in DCM, afforded the title compound as a white solid (320 mg, 15%).

^{1}H NMR (500 MHz, DMSO) δ 10.87 (s, 1H), 9.10 (d, J = 8.5 Hz, 1H), 8.75 (s, 1H), 8.13 – 8.02 (m, 2H), 4.84 (s, 2H), 4.83 – 4.75 (m, 1H), 2.86 – 2.76 (m, 1H), 2.57 – 2.52 (m, 1H), 2.29 – 2.17 (m, 1H), 2.03 – 1.97 (m, 1H).

^{13}C NMR (126 MHz, DMSO) δ 172.8, 171.9, 163.3, 148.9, 148.7, 138.4, 137.2, 122.0, 49.3, 30.8, 29.9, 23.8.

HRMS (ESI+) (*m/z*): calculated for [(C12H12BrN3O3)+H$^+$]$^+$: 326.01348, found: 326.01409

Synthesis of 1-(4-(Bromomethyl)phenyl)dihydropyrimidine-2,4(1H,3H)-dione (18)

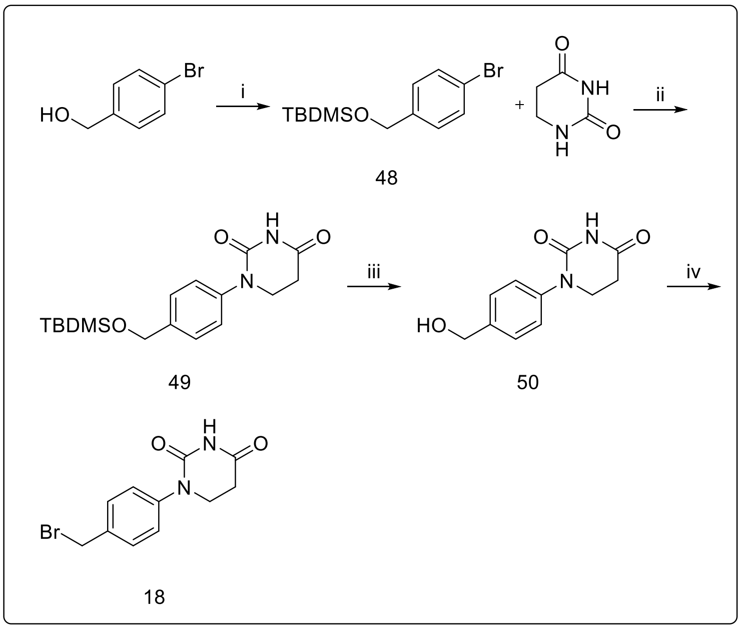

Figure S18: Synthetic route for 1-(4-(Bromomethyl)phenyl)dihydropyrimidine-2,4(1H,3H)-dione (18): (i) TBDMSCl, Imidazole, DMF; (ii) BrettPhos Pd G3, BrettPhos, Cs_2CO_3, 1,4-Dioxane, 100 °C; (iii) TBAF, DCM, 0 °C; (iv) HBr in water 90 °C

(4-Bromophenyl)methoxy-*tert*-butyl-dimethyl-silane (48)

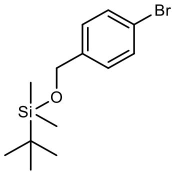

Synthesised according to the published procedure (ACS Catalysis (2024), 14(7), 4675-4682).

^{1}H NMR (500 MHz, $CDCl_3$) δ 7.45 (d, J = 8.5 Hz, 2H), 7.21 (d, J = 8.5 Hz, 2H), 4.69 (s, 2H), 0.95 (s, 9H), 0.10 (s, 6H).

^{13}C NMR (126 MHz, $CDCl_3$) δ 140.8, 131.6, 128.1, 120.9, 64.7, 26.3, 18.7, -4.9.

1-[4-[[*tert*-Butyl(dimethyl)silyl]oxymethyl]phenyl]hexahydropyrimidine-2,4-dione (49)

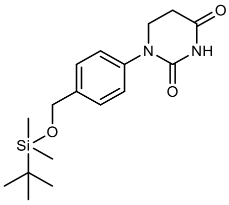

To a stirred solution of **48** (500 mg, 1.7 mmol) in 1,4-dioxane (16.6 mL) at ambient temperature was added dihydropyrimidine-2,4(1H,3H)-dione (852 mg, 7.5 mmol), Cs_2CO_3 (1.6 g, 5.0 mmol). The reaction vessel was then evacuated and backfilled with nitrogen three times. To the reaction was added BrettPhos Pd G3 (150 mg, 0.2 mmol) and BrettPhos (89 mg, 0.2 mmol) and the reaction vessel was sealed and left under inert atmosphere. The reaction was then stirred at 100 °C overnight before being filtered through cotton and filtrate was taken up in water (15 mL) and extracted with EtOAc, and the combined organic phase was separated. The aqueous component was extracted with EtOAc, and the combined organic extracts were dried over anhydrous magnesium sulfate and concentrated under reduced pressure. Purification by flash column chromatography, eluting with 0-100% EtOAc in heptane, afforded the title compound as a colourless oil (310 mg, 56%).

(ES+): 335 $[M+H^+]^+$.

^{1}H NMR (500 MHz, $CDCl_3$) δ 7.49 (s, 1H), 7.36 (d, *J* = 8.8 Hz, 2H), 7.26 – 7.24 (m, 2H), 4.73 (s, 2H), 3.86 (t, *J* = 6.7 Hz, 2H), 2.82 (t, *J* = 6.7 Hz, 2H), 0.94 (s, 9H), 0.10 (s, 6H).

^{13}C NMR (126 MHz, $CDCl_3$) δ 169.5, 151.8, 140.5, 139.8, 126.9, 125.0, 64.5, 45.4, 31.6, 26.1, 18.6, -5.1.

1-(4-(Hydroxymethyl)phenyl)dihydropyrimidine-2,4(1H,3H)-dione (50)

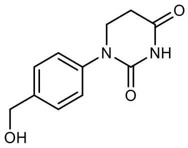

To a stirred solution of **49** (496 mg, 1.5 mmol) in dry THF (9.00 mL) at ambient temperature was added TBAF (2.5 mL, 2.5 mmol). The reaction was then stirred for 1 hour before concentrated under reduced pressure. Purification by flash column chromatography, eluting with 0-20% MeOH in DCM, afforded the title compound as a light yellow solid (302 mg, 92%).

^{1}H NMR (500 MHz, DMSO) δ 10.34 (s, 1H), 7.32 (d, *J* = 8.4 Hz, 2H), 7.27 (d, *J* = 8.4 Hz, 2H), 5.20 (t, *J* = 5.7 Hz, 1H), 4.49 (d, *J* = 5.7 Hz, 2H), 3.77 (t, *J* = 6.7 Hz, 2H), 2.70 (t, *J* = 6.7 Hz, 2H).

^{13}C NMR (126 MHz, DMSO) δ 170.6, 152.2, 140.6, 140.2, 126.7, 125.0, 62.5, 44.6, 31.1.

HRMS (ESI+) (*m/z*): calculated for $[(C11H12N2O3)+H^+]^+$: 221.09207 found: 221.09196

1-(4-(Bromomethyl)phenyl)dihydropyrimidine-2,4(1H,3H)-dione (18)

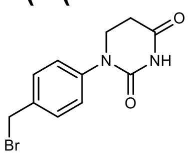

To a stirred solution of solution of 48% wt. hydrobromic acid in water (3.7 mL) was added **50** (115 mg, 0.5 mmol) at ambient temperature. The reaction was then stirred at 90 °C for 1 hour. The reaction was then taken up in water (10 mL) and extracted with EtOAc, and the combined

organic extracts were dried over anhydrous magnesium sulfate and concentrated under reduced pressure to afford title compound as a brown solid (83 mg, 56%).

^{1}H NMR (500 MHz, DMSO-H$_6$) δ 10.42 (s, 1H), 7.46 (d, *J* = 8.2 Hz, 2H), 7.32 (d, *J* = 8.2 Hz, 2H), 4.72 (s, 2H), 3.80 (t, *J* = 6.6 Hz, 2H), 2.70 (t, *J* = 6.6 Hz, 2H).

^{13}C NMR (126 MHz, DMSO) δ 171.0, 152.5, 144.3, 132.5, 130.1, 126.0, 125.7, 120.6, 56.9, 44.7, 31.5.

HRMS (ESI+) (*m/z*): calculated for [(C11H11BrN2O)+H$^+$]$^+$: 283.00767, found: 283.00806

Experimental Details of GSK3 Ligand Related Building Blocks

N-(3-Azidopropyl)-5-(3-chloro-4-methoxyphenyl)oxazole-4-carboxamide (19)

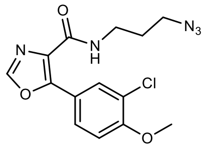

Synthesised according to the published procedure (European Journal of Medicinal Chemistry (2021) 226, 113889).

(ES+): 336 [M+H$^+$]$^+$.

^{1}H NMR (500 MHz, CDCl$_3$) δ 8.37 (dd, *J* = 8.7, 2.2 Hz, 1H), 8.32 (d, *J* = 2.2 Hz, 1H), 7.80 (s, 1H), 7.40 (s, 1H), 7.00 (d, *J* = 8.8 Hz, 1H), 3.96 (s, 3H), 3.54 (q, *J* = 6.6 Hz, 2H), 3.43 (t, *J* = 6.6 Hz, 2H), 1.92 (m, *J* = 6.7 Hz, 2H).

Synthesis of 2-(4-Azidophenyl)-N-(4-morpholinopyridin-3-yl)imidazo[1,2-b]pyridazine-8-carboxamide (20)

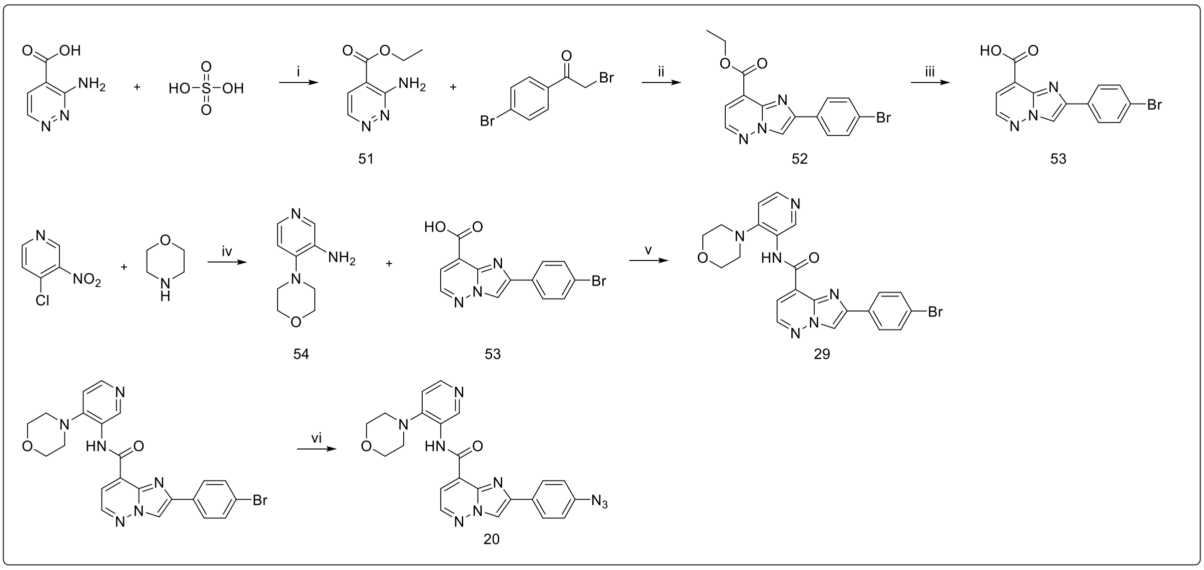

Figure S19: Synthetic route for 2-(4-Azidophenyl)-N-(4-morpholinopyridin-3-yl)imidazo[1,2-b]pyridazine-8-carboxamide (20): (i) H_2SO_4, EtOH, reflux; (ii) $NaHCO_3$, EtOH, 60 °C; (iii) LiOH, THF/MeOH/H_2O; (iv) Et_3N, AcO, Pd/C, H_2, MeOH; (v) T3P, Et_3N, DCM; (vi) $CuSO_4$, trans-N,N'-Dimethylcyclohexane-1,2-diamine, NaN_3, L(+)-Ascorbic acid sodium salt, EtOH, 80 °C, NaN_3, $NaNO_2$, AcOH, H_2O

Ethyl 3-aminopyridazine-4-carboxylate (51)

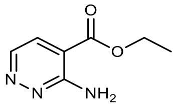

To a stirred solution of 3-aminopyridazine-4-carboxylic acid (4.0 g, 28.8 mmol) in EtOH (57.5 mL) at ambient temperature was added sulfuric acid (9.2 mL, 173.0 mmol). The reaction was then stirred at reflux overnight. The reaction was quenched at 0 °C by addition of saturated aqueous sodium hydrogen carbonate solution and the organic phase was separated. The aqueous component was extracted with DCM, and the combined organic extracts were dried over anhydrous magnesium sulfate and concentrated under reduced pressure to afford title compound, which was used directly in the next stage without further purification.

Ethyl 2-(4-bromophenyl)imidazo[1,2-b]pyridazine-8-carboxylate (52)

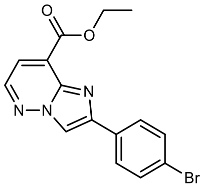

To a stirred suspension of 2-bromo-1-(4-bromophenyl)ethan-1-one (6.0 g, 21.6 mmol) in EtOH (43.3 mL) at ambient temperature was added **51** (3.6 g, 21.6 mmol). The reaction mixture was then stirred at 60 °C for 1.5 hours and allowed to cool to ambient temperature. Sodium hydrogen carbonate (s) (2.0 g, 23.8 mmol) was subsequently added, and the reaction was heated at 60 °C for 4 hours. The mixture was cooled to ambient temperature and filtered. The filtrate was resuspended in DCM and the organic phase was separated. The aqueous component was extracted with DCM, and the combined organic extracts were dried over anhydrous magnesium sulfate and concentrated under reduced pressure. Purification by flash column chromatography, eluting with 0-5% MeOH in DCM, afforded the title compound.

(ES+): 348 $[M+H^+]^+$.

2-(4-Bromophenyl)imidazo[1,2-b]pyridazine-8-carboxylic acid (53)

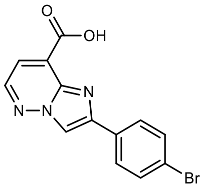

To a stirred solution of **52** (1.1 g, 3.3 mmol) in MeOH (9.3 mL) and THF (9.3 mL) at ambient temperature was added a solution of LiOH monohydrate (688 mg, 16.4 mmol) in water (1.9 mL). The reaction was then stirred for 1 hour. The reaction was quenched with HCl (4.1 mL, 16.4 mmol, 4M solution in 1,4-dioxane) before being concentrated under reduced pressure to afford the title compound which was used directly in the next stage without purification.

(ES+): 320 $[M+H^+]^+$.

4-Morpholinopyridin-3-amine (54)

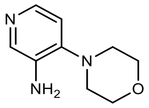

To a stirred solution of 4-chloro-3-nitropyridine (2.0 g, 12.6 mmol) in acetone (50.0 mL) at ambient temperature was added morpholine (1.7 mL, 18.9 mmol) and Et_3N (2.6 mL, 18.9 mmol). The reaction was then stirred overnight. The reaction was cooled to ambient temperature and filtered. The filtrate was washed in EtOAc and the combined filtrate was concentrated under reduced pressure before resuspended in MeOH (100 mL). The stirred suspension was placed under nitrogen atmosphere at ambient temperature and Pd/C (1.3 g,

1.3 mmol). The reaction was then evacuated and backfilled with nitrogen three times before placed under hydrogen atmosphere. The reaction was then stirred for 4 hours. The reaction mixture was filtered through celite, and the filtrate was concentrated under reduced pressure. Purification by flash column chromatography, eluting with 0-5% MeOH in DCM, afforded the title compound as an orange solid (1.68 g, 74%).

(ES+): 180 $[M+H^+]^+$.

^{1}H NMR (500 MHz, $CDCl_3$) δ 8.04 (s, 1H), 8.00 (d, *J* = 5.2 Hz, 1H), 6.79 (d, *J* = 5.2 Hz, 1H), 3.89 – 3.82 (m, 4H), 3.71 (s, 2H), 3.04 – 2.97 (m, 4H).

^{13}C NMR (126 MHz, $CDCl_3$) δ 145.4, 141.7, 137.8, 136.9, 113.5, 67.3, 49.9.

2-(4-Bromophenyl)-N-(4-morpholinopyridin-3-yl)imidazo[1,2-b]pyridazine-8-carboxamide (29)

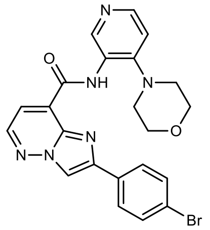

To a stirred solution of **53** (270 mg, 0.9 mmol) and **54** (167 mg, 0.9 mmol) in dry DCM (8.0 mL) at ambient temperature was added T3P (758 µL, 1.3 mmol) and then Et_3N (355 µL, 2.6 mmol). The reaction was then stirred overnight. The reaction was diluted with DCM and washed with water, and the organics were dried over anhydrous magnesium sulfate and concentrated under reduced pressure. Purification by flash column chromatography, eluting with 0-10% MeOH in DCM, afforded the title compound as a yellow solid (80 mg, 19%).

(ES+): 479 $[M+H^+]^+$.

1H NMR (500 MHz, CDCl3) δ 11.79 (s, 1H), 9.36 (s, 1H), 8.57 (d, J = 4.7 Hz, 1H), 8.43 – 8.40 (m, 2H), 8.02 (d, J = 4.7 Hz, 1H), 7.96 (d, J = 8.5 Hz, 2H), 7.62 (d, J = 8.5 Hz, 2H), 7.01 (d, J = 5.5 Hz, 1H), 3.73 – 3.67 (m, 4H), 3.14 – 3.08 (m, 4H).

13C NMR (126 MHz, CDCl3) δ 159.6, 150.7, 147.7, 146.0, 144.7, 143.9, 137.6, 132.3, 131.2, 128.1, 127.5, 127.4, 123.6, 118.8, 113.6, 113.4, 66.5, 50.8.

HRMS (ESI+) (*m/z*): calculated for $[(C22H19BrN6O2)+H^+]^+$: 479.08256, found: 479.08375

2-(4-Azidophenyl)-N-(4-morpholinopyridin-3-yl)imidazo[1,2-b]pyridazine-8-carboxamide (20)

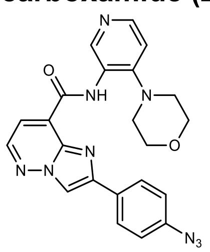

To a stirred solution of **29** (220 mg, 0.5 mmol) and *trans-N,N'*-dimethylcyclohexane-1,2-diamine (24 µL, 0.2 mmol) in ethanol (9.5 mL) at ambient temperature was added NaN_3 (90 mg, 1.4 mmol) and a solution of sodium L-ascorbate (60 mg, 0.3 mmol) in water (8.9 mL) and the reaction was placed under nitrogen atmosphere and $CuSO_4$ (22 mg, 0.1 mmol) was added. The reaction was then stirred under nitrogen atmosphere at 80 °C for 2 hours. After cooling to

ambient temperature, the reaction mixture was quenched by addition of saturated aqueous sodium hydrogen carbonate solution and the aqueous component was extracted with DCM, and the combined organic extracts were dried over anhydrous magnesium sulfate and concentrated under reduced pressure at ambient temperature. The residue was taken up in AcOH (13.5 mL) at 0 °C and $NaNO_2$ (32 mg, 0.5 mmol) in water (3.4 mL) was added. The reaction was then stirred at 0 °C for 20 minutes before addition of NaN_3 (30 mg, 0.5 mmol) in water (3.4 mL). The reaction was then stirred at ambient temperature for 1 hour. The reaction was diluted with water and the aqueous component was extracted with DCM, and the combined organic extracts were dried over anhydrous magnesium sulfate and concentrated under reduced pressure. Purification by flash column chromatography, eluting with 0-10% MeOH in DCM, afforded the title compound as a yellow solid (127 mg, 63%).

^{1}H NMR (500 MHz, $CDCl_3$) δ 11.82 (s, 1H), 9.37 (s, 1H), 8.56 (d, *J* = 4.7 Hz, 1H), 8.41 (d, *J* = 5.4 Hz, 1H), 8.38 (s, 1H), 8.07 (d, *J* = 8.6 Hz, 2H), 8.01 (d, *J* = 4.7 Hz, 1H), 7.14 (d, *J* = 8.6 Hz, 2H), 7.01 (d, *J* = 5.4 Hz, 1H), 3.73 – 3.67 (m, 4H), 3.14 – 3.08 (m, 4H).

^{13}C NMR (126 MHz, $CDCl_3$) δ 162.7, 159.7, 150.7, 147.6, 146.0, 145.0, 143.8, 141.1, 137.5, 129.1, 128.1, 127.3, 119.7, 118.6, 113.4, 113.3, 66.5, 50.7.

HRMS (ESI+) (*m/z*): calculated for [(C22H19N9O2)+H^{+}]$^{+}$: 442.17345, found: 442.17432

Experimental Details of Negative Control Related Building Blocks

Synthesis of 1-(6-(Bromomethyl)-1-methyl-1H-indazol-3-yl)-3-methyldihydropyrimidine-2,4(1H,3H)-dione (57)

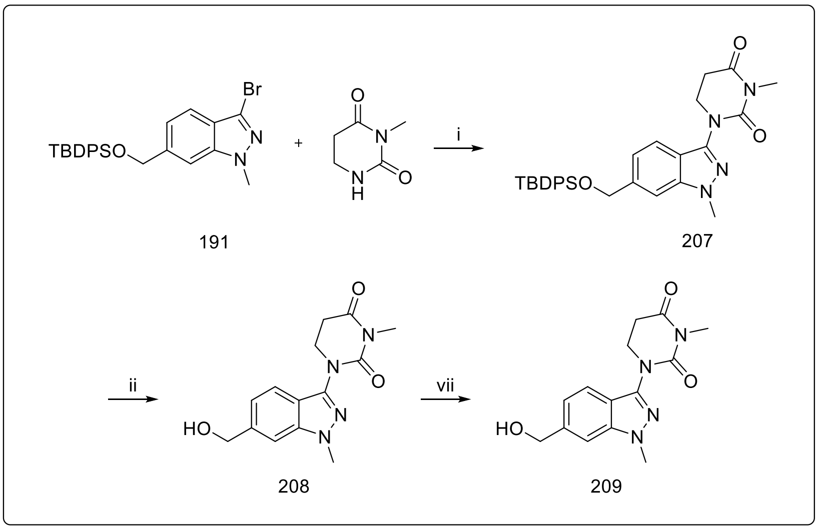

Figure S20: Synthetic route for 1-(6-(Bromomethyl)-1-methyl-1H-indazol-3-yl)-3-methyldihydropyrimidine-2,4(1H,3H)-dione (209): (i) Cs_2CO_3, CuI, trans-N,N'-Dimethylcyclohexane-1,2-diamine, 1,4-Dioxane, 80 °C; (ii) TBAF, THF; (iii) HBr in water, 90°C

1-(6-(((*tert*-Butyldiphenylsilyl)oxy)methyl)-1-methyl-1H-indazol-3-yl)-3-methyldihydropyrimidine-2,4(1H,3H)-dione (55)

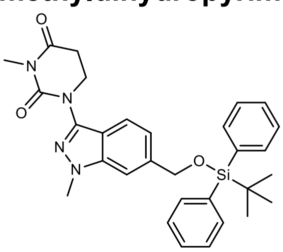

To a stirred solution of **46** (674 g, 1.4 mmol) in 1,4-dioxane (5.9 mL) at ambient temperature

was added 3-(4-methoxybenzyl)dihydropyrimidine-2,4(1H,3H)-dione (150 mg, 1.2 mmol), Cs_2CO_3 (763 mg, 2.3 mmol), CuI (45 mg, 0.2 mmol) and *trans-N,N'*-dimethylcyclohexane-1,2-diamine (37 µL, 0.2 mmol). The reaction vessel was then evacuated and backfilled with nitrogen three times. The reaction was then stirred at 80 °C under inert atmosphere overnight. The reaction was filtered through cotton wool and concentrated under reduced pressure. Purification by flash column chromatography, eluting with 0-100% EtOAc in heptane, afforded the title compound as a colourless oil (335 mg, 54%).

^{1}H NMR (500 MHz, $CDCl_3$) δ 7.62 – 7.57 (m, 4H), 7.50 (d, *J* = 8.4 Hz, 1H), 7.35 – 7.30 (m, 2H), 7.29 – 7.24 (m, 5H), 6.95 (d, *J* = 8.4 Hz, 1H), 4.80 (s, 2H), 3.92 (t, *J* = 6.7 Hz, 2H), 3.87 (s, 3H), 3.18 (s, 3H), 2.83 (t, *J* = 6.7 Hz, 2H), 1.01 (s, 9H).

^{13}C NMR (126 MHz, $CDCl_3$) δ 170.0, 153.2, 142.4, 141.9, 141.1, 136.0, 133.8, 130.2, 128.2, 121.7, 119.8, 117.0, 106.4, 66.0, 43.8, 35.8, 32.4, 28.3, 27.3, 19.8.

HRMS (ESI+) (*m/z*): calculated for [(C30H34N4O3Si)+H$^+$]$^+$: 527.24730, found: 527.24857

1-(6-(Hydroxymethyl)-1-methyl-1H-indazol-3-yl)-3-methyldihydropyrimidine-2,4(1H,3H)-dione (56)

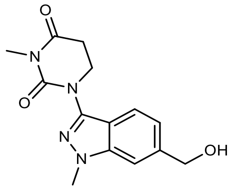

To a stirred solution of **55** (335 mg, 0.6 mmol) in dry THF (4.2 mL) at ambient temperature was added TBAF (763 µL, 0.8 mmol). The reaction was then stirred for 1 hour. The reaction was quenched by addition of HCl (320 µL, 1.3 mmol, 4M solution in 1,4-dioxane) and stirred for 5 minutes before being concentrated under reduced pressure. Purification by flash column chromatography, eluting with 0-15% MeOH in DCM, afforded the title compound as a white solid (155 mg, 85%).

^{1}H NMR (500 MHz, DMSO) δ 7.59 (d, *J* = 8.4 Hz, 1H), 7.50 (s, 1H), 7.06 (d, *J* = 8.4 Hz, 1H), 5.38 (t, *J* = 5.6 Hz, 1H), 4.64 (d, *J* = 5.6 Hz, 2H), 3.97 (s, 3H), 3.90 (t, *J* = 6.6 Hz, 2H), 3.09 (s, 3H), 2.88 (t, *J* = 6.6 Hz, 2H).

^{13}C NMR (126 MHz, DMSO) δ 170.2, 152.9, 142.3, 142.2, 141.4, 121.6, 119.8, 116.6, 107.0, 63.4, 43.5, 35.7, 31.8, 27.8.

HRMS (ESI+) (*m/z*): calculated for [(C14H16N4O3)+H$^+$]$^+$: 289.12952, found: 289.12991

1-(6-(Bromomethyl)-1-methyl-1H-indazol-3-yl)-3-methyldihydropyrimidine-2,4(1H,3H)-dione (57)

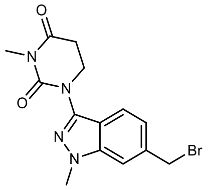

To a stirred solution of solution of 48% wt. hydrobromic acid in water (3.6 mL) was added **56** (155 mg, 0.5 mmol) at ambient temperature. The reaction was then stirred at 90 °C for 30 minutes. The reaction was then taken up in water (10 mL) and extracted with EtOAc, and the combined organic extracts were dried over anhydrous magnesium sulfate and concentrated under reduced pressure. Purification by reverse-phase flash column chromatography, eluting with 0-100% aq 0.1% formic acid in MeCN, afforded the title compound as a white solid (34 mg, 18%).

(ES+): 353 [M+H$^+$]$^+$.

^{1}H NMR (500 MHz, $CDCl_3$) δ 7.67 (d, *J* = 8.4 Hz, 1H), 7.37 (s, 1H), 7.19 (dd, *J* = 8.4, 1.2 Hz, 1H), 4.63 (s, 2H), 4.04 – 3.99 (m, 5H), 3.29 (s, 3H), 2.93 (t, *J* = 6.7 Hz, 2H).

^{13}C NMR (126 MHz, $CDCl_3$) δ 169.9, 153.1, 142.6, 141.4, 137.3, 122.8, 122.5, 117.8, 109.8, 43.7, 36.0, 34.2, 32.4, 28.3.

General Protocol for Plate-based Library Synthesis

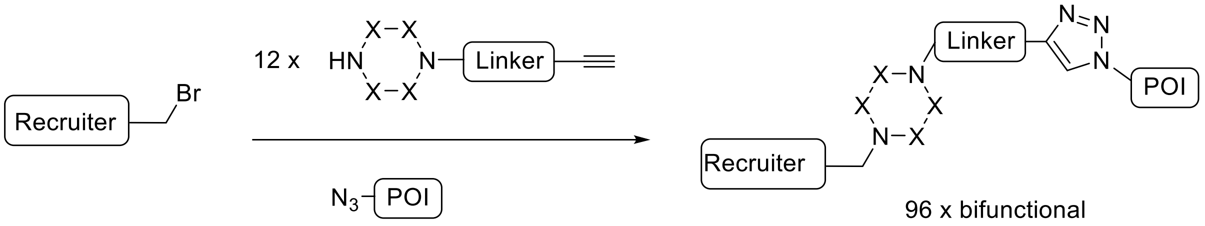

Reagents were prepared as the following DMF stock solutions unless stated otherwise in 1.9 mL Tri-coded FluidX tubes, 48-format: E3-ligands (0.08 M), linkers (0.095 M with 3.2 eq of Et_3N), GSK3 Ligand-Azide (0.1 M), $CuSO_4{*}5H_2O$ with THPTA in water (0.05 M $CuSO_4$, 0.1 M THPTA), sodium L-ascorbate in water (0.4 M) and Benzyl-bromide (negative control) (0.08 M). All liquid transfers were done using Opentrons OT-2 liquid handling robot equipped with 1-channel P300 and 8-channel P20 pipettes. All plate-based synthesis were carried out in a Para-dox standard 96-well plate, Parallel synthesis aluminium reaction block, with 1 mL, 8x30mm glass inserts. All final crude products were made to 10 mM DMSO stock solutions, assuming 100% conversion and purity. Low resolution electrospray (ES) mass spectra were recorded on an Advion Compact Mass Spectrometer (CMS; model ExpressIon CMS) connected to Dionex µLtimate 3000 UPLC system with diode array detector. HPLC chromatographic separations were conducted using a Waters XBridge C18 column, 2.1 x 50mm, 3.5 µm particle size or Waters XSelect 2.1 x 30mm, 2.5 µm particle size. The compounds were eluted with a gradient of 5 to 95% acetonitrile/water +0.1% Ammonia or +0.1% formic acid.

To a 96-well plate was added 18.8 µL of E3 ligase ligand containing stock solution with compound **13** in each well of row A, **14** in row B, **15** in row C, … **18** in row H at ambient temperature. Then 10.5 µL of stock solution containing linker **1** was added to each well of column 1, linker **2** to each well of column 2, linker **3** to each well of column 3, … linker **12** to each well of column 12 to achieve final reaction concentration of 0.03 M. The reaction plate was sealed and left for 24 hours. The reaction plate was unsealed, and each reaction well was further diluted with 19.5 µL of DMF before addition of 10 µL of GSK3 Ligand-Azide (**19** or **20**) to each reaction well followed by addition of 10 µL $CuSO_4$/THPTA stock solution and 7.5 µL sodium L-ascorbate stock solution to each reaction well to achieve a final reaction concentration of 0.015 M. The reaction plate was then sealed and left for 24 hours before addition of 1 µL Formic acid to each reaction well. Reaction mixtures were loaded on to ISOLUTE-96 SCX-plate washing with MeCN (2 mL) and then eluting desired products with 3.5 N NH_3 in MeCN (1 mL) into 2 mL ISOLUTE collection plate before concentration *in vacuo*. Following concentration, 100 µL of DMSO was added to each reaction well to afford a 10 mM DMSO crude stock solution which was taken forward for analytics and screening.

Experimental Details of Selected Hits

2-(4-(4-(5-(4-(4-((2,6-dioxopiperidin-3-yl)carbamoyl)-3-fluorobenzyl)piperazin-1-yl)pentyl)-1H-1,2,3-triazol-1-yl)phenyl)-N-(4-morpholinopyridin-3-yl)imidazo[1,2-b]pyridazine-8-carboxamide (21)

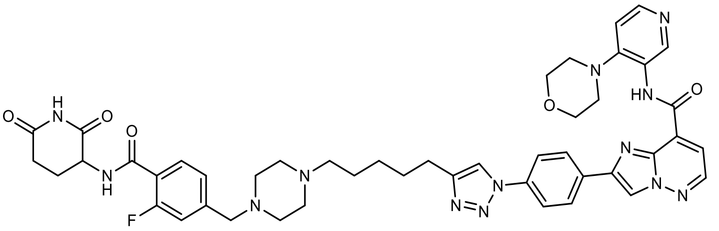

To a stirred solution of **12** (660 µL, 0.1 M, 72 µmol) in DMF at ambient temperature was added Et_3N (20 µL, 140 µmol). The reaction was then stirred for 10 minutes. To the solution was added **15** (950 µL, 0.08 M, 79 µmol) in DMF. The reaction was stirred overnight before being concentrated *in vacuo*. The residue was taken up in MeCN/Water (0.8 mL; 1:1), and purification by preparative HPLC, eluting with 0-100% aq 0.1% formic acid in MeCN, and the collected residue was taken up in DMF/Water (3.5 mL; 1:5) and stirred at ambient temperature. To the solution was added a mixture of mixture of $CuSO_4{*}5H_2O$ (660 µL, 0.05 M, 33 µmol) and THPTA (660 µL, 0.1 M, 66 µmol) in water followed by addition of sodium L-ascorbate (330 µL, 0.2M, 132 µmol) in water. A solution of **20** (722 µL, 0.1 M, 72 µmol) in DMF was added and the reaction was then stirred overnight before being concentrated *in vacuo*. The residue was taken up in DMSO/Water (0.5 mL; 1:5), and purification by preparative HPLC, eluting with 0-100% aq 0.1% formic acid in MeCN, afforded the title compound as a yellow solid (6.0 mg, 10%).

(ES+): 884 $[M+H^{+}]^{+}$.

^{1}H NMR (500 MHz, DMSO) δ 11.76 (s, 1H), 10.87 (s, 1H), 9.27 (s, 1H), 9.18 (s, 1H), 8.83 (d, *J* = 4.7 Hz, 1H), 8.68 (s, 1H), 8.50 (dd, *J* = 8.1, 3.1 Hz, 1H), 8.43 (d, *J* = 8.5 Hz, 2H), 8.35 (d, *J* = 5.3 Hz, 1H), 8.08 (d, *J* = 8.5 Hz, 2H), 7.96 (d, *J* = 4.7 Hz, 1H), 7.63 (t, *J* = 7.7 Hz, 1H), 7.23 – 7.19 (m, 3H), 4.78 – 4.73 (m, 1H), 3.66 – 3.60 (m, 4H), 3.49 (s, 2H), 3.12 – 3.06 (m, 4H), 2.83 – 2.70 (m, 4H), 2.55 (t, *J* = 3.8 Hz, 1H), 2.37 (s, 4H), 2.27 (t, *J* = 7.2 Hz, 2H), 2.15 – 1.97 (m, 4H), 1.73 – 1.68 (m, 2H), 1.50 – 1.44 (m, 2H), 1.41 – 1.33 (m, 2H).

^{13}C NMR (101 MHz, DMSO) δ 173.37, 172.30, 159.69, 158.55, 150.77, 148.76, 147.54, 145.25, 143.64, 137.53, 137.42, 132.34, 130.62, 127.99, 125.01, 120.64, 120.54, 119.21, 116.51, 116.28, 115.35, 114.35f, 110.27, 66.15, 61.43, 58.17, 53.25, 53.14, 50.58, 50.15, 31.37, 29.16, 26.91, 26.53, 25.48, 24.50. N.B. 4x carbons not visible in ^{13}C spectra.

HRMS (ESI+) (*m/z*): calculated for $[(C46H50FN13O5)+H^{+}]^{2+}$: 442.70938, found: 442.71076

2-(4-(4-(5-((4-((6-((2,6-dioxopiperidin-3-yl)carbamoyl)pyridin-3-yl)methyl)piperazin-1-yl)methyl)pyridin-2-yl)-1H-1,2,3-triazol-1-yl)phenyl)-N-(4-morpholinopyridin-3-yl)imidazo[1,2-b]pyridazine-8-carboxamide (22)

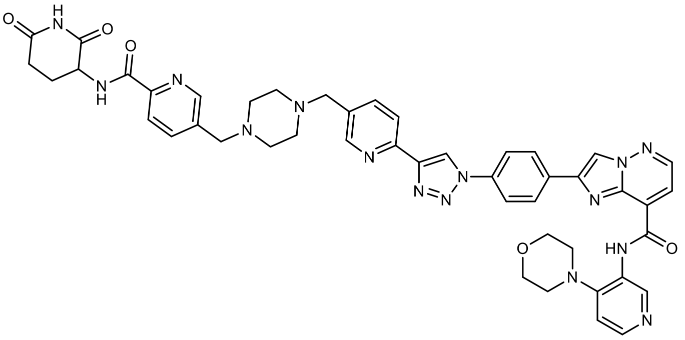

To a stirred solution of **2** (660 µL, 0.1 M, 66 µmol) in DMF at ambient temperature was added Et_3N (20 µL, 144 µmol). The reaction was then stirred for 10 minutes. To the solution was added **17** (980 µL, 0.08 M, 79 µmol) in DMF. The reaction was stirred overnight before being concentrated *in vacuo*. The residue was taken up in MeCN/Water (0.8 mL; 1:1), and purification by preparative HPLC, eluting with 0-100% aq 0.1% formic acid in MeCN, and the

collected residue was taken up in DMF/Water (3.5 mL; 1:5) and stirred at ambient temperature. To the solution was added a mixture of mixture of $CuSO_4*5H_2O$ (660 µL, 0.05 M, 33 µmol) and THPTA (660 µL, 0.1 M, 66 µmol) in water followed by addition of sodium L-ascorbate (330 µL, 0.2 M, 132 µmol) in water. A solution of **20** (361 µL, 0.1 M, 36 µmol) in DMF was added and the reaction was then stirred overnight before being concentrated *in vacuo*. The residue was taken up in DMSO/Water (0.5 mL; 1:5), and purification by preparative HPLC, eluting with 0-100% aq 0.1% formic acid in MeCN, afforded the title compound as a yellow solid (5.0 mg, 9%).
(ES+): 889 $[M+H^+]^+$.

^1H NMR (500 MHz, DMSO) δ 11.75 (s, 1H), 10.87 (s, 1H), 9.43 (s, 1H), 9.27 (s, 1H), 9.17 (s, 1H), 9.05 (d, *J* = 8.2 Hz, 1H), 8.82 (d, *J* = 4.5 Hz, 1H), 8.57 (d, *J* = 7.3 Hz, 2H), 8.45 (d, *J* = 8.4 Hz, 2H), 8.35 (d, *J* = 5.4 Hz, 1H), 8.23 (d, *J* = 8.3 Hz, 2H), 8.10 (d, *J* = 7.9 Hz, 1H), 8.03 (d, *J* = 7.9 Hz, 1H), 7.95 (d, *J* = 4.5 Hz, 1H), 7.92 (d, *J* = 8.0 Hz, 1H), 7.86 (d, *J* = 8.0 Hz, 1H), 7.21 (d, *J* = 5.4 Hz, 1H), 4.82 – 4.75 (m, 1H), 3.67 – 3.59 (m, 6H), 3.56 (s, 2H), 3.13 – 3.05 (m, 4H), 2.83 – 2.76 (m, 1H), 2.54 (s, 4H), 2.46 – 2.41 (m, 4H), 2.29 – 2.14 (m, 2H), 2.05 – 1.95 (m, 2H).

^{13}C NMR (126 MHz, DMSO) δ 173.2, 172.4, 164.0, 159.4, 150.4, 150.2, 148.9, 148.6, 148.4, 147.2, 144.9, 143.2, 137.9, 137.3, 137.2, 136.8, 133.5, 132.4, 127.7, 126.9, 122.0, 121.2, 120.6, 119.6, 115.2, 114.1, 113.5, 65.9, 59.1, 58.9, 52.7, 50.3, 49.6, 40.6, 31.2, 28.6, 24.2. N.B. 2x carbons not visible in ^{13}C spectra.

HRMS (ESI+) (*m/z*): calculated for $[(C46H45N15O5)+H^+]^{2+}$: 444.69369, found: 444.69477

2-(4-(4-(3-(4-((6-((2,6-dioxopiperidin-3-yl)carbamoyl)pyridin-3-yl)methyl)piperazin-1-yl)propyl)-1H-1,2,3-triazol-1-yl)phenyl)-N-(4-morpholinopyridin-3-yl)imidazo[1,2-b]pyridazine-8-carboxamide (23)

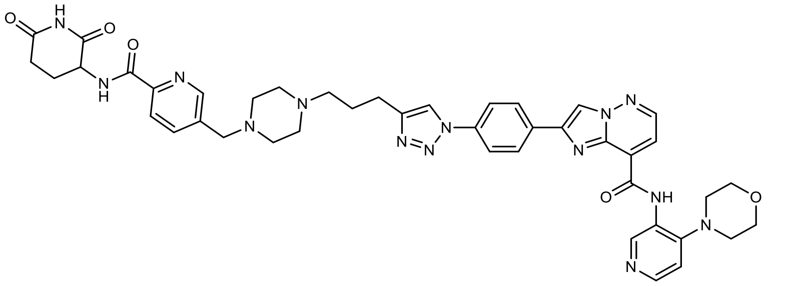

To a stirred solution of **12** (660 µL, 0.1 M, 66 µmol) in DMF at ambient temperature was added Et_3N (29 µL, 21 µmol). The reaction was then stirred for 10 minutes. To the solution was added **17** (980 µL, 0.08 M, 79 µmol) in DMF. The reaction was stirred overnight before being concentrated *in vacuo*. The residue was taken up in MeCN/Water (0.8 mL; 1:1), and purification by preparative HPLC, eluting with 0-100% aq 0.1% formic acid in MeCN, and the collected residue was taken up in DMF/Water (3.5 mL; 1:5) and stirred at ambient temperature. To the solution was added a mixture of mixture of $CuSO_4*5H_2O$ (660 µL, 0.05 M, 33 µmol) and THPTA (660 µL, 0.1 M, 66 µmol) in water followed by addition of sodium L-ascorbate (330 µL, 0.2 M, 132 µmol) in water. A solution of **20** (660 µL, 0.1 M, 66 µmol) in DMF was added and the reaction was then stirred overnight before being concentrated *in vacuo*. The residue was taken up in DMSO/Water (0.5 mL; 1:5), and purification by preparative HPLC, eluting with 0-100% aq 0.1% formic acid in MeCN, afforded the title compound as a yellow solid (4 mg, 7%).
(ES+): 839 $[M+H^+]^+$.

^1H NMR (500 MHz, DMSO) δ 11.74 (s, 1H), 10.87 (s, 1H), 9.25 (s, 1H), 9.17 (s, 1H), 9.05 (d, *J* = 8.4 Hz, 1H), 8.81 (d, *J* = 4.8 Hz, 1H), 8.67 (s, 1H), 8.57 (s, 1H), 8.41 (d, *J* = 8.2 Hz, 2H), 8.34 (s, 1H), 8.11 – 8.01 (m, 3H), 7.96 – 7.90 (m, 2H), 7.20 (d, *J* = 5.5 Hz, 1H), 4.82 – 4.76 (m, 1H), 3.65 – 3.57 (m, 6H), 3.07 (s, 4H), 2.83 – 2.78 (m, 1H), 2.73 (t, *J* = 7.7 Hz, 2H),

2.54 (s, 4H), 2.43 – 2.34 (m, 6H), 2.27 – 2.17 (m, 2H), 2.02 – 1.94 (m, 2H), 1.88 – 1.80 (m, 2H).

^{13}C NMR (126 MHz, DMSO) δ 173.5, 172.6, 164.3, 159.7, 150.7, 149.2, 148.9, 148.7, 147.5, 145.2, 143.6, 138.5, 137.7, 137.5, 137.4, 132.3, 128.0, 127.2, 122.3, 120.6, 115.4, 114.3, 66.1, 59.3, 57.6, 53.2, 53.1, 50.6, 49.9, 40.9, 31.5, 26.6, 24.5, 23.4. N.B. 3x carbons not visible in ^{13}C spectra.

HRMS (ESI+) (*m/z*): calculated for [(C43H46N14O5)+H$^+$]$^{2+}$: 420.19606, found: 420.19686

2-(4-(4-(5-((4-((3-(2,4-Dioxotetrahydropyrimidin-1(2H)-yl)-1-methyl-1H-indazol-6-yl)methyl)piperazin-1-yl)methyl)pyridin-2-yl)-1H-1,2,3-triazol-1-yl)phenyl)-*N*-(4-morpholinopyridin-3-yl)imidazo[1,2-b]pyridazine-8-carboxamide (KH1) (24)

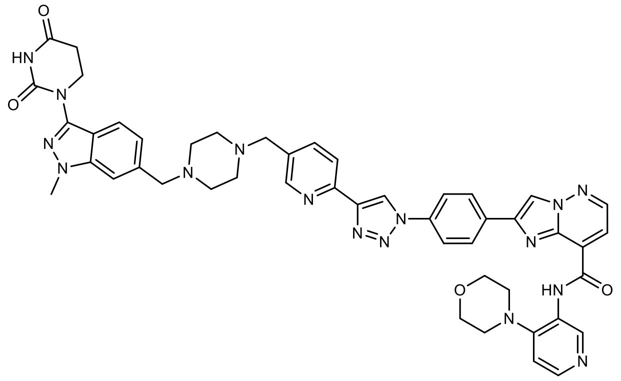

To a stirred solution of **2** (1.1 mL, 0.1 M, 114 µmol) in DMF at ambient temperature was added Et_3N (18 µL, 126 µmol). The reaction was then stirred for 10 minutes. To the solution was added **16** (1.7 mL, 0.08 M, 137 µmol) in DMF. The reaction was stirred overnight before being concentrated *in vacuo*. The residue was taken up in MeCN/Water (1 mL; 1:1), and purification by preparative HPLC, eluting with 0-100% aq 0.1% formic acid in MeCN, and the collected residue was taken up in DMF/Water (5 mL; 1:5) and stirred at ambient temperature. To the solution was added a mixture of mixture of $CuSO_4{*}5H_2O$ (1.1 mL, 0.05 M, 57 µmol) and THPTA (1.1 mL, 0.1 M, 114 µmol) in water followed by addition of sodium L-ascorbate (570 µL, 0.2 M, 229 µmol) in water. A solution of **20** (1.2 mL, 0.1 M, 126 µmol) in DMF was added and the reaction was then stirred overnight before being concentrated *in vacuo*. The residue was taken up in DMSO/Water (1 mL; 1:5), and purification by preparative HPLC, eluting with 0-100% aq 0.1% formic acid in MeCN, afforded the title compound as a yellow solid (25 mg, 24%).

(ES+): 900 [M+H$^+$]$^+$.

^{1}H NMR (500 MHz, DMSO) δ 11.77 (s, 1H), 10.55 (s, 1H), 9.43 (s, 1H), 9.29 (s, 1H), 9.18 (s, 1H), 8.83 (d, *J* = 4.7 Hz, 1H), 8.56 (d, *J* = 2.1 Hz, 1H), 8.46 (d, *J* = 8.4 Hz, 2H), 8.35 (d, *J* = 5.4 Hz, 1H), 8.24 (d, *J* = 8.4 Hz, 2H), 8.11 (d, *J* = 8.0 Hz, 1H), 7.96 (d, *J* = 4.7 Hz, 1H), 7.87 (dd, *J* = 8.0, 2.2 Hz, 1H), 7.58 (d, *J* = 8.4 Hz, 1H), 7.47 (s, 1H), 7.22 (d, *J* = 5.5 Hz, 1H), 7.10 (d, *J* = 8.4 Hz, 1H), 3.97 (s, 3H), 3.91 (t, *J* = 6.7 Hz, 2H), 3.64 (t, *J* = 4.7 Hz, 4H), 3.60 (s, 2H), 3.57 (s, 2H), 3.10 (t, *J* = 4.7 Hz, 4H), 2.75 (t, *J* = 6.7 Hz, 2H), 2.54 (s, 4H), 2.45 (s, 4H).

^{13}C NMR (126 MHz, DMSO) δ 170.7, 159.2, 152.0, 150.3, 150.0, 148.3, 147.1, 144.8, 143.0, 141.6, 140.9, 137.8, 137.3, 137.1, 136.6, 133.4, 132.3, 127.5, 126.8, 121.6, 121.3, 121.0, 120.5, 119.5, 118.8, 116.5, 115.0, 113.9, 109.3, 65.7, 62.3, 59.0, 52.7, 52.6, 50.1, 44.1, 40.4, 35.3, 31.0. N.B. 2x carbons not visible in ^{13}C spectra.

HRMS (ESI+) (*m/z*): calculated for [(C47H46N16O4)+H$^+$]$^{2+}$: 450.20168, found: 450.20276

2-(4-(4-(5-(4-((3-(2,4-dioxotetrahydropyrimidin-1(2H)-yl)-1-methyl-1H-indazol-6-yl)methyl)piperazin-1-yl)pentyl)-1H-1,2,3-triazol-1-yl)phenyl)-N-(4-morpholinopyridin-3-yl)imidazo[1,2-b]pyridazine-8-carboxamide (25)

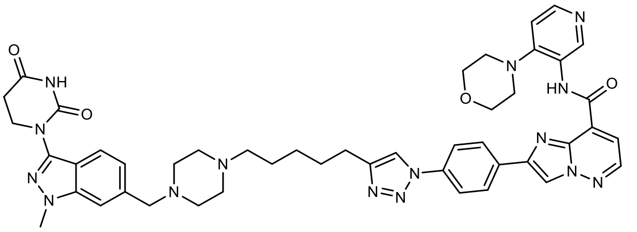

To a stirred solution of **7** (490 µL, 0.1 M, 39 µmol) in DMF at ambient temperature was added Et_3N (10 µL, 72 µmol). The reaction was then stirred for 10 minutes. To the solution was added **16** (330 µL, 0.08 M, 33 µmol) in DMF. The reaction was stirred overnight before being concentrated *in vacuo*. The residue was taken up in MeCN/Water (0.5 mL; 1:1), and purification by preparative HPLC, eluting with 0-100% aq 0.1% formic acid in MeCN, and the collected residue was taken up in DMF/Water (2.5 mL; 1:5) and stirred at ambient temperature. To the solution was added a mixture of mixture of $CuSO_4{*}5H_2O$ (330 µL, 0.05 M, 16 µmol) and THPTA (330 µL, 0.1 M, 32 µmol) in water followed by addition of sodium L-ascorbate (160 µL, 0.2 M, 66 µmol) in water. A solution of **20** (360 µL, 0.1 M, 36 µmol) in DMF was added and the reaction was then stirred overnight before being concentrated *in vacuo*. The residue was taken up in DMSO/Water (0.5 mL; 1:5), and purification by preparative HPLC, eluting with 0-100% aq 0.1% formic acid in MeCN, afforded the title compound as a yellow solid (5.0 mg, 18%).

(ES+): 864 $[M+H^+]^+$.

^1H NMR (500 MHz, DMSO) δ 11.73 (s, 1H), 10.55 (s, 1H), 9.23 (s, 1H), 9.17 (s, 1H), 8.80 (d, *J* = 4.7 Hz, 1H), 8.66 (s, 1H), 8.40 (d, *J* = 8.2 Hz, 2H), 8.33 (d, *J* = 5.5 Hz, 1H), 8.06 (d, *J* = 8.2 Hz, 2H), 7.94 (d, *J* = 4.6 Hz, 1H), 7.56 (d, *J* = 8.4 Hz, 1H), 7.44 (s, 1H), 7.19 (d, *J* = 5.5 Hz, 1H), 7.07 (d, *J* = 8.5 Hz, 1H), 3.95 (s, 3H), 3.91 (t, *J* = 6.6 Hz, 2H), 3.61 (t, *J* = 4.5 Hz, 4H), 3.55 (s, 2H), 3.09 – 3.05 (m, 4H), 2.76 – 2.70 (m, 4H), 2.54 (s, 4H), 2.37 (s, 4H), 2.25 (t, *J* = 7.3 Hz, 2H), 1.69 (p, *J* = 7.5 Hz, 2H), 1.45 (q, *J* = 7.4 Hz, 2H), 1.36 (q, *J* = 7.7 Hz, 3H).

^{13}C NMR (126 MHz, DMSO) δ 171.11, 159.67, 152.34, 150.73, 149.15, 147.52, 145.26, 143.99, 143.58, 142.06, 141.32, 140.00, 137.98, 137.52, 137.37, 132.32, 127.97, 127.20, 122.51, 120.59, 119.15, 116.98, 115.36, 114.35, 110.05, 109.06, 66.14, 63.21, 50.55, 44.58, 35.77, 31.49, 26.68 25.39. N.B 4x quaternary carbons not visible in ^{13}C spectra, all 25 expected non-quaternary carbons identified in HSQC (see Supplementary spectrum 103).

HRMS (ESI+) (*m/z*): calculated for $[(C46H51N15O4)+H^+]^{2+}$: 439.71971, found: 439.72101

2-(4-(4-(3-(4-((3-(2,4-dioxotetrahydropyrimidin-1(2H)-yl)-1-methyl-1H-indazol-6-yl)methyl)piperazin-1-yl)propyl)-1H-1,2,3-triazol-1-yl)phenyl)-N-(4-morpholinopyridin-3-yl)imidazo[1,2-b]pyridazine-8-carboxamide (KH2) (26)

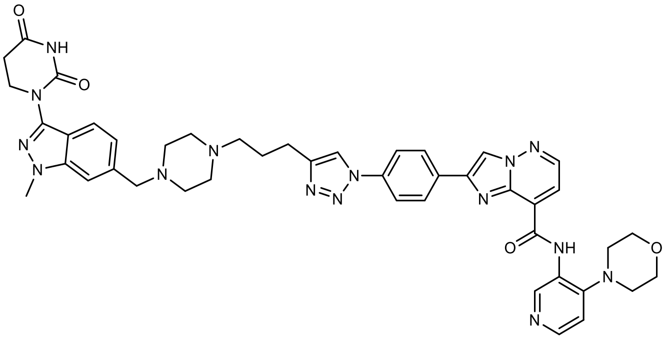

To a stirred solution of **12** (660 µL, 0.1 M, 66 µmol) in DMF at ambient temperature was added Et_3N (20 µL, 140 µmol). The reaction was then stirred for 10 minutes. To the solution was added **16** (980 µL, 0.08 M, 79 µmol) in DMF. The reaction was stirred overnight before being concentrated *in vacuo*. The residue was taken up in MeCN/Water (0.8 mL; 1:1), and purification by preparative HPLC, eluting with 0-100% aq 0.1% formic acid in MeCN, and the collected residue was taken up in DMF/Water (3.5 mL; 1:5) and stirred at ambient temperature. To the solution was added a mixture of mixture of $CuSO_4$*$5H_2O$ (660 µL, 0.05 M, 33 µmol) and THPTA (660 µL, 0.1 M, 66 µmol) in water followed by addition of sodium L-ascorbate (330 µL, 0.2 M, 132 µmol) in water. A solution of **20** (660 µL, 0.1 M, 66 µmol) in DMF was added and the reaction was then stirred overnight before being concentrated *in vacuo*. The residue was taken up in DMSO/Water (0.5 mL; 1:5), and purification by preparative HPLC, eluting with 0-100% aq 0.1% formic acid in MeCN, afforded the title compound as a yellow solid (11.0 mg, 20%).

(ES+): 850 $[M+H^+]^+$.

^{1}H NMR (500 MHz, DMSO) δ 11.75 (s, 1H), 10.56 (s, 1H), 9.26 (s, 1H), 9.18 (s, 1H), 8.82 (d, *J* = 4.7 Hz, 1H), 8.68 (s, 1H), 8.42 (d, *J* = 8.3 Hz, 2H), 8.35 (d, *J* = 5.4 Hz, 1H), 8.07 (d, *J* = 8.3 Hz, 2H), 7.96 (d, *J* = 4.7 Hz, 1H), 7.59 (d, *J* = 8.3 Hz, 1H), 7.48 (s, 1H), 7.21 (d, *J* = 5.4 Hz, 1H), 7.10 (d, *J* = 8.3 Hz, 1H), 3.98 (s, 3H), 3.92 (t, *J* = 6.7 Hz, 2H), 3.65 – 3.61 (m, 4H), 3.60 (s, 2H), 3.11 – 3.07 (m, 4H), 2.78 – 2.73 (m, 4H), 2.46 – 2.37 (m, 7H), 1.88 – 1.82 (m, 2H).

^{13}C NMR (126 MHz, DMSO) δ 171.1, 163.6, 159.7, 152.3, 150.7, 148.5, 147.5, 145.3, 145.2, 143.6, 142.1, 141.4, 137.5, 137.4, 132.3, 128.0, 127.2, 122.1, 121.8, 120.7, 120.6, 119.2, 117.0, 115.4, 114.4, 109.9, 66.2, 62.7, 57.4, 53.1, 52.9, 50.6, 44.6, 35.8, 31.5, 26.3, 23.3.

HRMS (ESI+) (*m/z*): calculated for $[(C44H47N15O4)+H^+]^{2+}$: 425.70406, found: 425.70484

2-(4-(4-(5-((4-((1-methyl-3-(3-methyl-2,4-dioxotetrahydropyrimidin-1(2H)-yl)-1H-indazol-6-yl)methyl)piperazin-1-yl)methyl)pyridin-2-yl)-1H-1,2,3-triazol-1-yl)phenyl)-N-(4-morpholinopyridin-3-yl)imidazo[1,2-b]pyridazine-8-carboxamide (27)

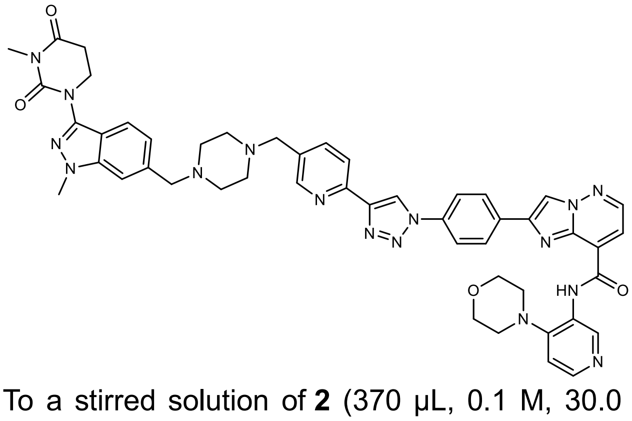

To a stirred solution of **2** (370 µL, 0.1 M, 30.0 µmol) in DMF at ambient temperature was added Et_3N (3.8 µL, 27.0 µmol). The reaction was then stirred for 10 minutes. To the solution was added **57** (250 µL, 0.08 M, 25.0 µmol) in DMF. The reaction was stirred overnight before being concentrated *in vacuo*. The residue was taken up in MeCN/Water (0.5 mL; 1:1), and purification by preparative HPLC, eluting with 0-100% aq 0.1% formic acid in MeCN, and the collected residue was taken up in DMF/Water (2.5 mL; 1:5) and stirred at ambient temperature. To the solution was added a mixture of mixture of $CuSO_4$*$5H_2O$ (250 µL, 0.05M, 12.0 µmol) and THPTA (250 µL, 0.1 M, 25.0 µmol) in water followed by addition of sodium L-ascorbate (120 µL, 0.2 M, 50.0 µmol) in water. A solution of **20** (270 µL, 0.1 M, M, 27 µmol) in DMF was added and the reaction was then stirred overnight before being concentrated *in vacuo*. The

residue was taken up in DMSO/Water (0.5 mL; 1:5), and purification by preparative HPLC, eluting with 0-100% aq 0.1% formic acid in MeCN, afforded the title compound as a yellow solid (4.2 mg, 18%).

(ES+): 914 $[M+H^+]^+$.

^{1}H NMR (500 MHz, DMSO) δ 11.73 (s, 1H), 9.41 (s, 1H), 9.24 (s, 1H), 9.17 (s, 1H), 8.80 (d, *J* = 4.7 Hz, 1H), 8.55 (d, *J* = 2.1 Hz, 1H), 8.42 (d, *J* = 8.3 Hz, 2H), 8.34 (d, *J* = 5.4 Hz, 1H), 8.21 (d, *J* = 8.3 Hz, 2H), 8.09 (d, *J* = 8.0 Hz, 1H), 7.93 (d, *J* = 4.6 Hz, 1H), 7.85 (dd, *J* = 8.0, 2.1 Hz, 1H), 7.58 (d, *J* = 8.3 Hz, 1H), 7.47 (s, 1H), 7.20 (d, *J* = 5.4 Hz, 1H), 7.09 (d, *J* = 8.3 Hz, 1H), 3.97 (s, 3H), 3.89 (t, *J* = 6.6 Hz, 2H), 3.63 (t, *J* = 4.4 Hz, 4H), 3.60 (s, 2H), 3.56 (s, 2H), 3.5 (br) 3.08 (d, *J* = 7.4 Hz, 7H), 2.87 (t, *J* = 6.6 Hz, 2H), 2.47 – 2.42 (m, 4H).

^{13}C NMR (126 MHz, DMSO) δ 169.7, 159.2, 152.4, 150.3, 150.0, 148.3, 148.2, 147.0, 144.8, 143.0, 141.9, 140.9, 137.7, 137.4, 137.0, 136.6, 133.4, 132.2, 127.5, 126.7, 121.6, 121.2, 121.0, 120.4, 119.5, 118.7, 116.4, 115.0, 113.9, 109.3, 65.7, 62.3, 59.0, 52.7, 52.6, 50.1, 43.1, 35.3, 31.4, 27.4. N.B 2x quaternary carbons not visible in ^{13}C spectra, all 25 expected non-quaternary carbons identified in HSQC (see supplementary spectrum 97 and supplementary spectrum 98).

HRMS (ESI+) (*m/z*): calculated for $[(C48H48N16O4)+H^+]^{2+}$: 457.20950, found: 457.21087

2-(4-(4-(3-(4-((1-methyl-3-(3-methyl-2,4-dioxotetrahydropyrimidin-1(2H)-yl)-1H-indazol-6-yl)methyl)piperazin-1-yl)propyl)-1H-1,2,3-triazol-1-yl)phenyl)-N-(4-morpholinopyridin-3-yl)imidazo[1,2-b]pyridazine-8-carboxamide (28)

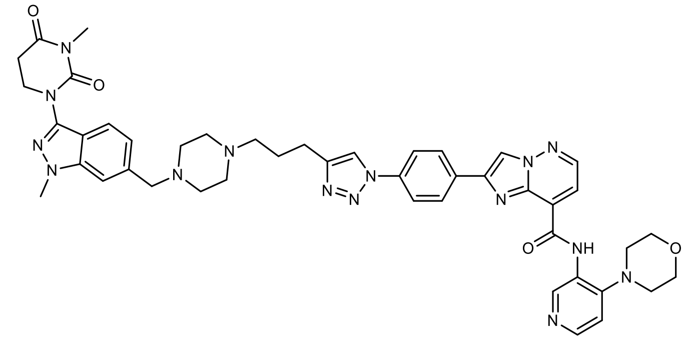

To a stirred solution of **12** (330 µL, 0.1 M, 33 µmol) in DMF at ambient temperature was added Et_3N (10 µL, 73 µmol). The reaction was then stirred for 10 minutes. To the solution was added **57** (490 µL, 0.08 M, 39 µmol) in DMF. The reaction was stirred overnight before being concentrated *in vacuo*. The residue was taken up in MeCN/Water (0.5 mL; 1:1), and purification by preparative HPLC, eluting with 0-100% aq 0.1% formic acid in MeCN, and the collected residue was taken up in DMF/Water (2.5 mL; 1:5) and stirred at ambient temperature. To the solution was added a mixture of mixture of $CuSO_4{*}5H_2O$ (330 µL, 0.05 M, 16 µmol) and THPTA (330 µL, 0.1 M, 33 µmol) in water followed by addition of sodium L-ascorbate (160 µL, 0.2 M, 66 µmol) in water. A solution of **20** (330 µL, 0.1 M, 33 µmol) in DMF was added and the reaction was then stirred overnight before being concentrated *in vacuo*. The residue was taken up in DMSO/Water (0.5 mL; 1:5), and purification by preparative HPLC, eluting with 0-100% aq 0.1% formic acid in MeCN, afforded the title compound as a yellow solid (4.0 mg, 14%).

(ES+): 865 $[M+H^+]^+$.

^{1}H NMR (500 MHz, DMSO) δ 11.74 (s, 1H), 9.24 (s, 1H), 9.17 (s, 1H), 8.81 (d, *J* = 4.7 Hz, 1H), 8.67 (s, 1H), 8.41 (d, *J* = 7.7 Hz, 2H), 8.34 (s, 1H), 8.06 (d, *J* = 7.7 Hz, 2H), 7.94 (d, *J* = 4.7 Hz, 1H), 7.58 (d, *J* = 8.3 Hz, 1H), 7.47 (s, 1H), 7.20 (d, *J* = 4.7 Hz, 1H), 7.09 (d, *J* = 8.3 Hz, 1H), 3.97 (s, 3H), 3.90 (t, *J* = 6.7 Hz, 2H), 3.61 (s, 4H), 3.58 (s, 2H), 3.10 – 3.07 (m, 5H), 2.87

(t, *J* = 6.7 Hz, 2H), 2.73 (t, *J* = 7.6 Hz, 2H), 2.54 (s, 4H), 2.44 – 2.36 (m, 6H), 1.88 – 1.79 (m, 2H).

^{13}C NMR (126 MHz, DMSO) δ 169.9, 159.4, 152.6, 150.4, 148.4, 147.2, 144.9, 143.3, 142.1, 141.1, 137.6, 137.2, 132.0, 127.7, 126.9, 121.8, 121.4, 120.3, 117.9, 116.6, 115.1, 114.1, 109.5, 65.8, 62.6, 57.4, 53.0, 50.2, 43.3, 40.6, 35.5, 31.5, 27.5, 26.3, 23.1. N.B 4 quaternary carbons not visible in ^{13}C spectra, all 24 expected non-quaternary carbons identified in HSQC (see Supplementary spectrum 111).

HRMS (ESI+) (*m/z*): calculated for $[(C45H49N15O4)+H^{+}]^{2+}$: 432.71188, found: 432.71297

NMR Spectra

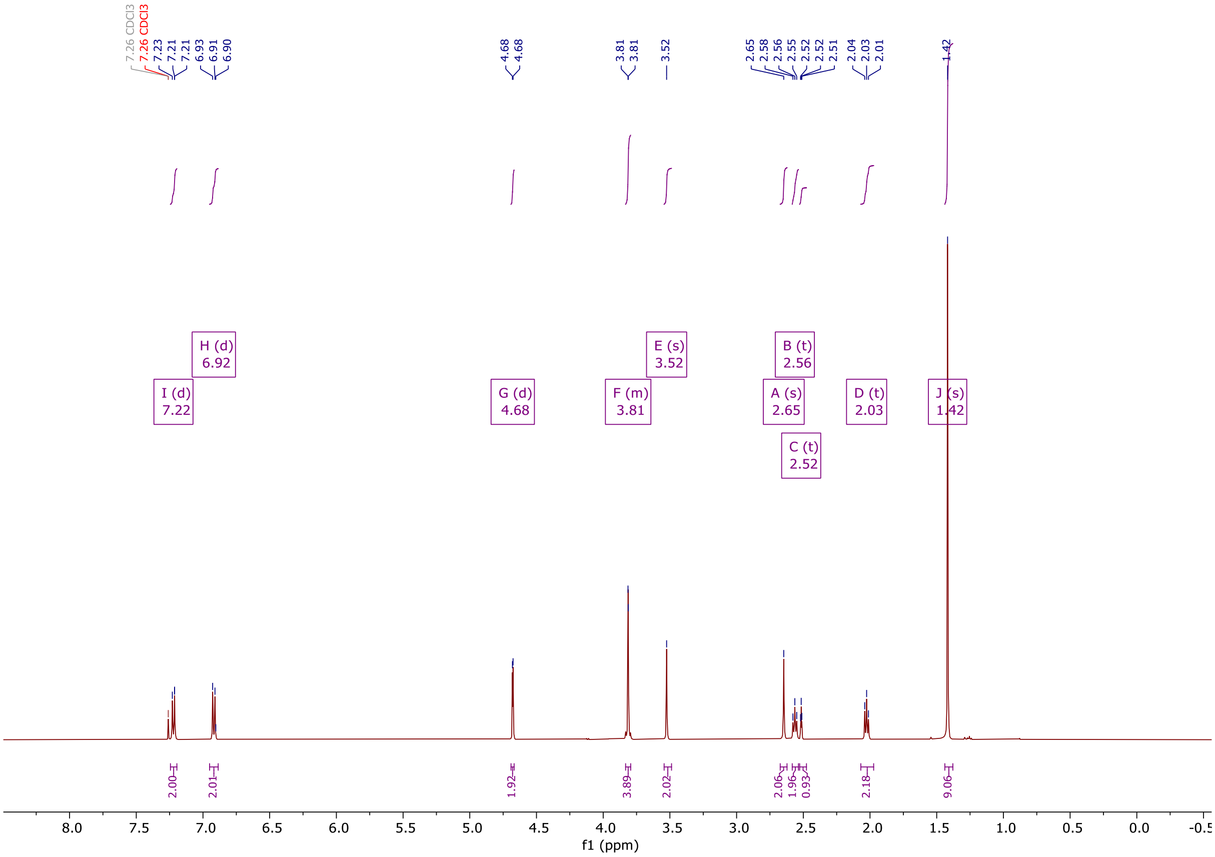

Supplementary spectrum 1: ¹H-NMR spectrum of **33**

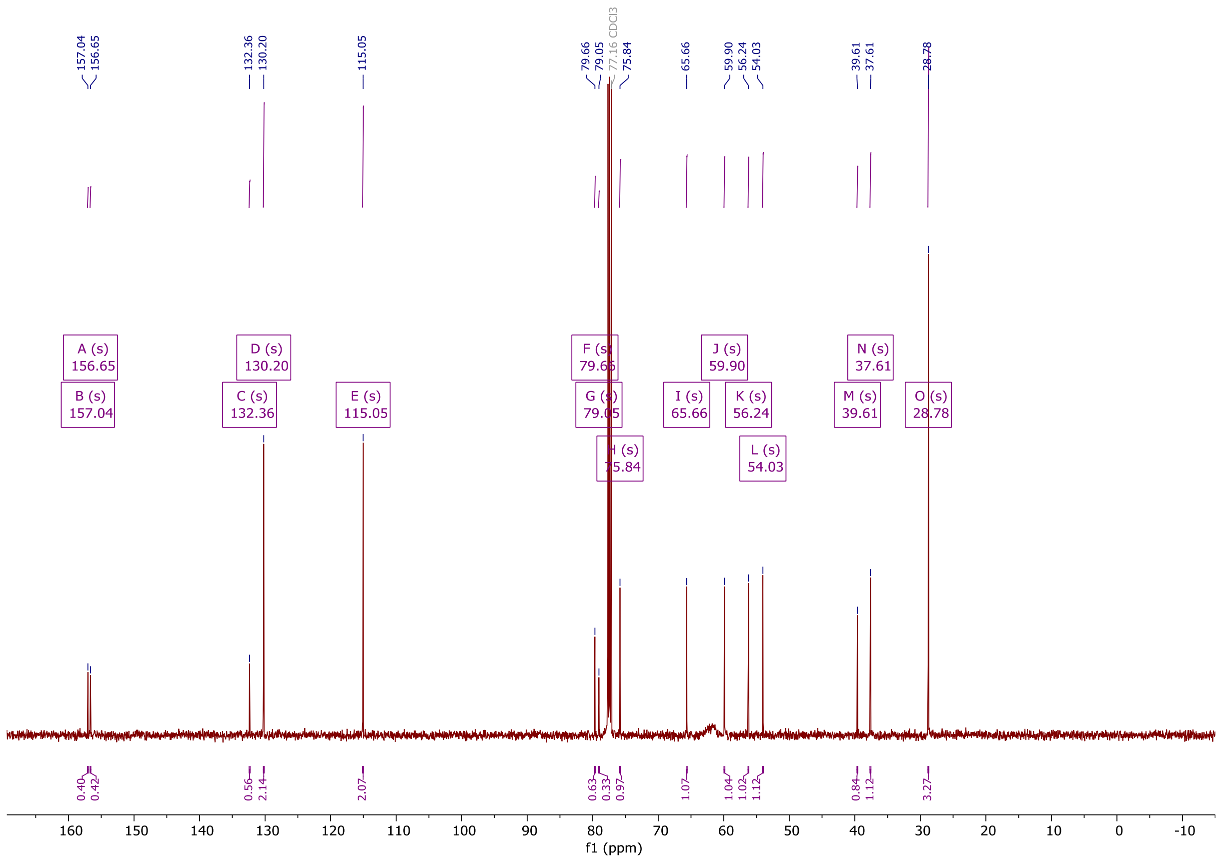

Supplementary spectrum 2: ^{13}C-NMR spectrum of **34**

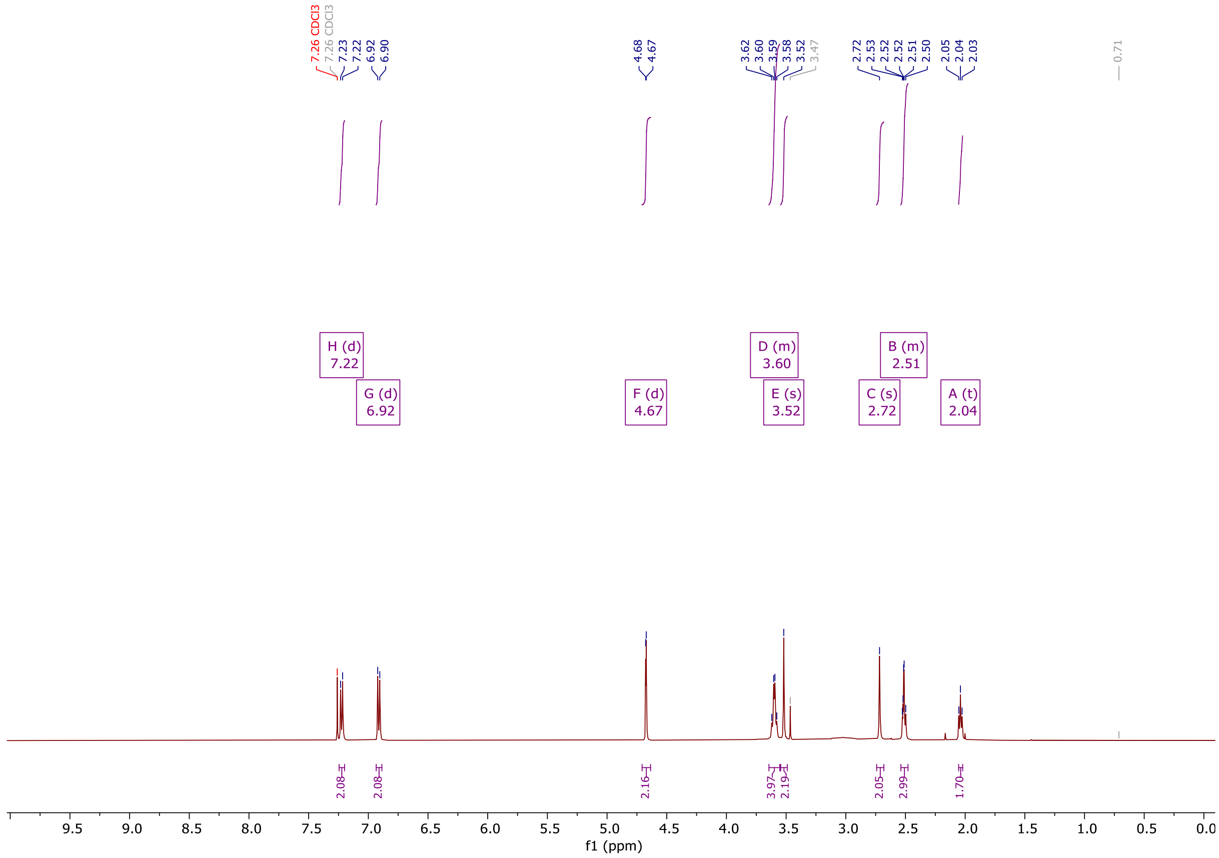

Supplementary spectrum 3: ^{1}H-NMR spectrum of **6**

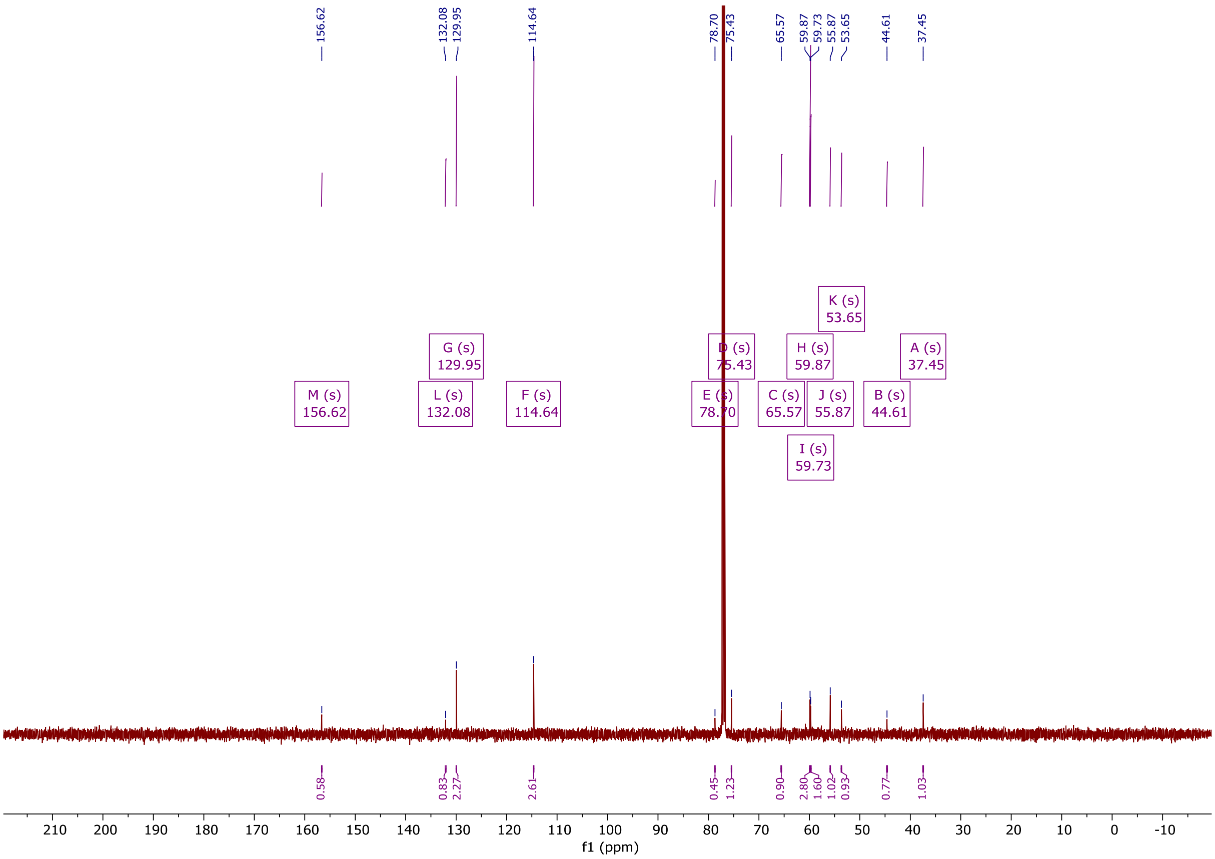

Supplementary spectrum 4: ^{13}C-NMR spectrum of **6**

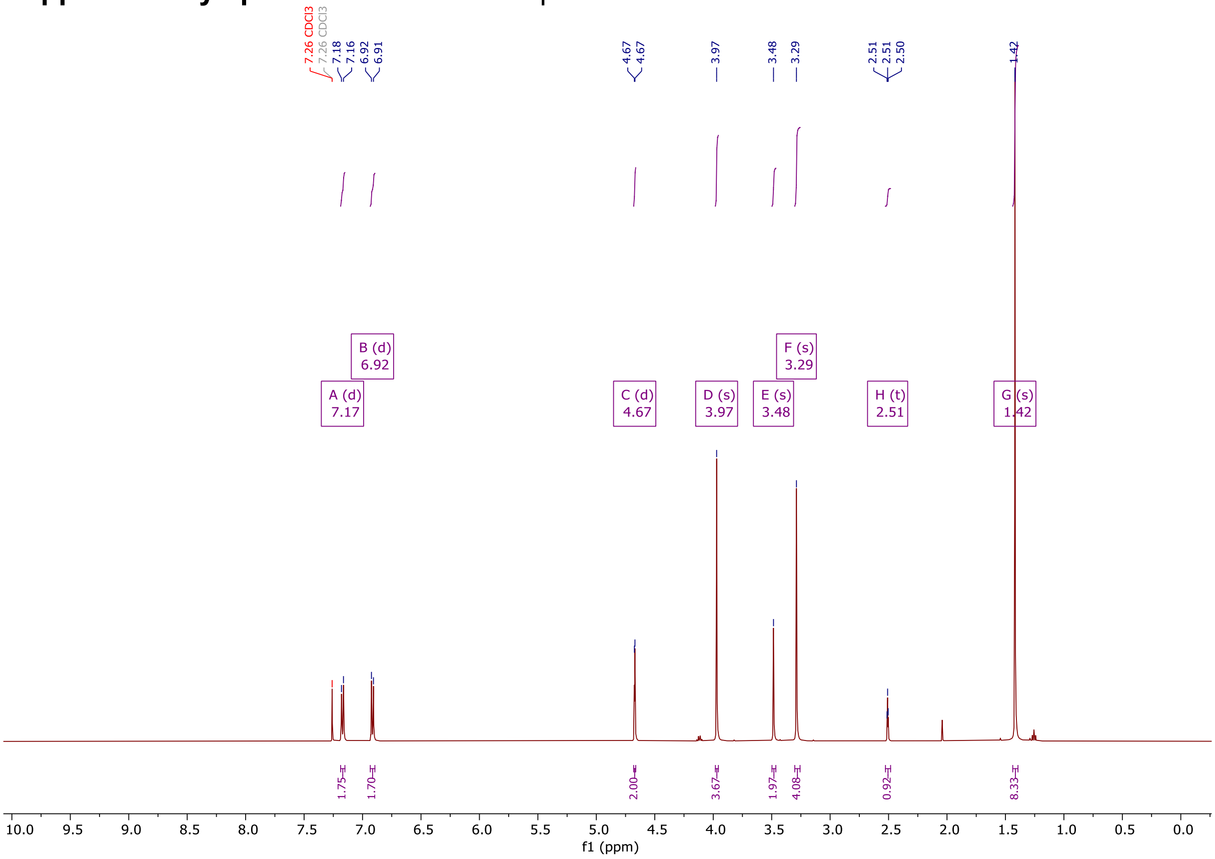

Supplementary spectrum 5: ^{1}H-NMR spectrum of **39**

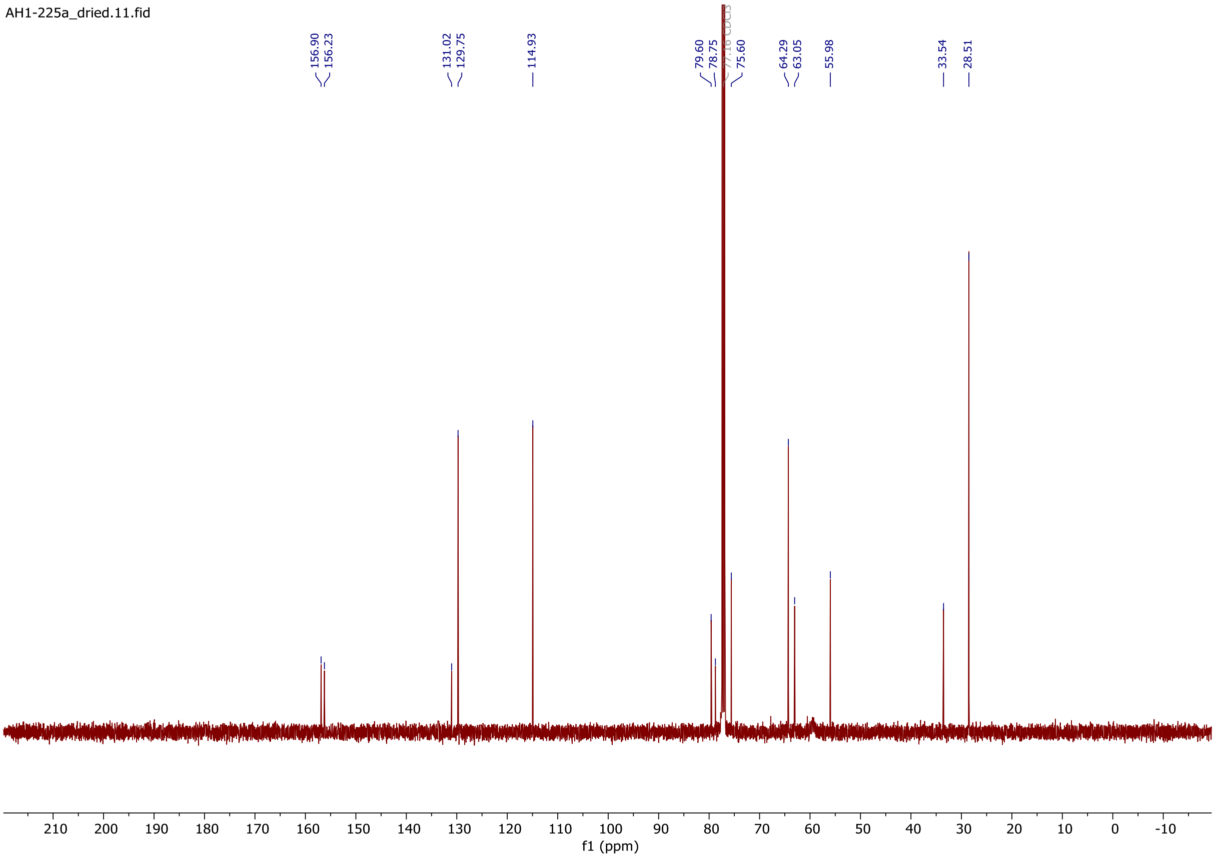

Supplementary spectrum 6: ^{13}C-NMR spectrum of **39**

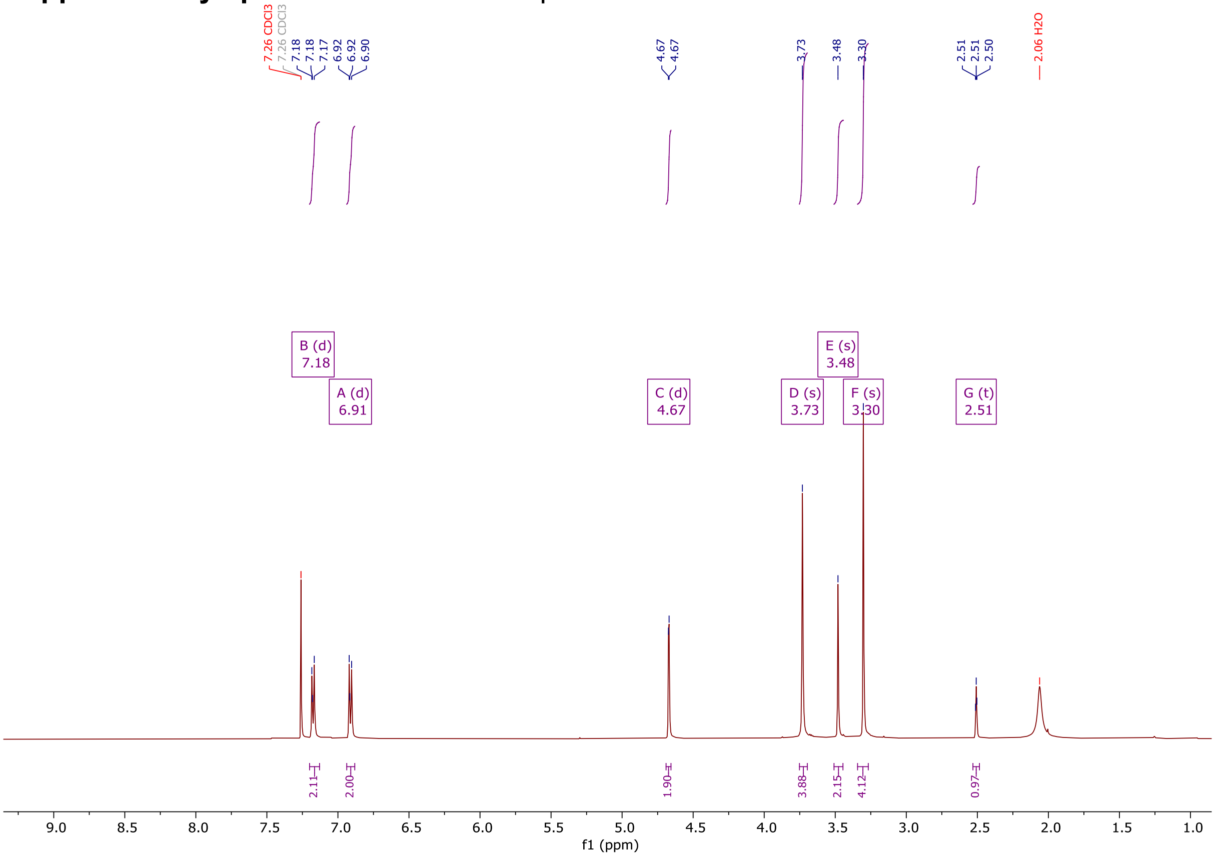

Supplementary spectrum 7: ^{1}H-NMR spectrum of **11**

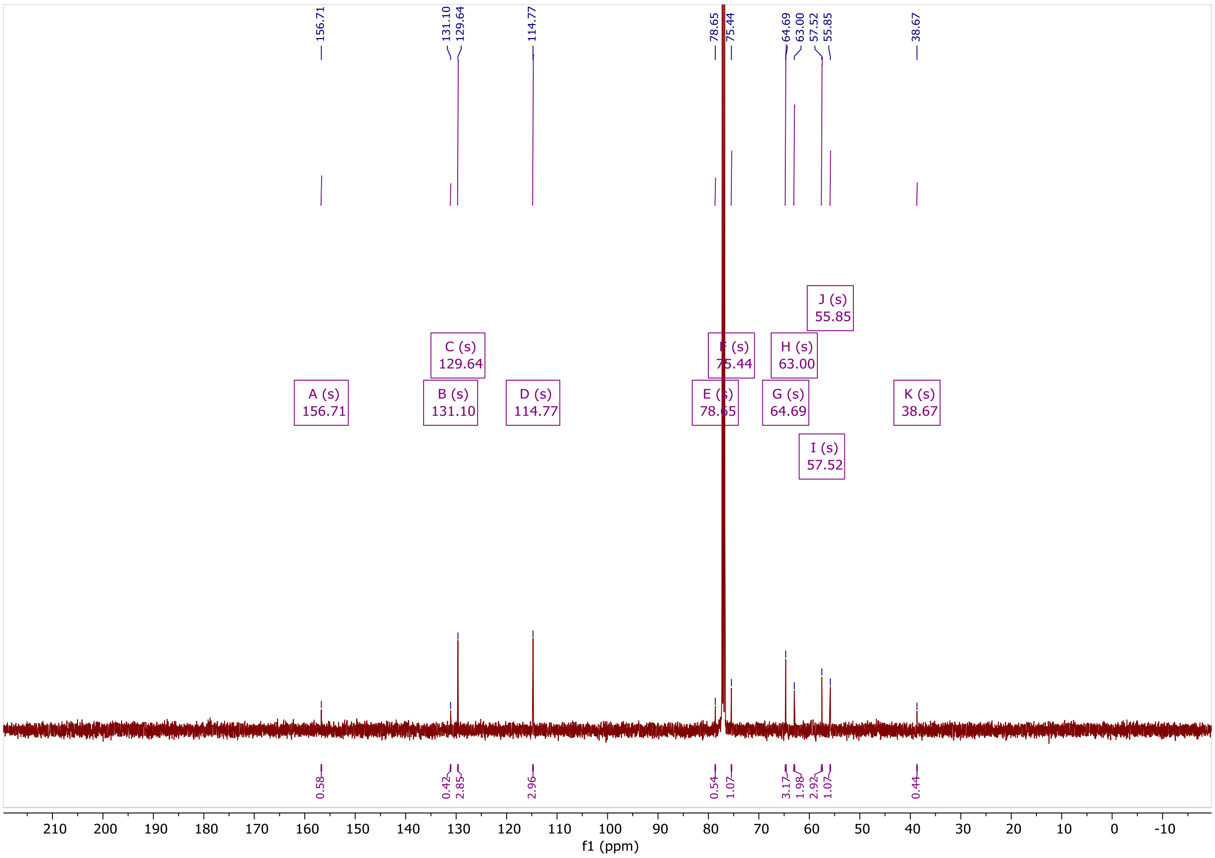

Supplementary spectrum 8: ^{13}C-NMR spectrum of **11**

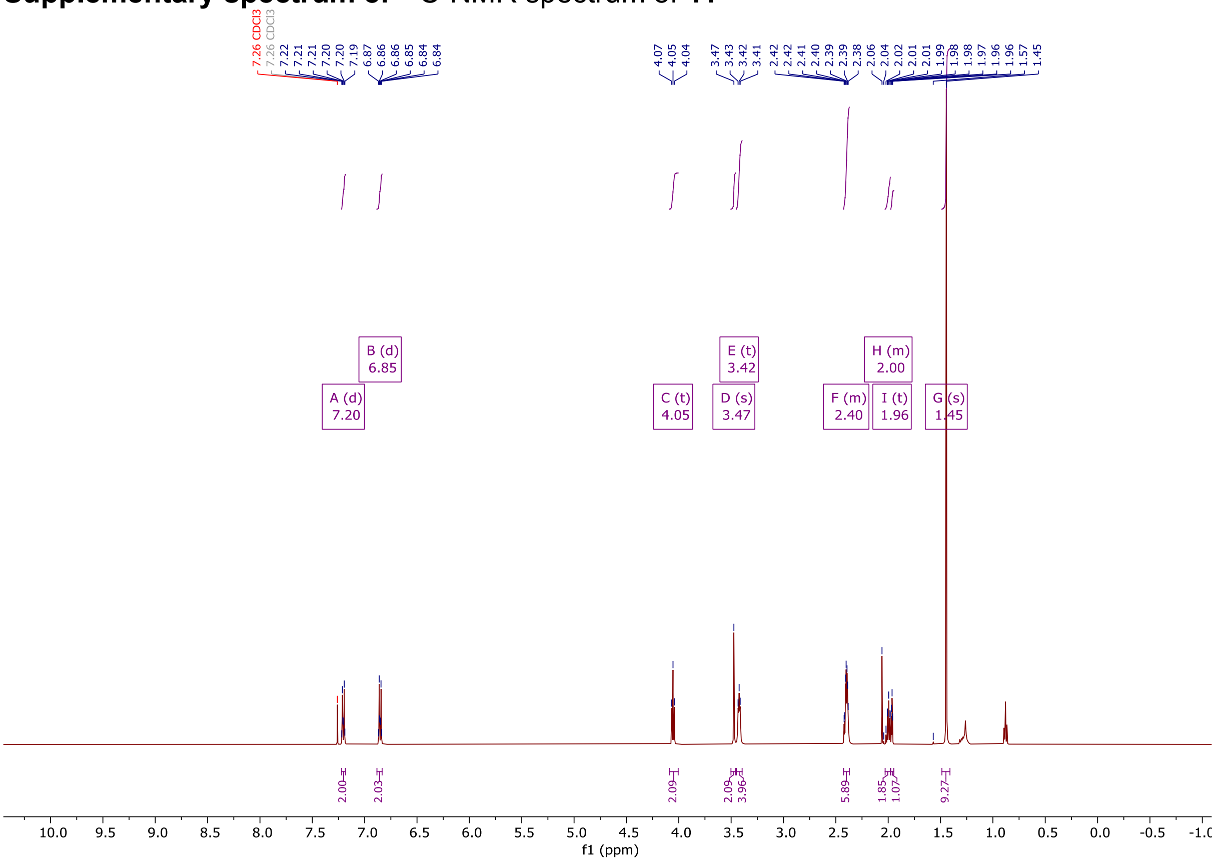

Supplementary spectrum 9: ^{1}H-NMR spectrum of **31**

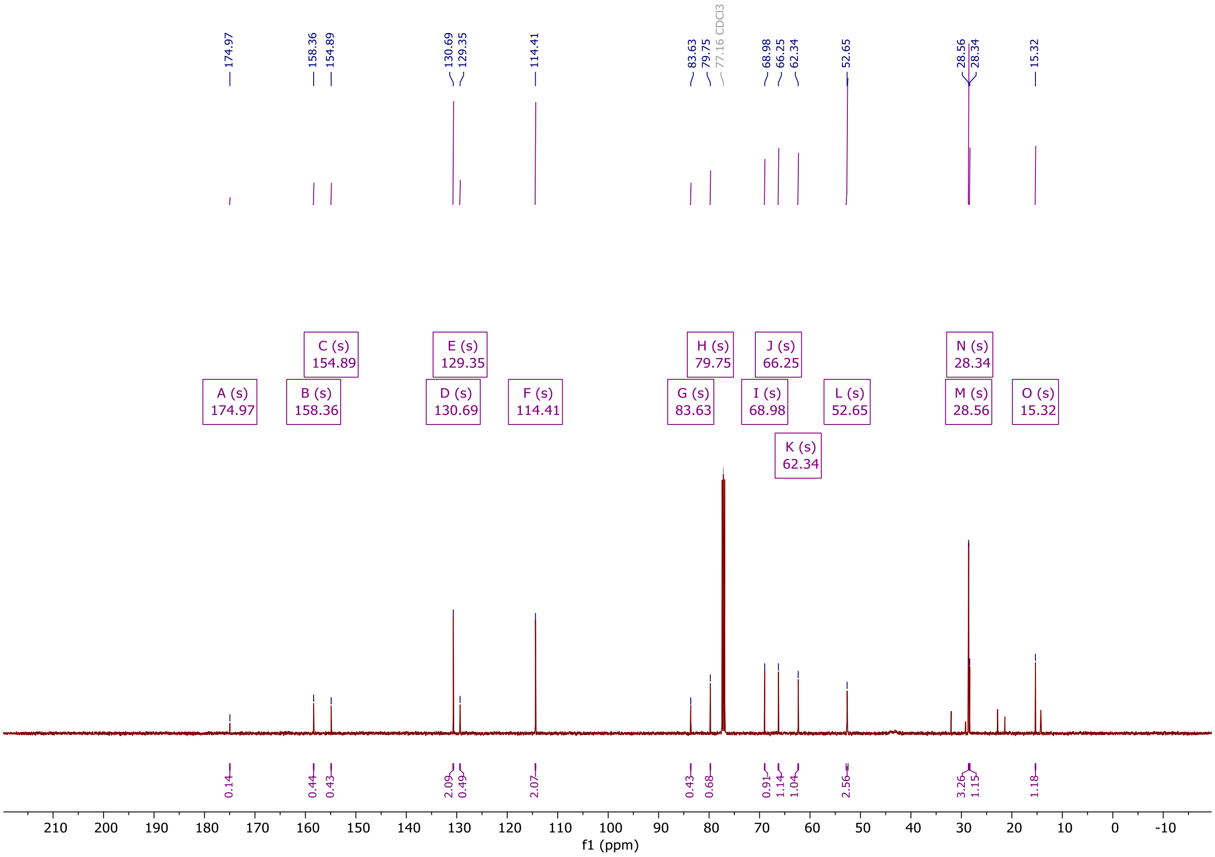

Supplementary spectrum 10: ^{13}C-NMR spectrum of **31**

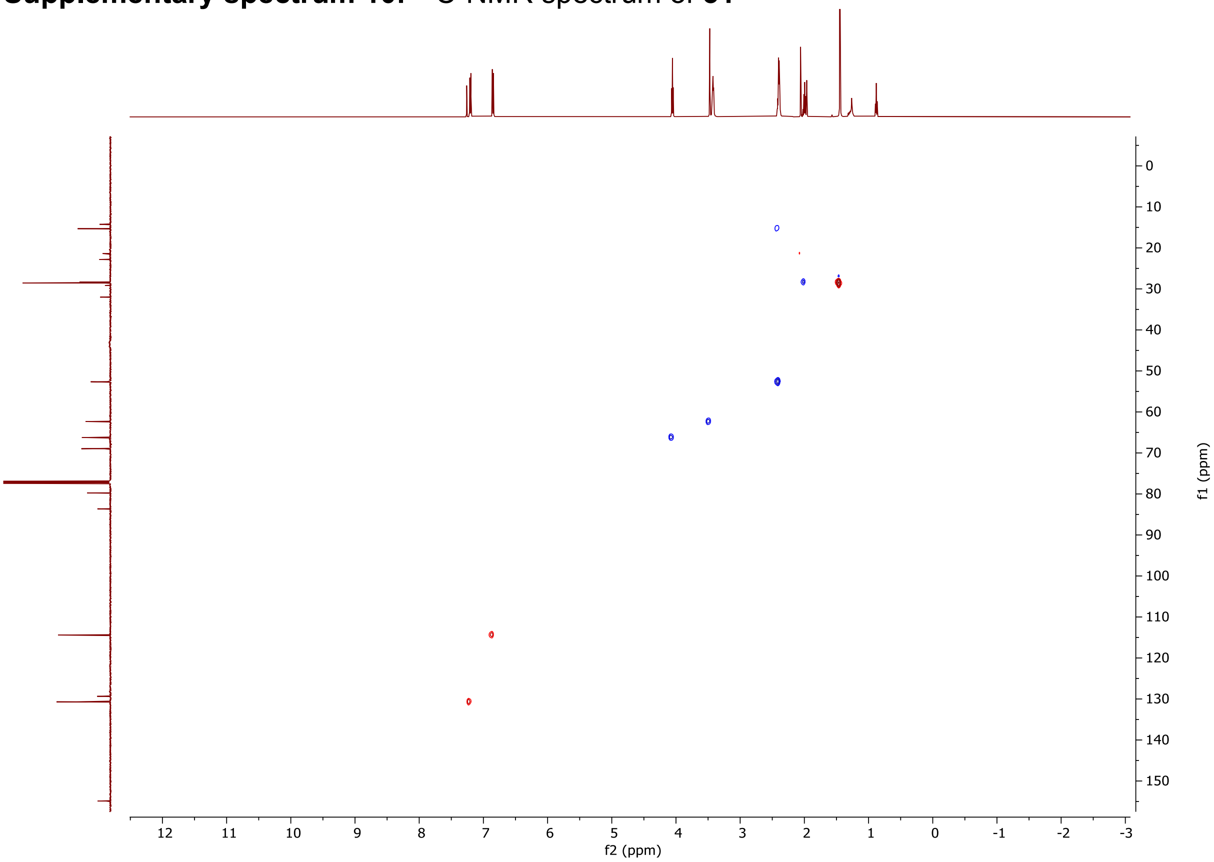

Supplementary spectrum 11: HSQC-NMR spectrum of **31**

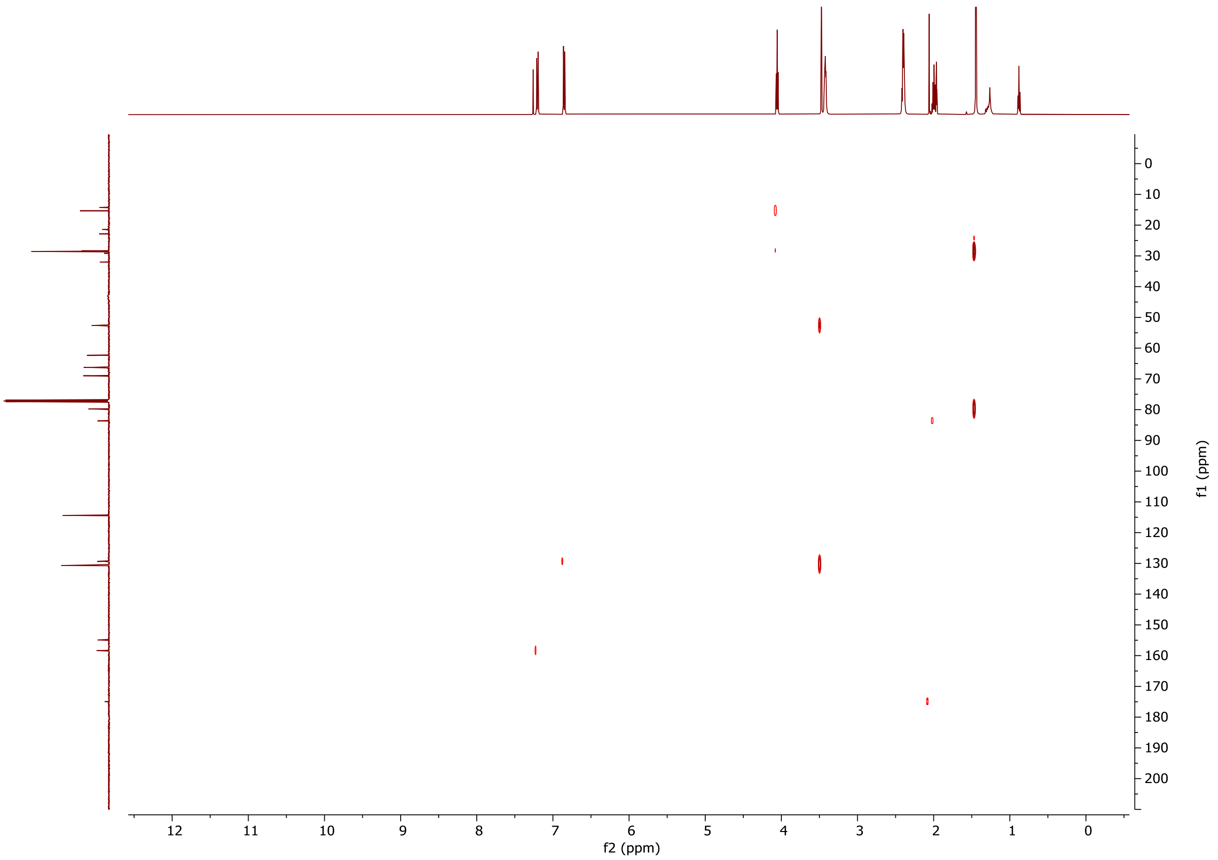

Supplementary spectrum 12: HSBC-NMR spectrum of **31**

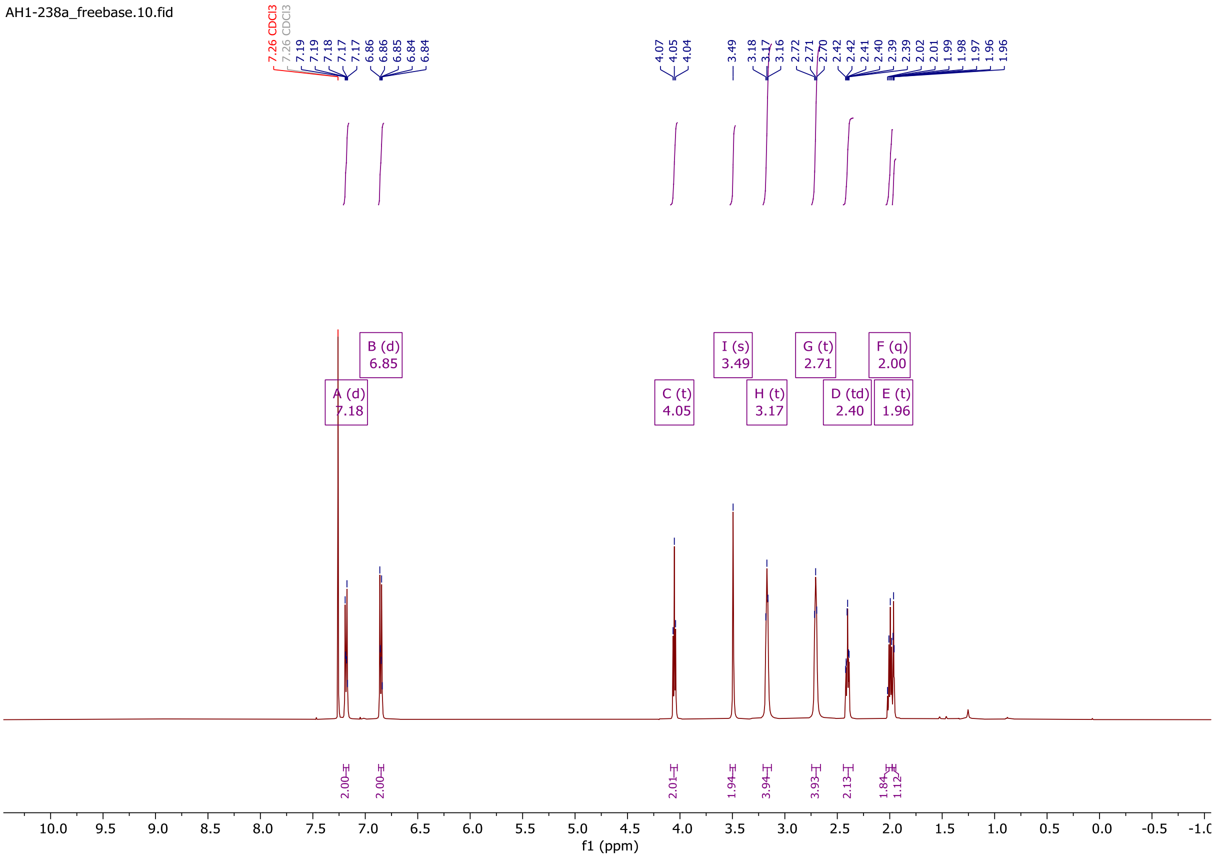

Supplementary spectrum 13: ^{1}H-NMR spectrum of **4**

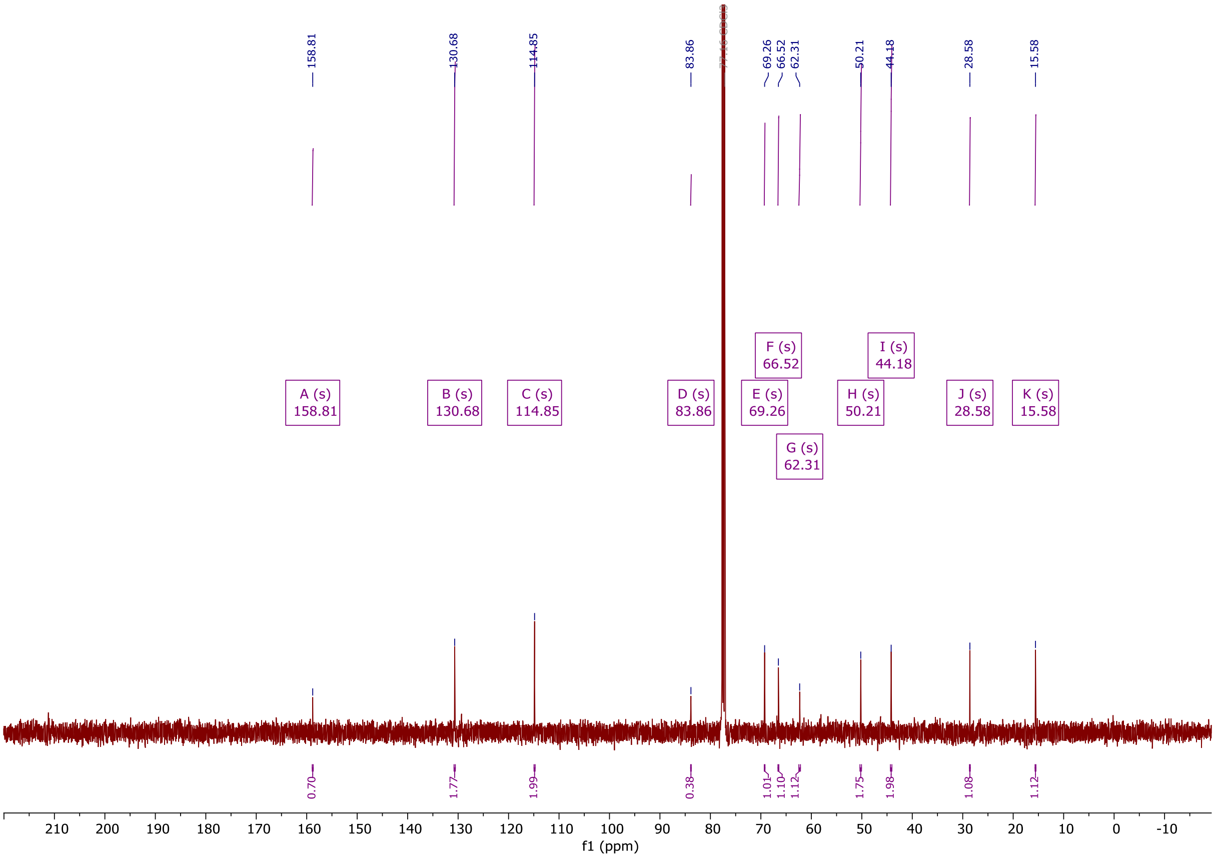

Supplementary spectrum 14: ^{13}C-NMR spectrum of **4**

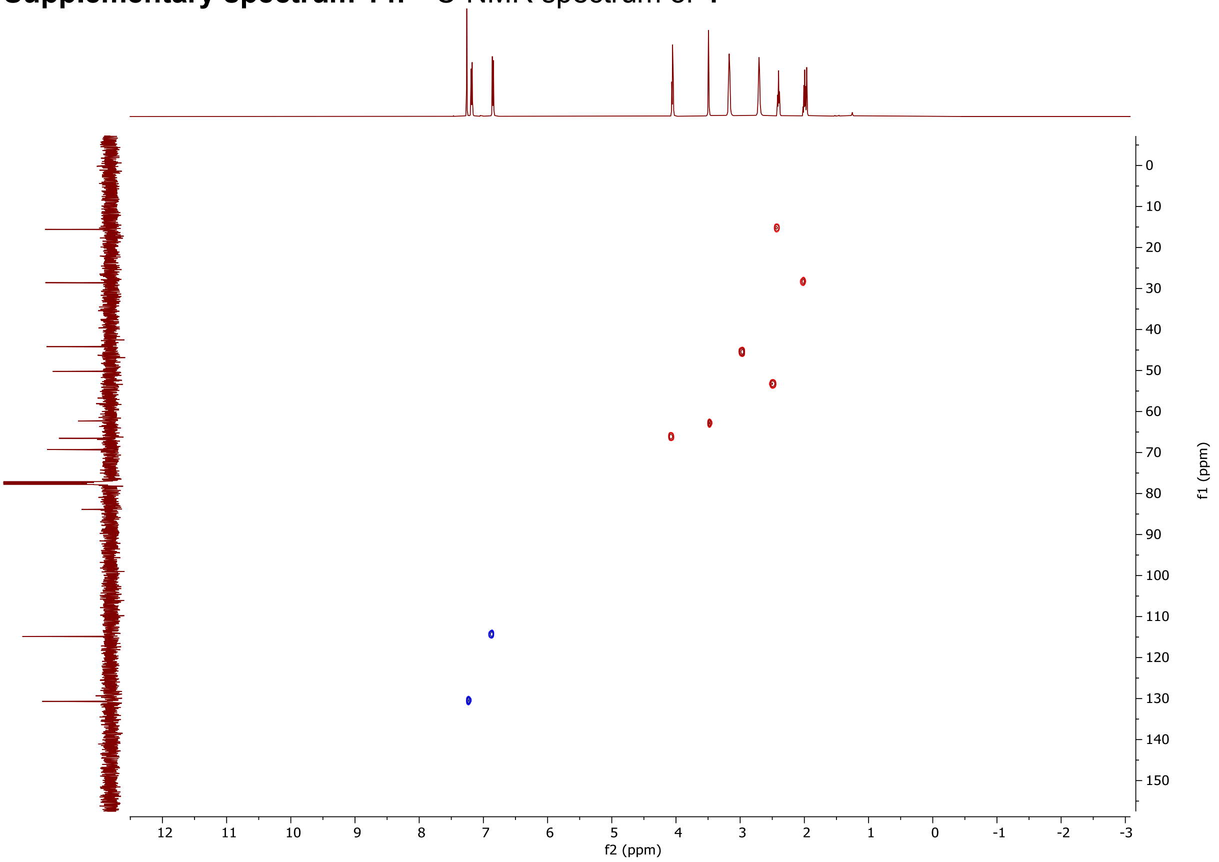

Supplementary spectrum 15: HSQC-NMR spectrum of **4**

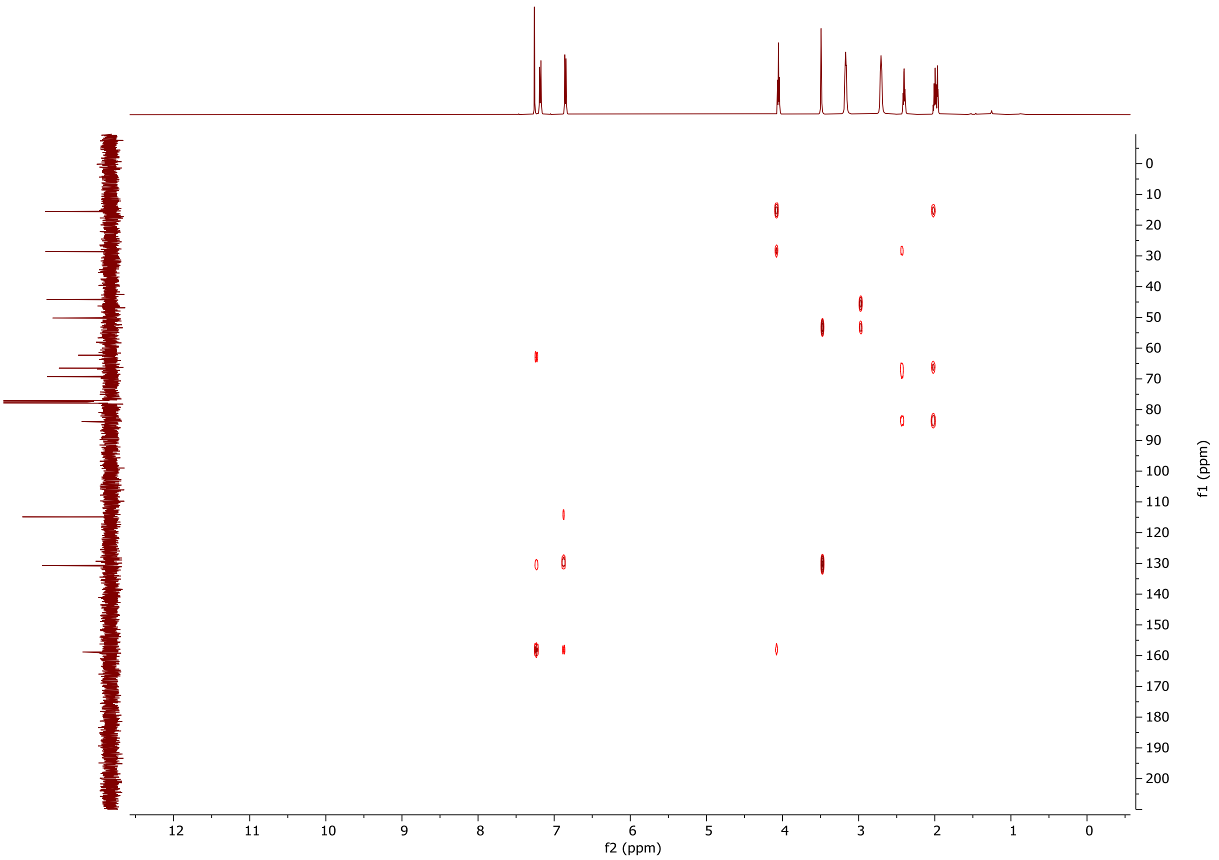

Supplementary spectrum 16: HMBC-NMR spectrum of **4**

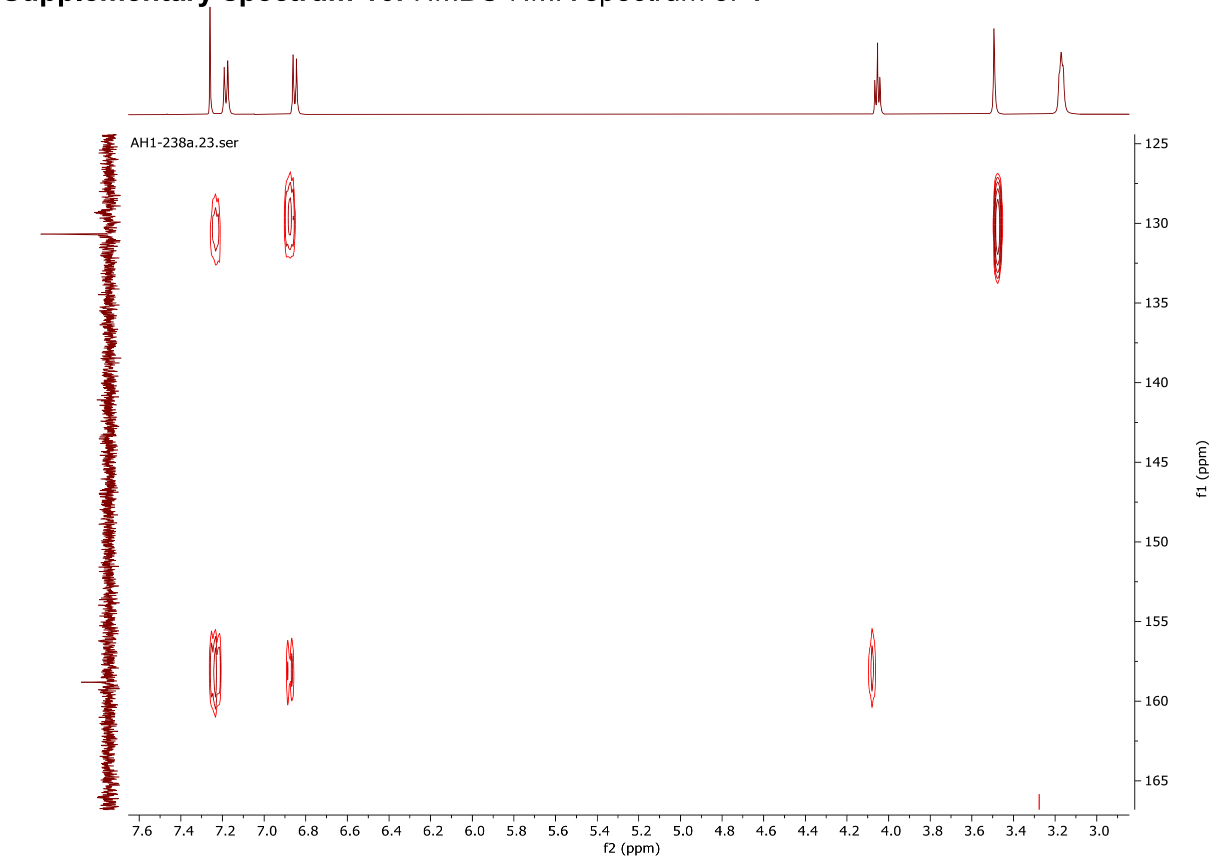

Supplementary spectrum 17: Zoomed in HMBC-NMR spectrum of **4**

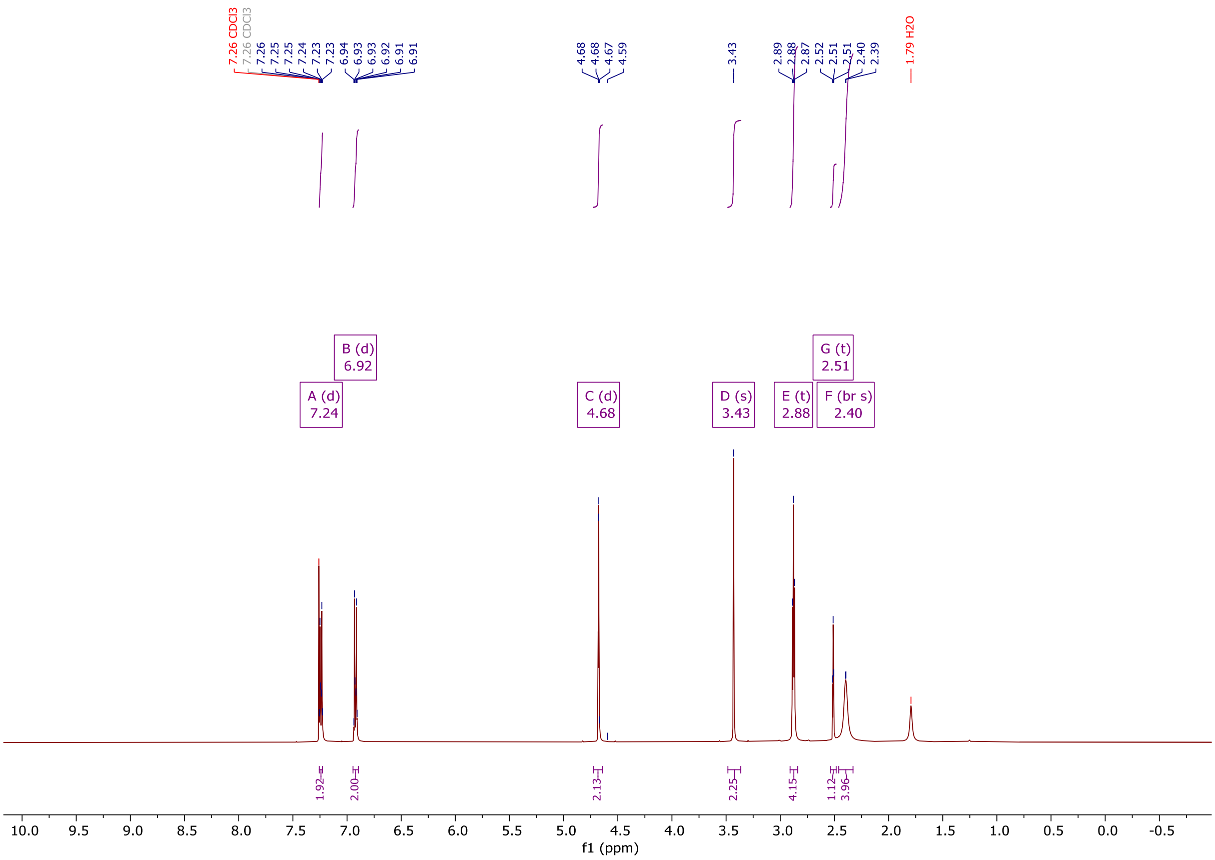

Supplementary spectrum 18: ^{1}H-NMR spectrum of **3**

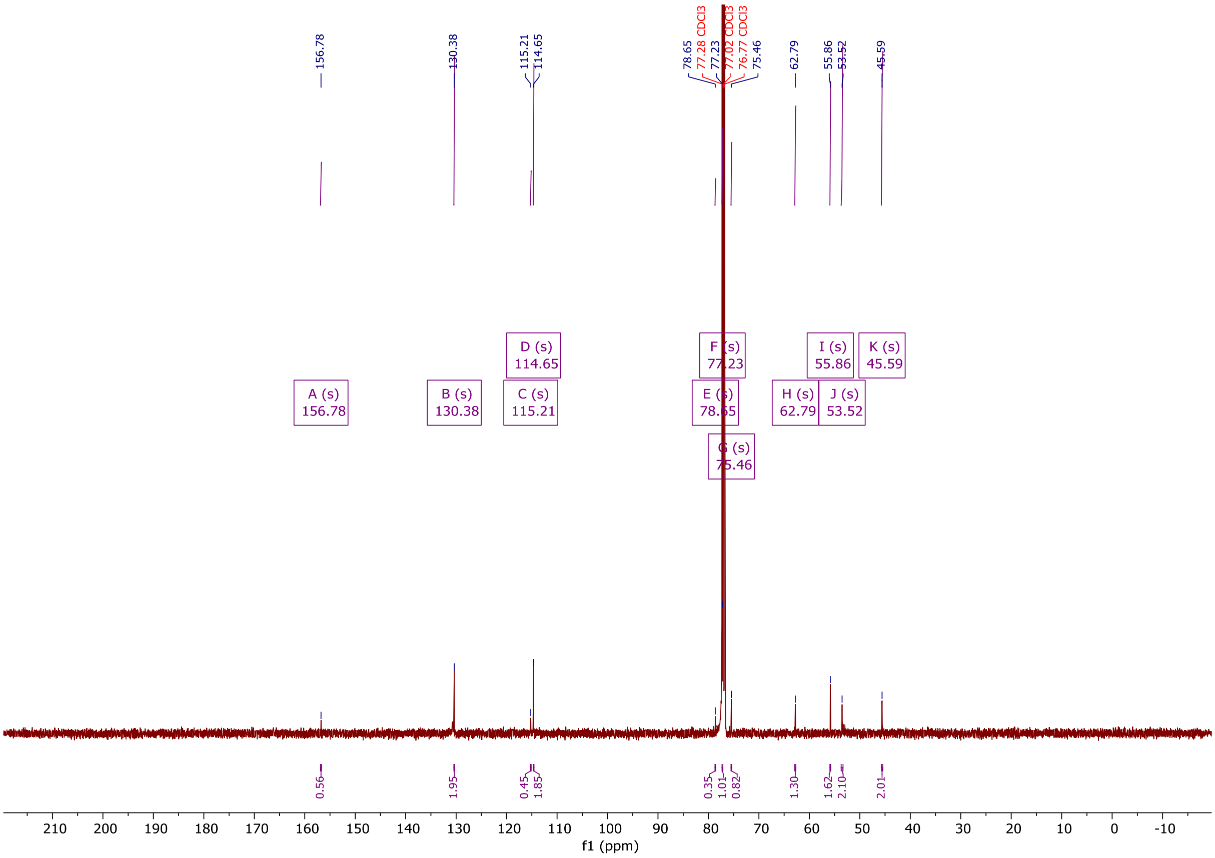

Supplementary spectrum 19: ^{13}C-NMR spectrum of **3**

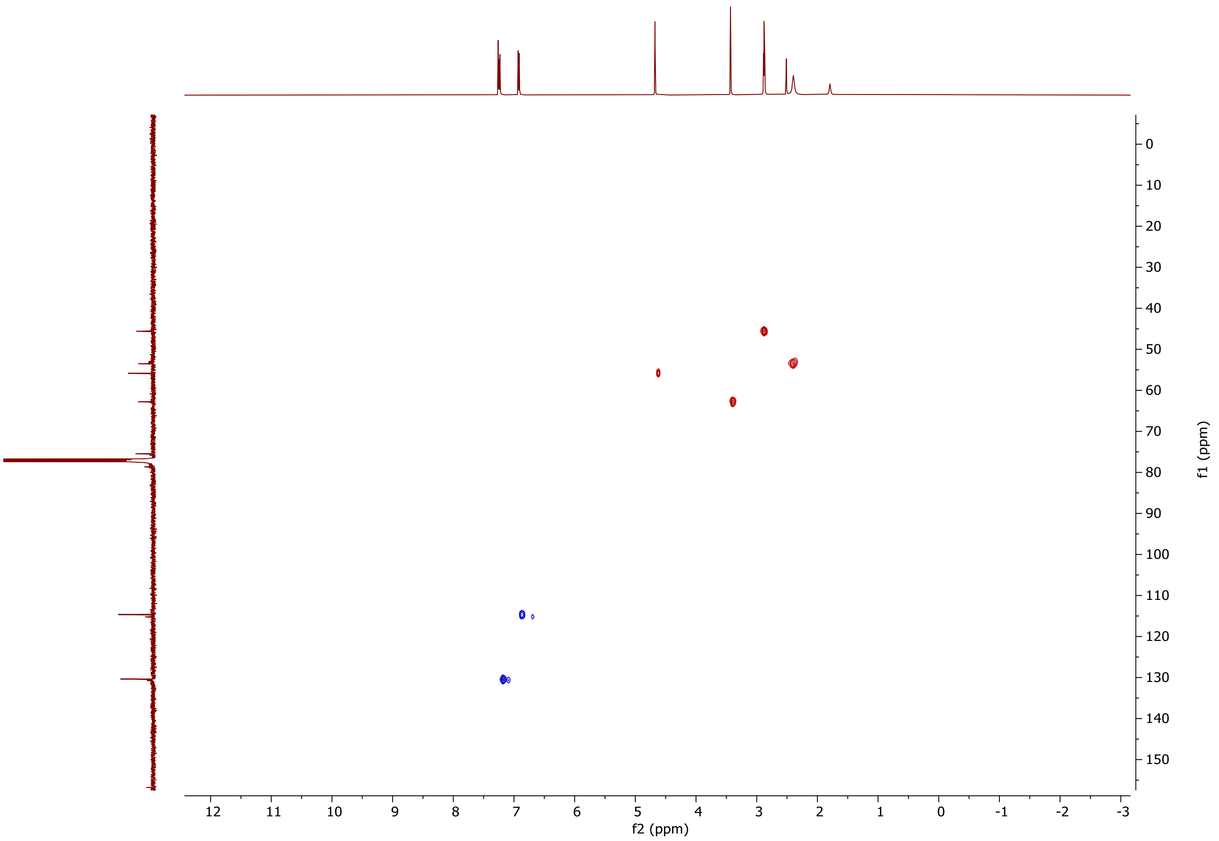

Supplementary spectrum 20: HSQC-NMR spectrum of **3**

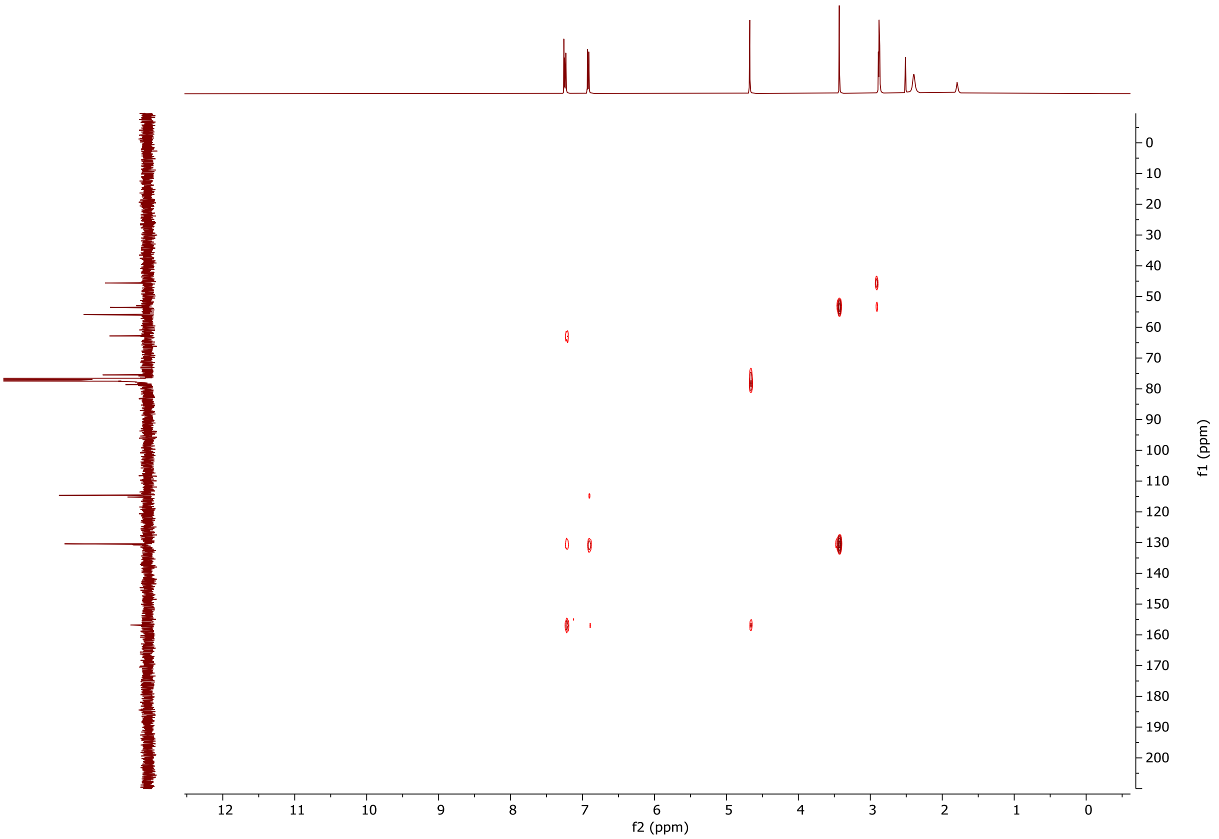

Supplementary spectrum 21: HMBC-NMR spectrum of **3**

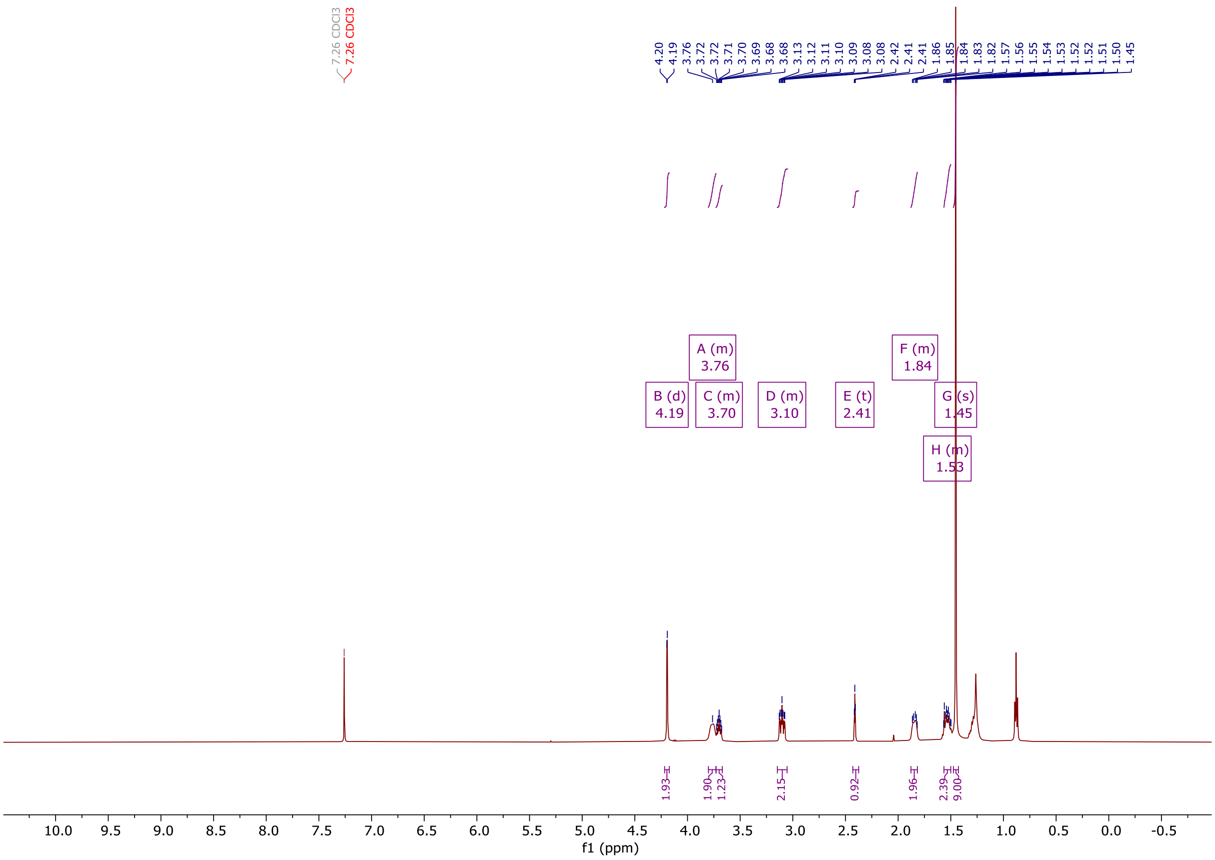

Supplementary spectrum 22: ^{1}H-NMR spectrum of **32**

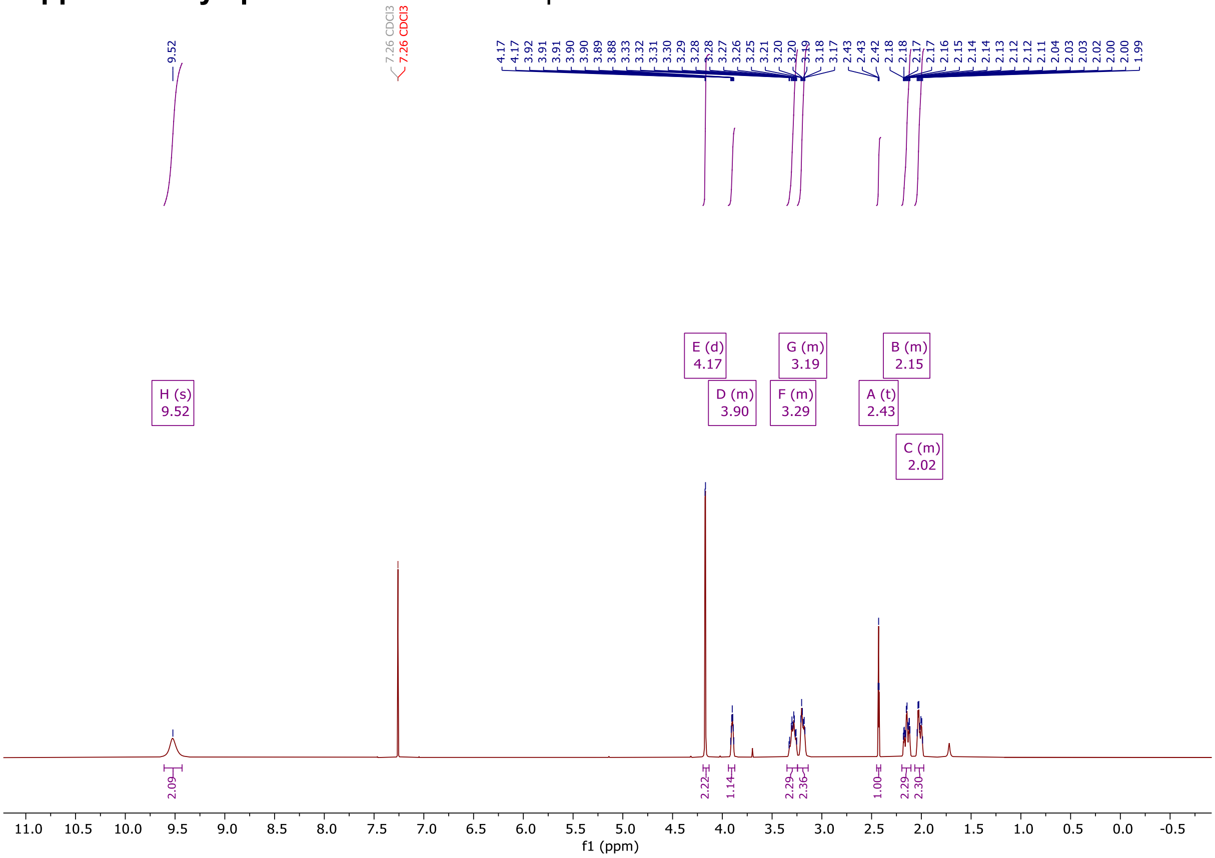

Supplementary spectrum 23: ^{1}H-NMR spectrum of **5**

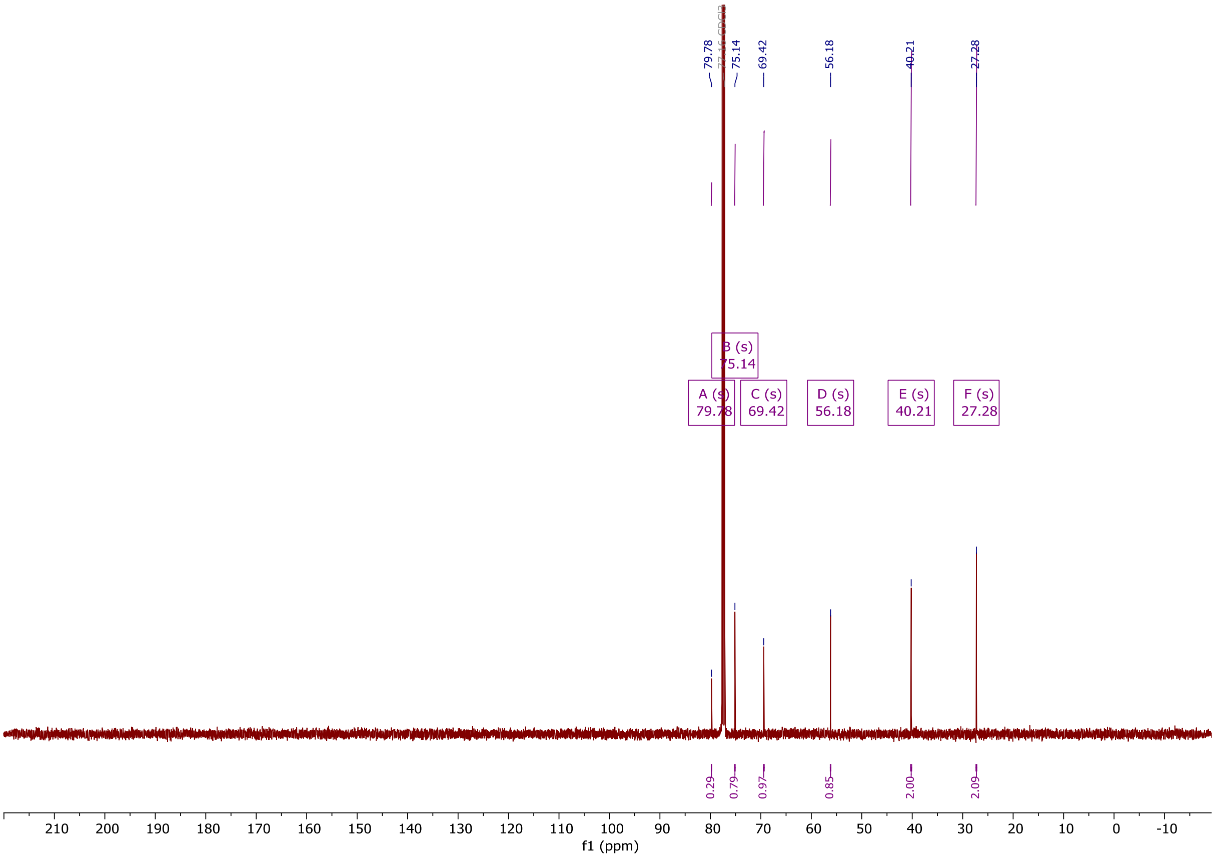

Supplementary spectrum 24: ^{13}C-NMR spectrum of **5**

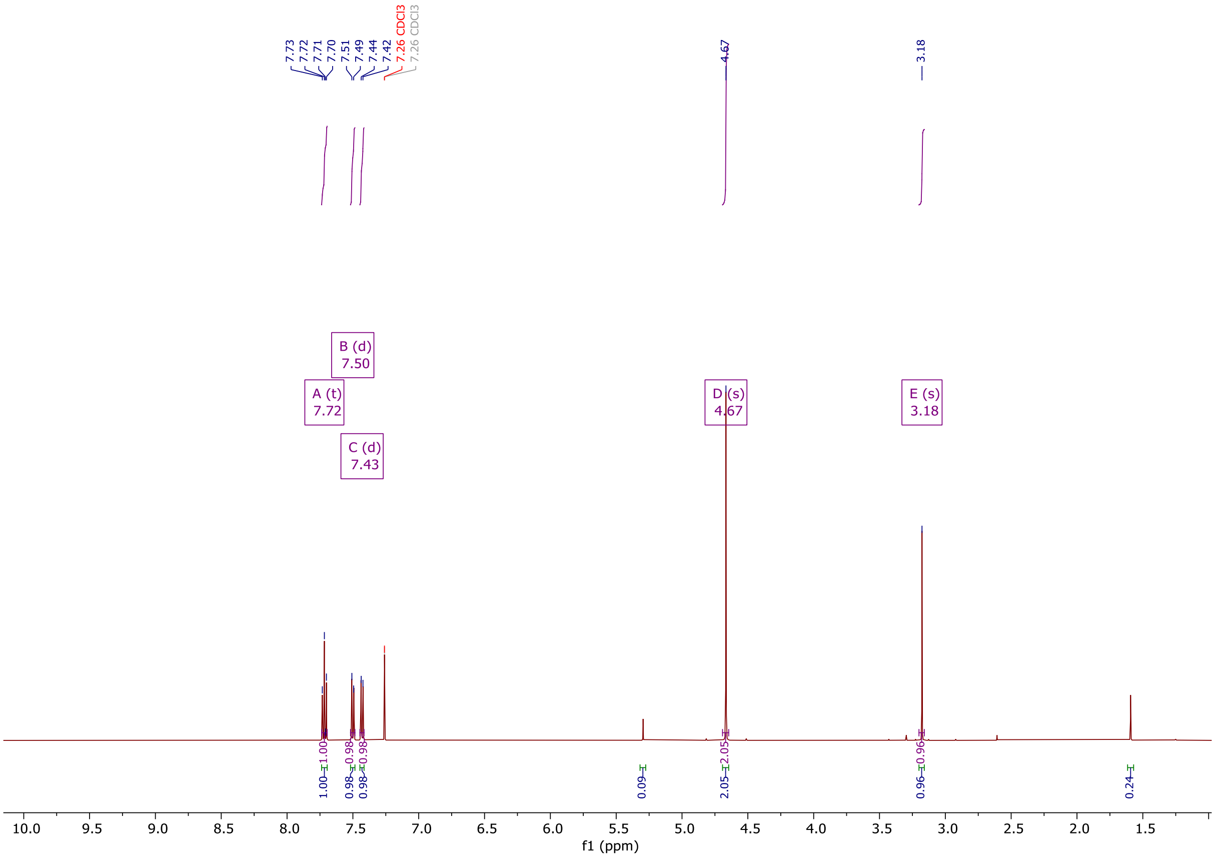

Supplementary spectrum 25: ^{1}H-NMR spectrum of **35**

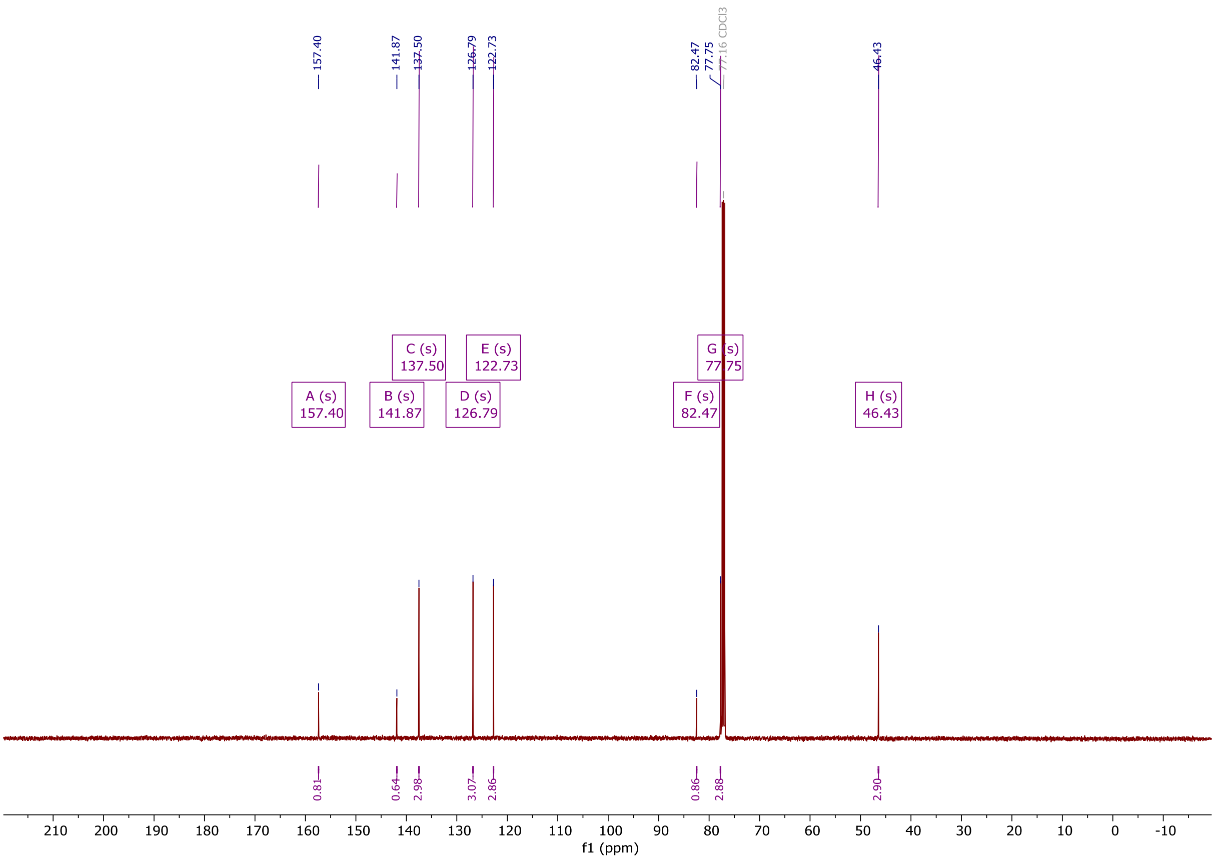

Supplementary spectrum 26: ^{13}C-NMR spectrum of **35**

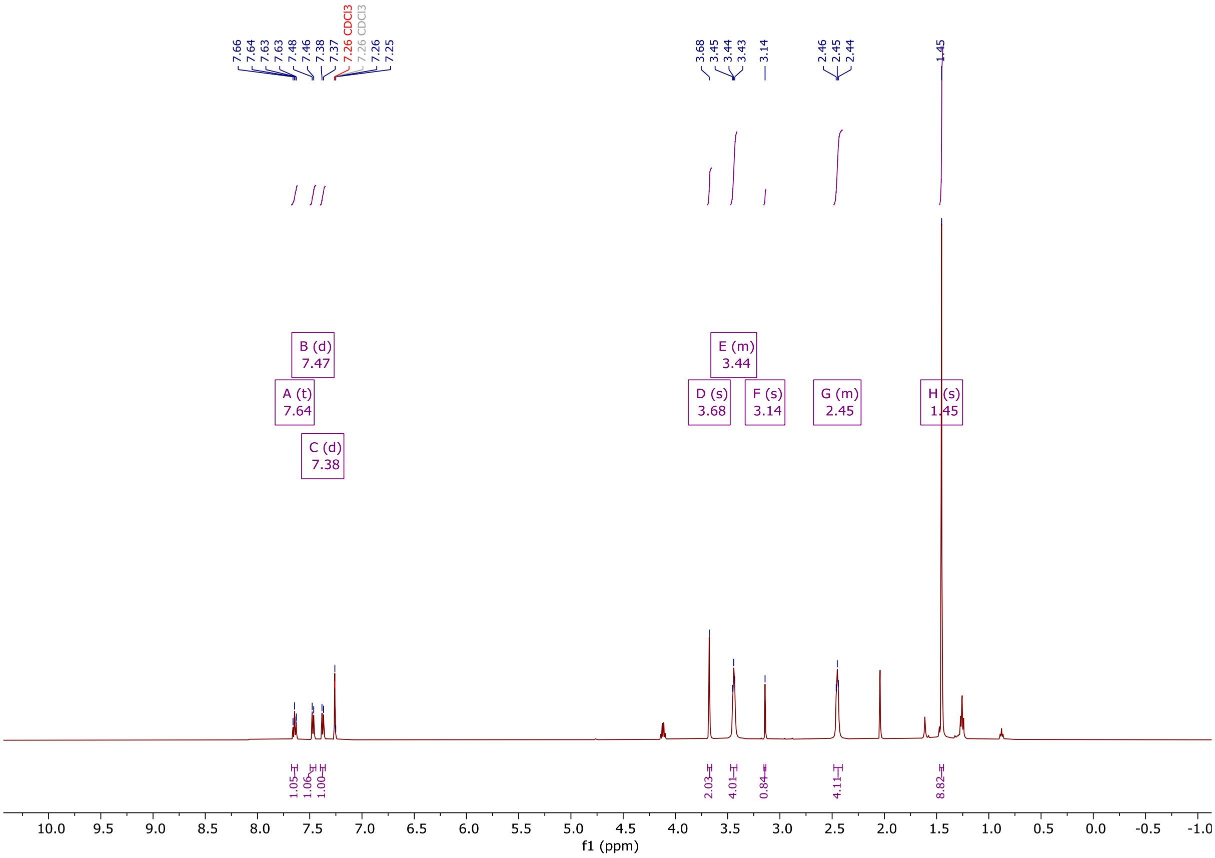

Supplementary spectrum 27: ^{1}H-NMR spectrum of **36**

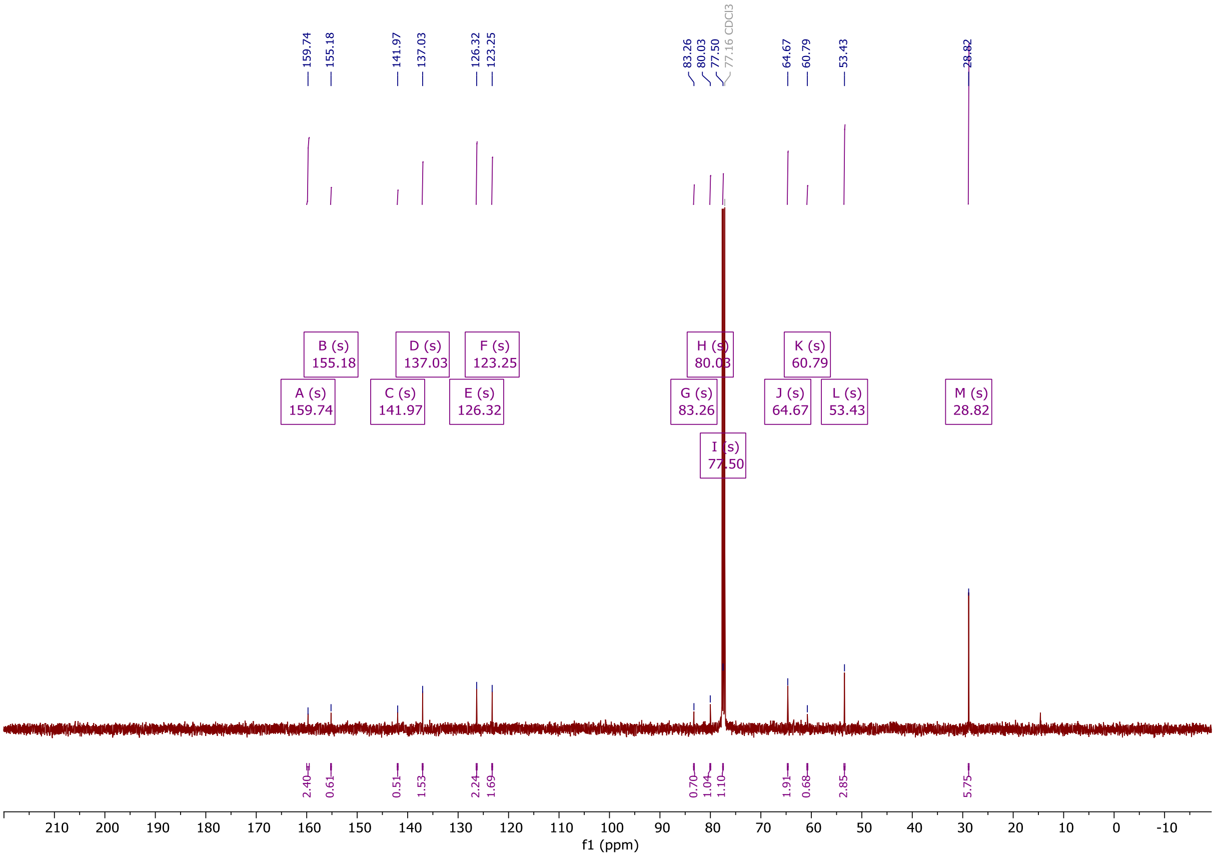

Supplementary spectrum 28: ^{13}C-NMR spectrum of **36**

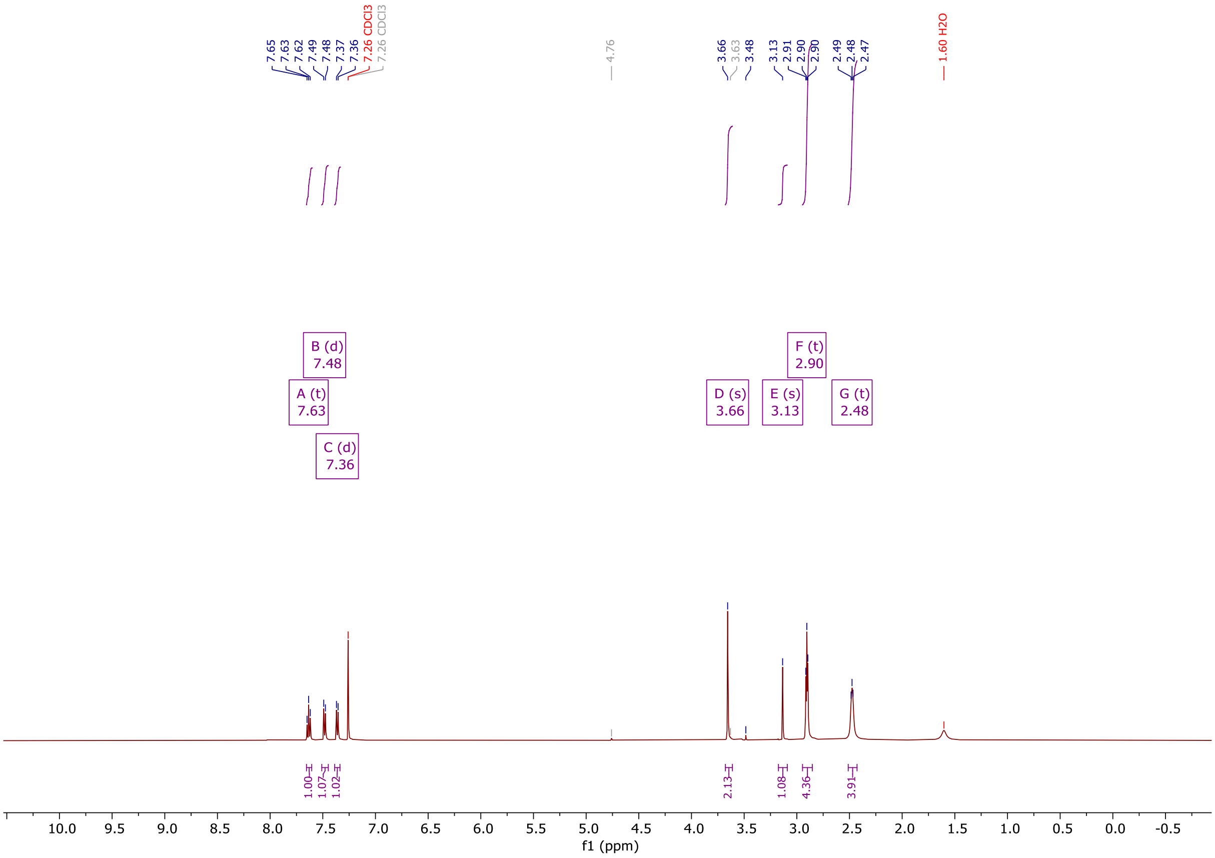

Supplementary spectrum 29: ^{1}H-NMR spectrum of **8**

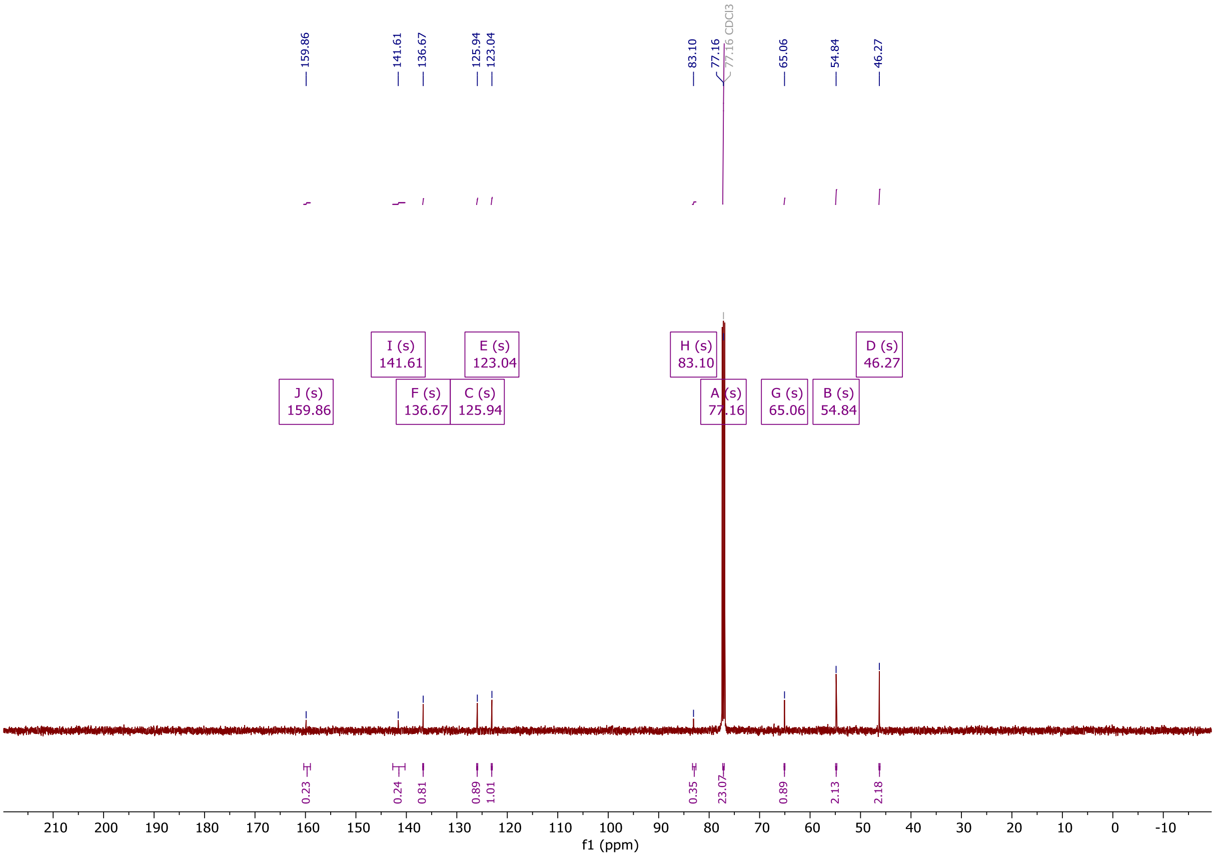

Supplementary spectrum 30: ^{13}C-NMR spectrum of **8**.

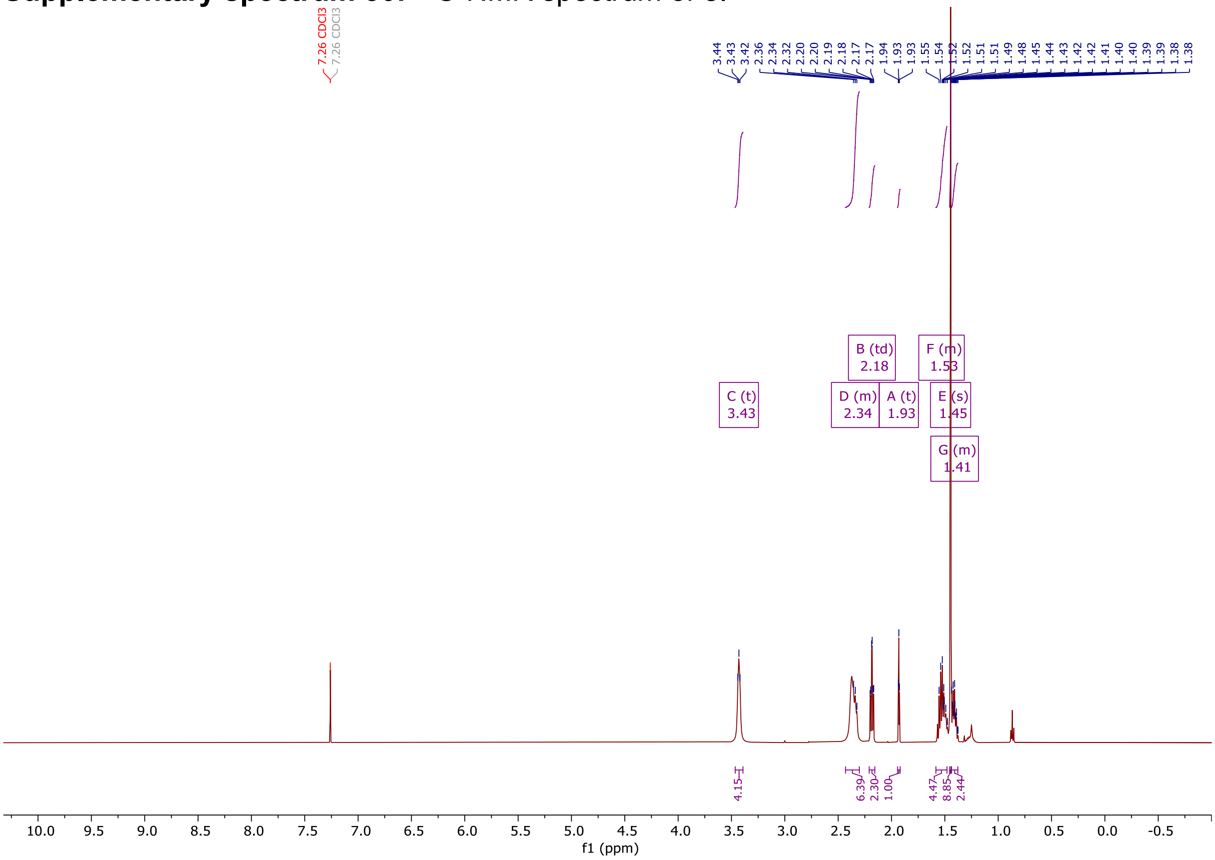

Supplementary spectrum 31: ^{1}H-NMR spectrum of **34**

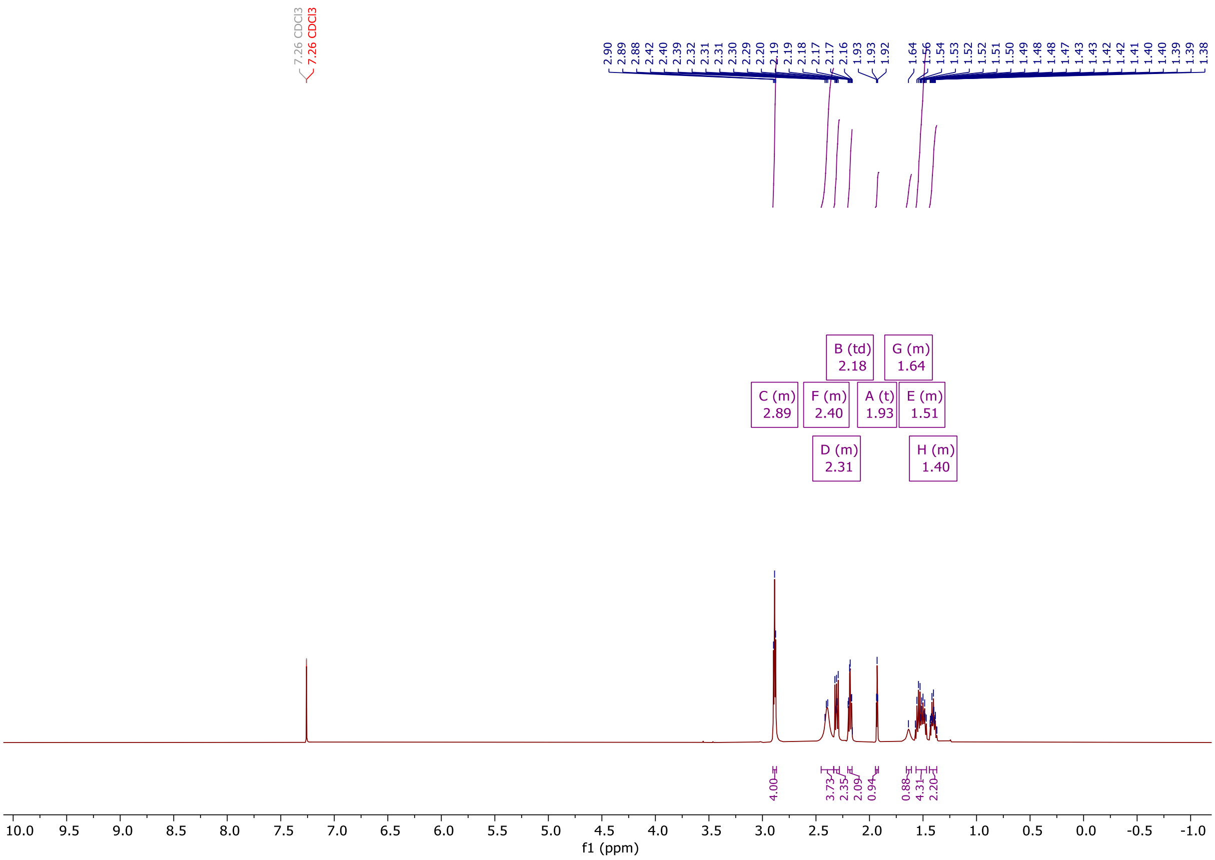

Supplementary spectrum 32: ^{1}H-NMR spectrum of **7**

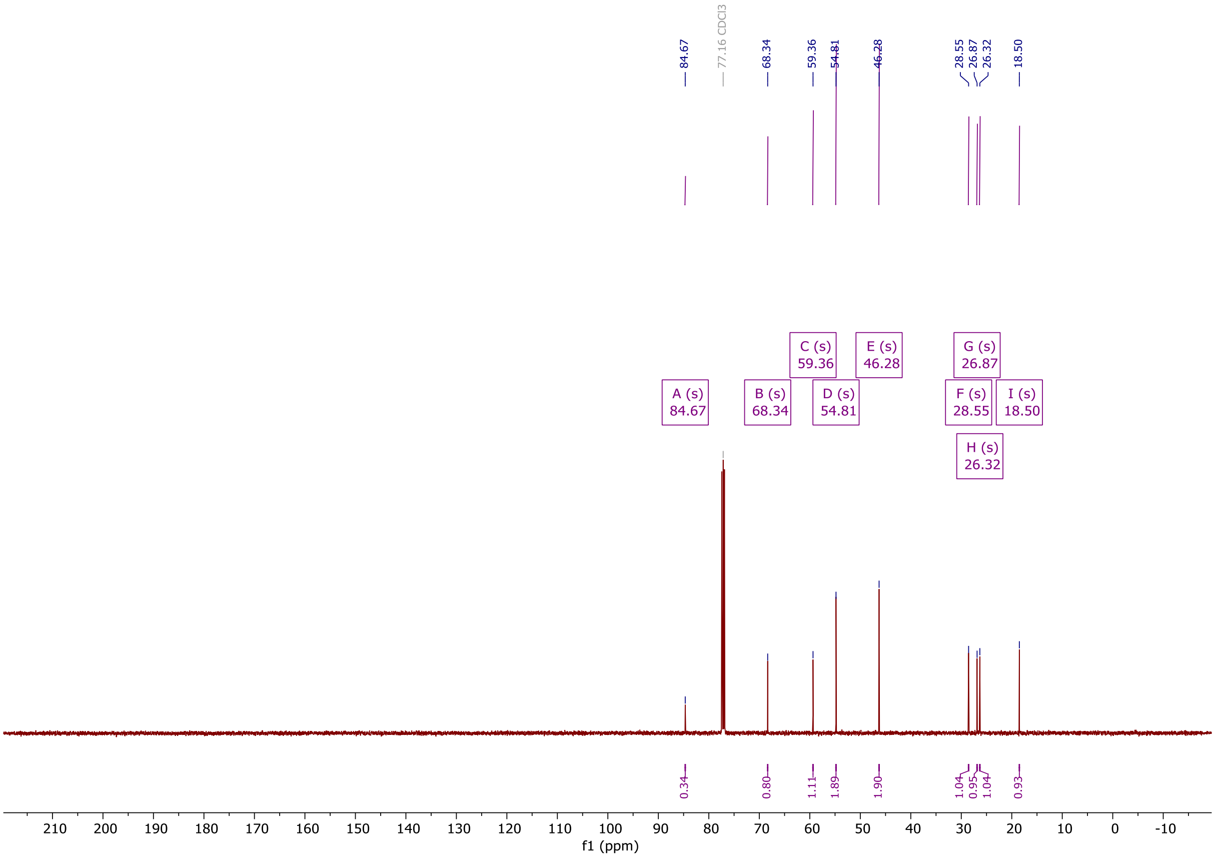

Supplementary spectrum 33: ^{13}C-NMR spectrum of **7**

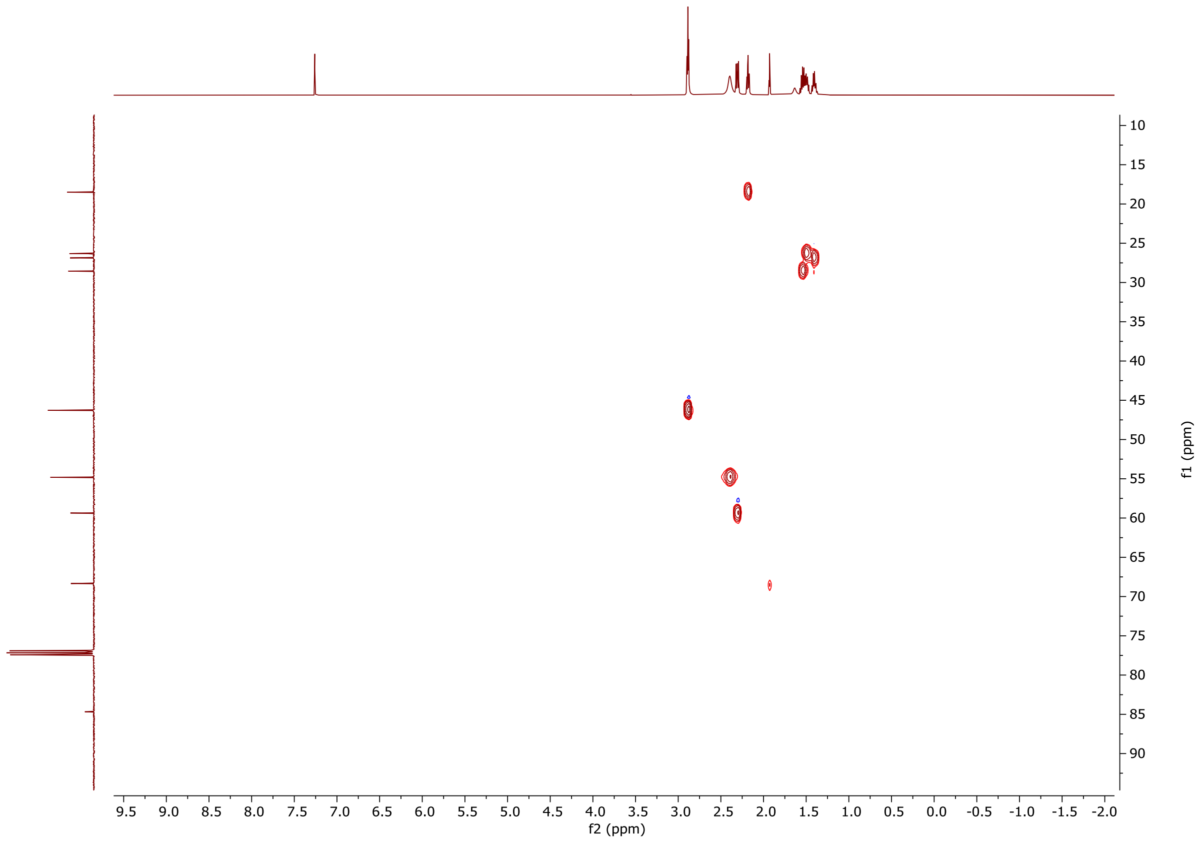

Supplementary spectrum 34: HSQC-NMR spectrum of **7**

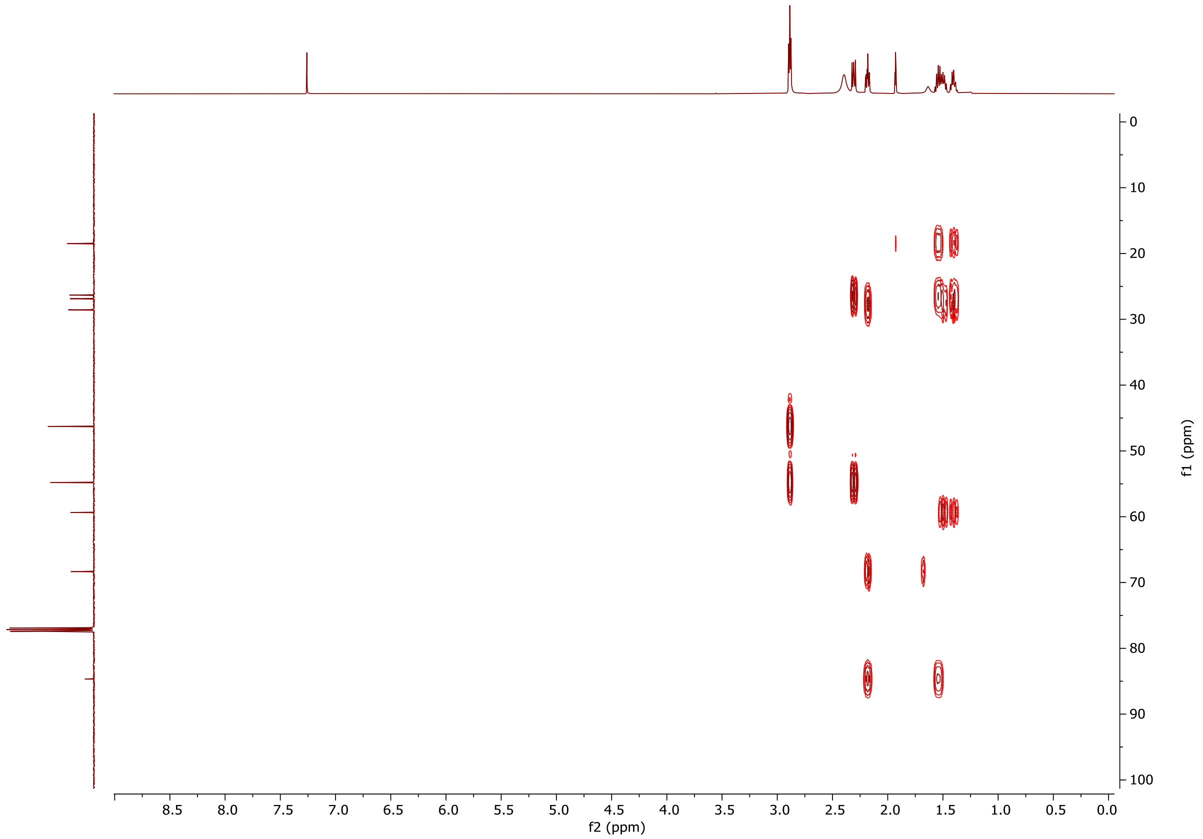

Supplementary spectrum 35: HMBC-NMR spectrum of **7**

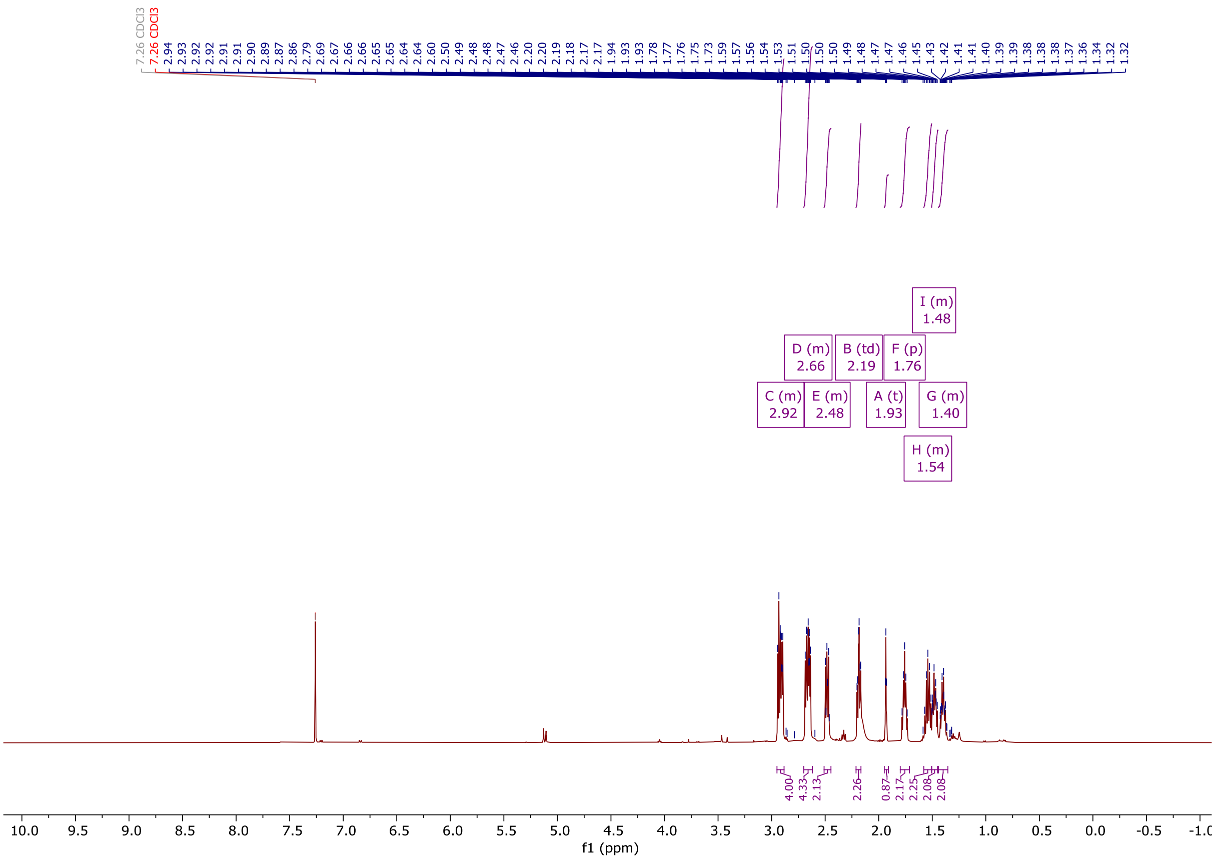

Supplementary spectrum 36: ^{1}H-NMR spectrum of **9**

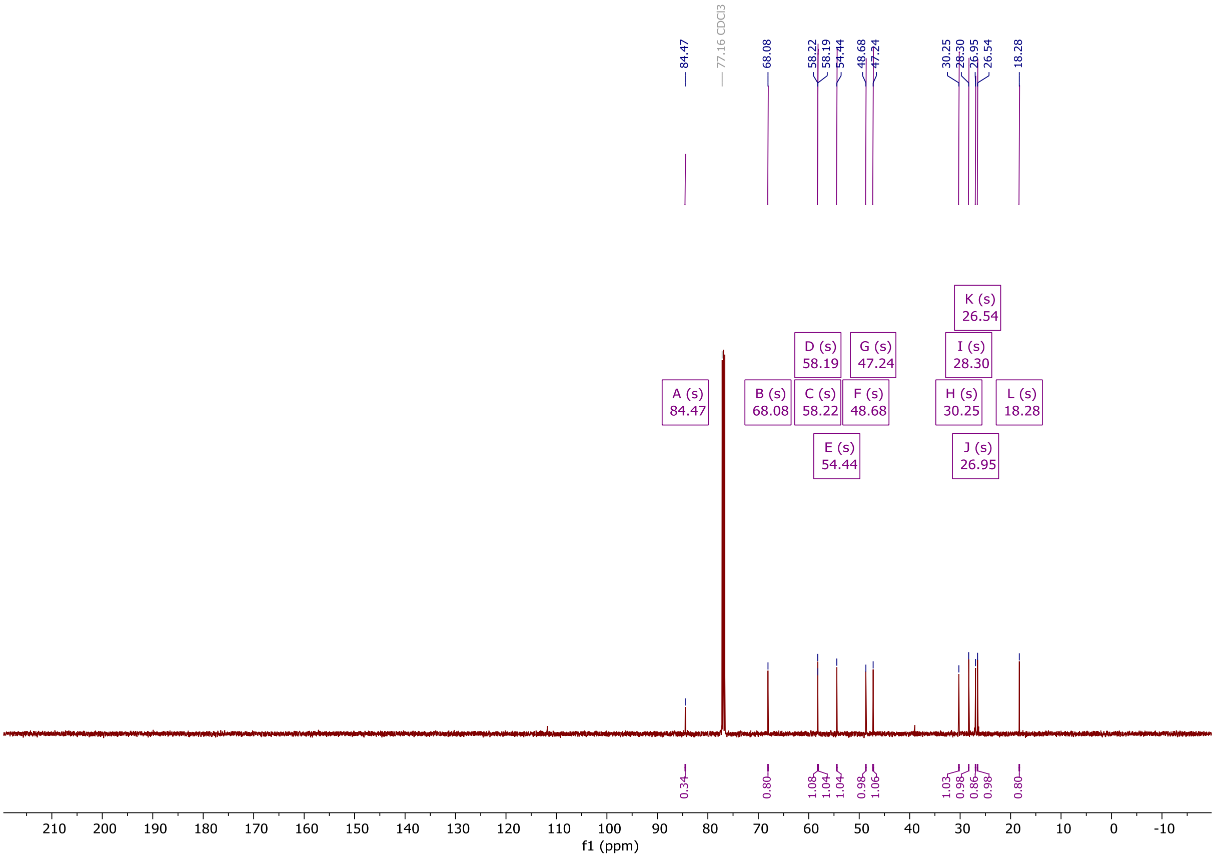

Supplementary spectrum 37: ^{13}C-NMR spectrum of **9**

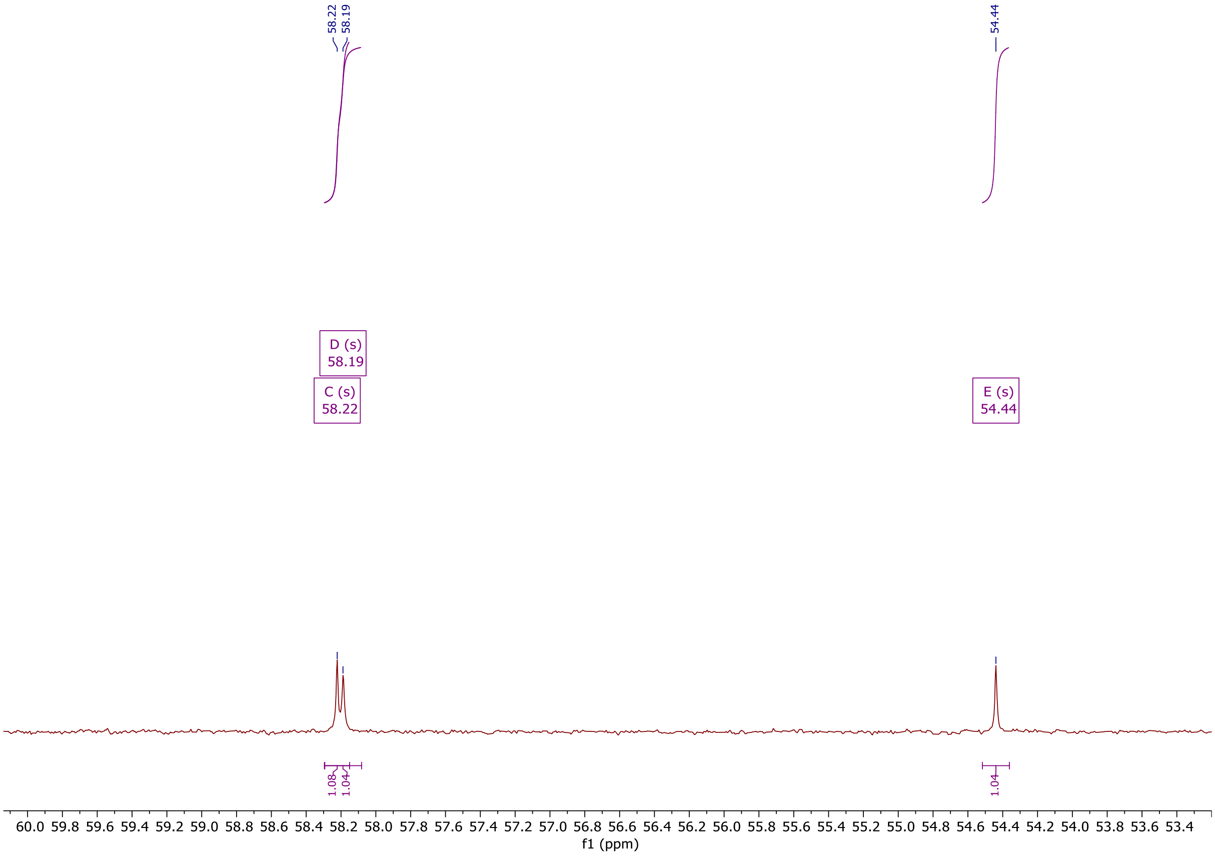

Supplementary spectrum 38: Zoomed in 13C-NMR spectrum of **9**

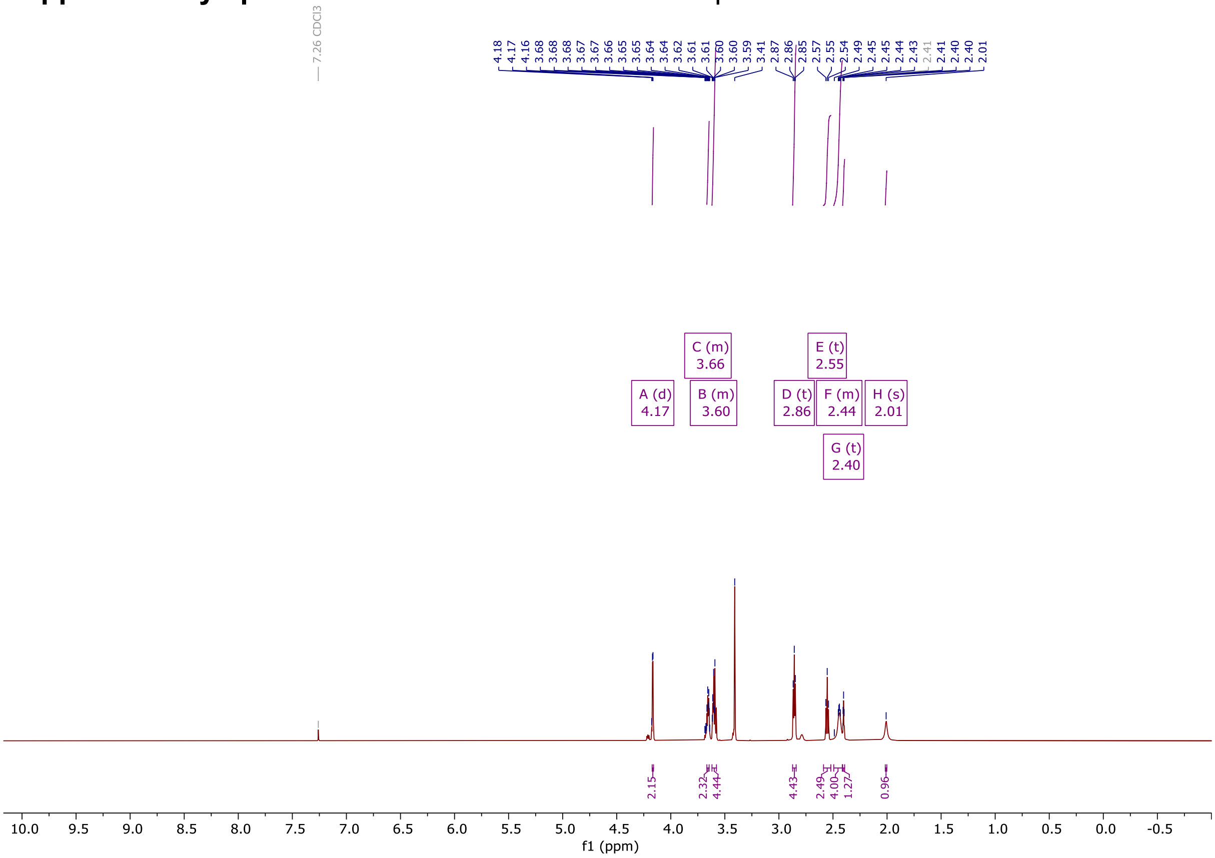

Supplementary spectrum 39: ^{1}H-NMR spectrum of **10**

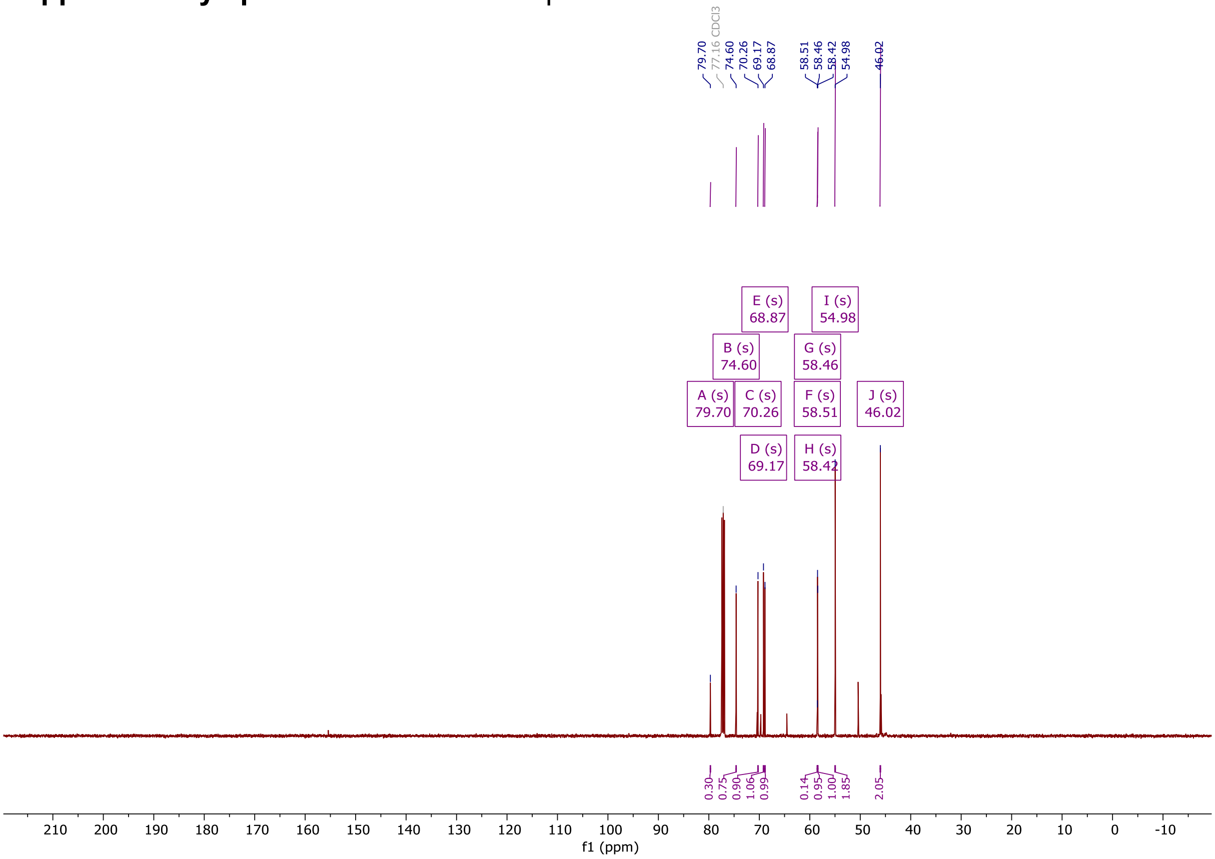

Supplementary spectrum 40: ^{13}C-NMR spectrum of **10**

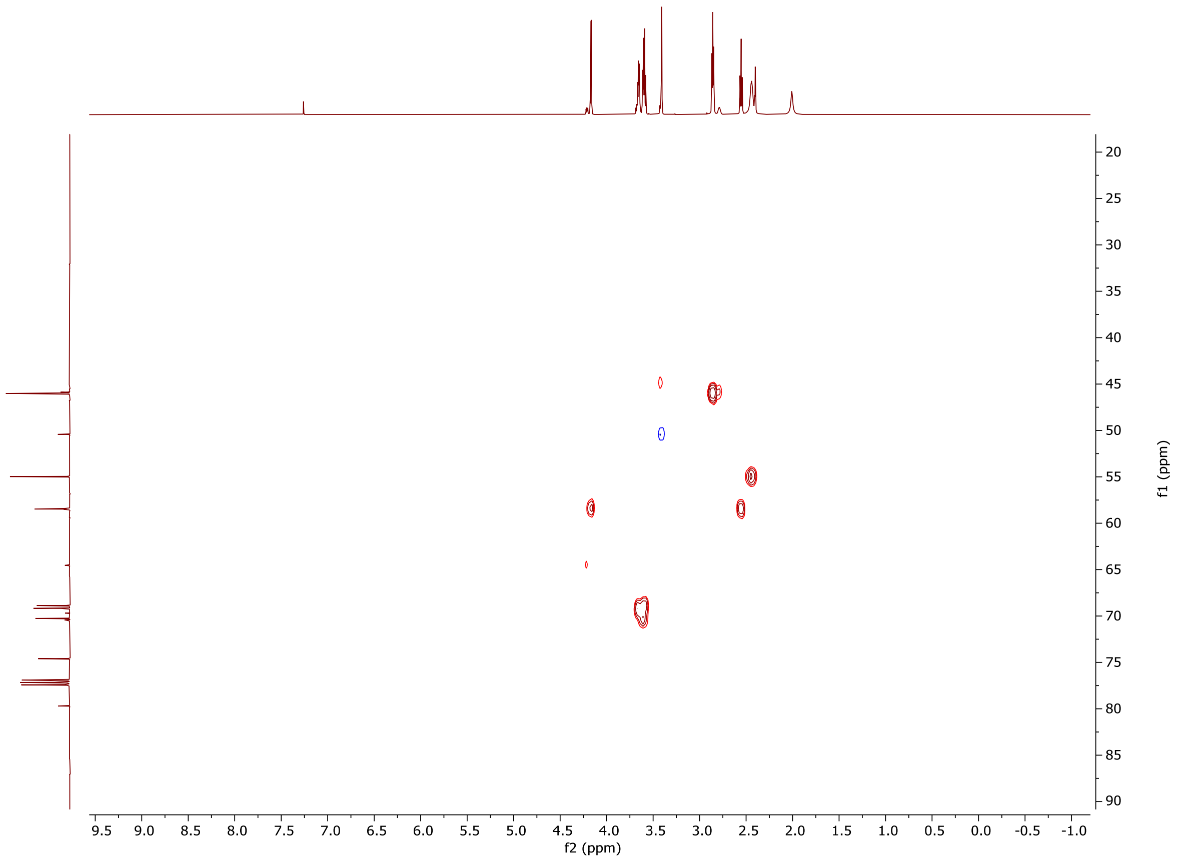

Supplementary spectrum 41: HSQC-NMR spectrum of **10**

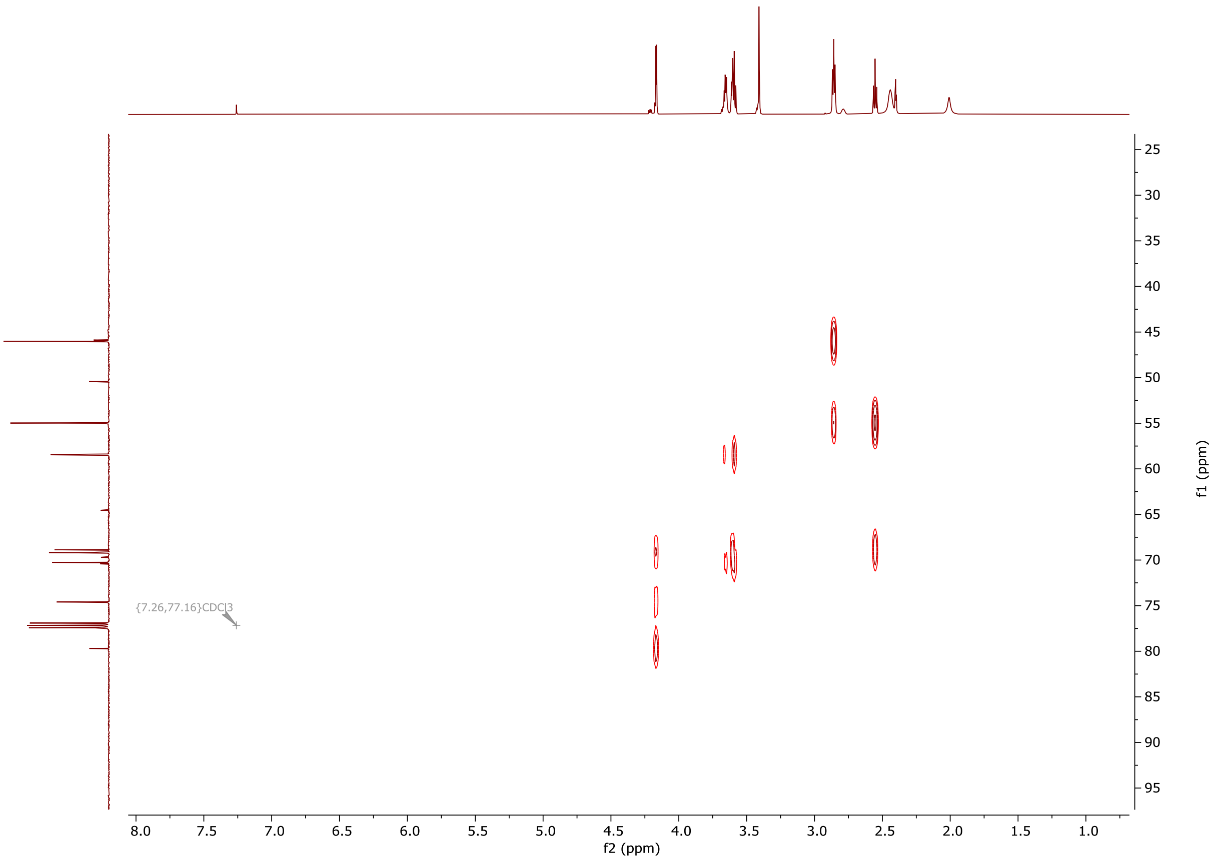

Supplementary spectrum 42: HMBC-NMR spectrum of **10**

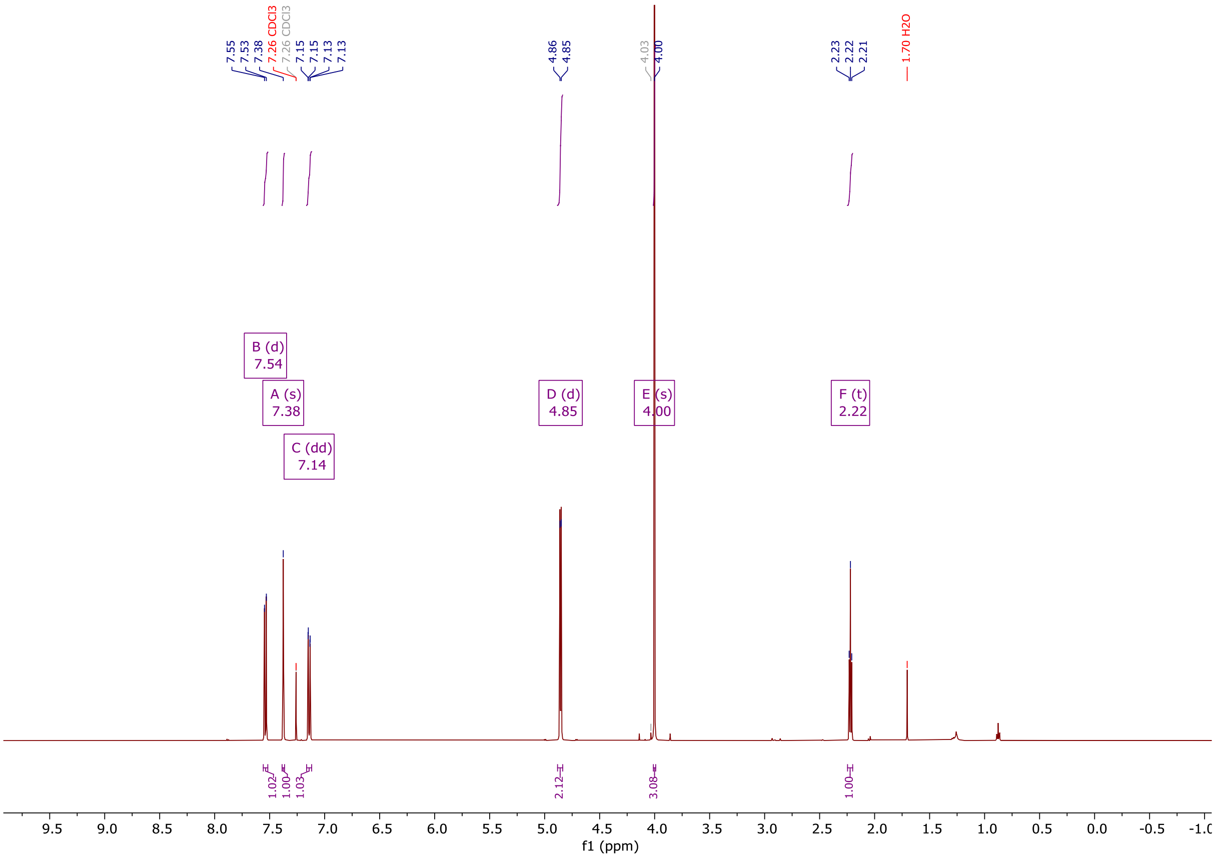

Supplementary spectrum 43: ^{1}H-NMR spectrum of **45**

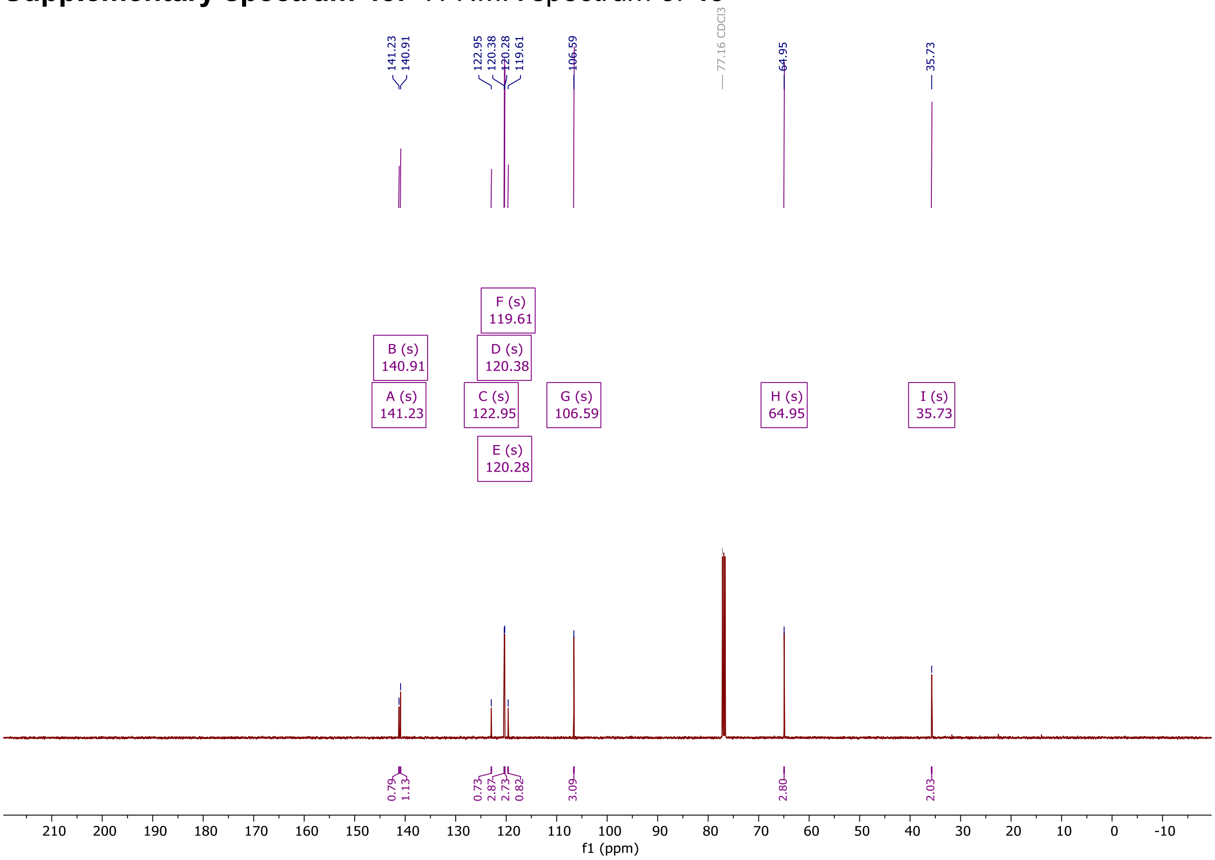

Supplementary spectrum 44: ^{13}C-NMR spectrum of **45**

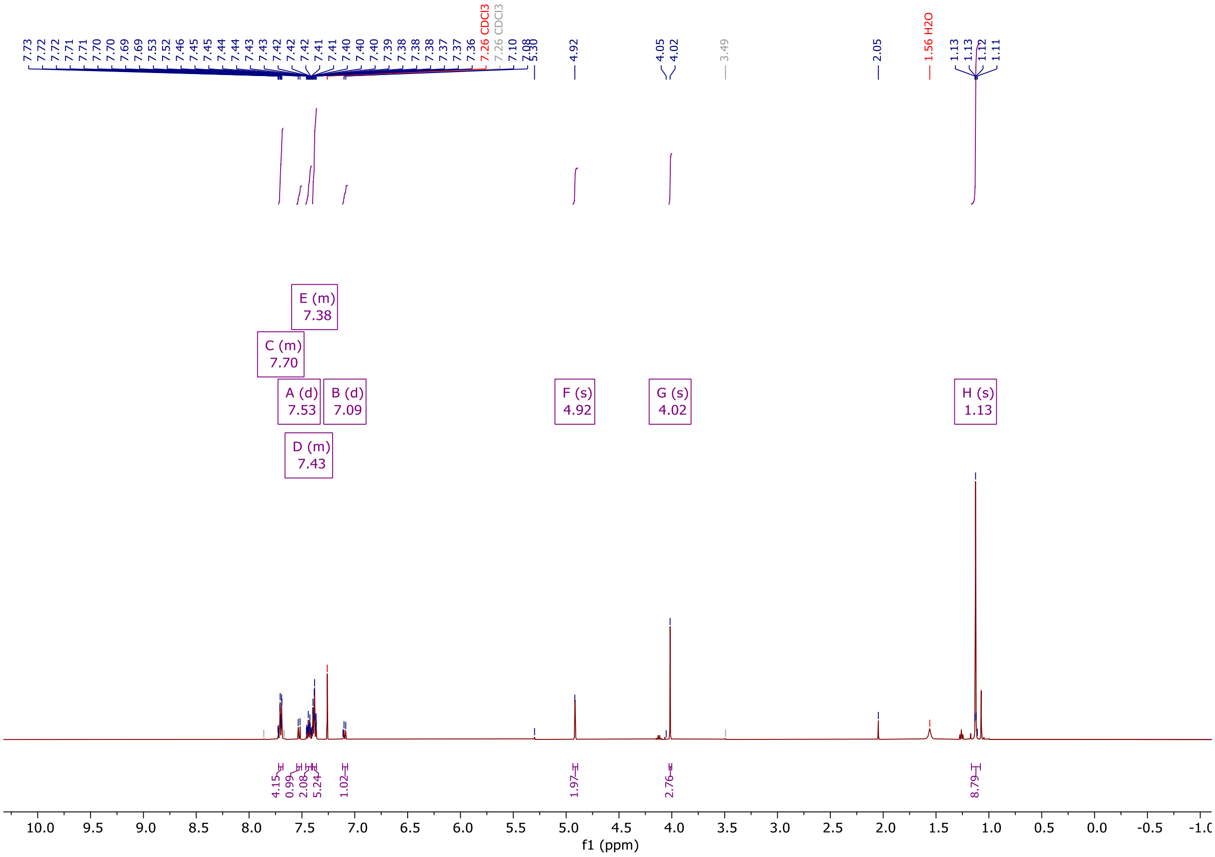

Supplementary spectrum 45: ^{1}H-NMR spectrum of **46**

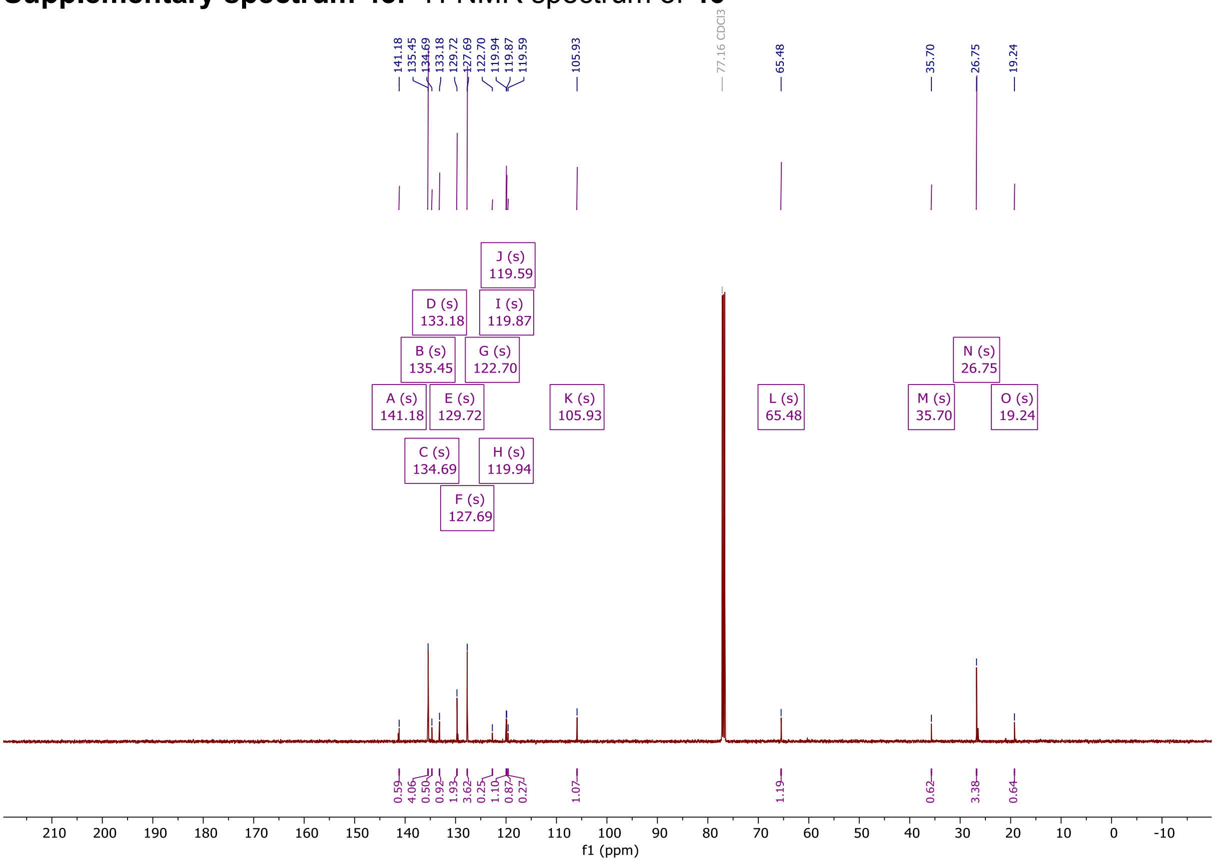

Supplementary spectrum 46: ^{13}C-NMR spectrum of **46**

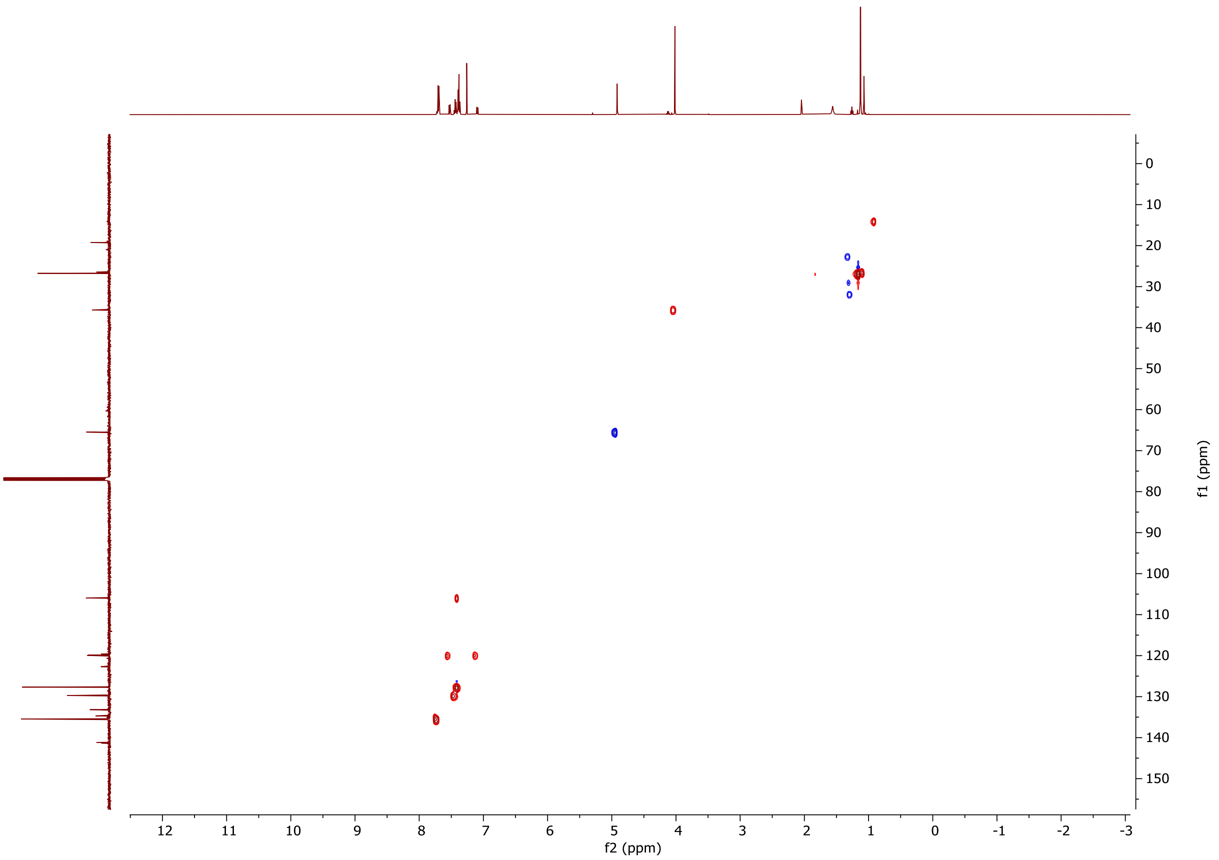

Supplementary spectrum 47: HSQC-NMR spectrum of **46**

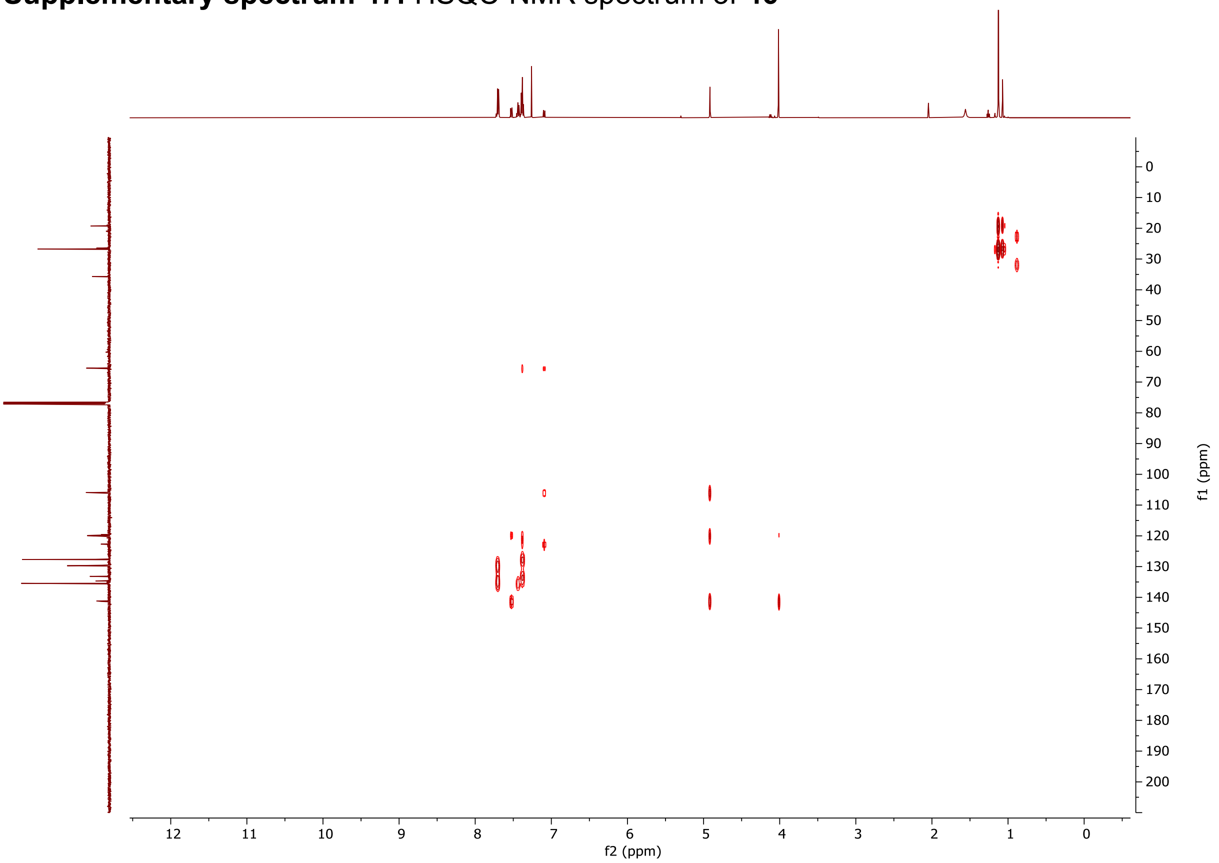

Supplementary spectrum 48: HMBC-NMR spectrum of **46**

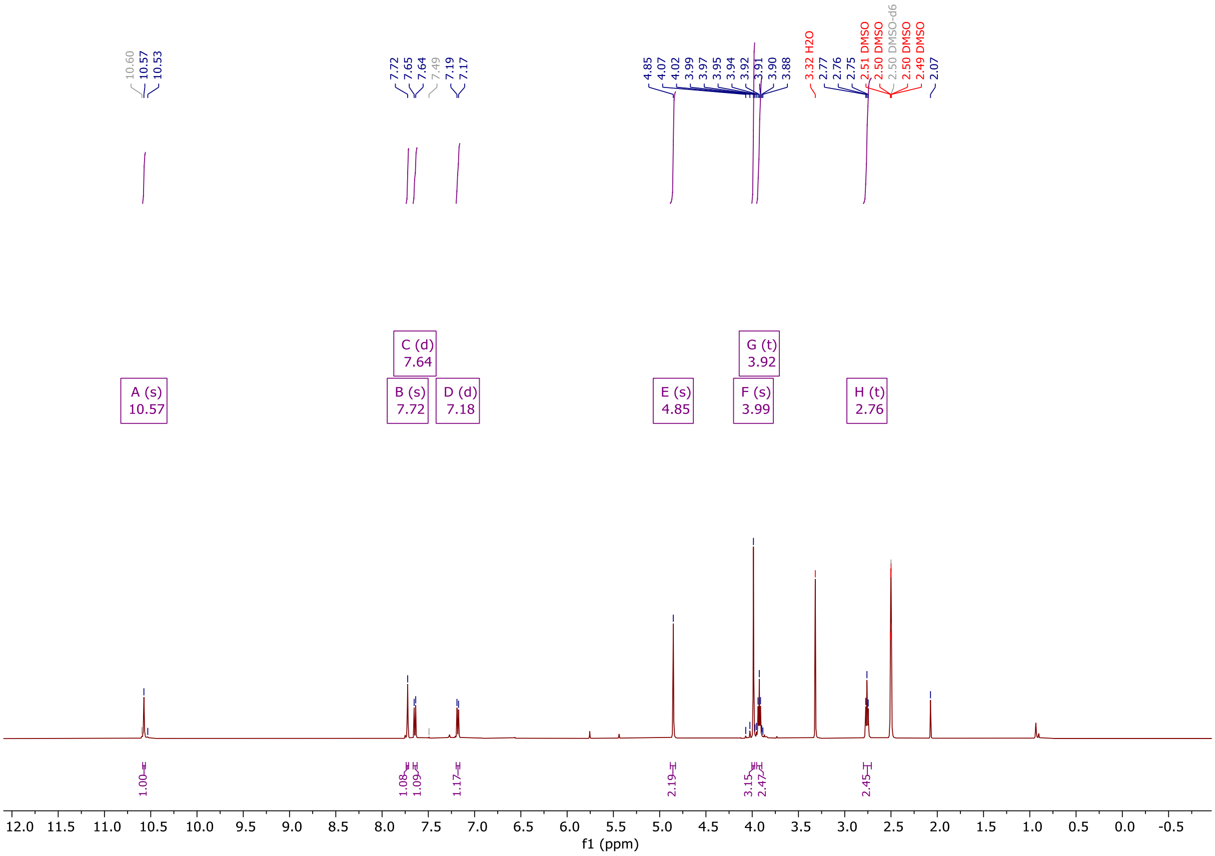

Supplementary spectrum 49: ^{1}H-NMR spectrum of **16**

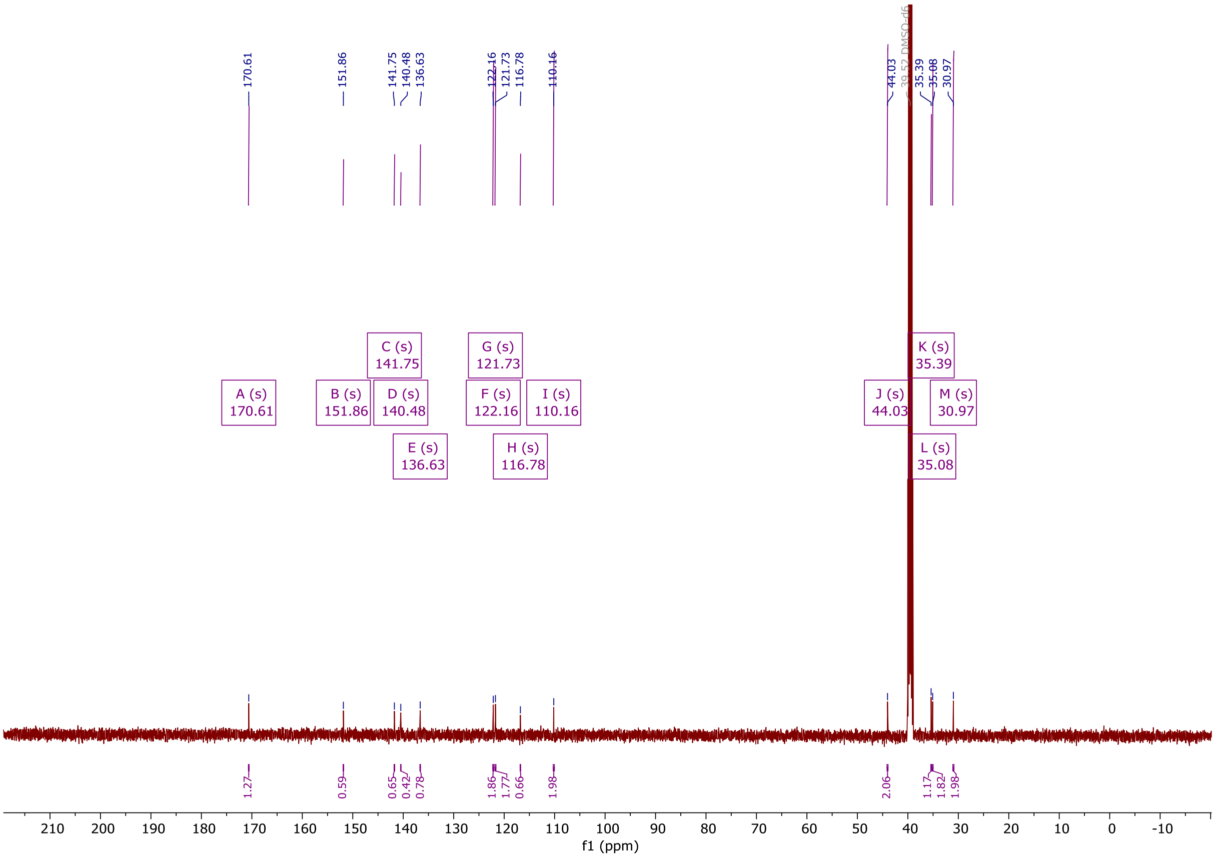

Supplementary spectrum 50: ^{13}C-NMR spectrum of **16**

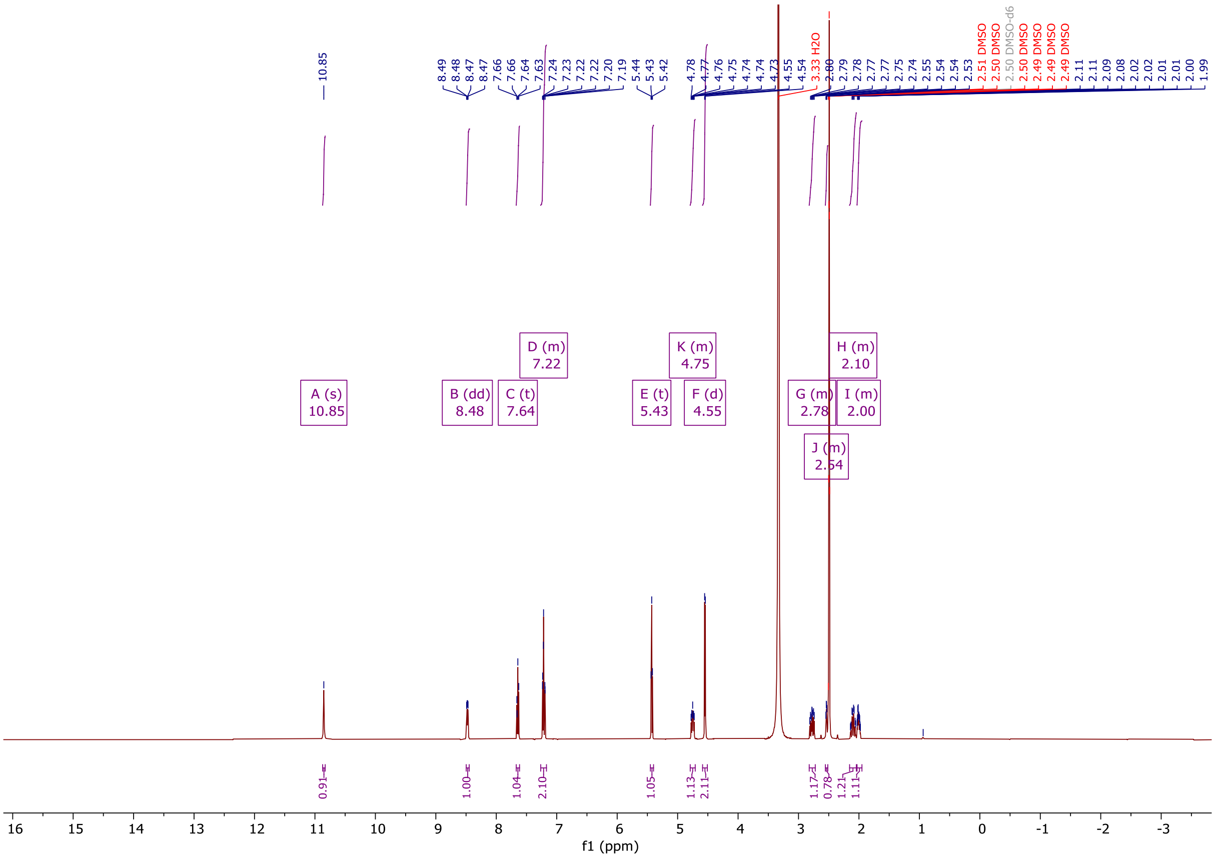

Supplementary spectrum 51: ^{1}H-NMR spectrum of **43**

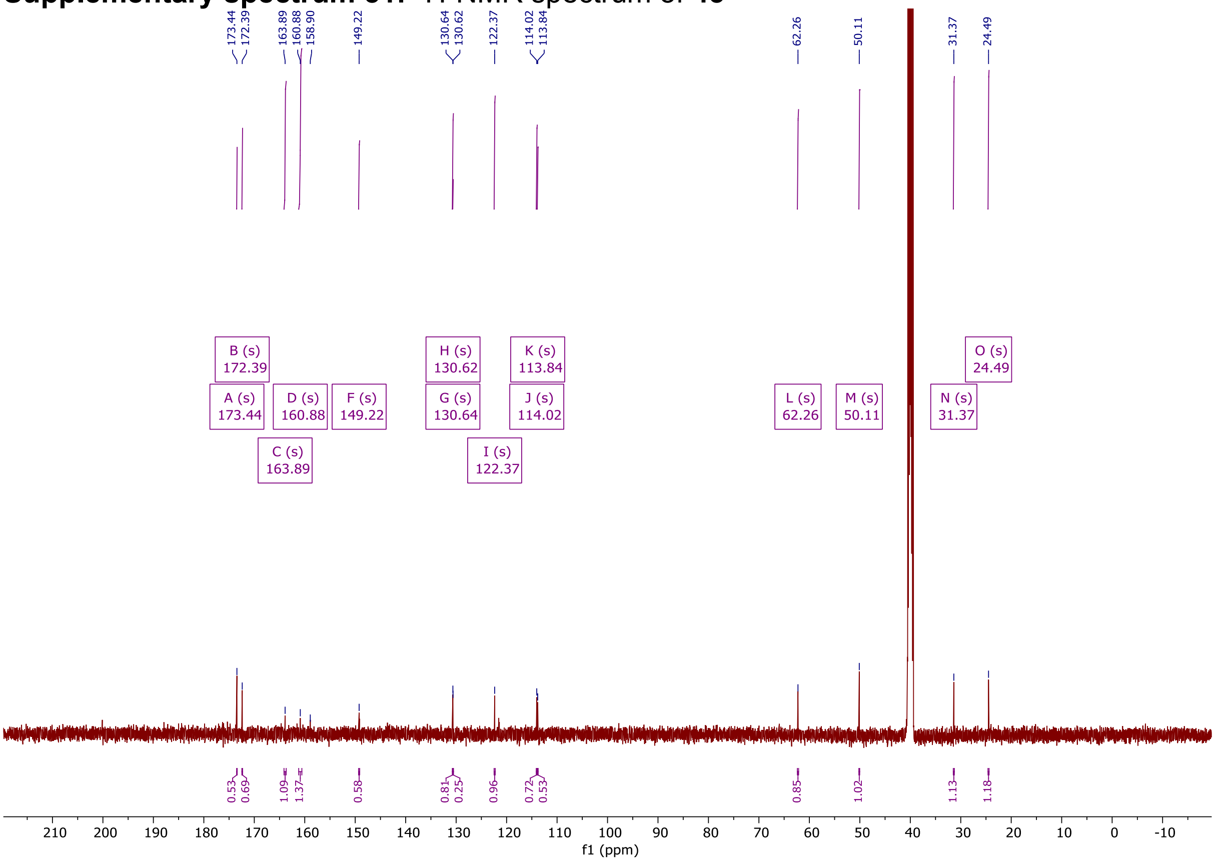

Supplementary spectrum 52: ^{13}C-NMR spectrum of **43**

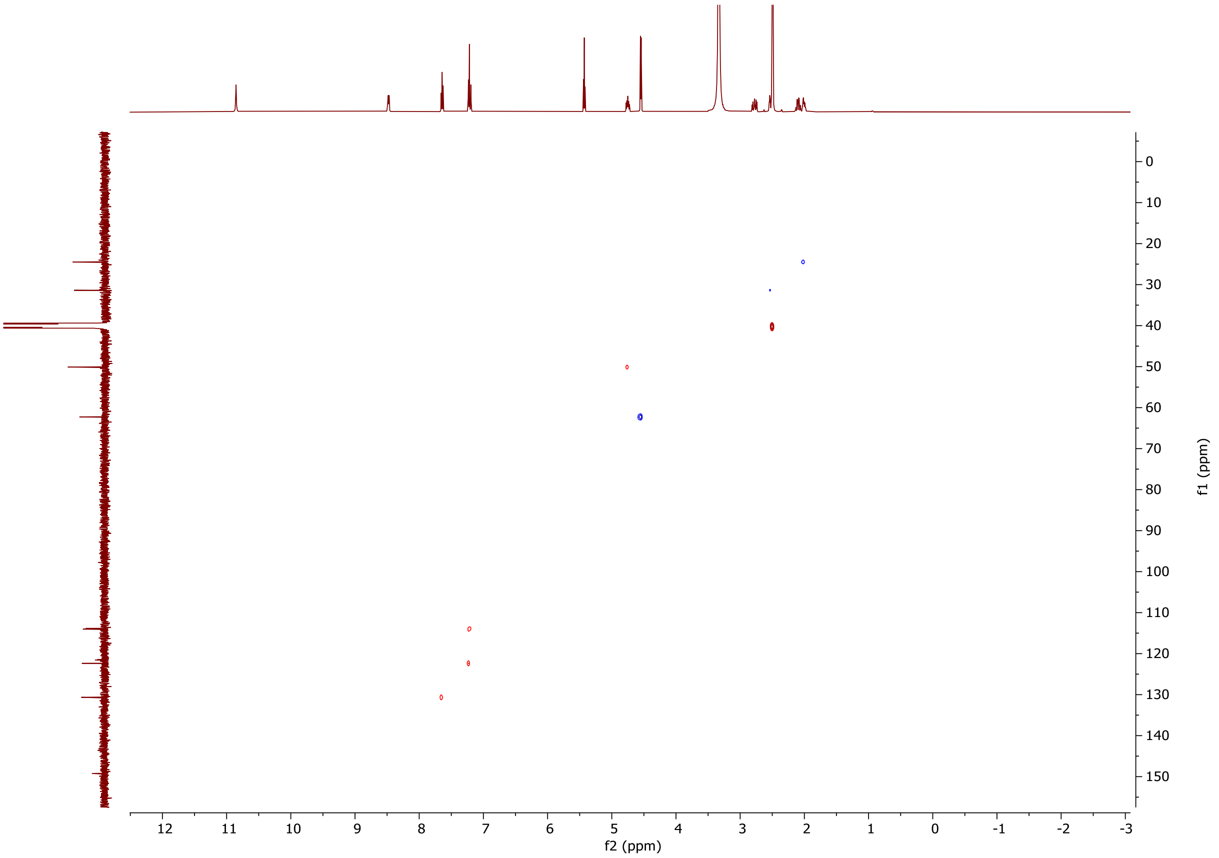

Supplementary spectrum 53: HSQC-NMR spectrum of **43**

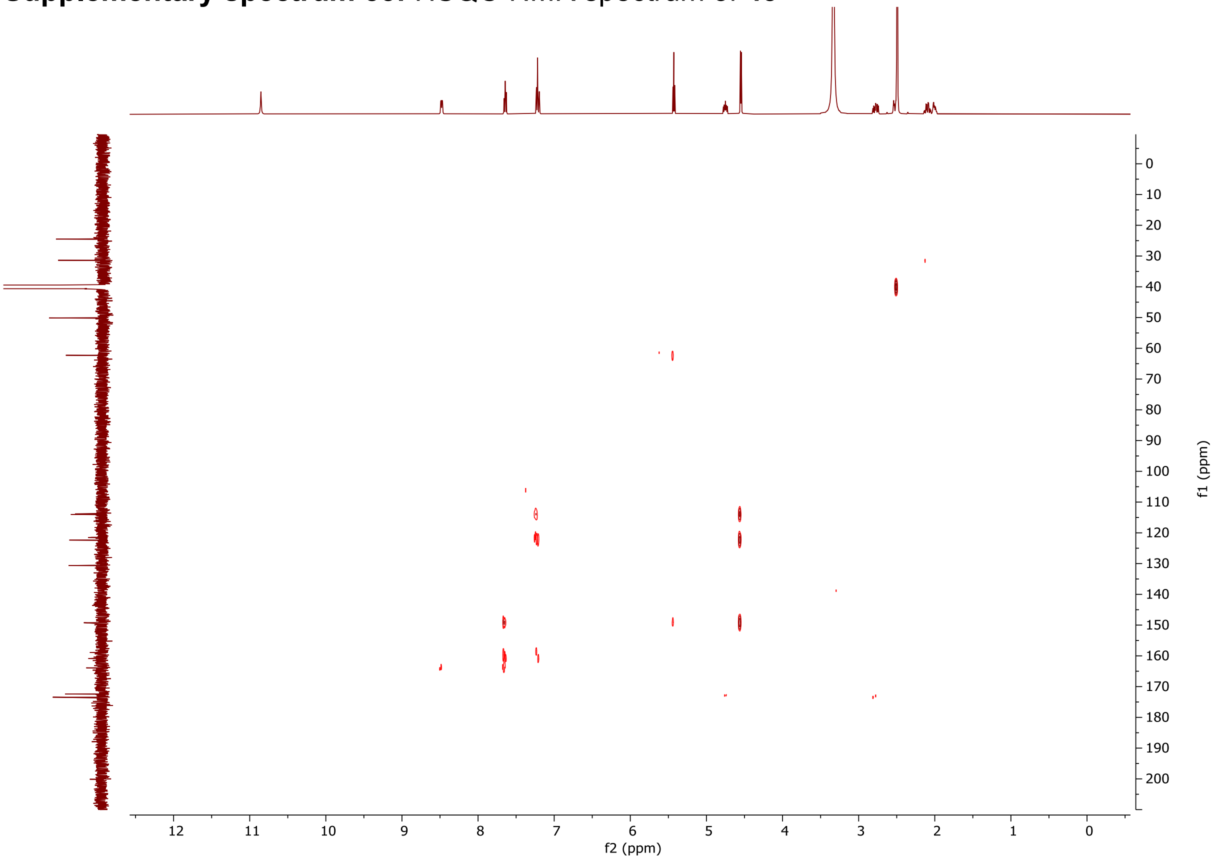

Supplementary spectrum 54: HMBC-NMR spectrum of **43**

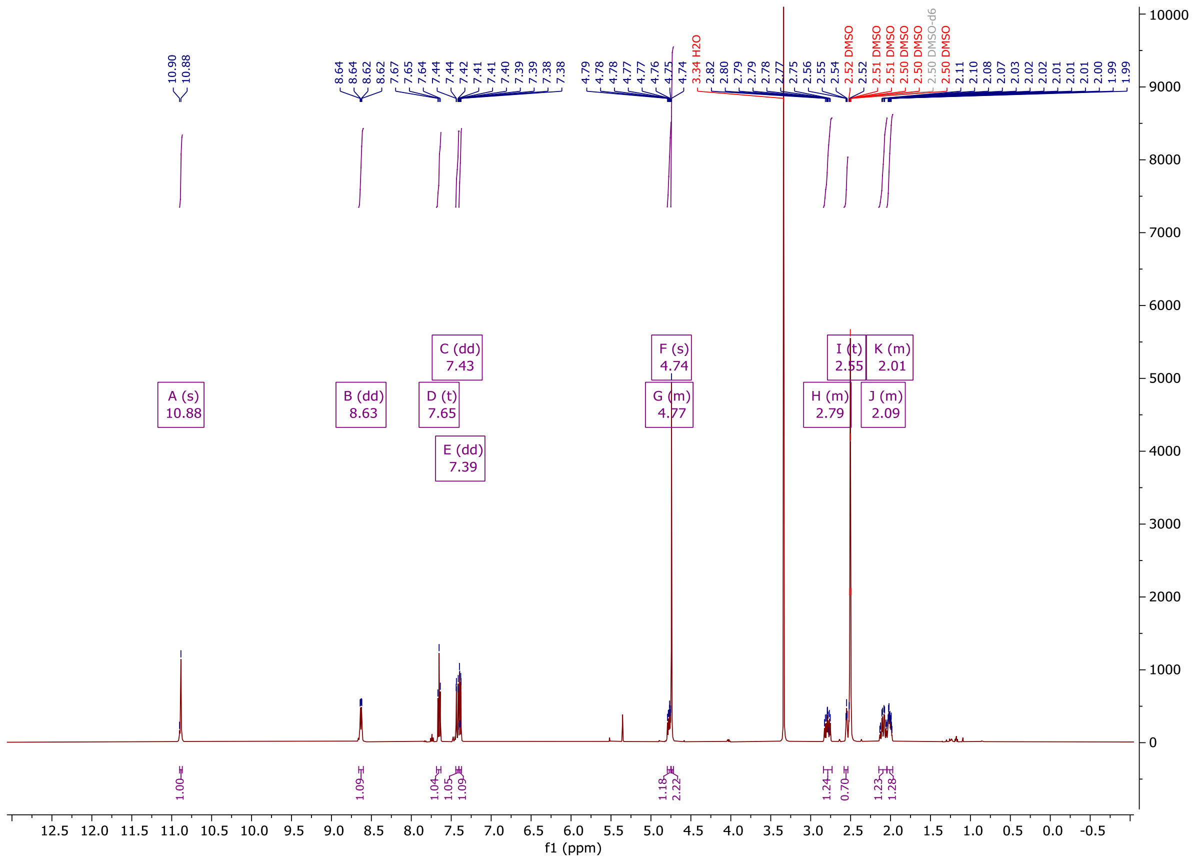

Supplementary spectrum 55: ^{1}H-NMR spectrum of **15**

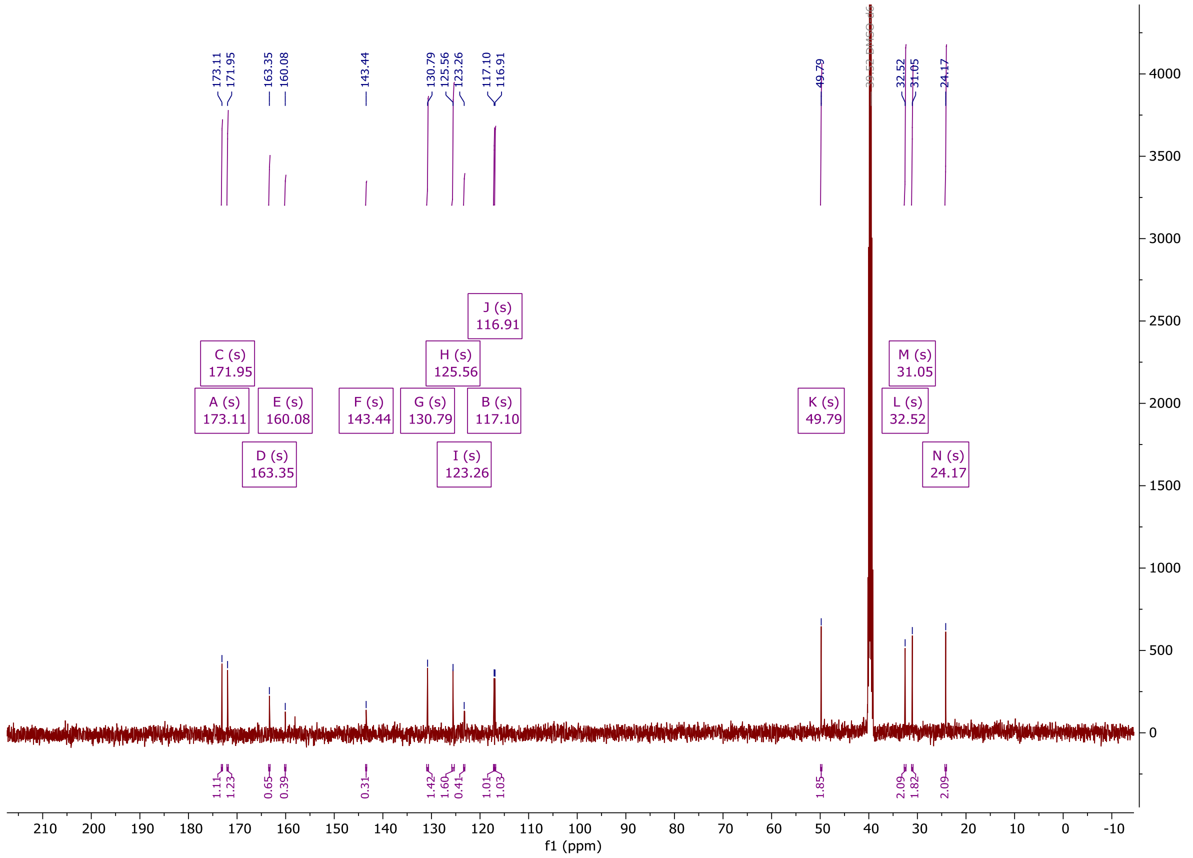

Supplementary spectrum 56: ^{13}C-NMR spectrum of **15**

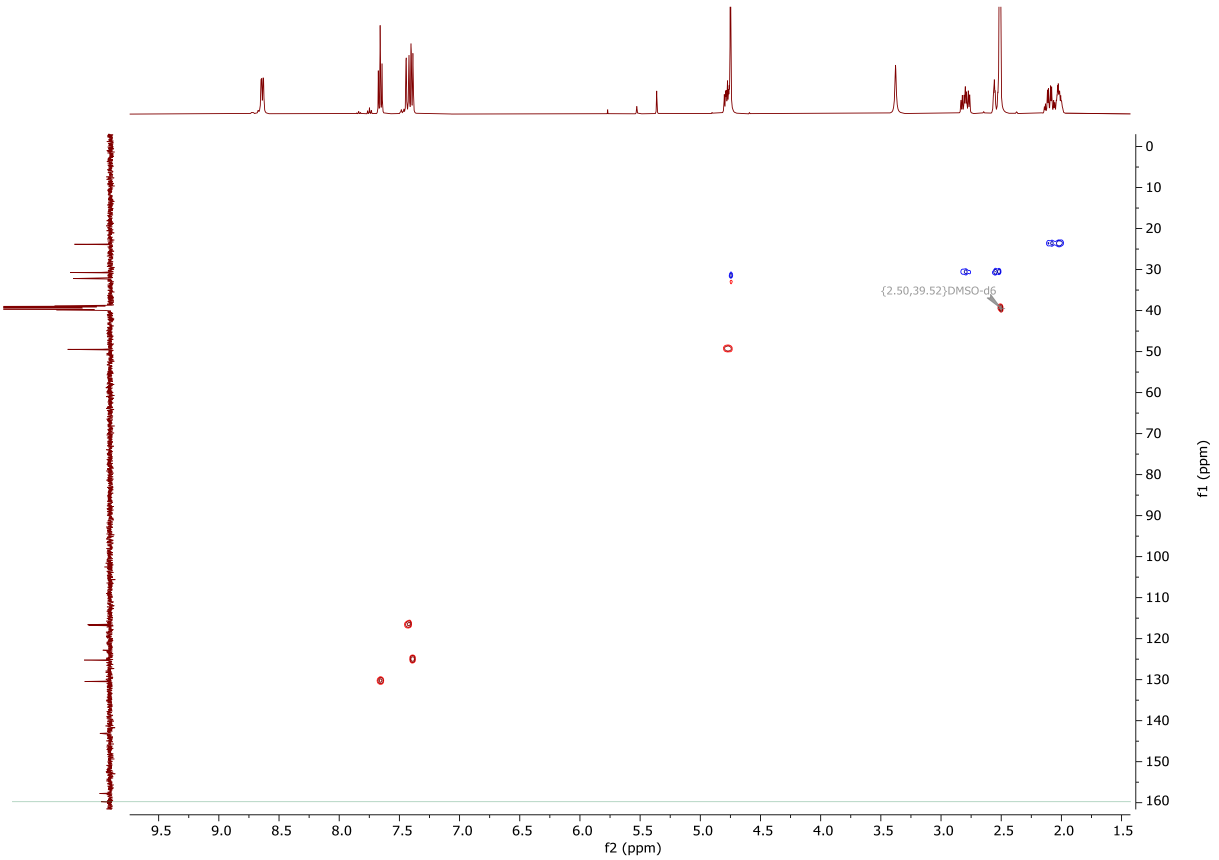

Supplementary spectrum 57: HSQC-NMR spectrum of **15**

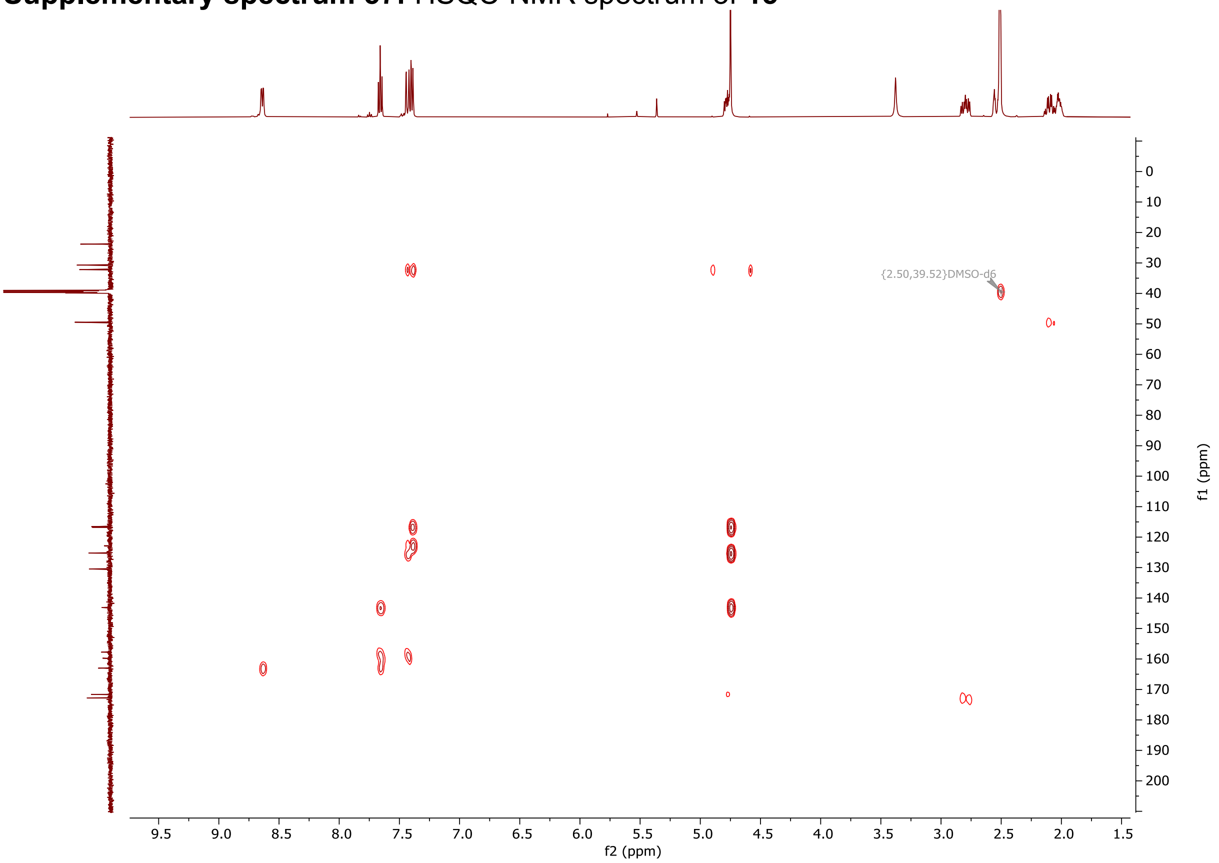

Supplementary spectrum 58: HMBC-NMR spectrum of **15**

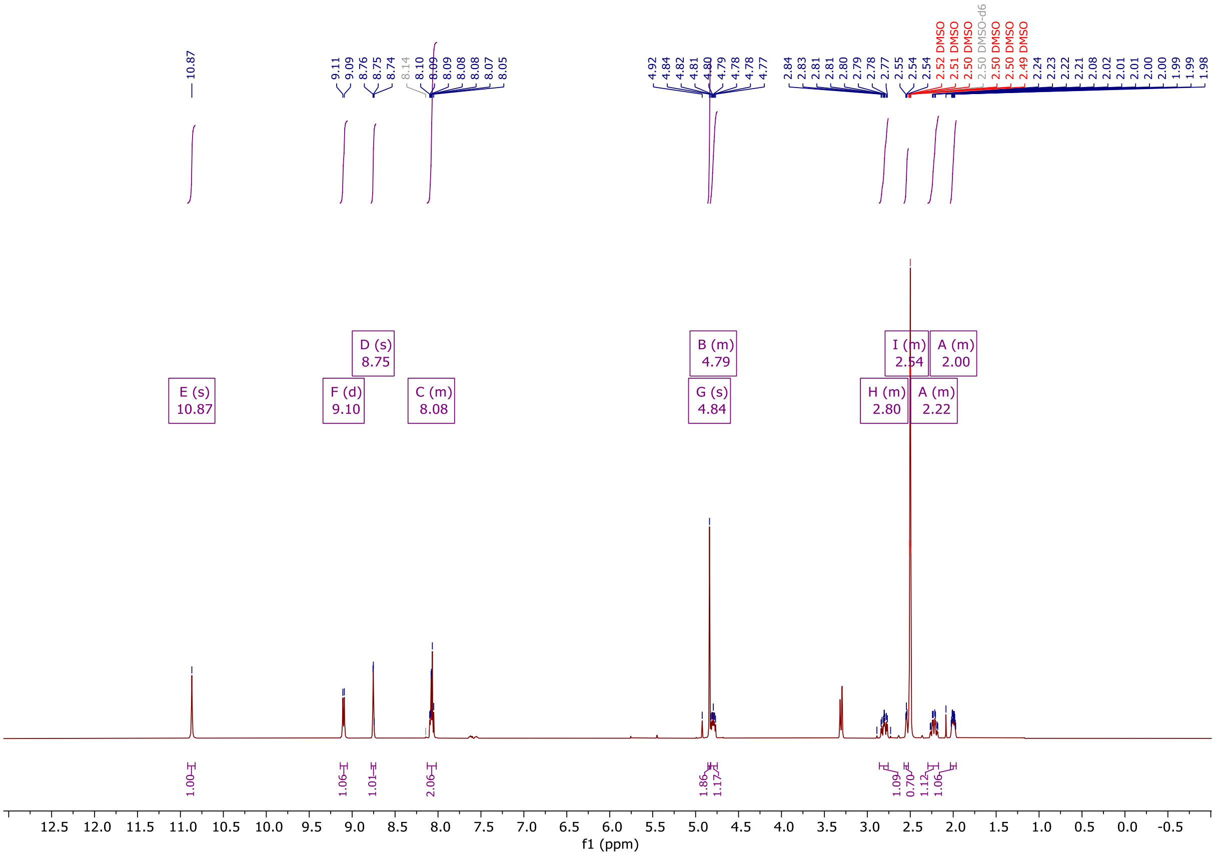

Supplementary spectrum 59: ^{1}H-NMR spectrum of **17**

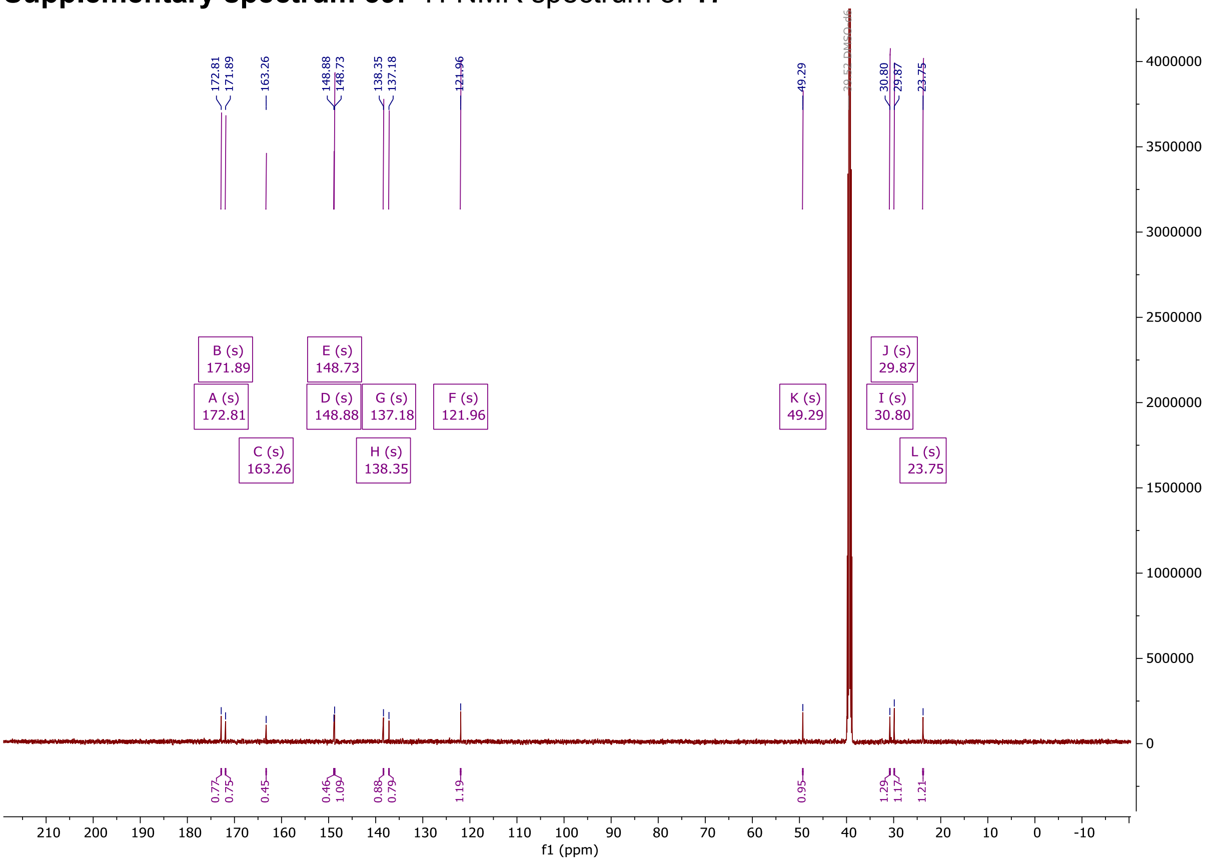

Supplementary spectrum 60: ^{13}C-NMR spectrum of **17**

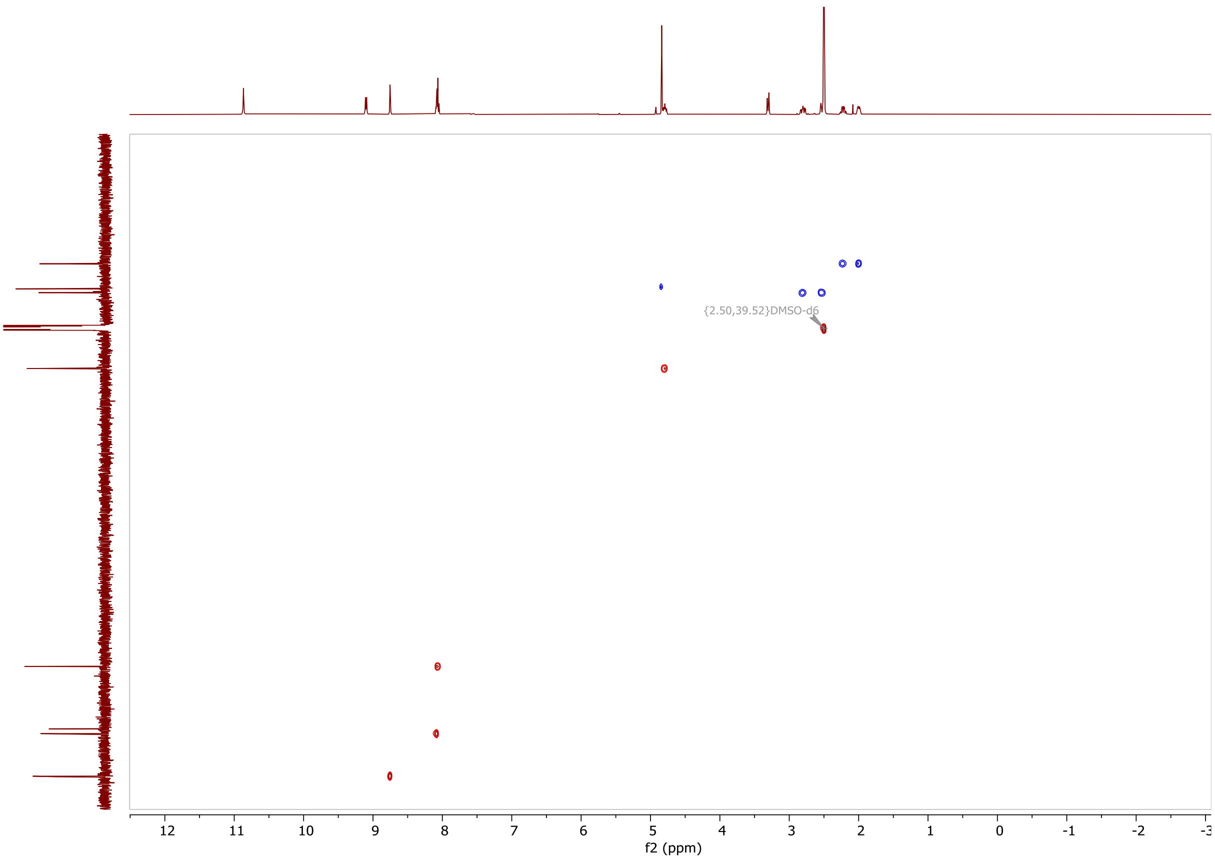

Supplementary spectrum 61: HSQC-NMR spectrum of **17**

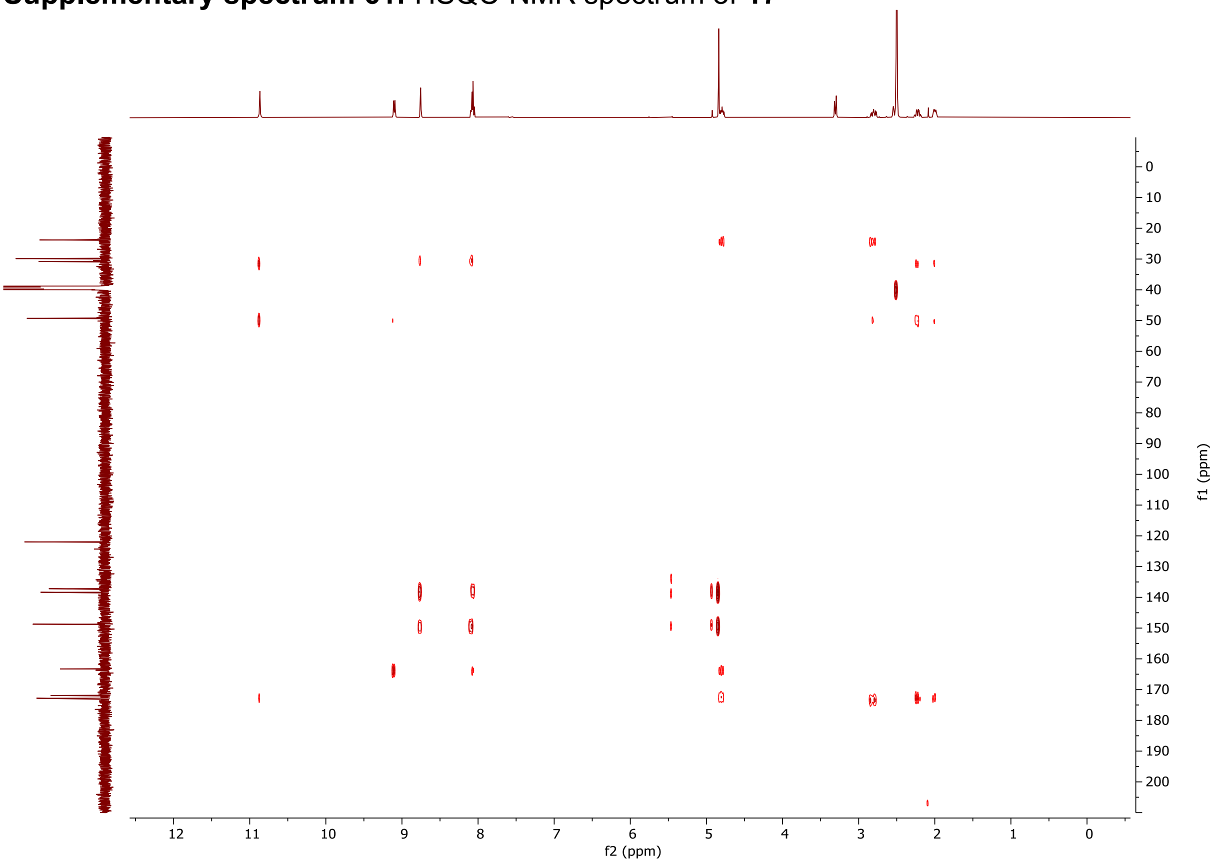

Supplementary spectrum 62: HMBC-NMR spectrum of **17**

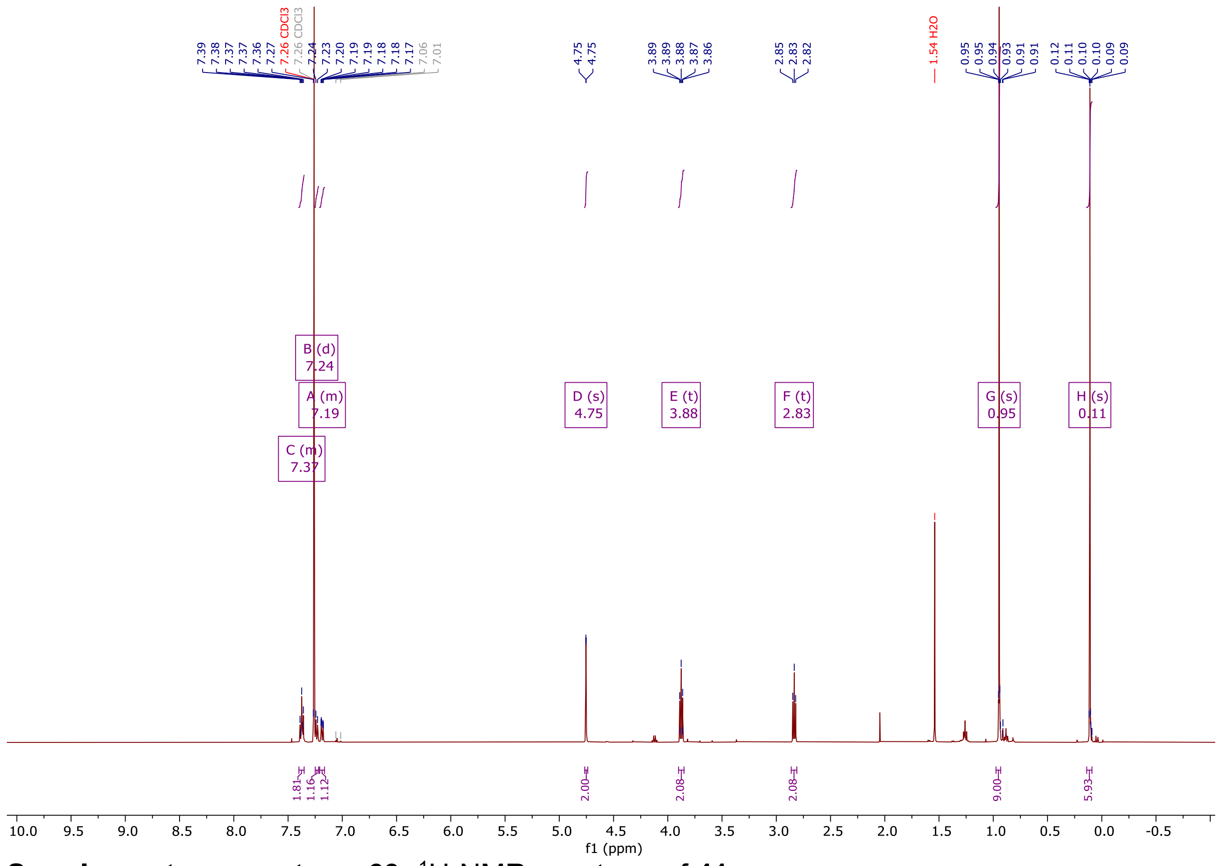

Supplementary spectrum 63: ^{1}H-NMR spectrum of **41**

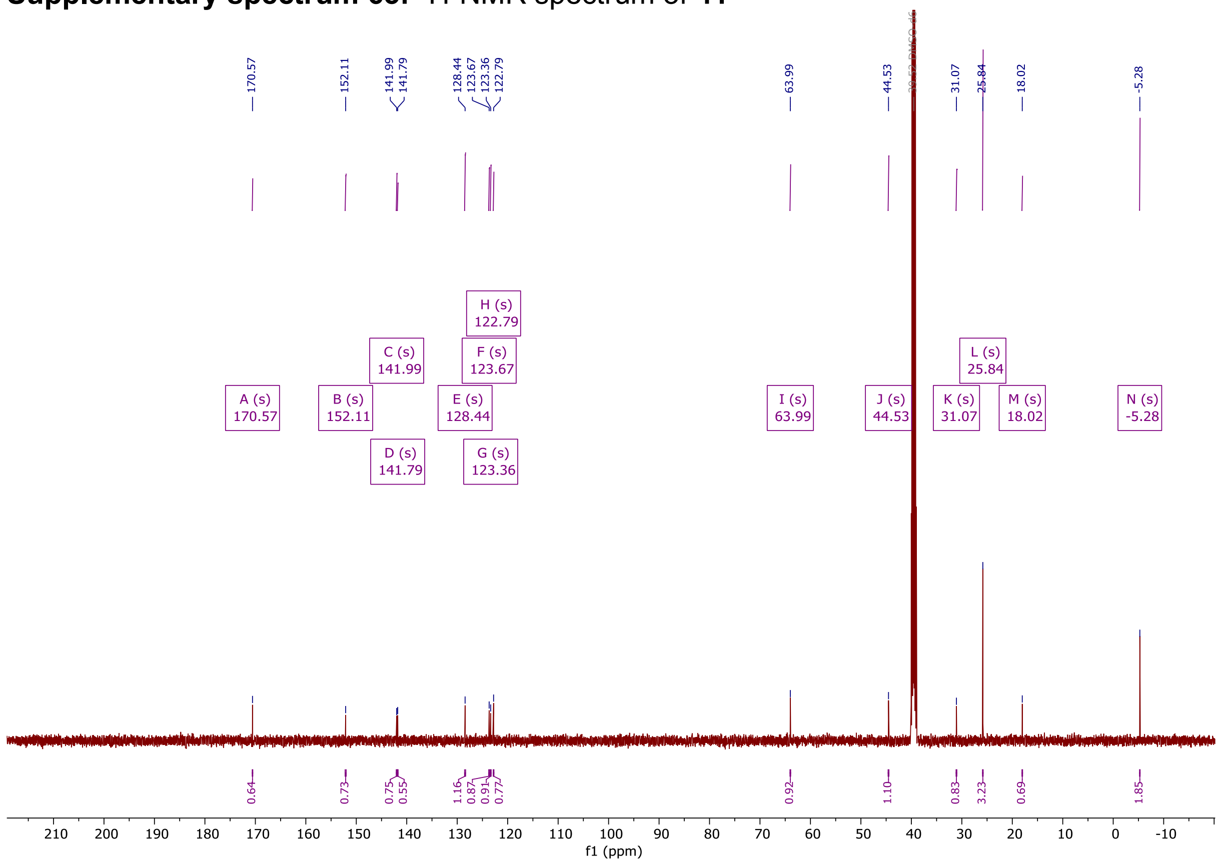

Supplementary spectrum 64: ^{13}C-NMR spectrum of **41**

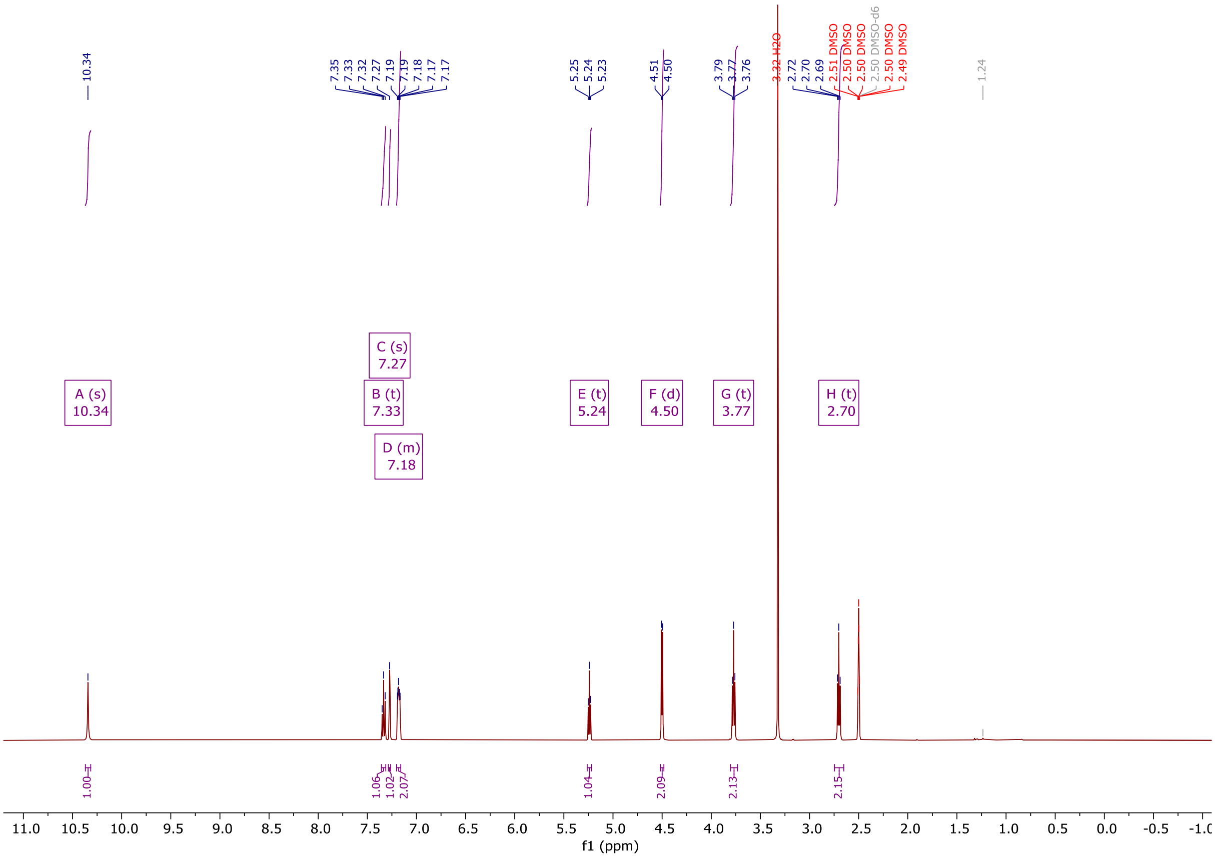

Supplementary spectrum 65: ^{1}H-NMR spectrum of **42**

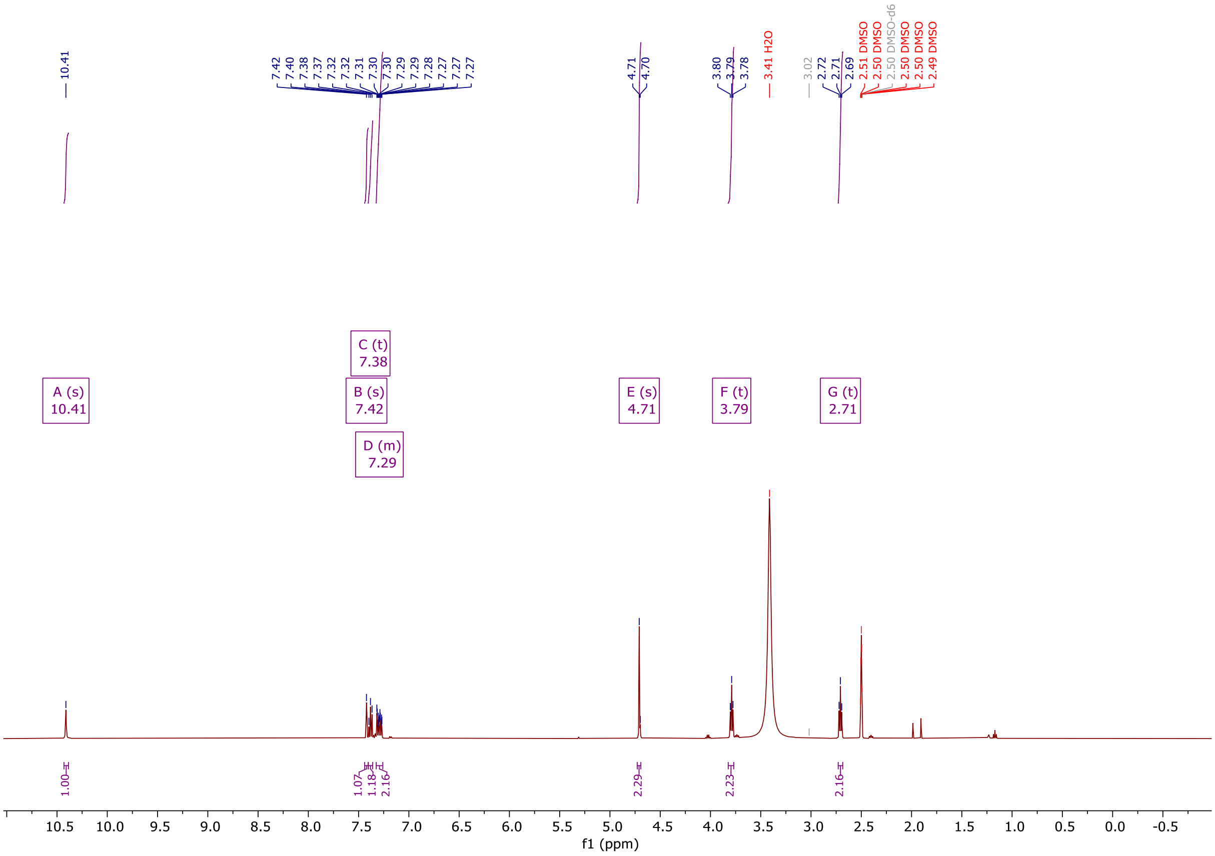

Supplementary spectrum 66: ^{1}H-NMR spectrum of **13**

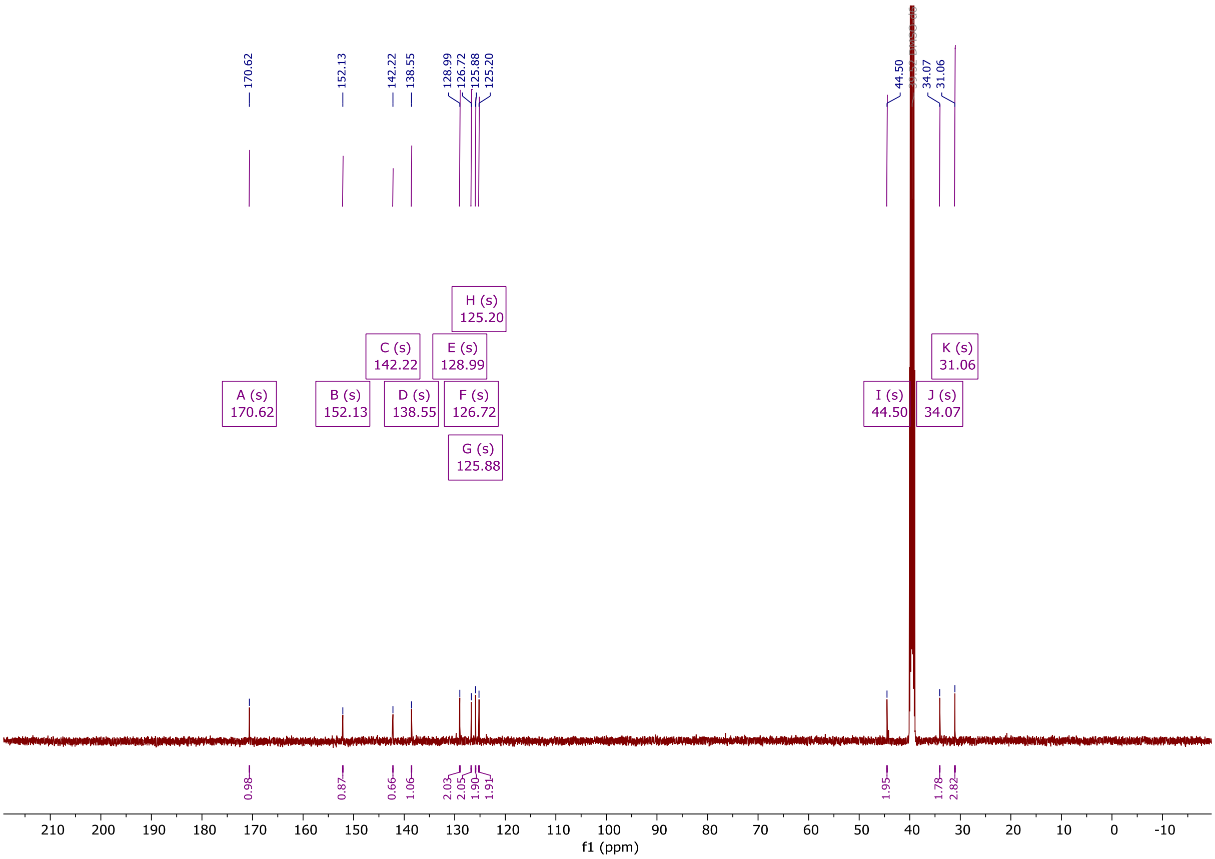

Supplementary spectrum 67: ^{13}C-NMR spectrum of **13**

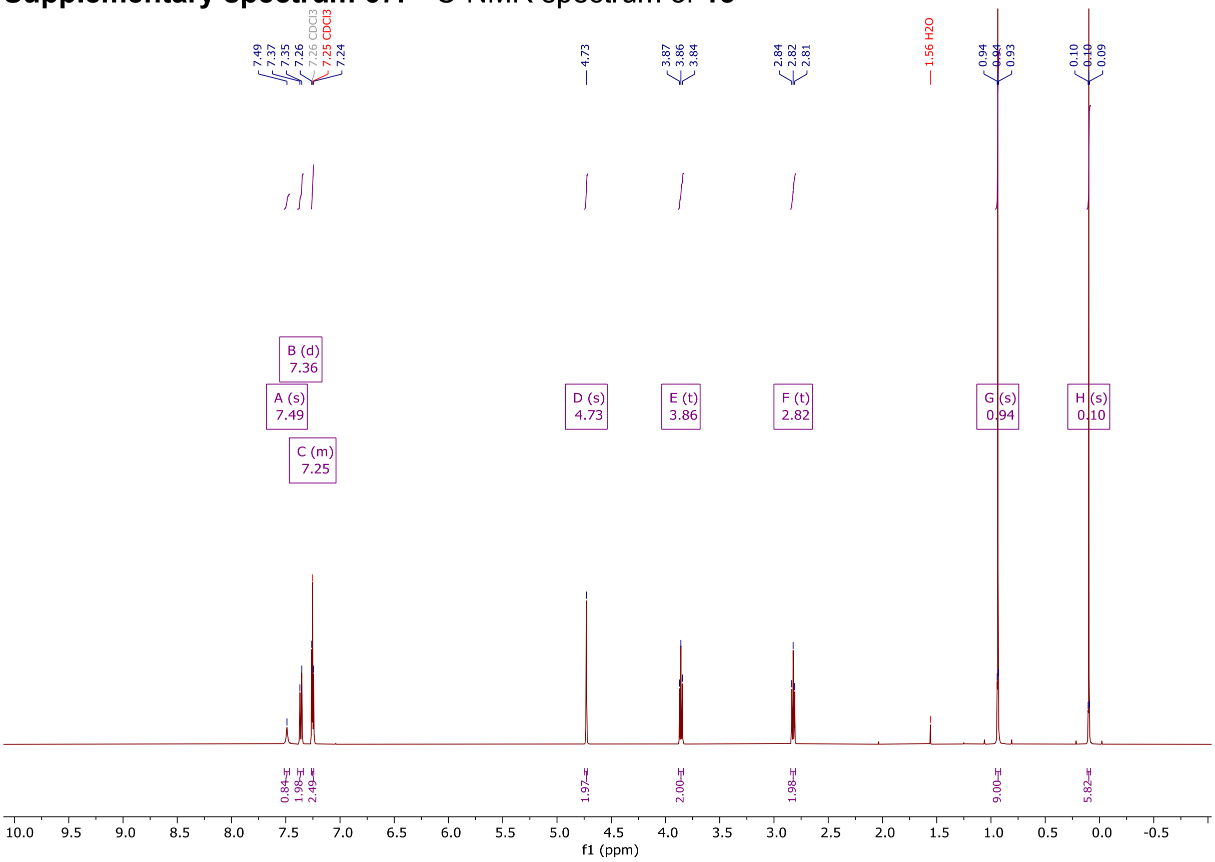

Supplementary spectrum 68: ^{1}H-NMR spectrum of **49**

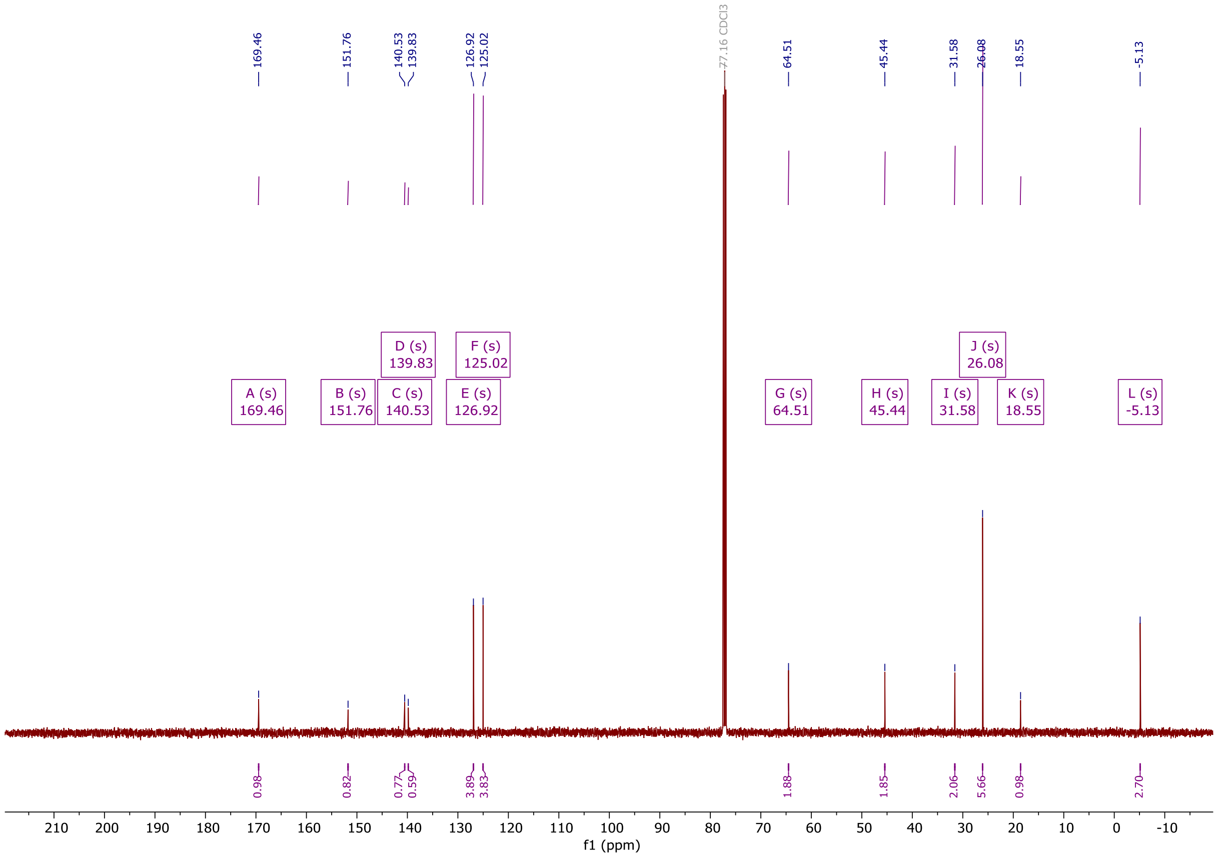

Supplementary spectrum 69: ^{13}C-NMR spectrum of **49**

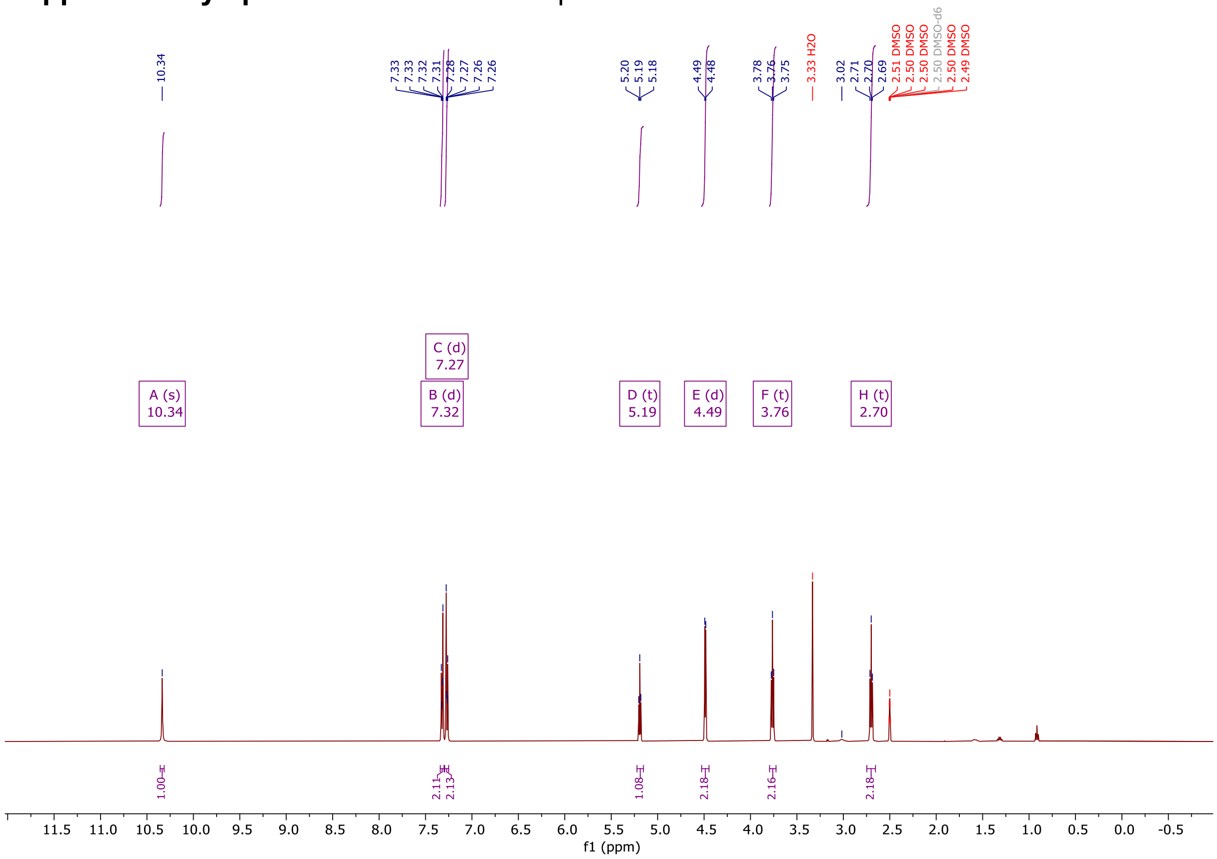

Supplementary spectrum 70: ^{1}H-NMR spectrum of **50**

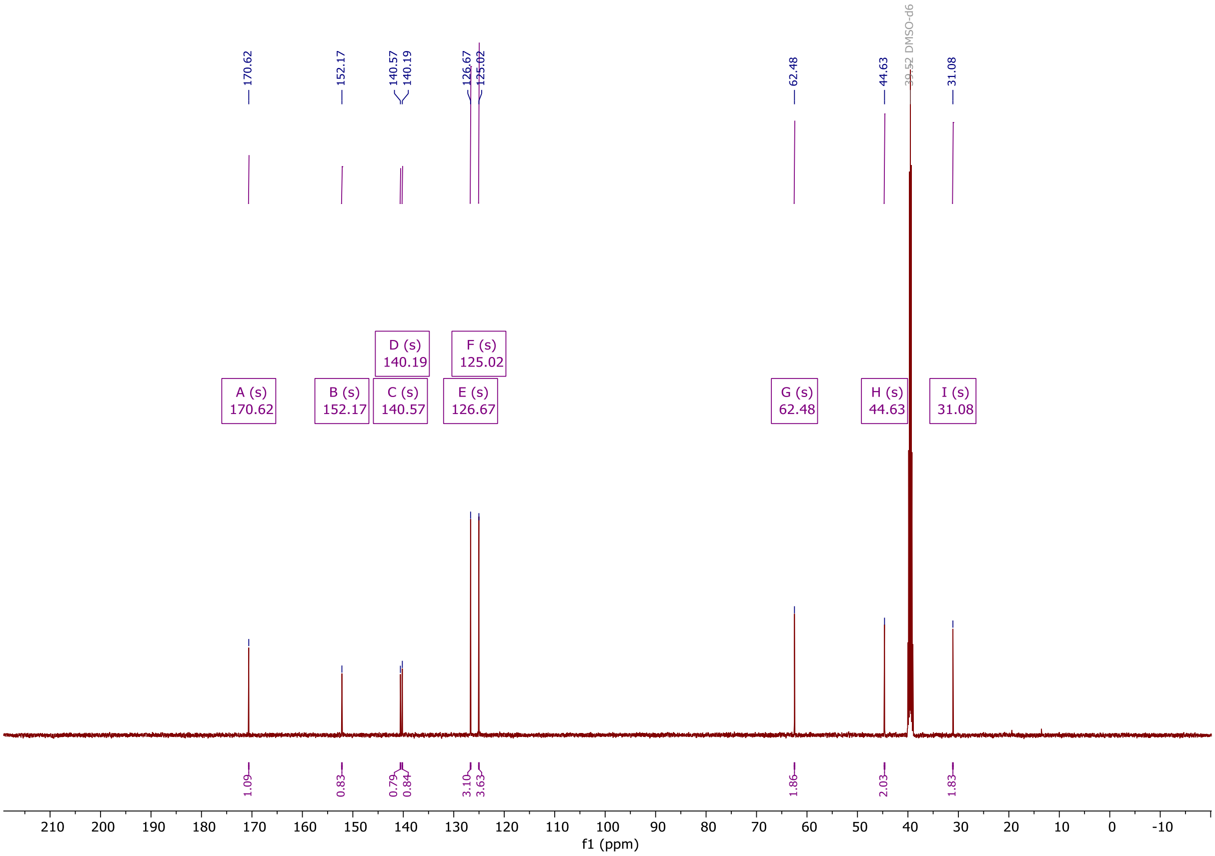

Supplementary spectrum 71: ^{13}C-NMR spectrum of **50**

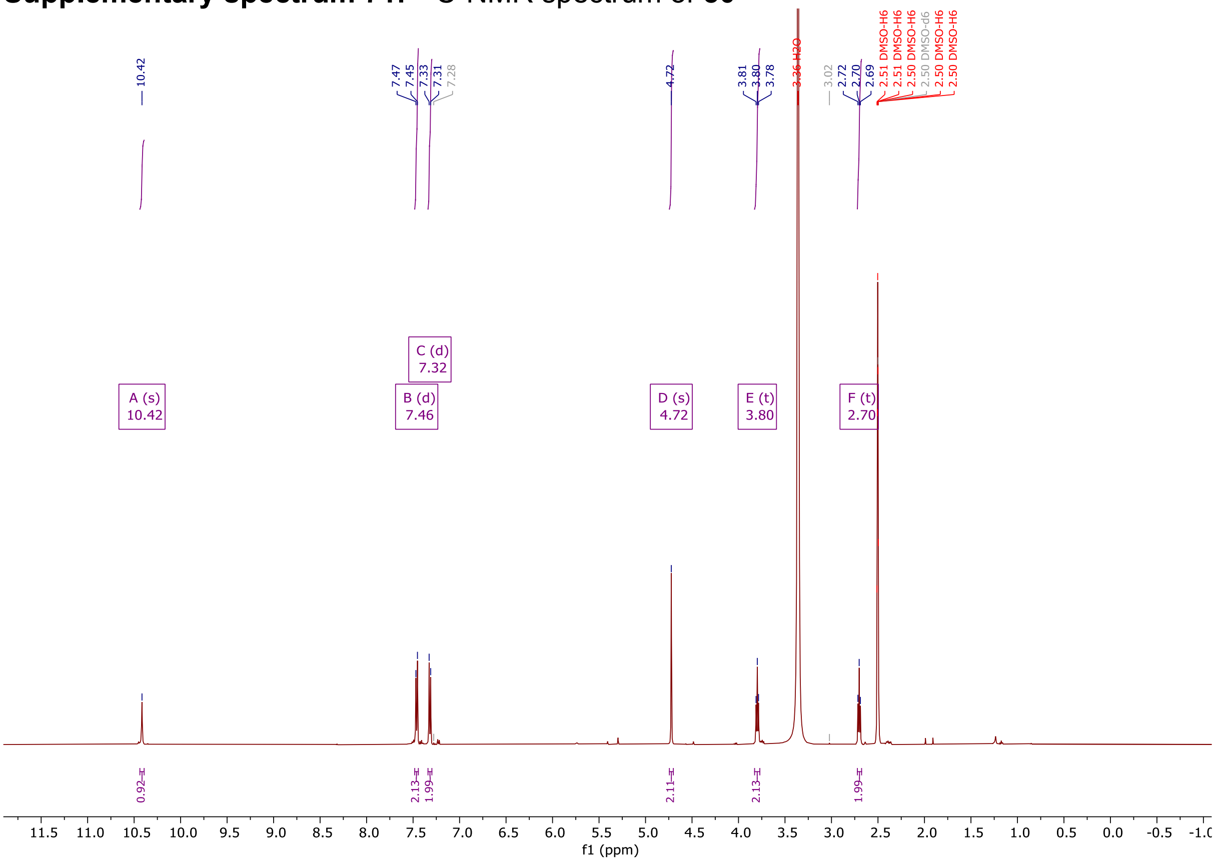

Supplementary spectrum 72: ^{1}H-NMR spectrum of **18**

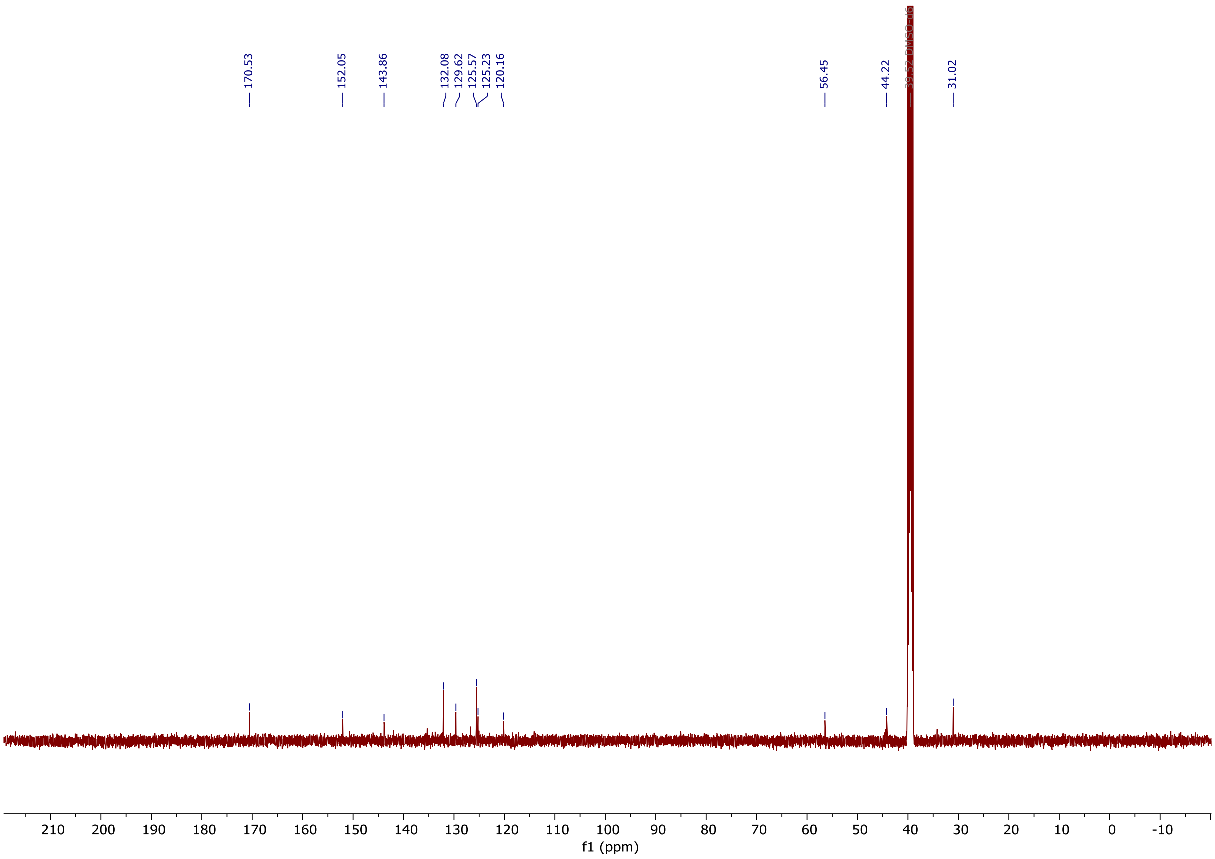

Supplementary spectrum 73: ^{13}C-NMR spectrum of **18**

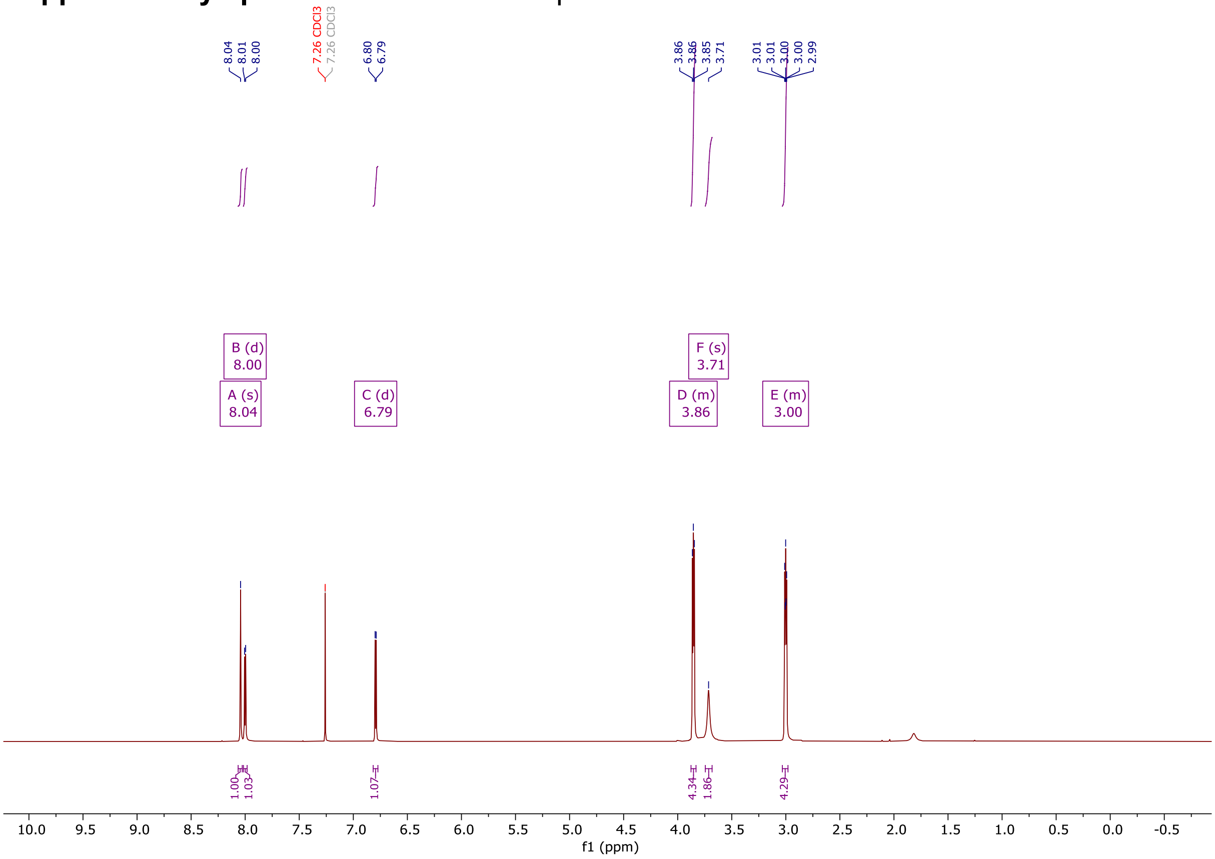

Supplementary spectrum 74: ^{1}H-NMR spectrum of **54**

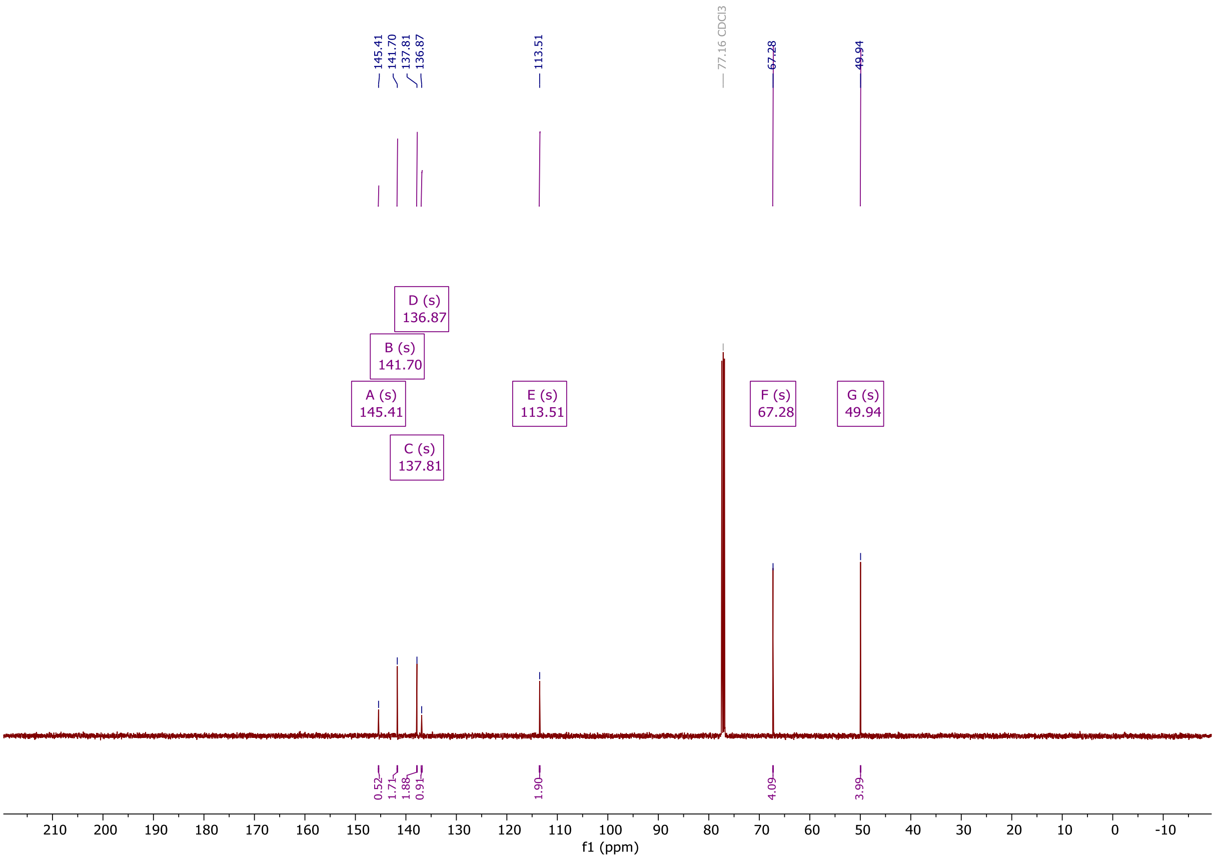

Supplementary spectrum 75: ^{13}C-NMR spectrum of **54**

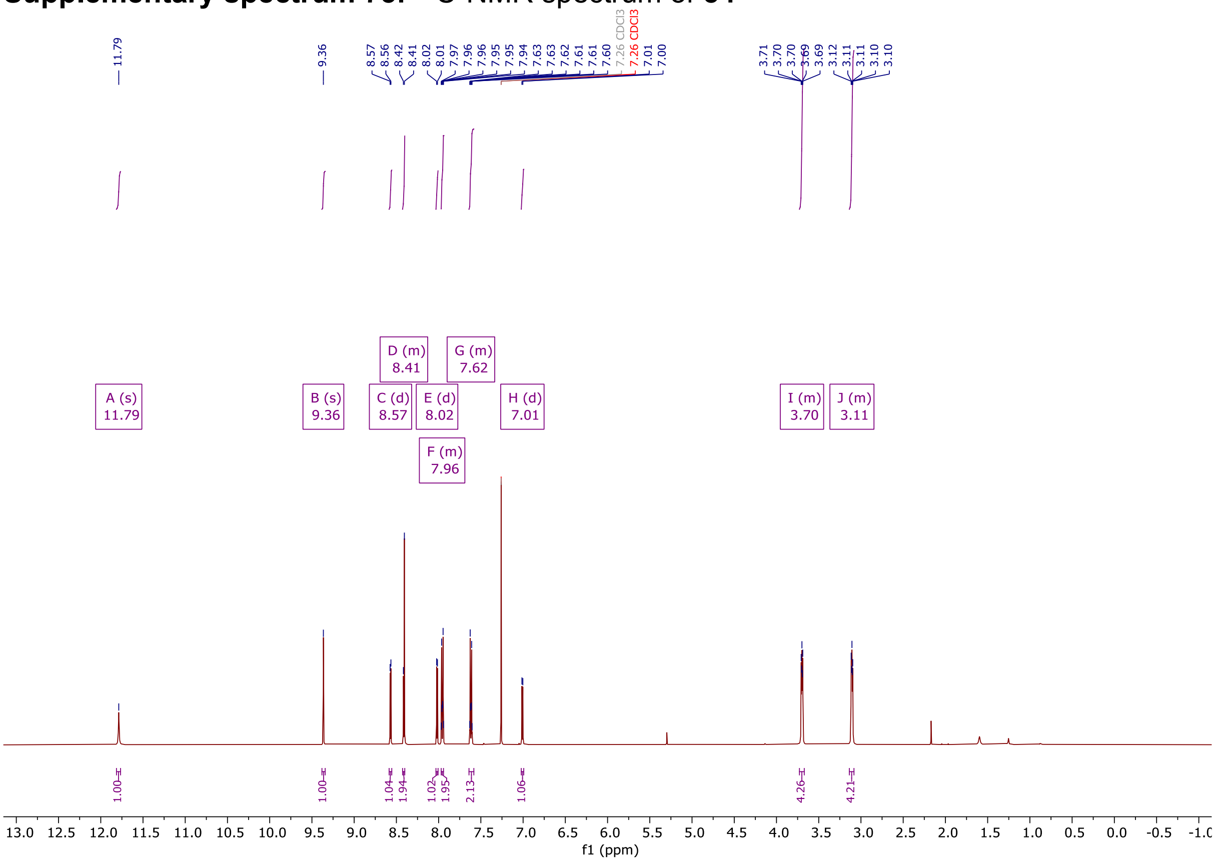

Supplementary spectrum 76: ^{1}H-NMR spectrum of **29**

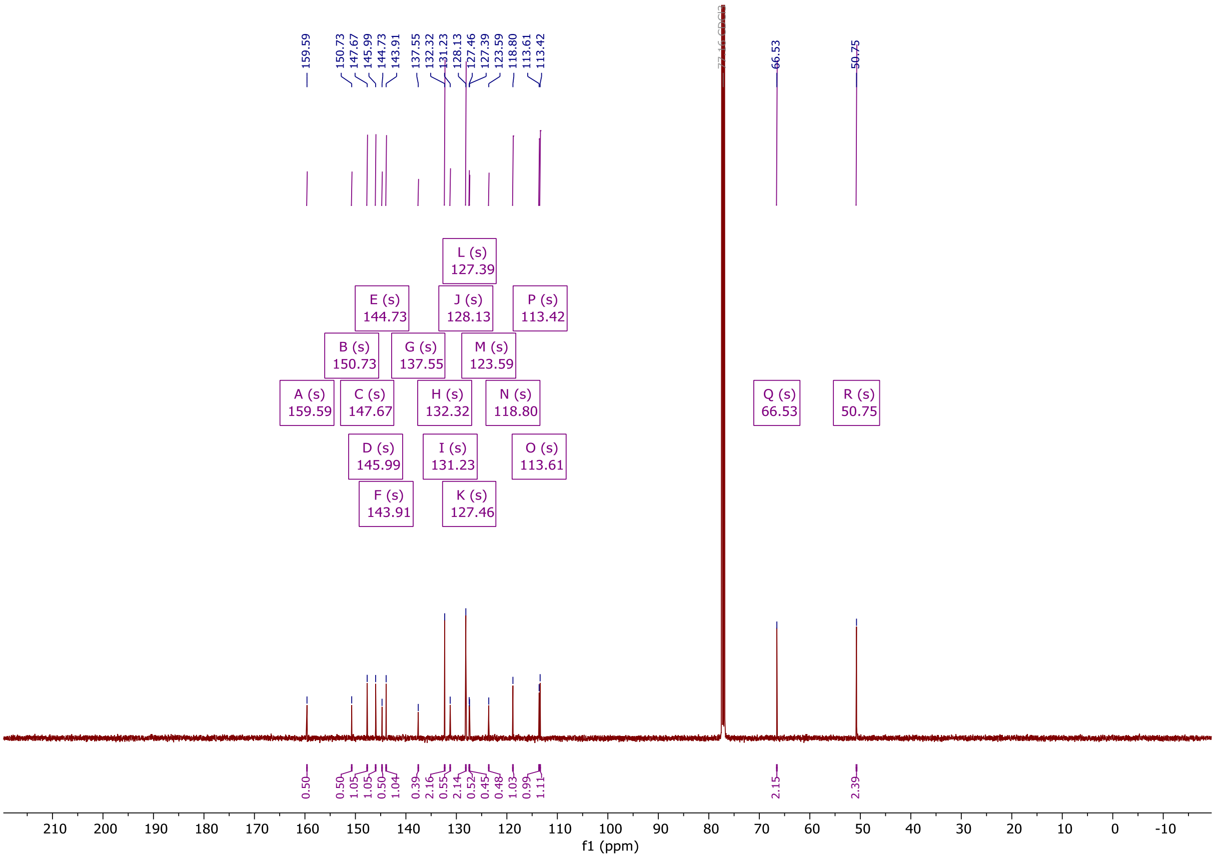

Supplementary spectrum 77: ^{13}C-NMR spectrum of **29**

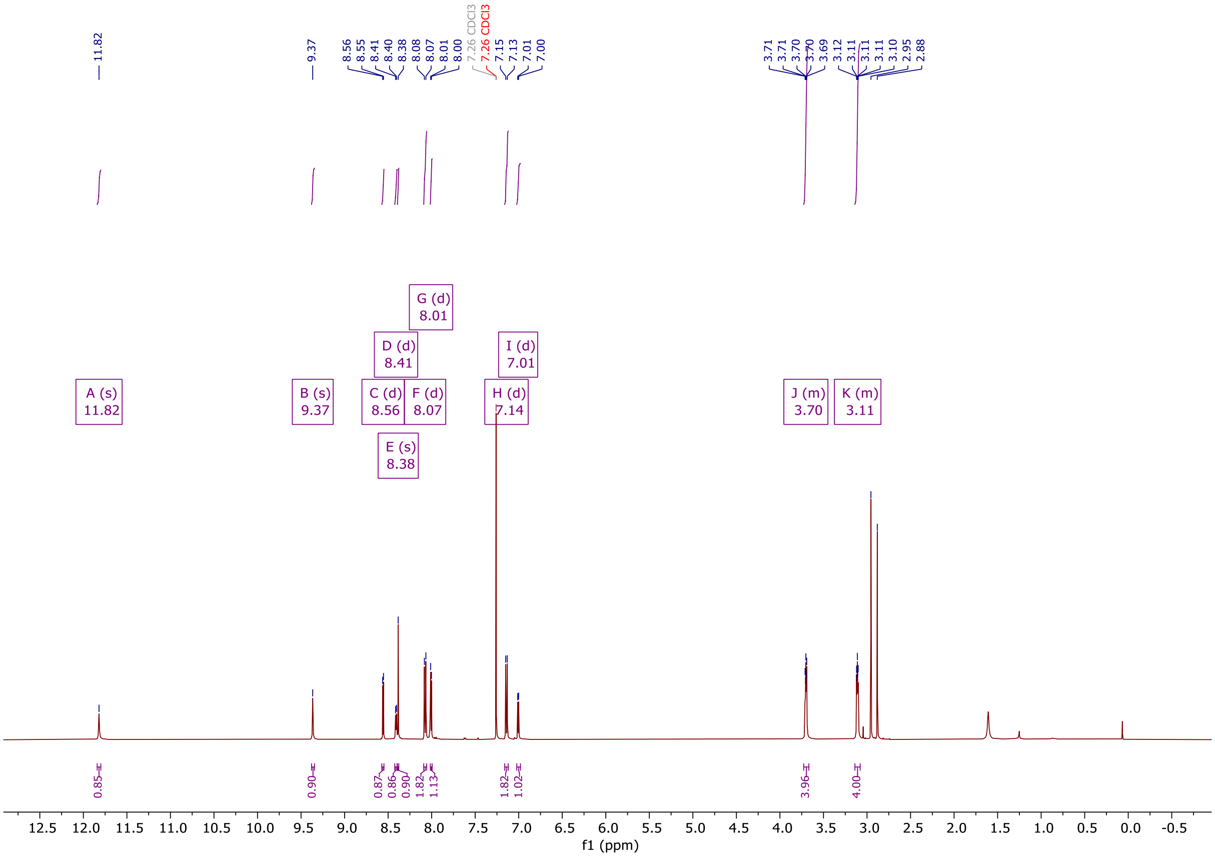

Supplementary spectrum 78: ^{1}H-NMR spectrum of **20**

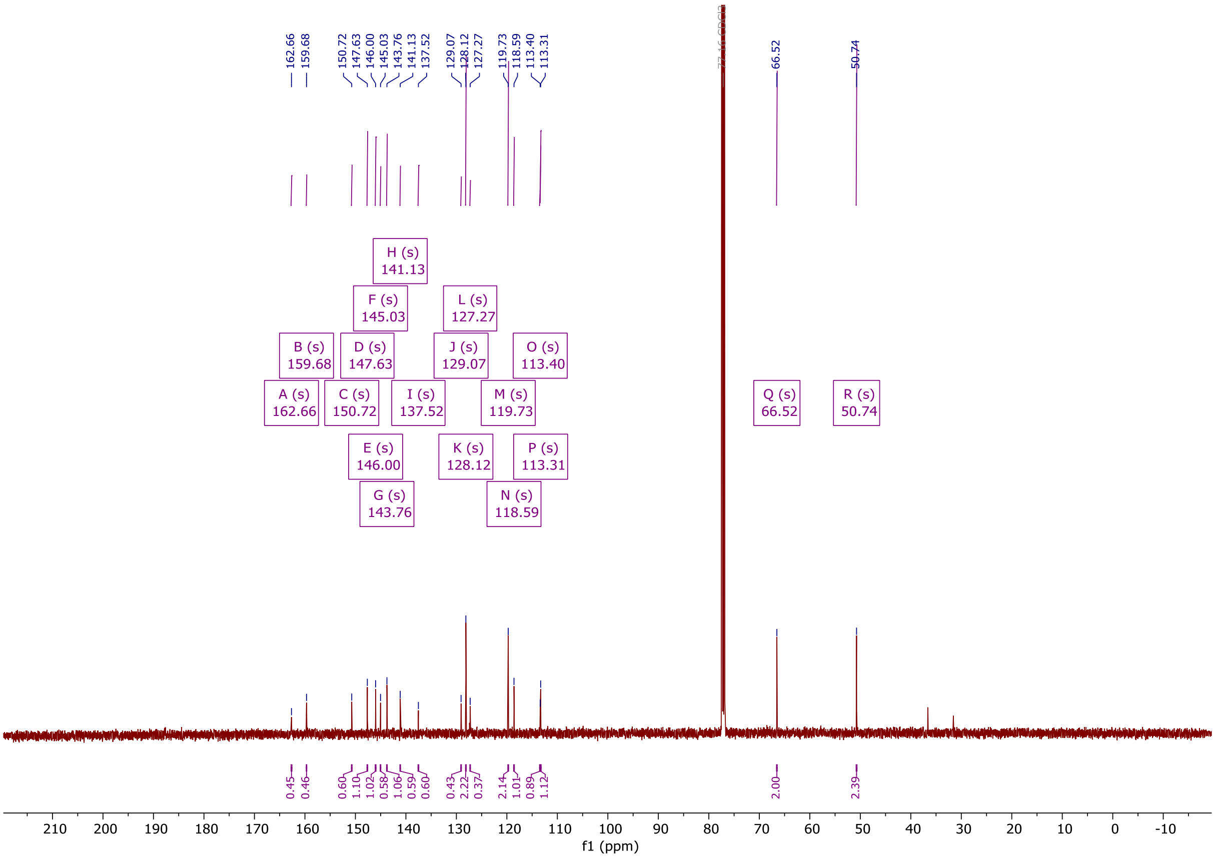

Supplementary spectrum 79: ^{13}C-NMR spectrum of **20**

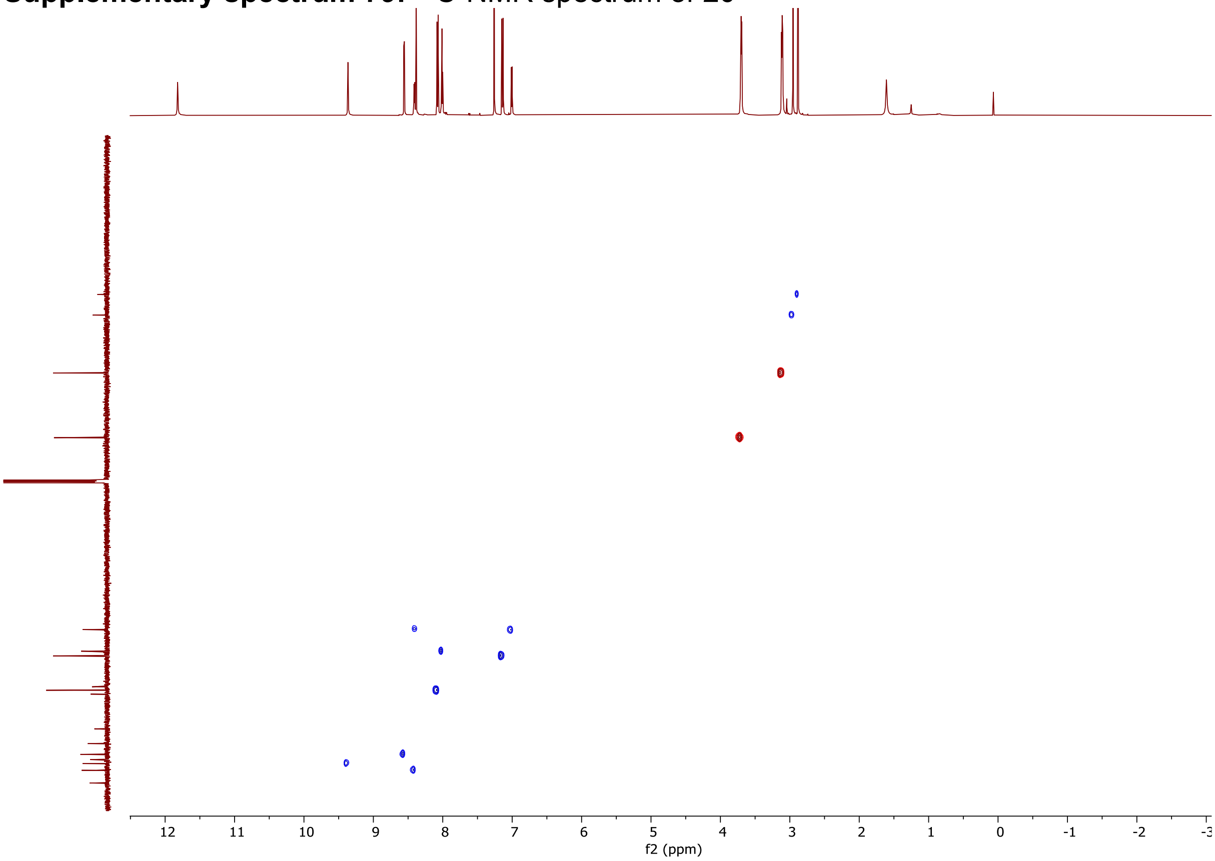

Supplementary spectrum 80: HSQC-NMR spectrum of **20**

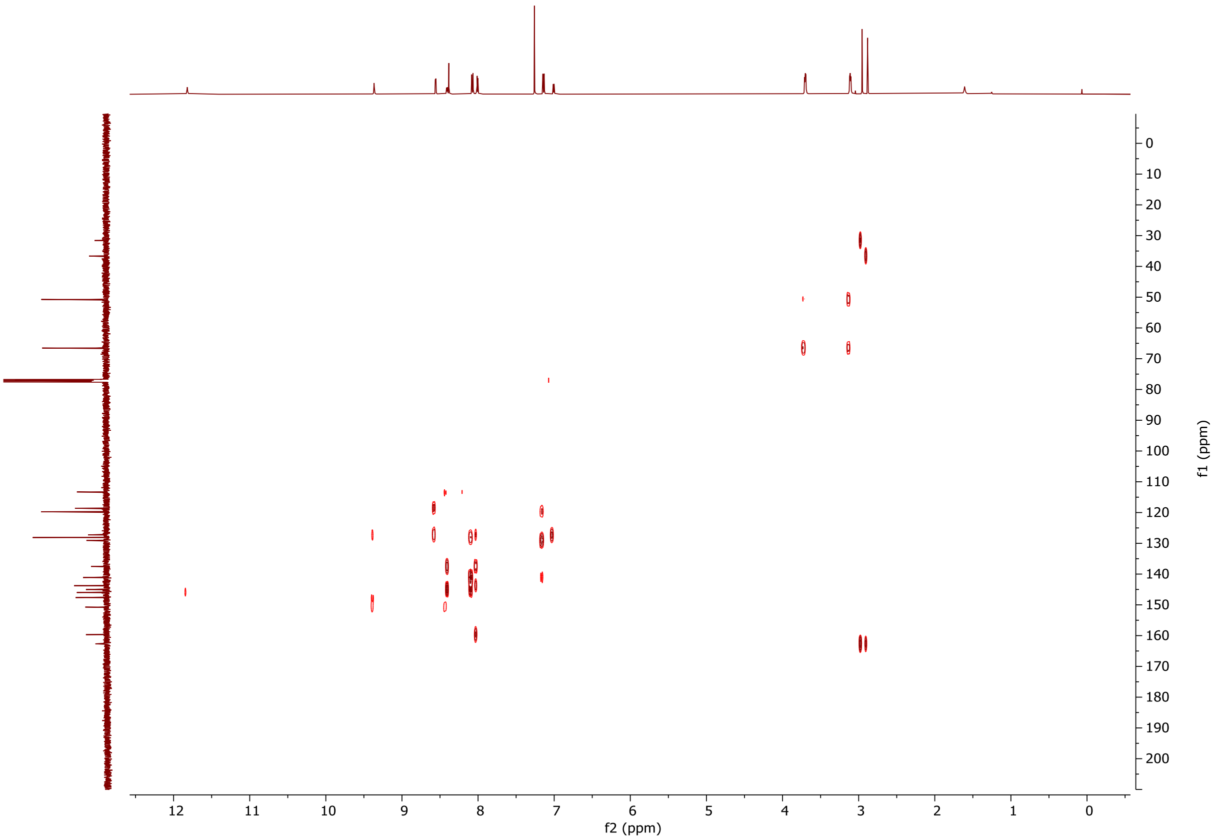

Supplementary spectrum 81: HMBC-NMR spectrum of **20**

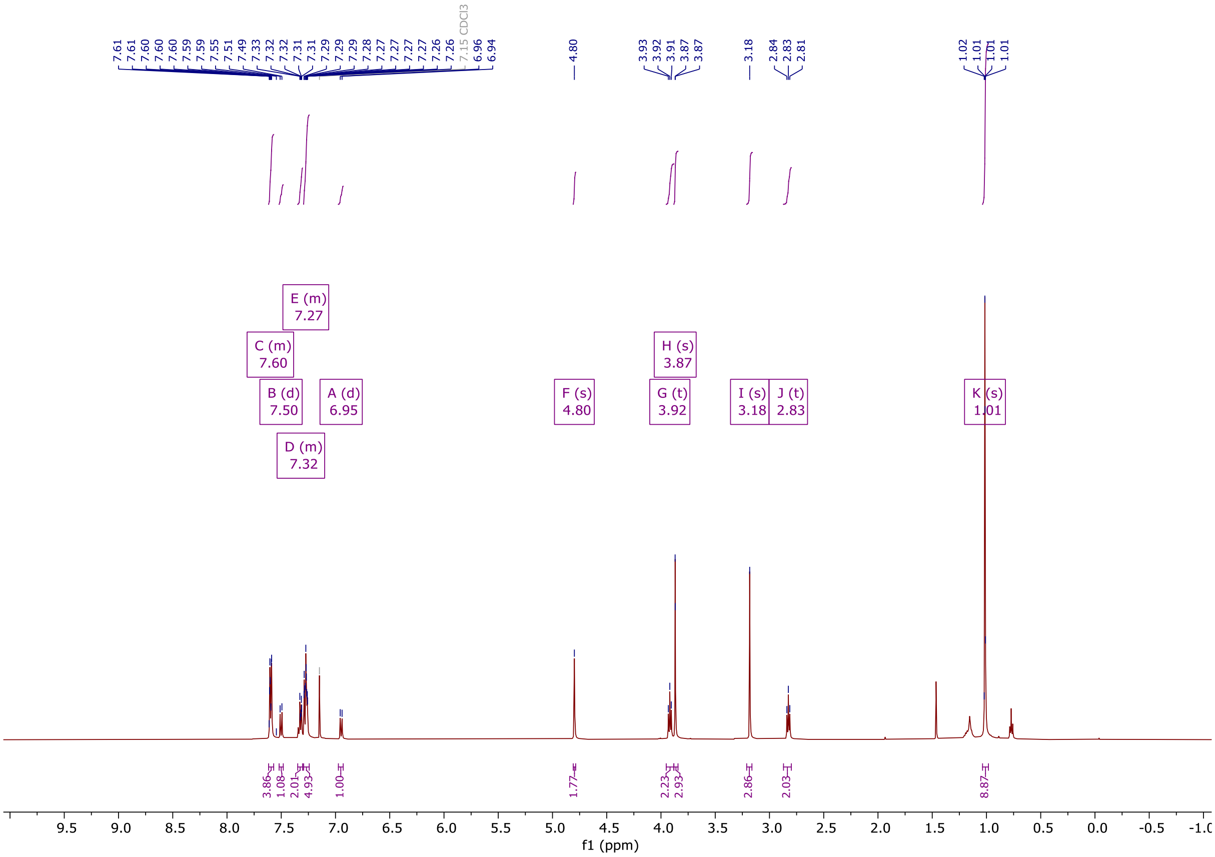

Supplementary spectrum 82: ^{1}H-NMR spectrum of **55**

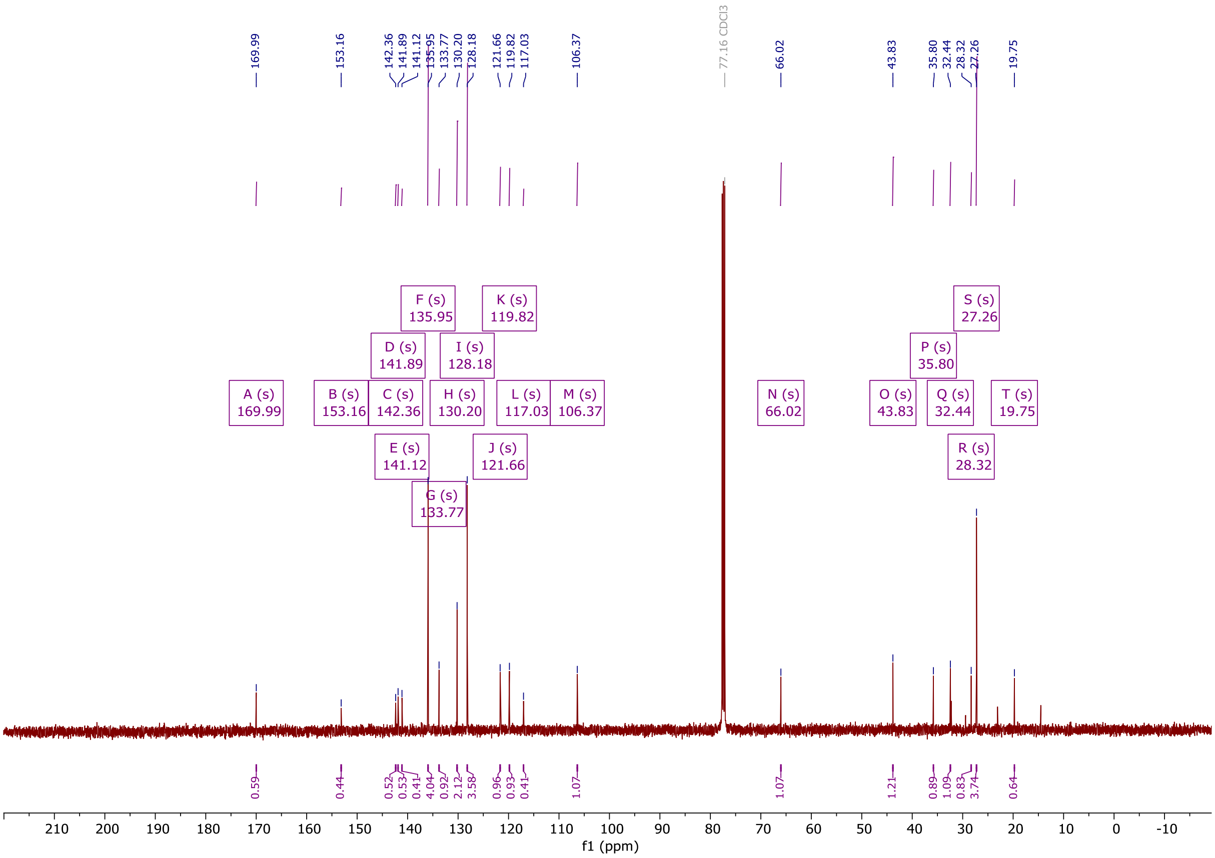

Supplementary spectrum 83: ^{13}C-NMR spectrum of **55**

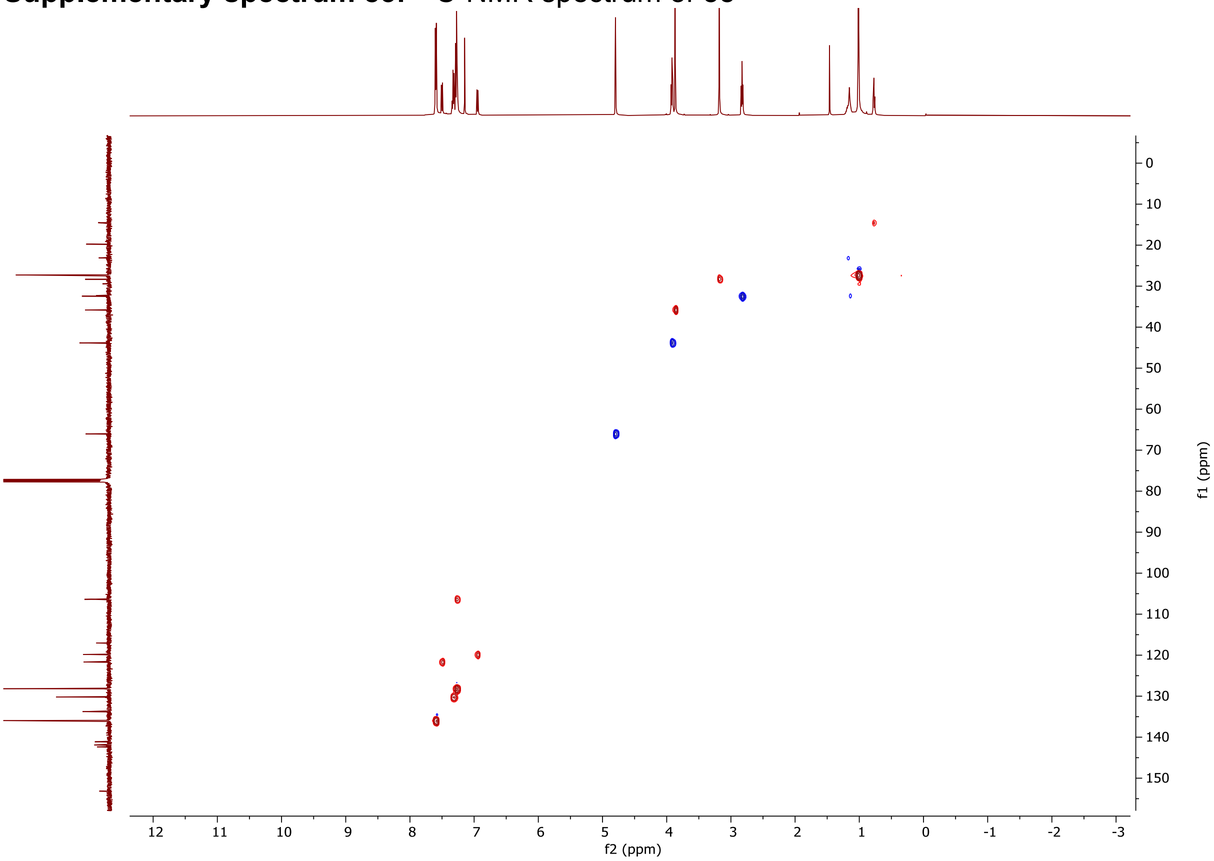

Supplementary spectrum 84: HSQC-NMR spectrum of **55**

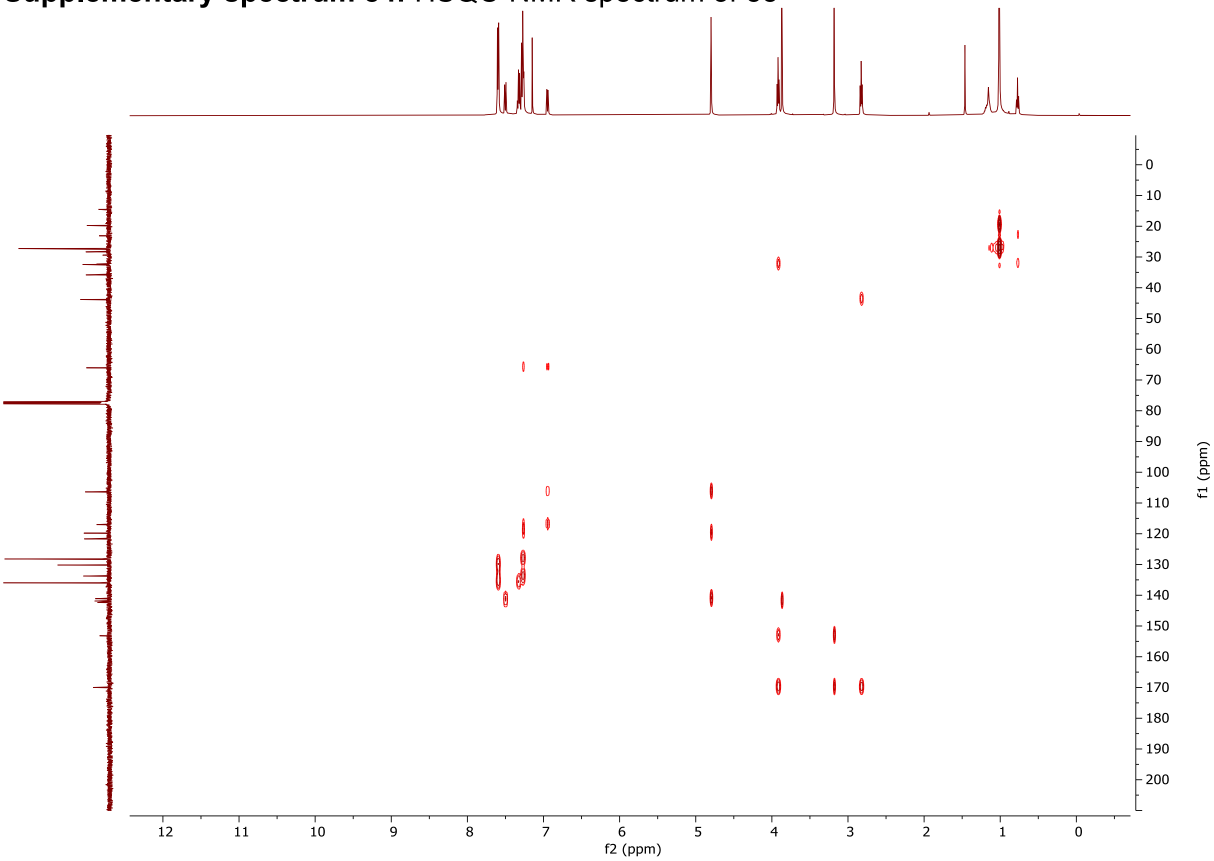

Supplementary spectrum 85: HMBC-NMR spectrum of **55**

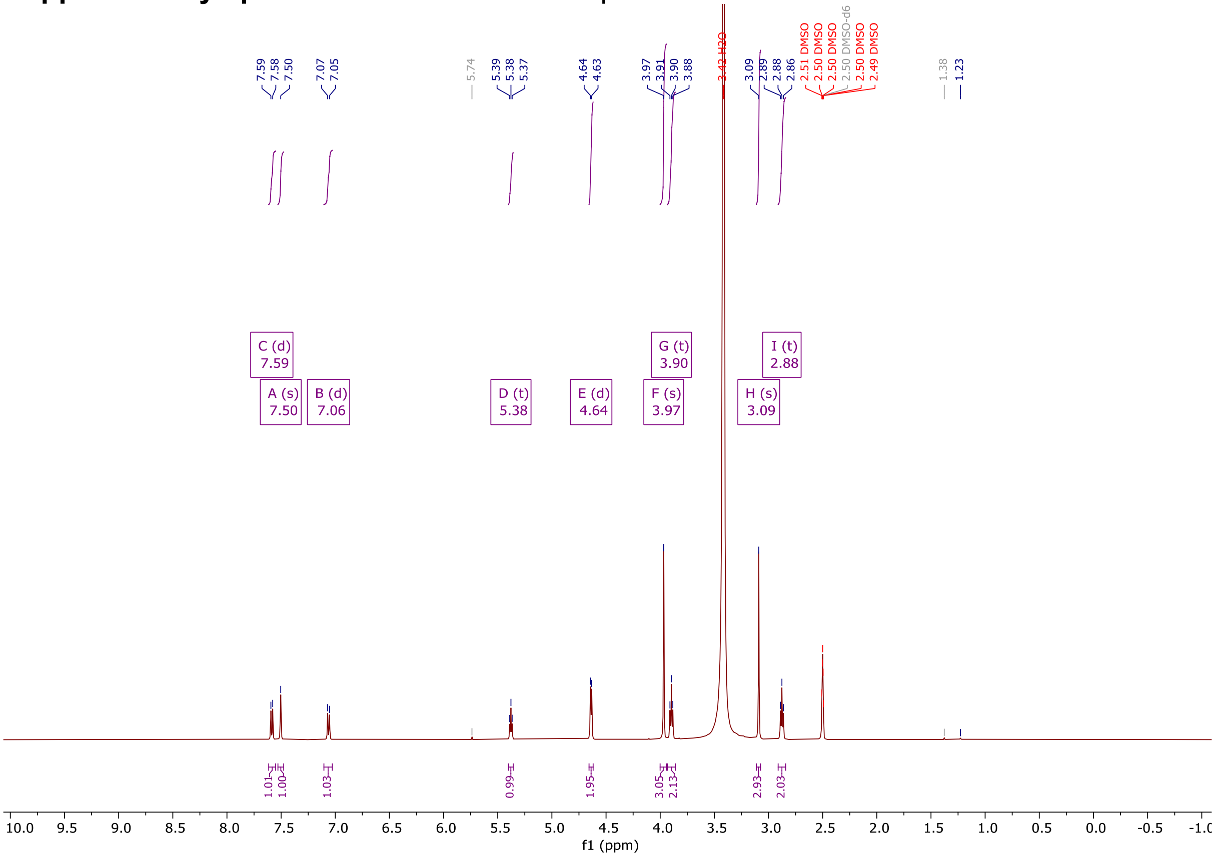

Supplementary spectrum 86: ^{1}H-NMR spectrum of **55**

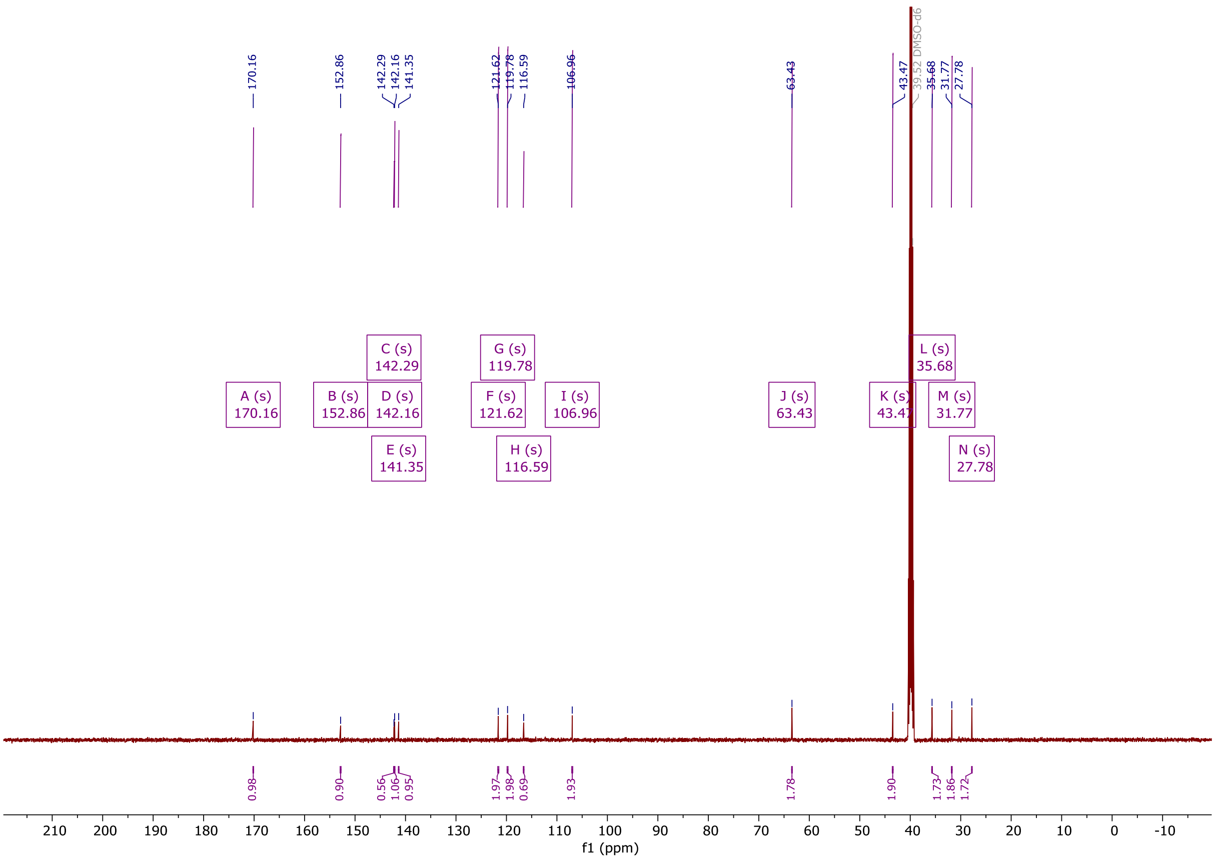

Supplementary spectrum 87: ^{13}C-NMR spectrum of **56**

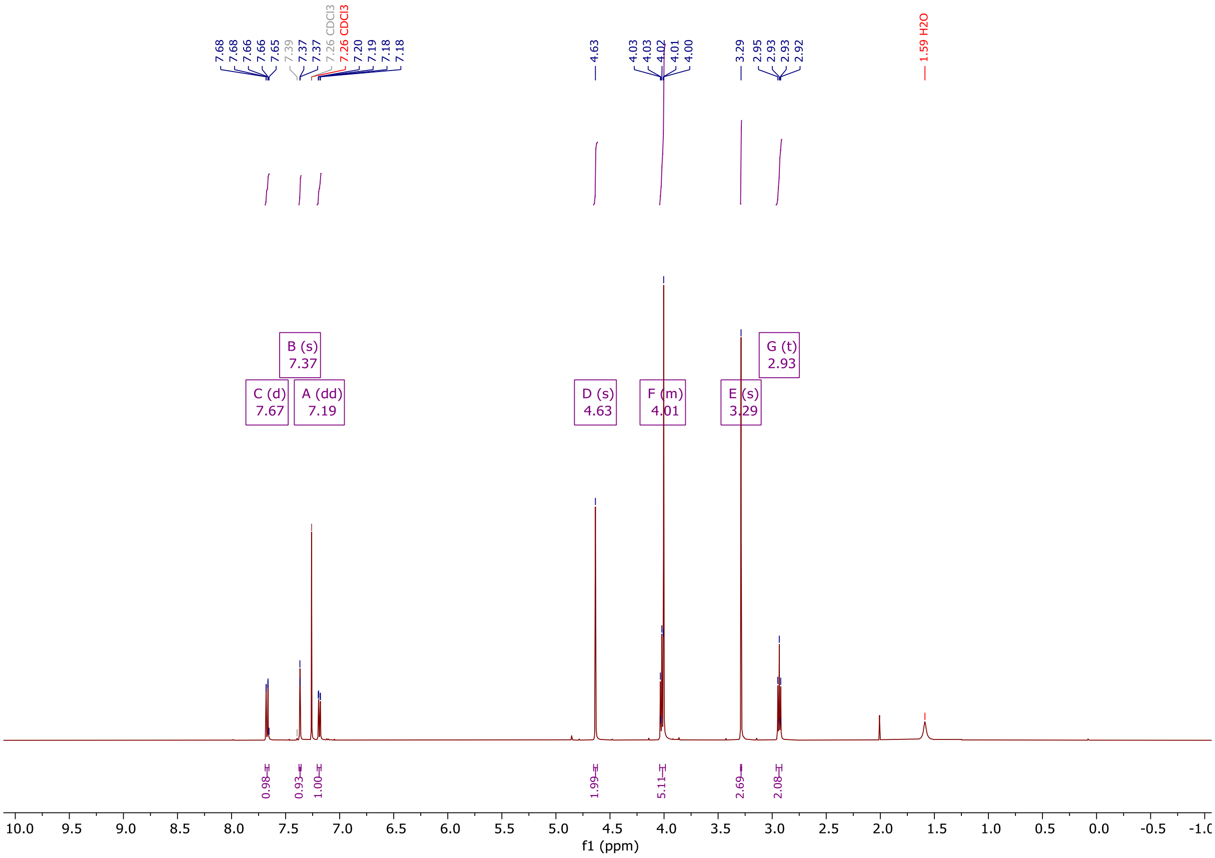

Supplementary spectrum 88: ^{1}H-NMR spectrum of **57**

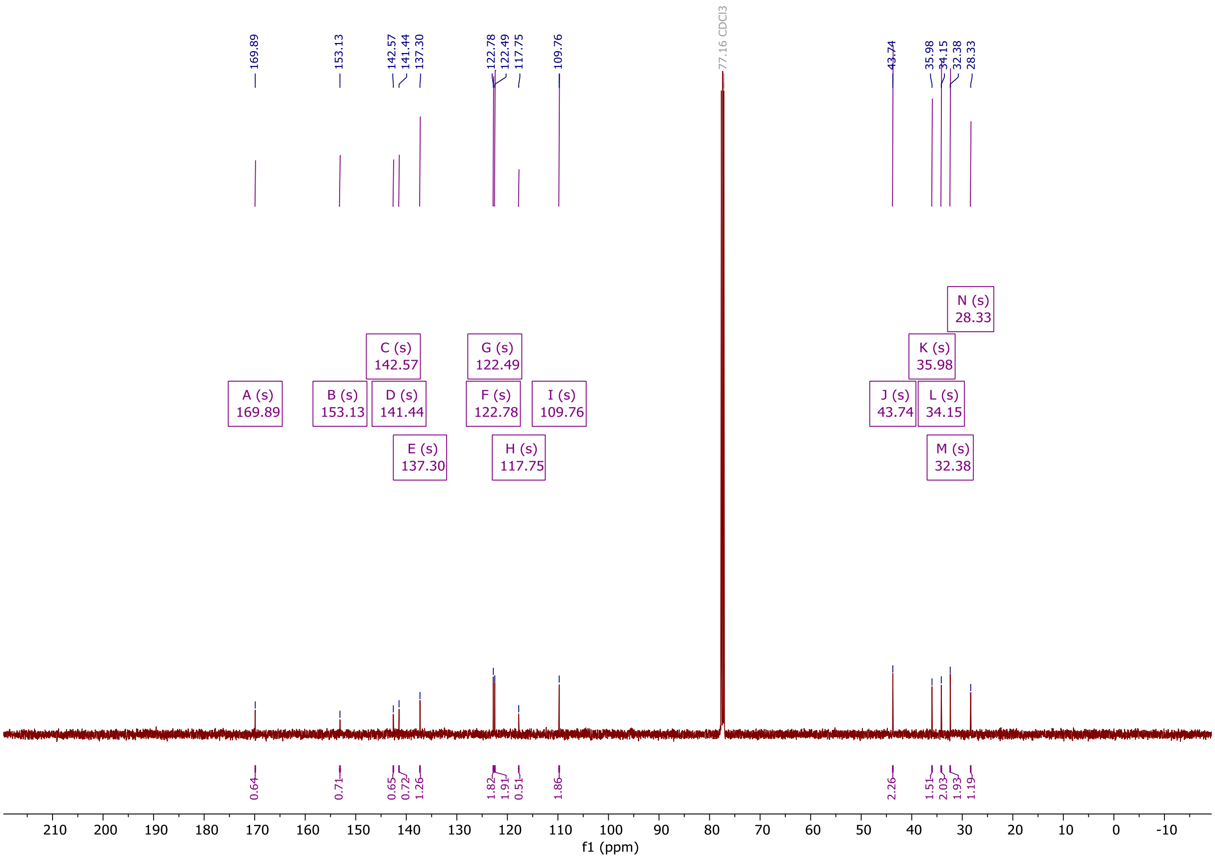

Supplementary spectrum 89: ^{13}C-NMR spectrum of **57**

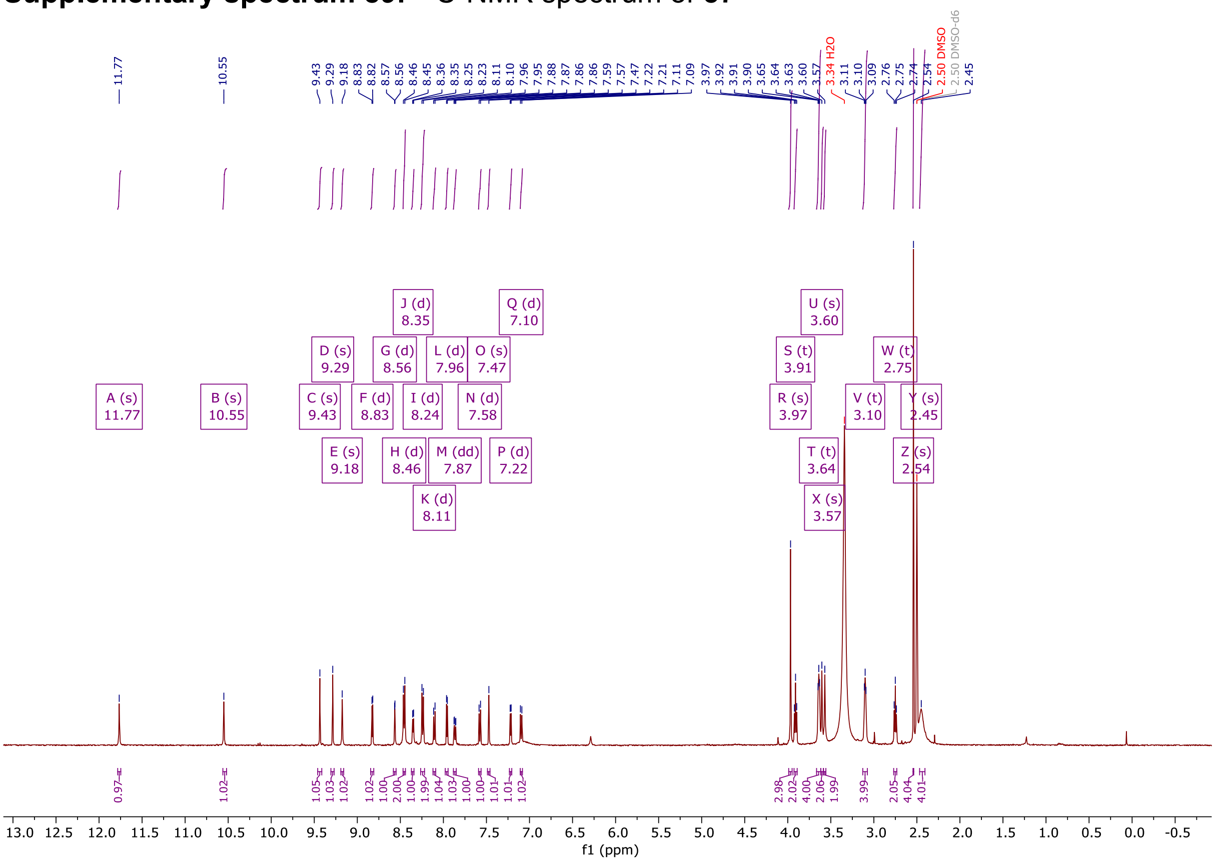

Supplementary spectrum 91: ^{1}H-NMR spectrum of KH1 (**24**)

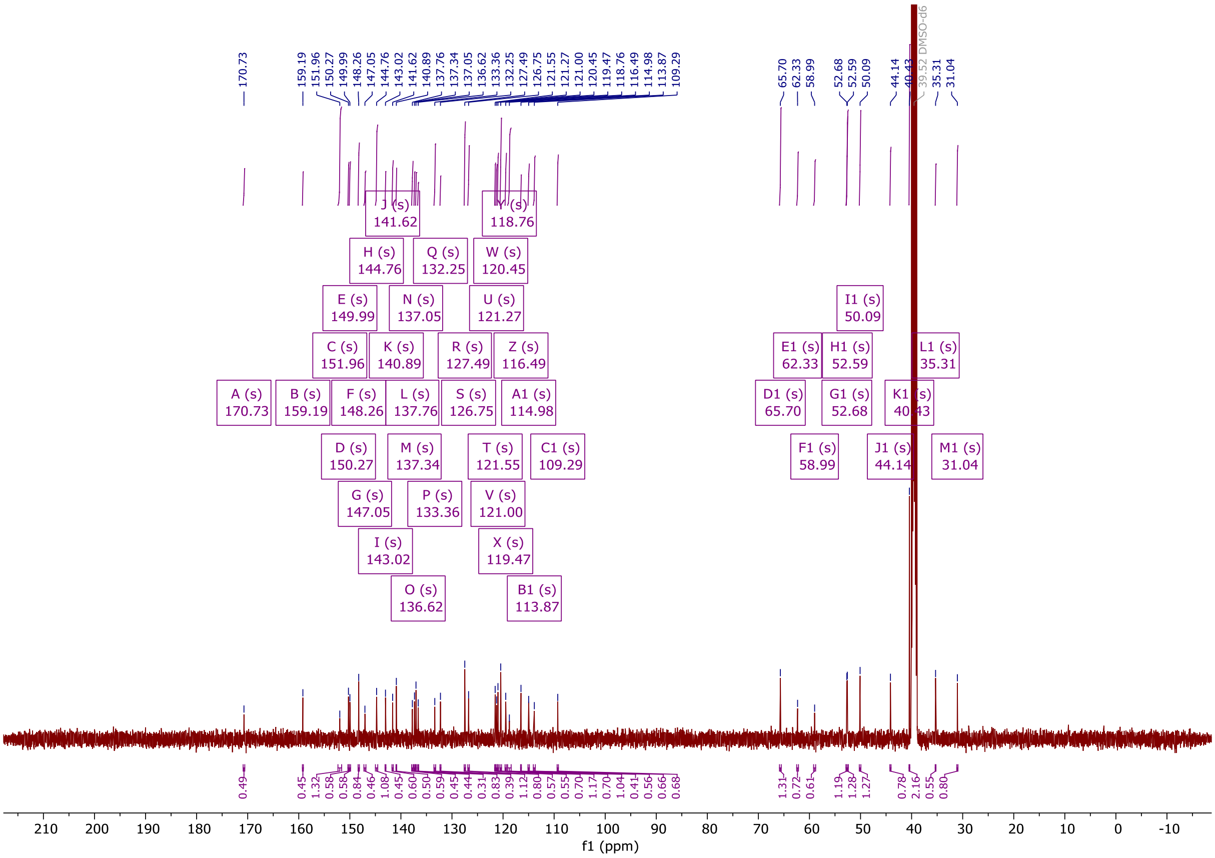

Supplementary spectrum 92: ^{13}C-NMR spectrum of KH1 (**24**)

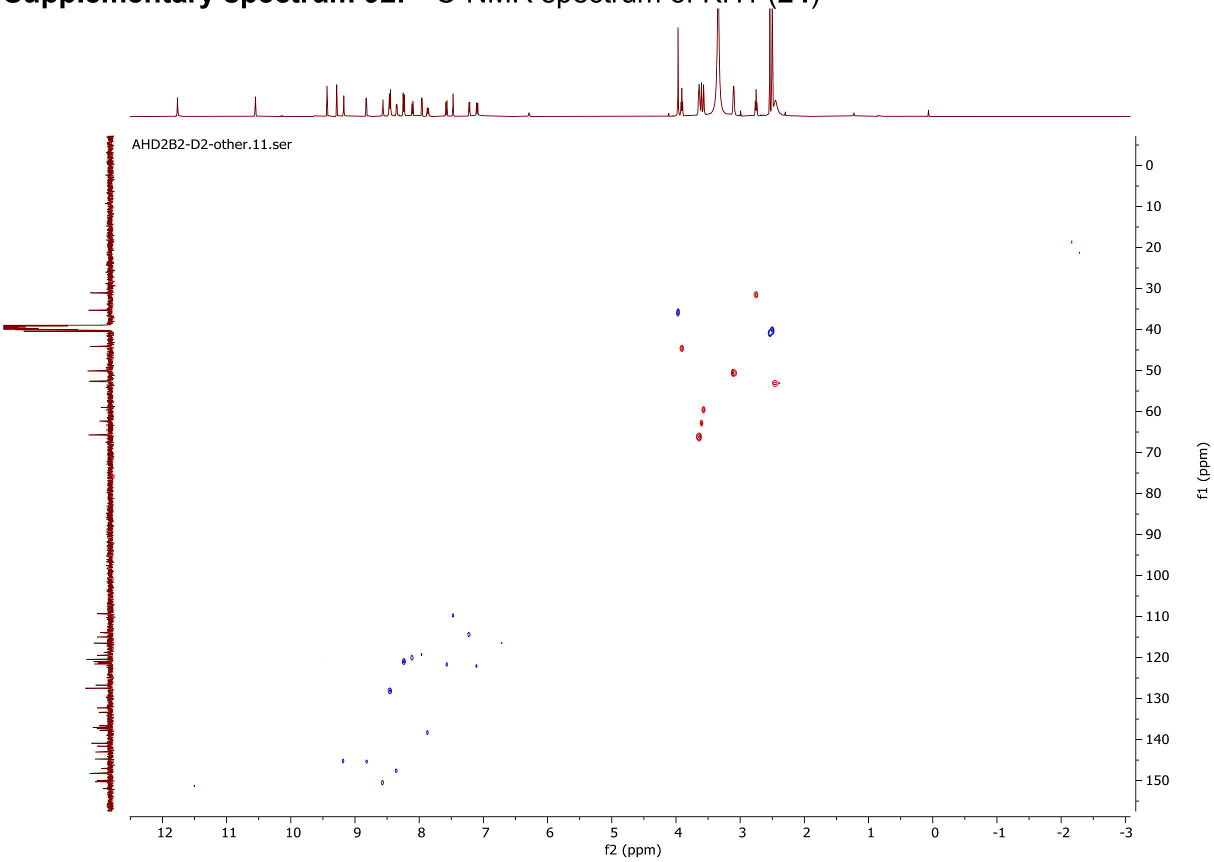

Supplementary spectrum 93: HSQC-NMR spectrum of KH1 (**24**)

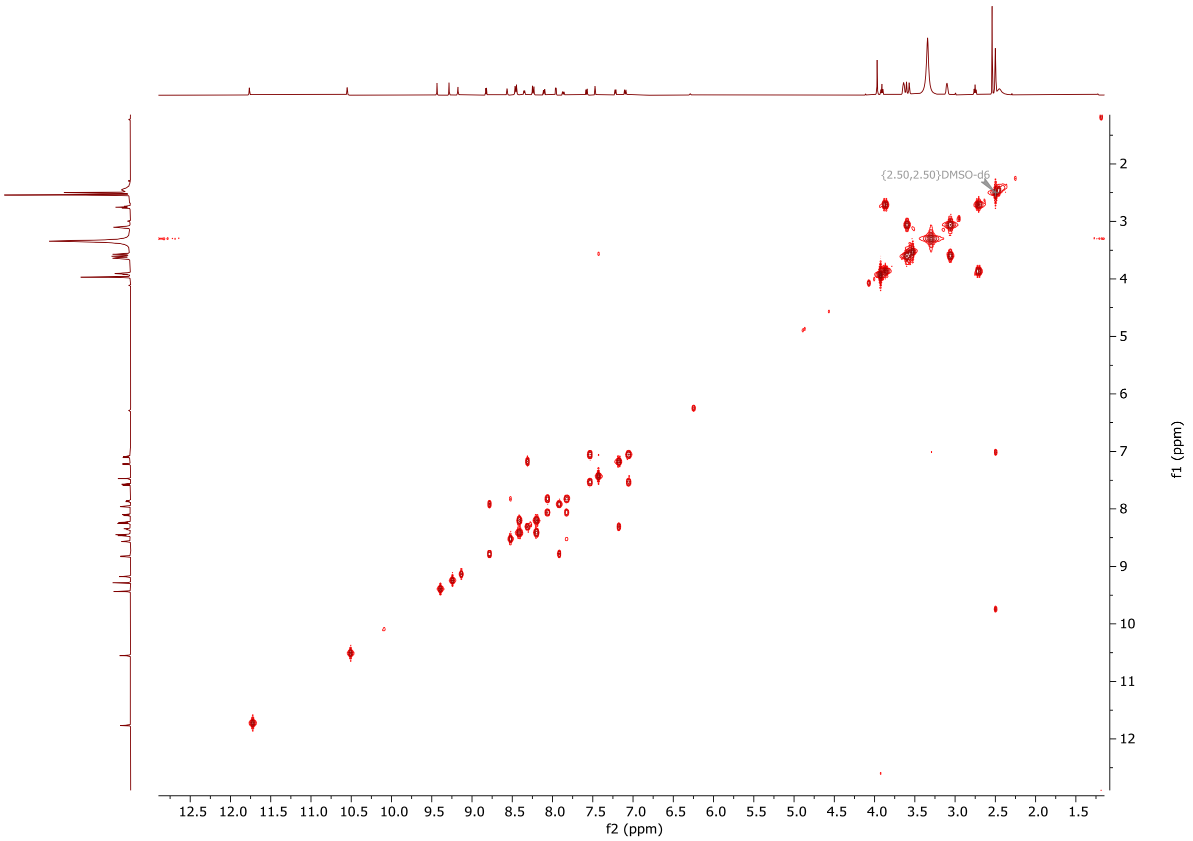

Supplementary spectrum 94: COSY-NMR spectrum of KH1 (**24**)

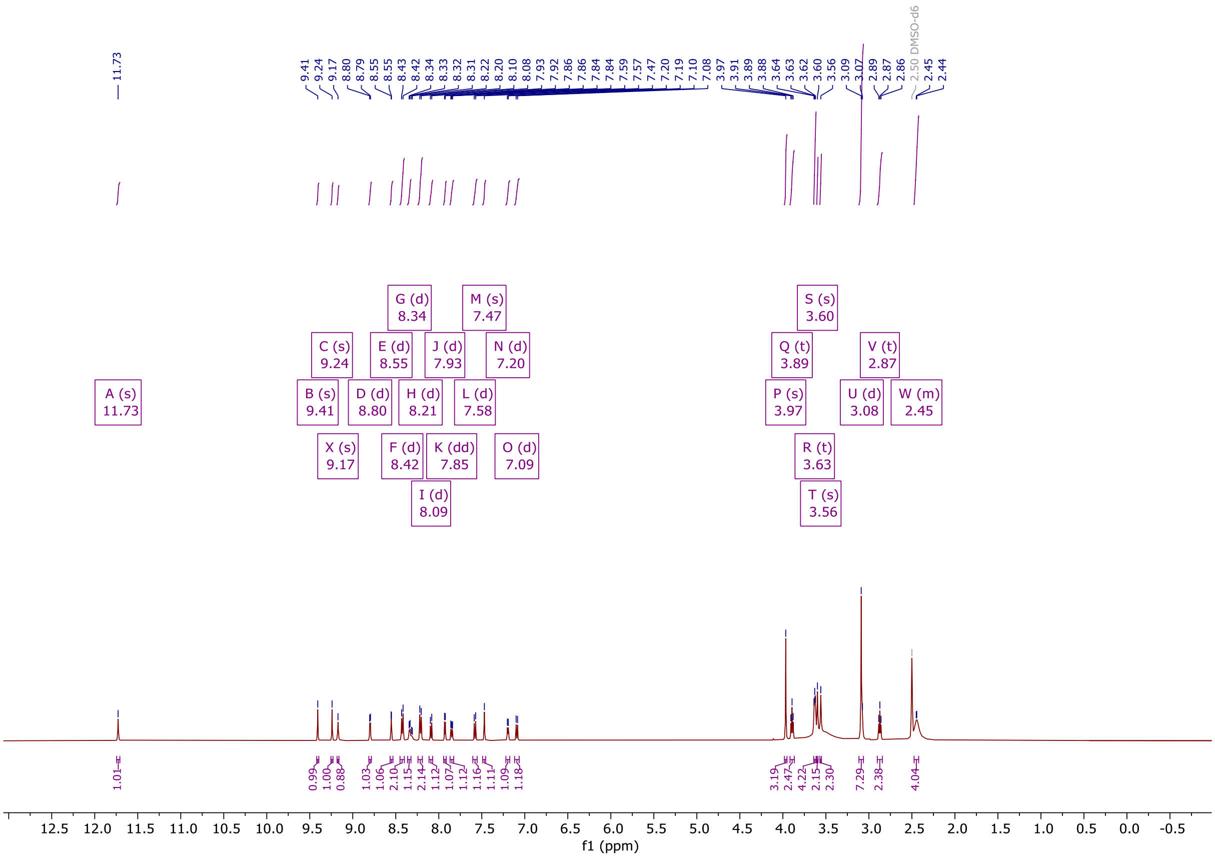

Supplementary spectrum 95: ^{1}H-NMR spectrum of **27**

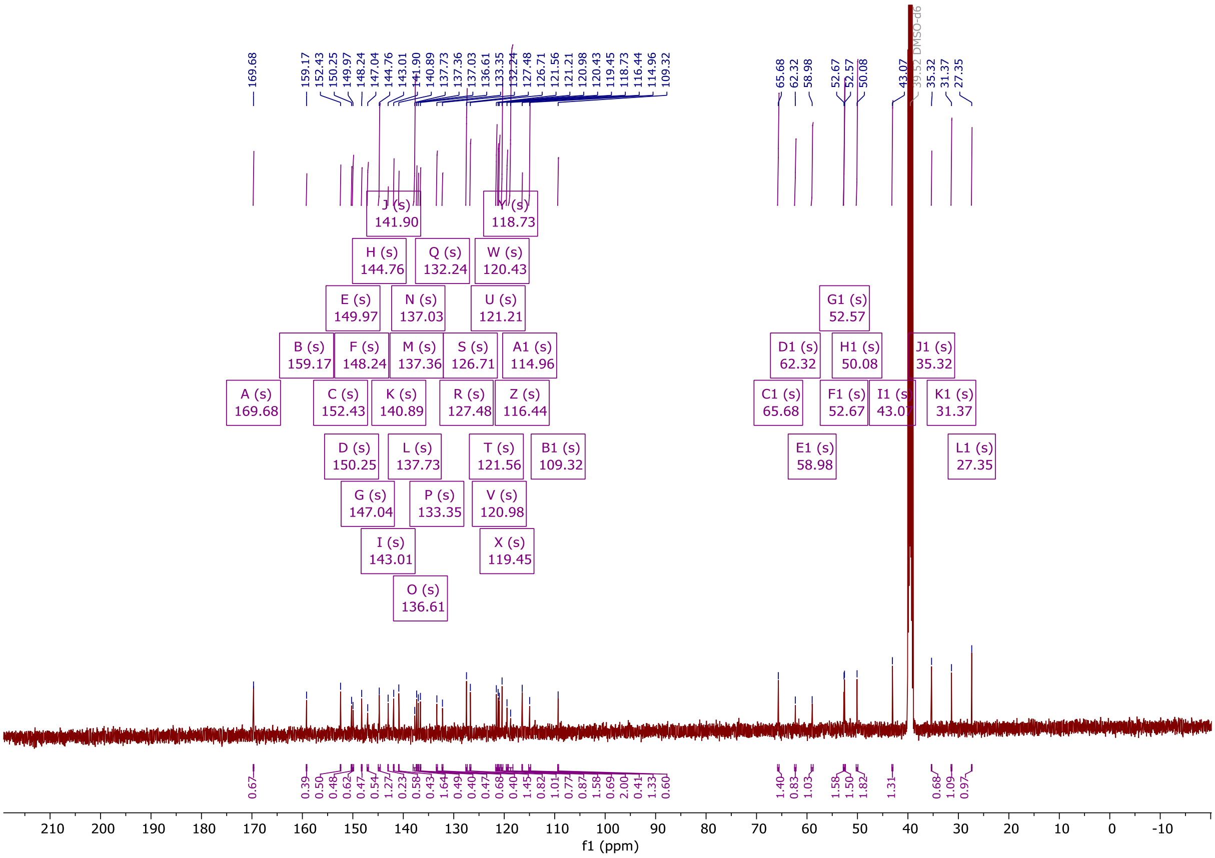

Supplementary spectrum 96: ^{13}C-NMR spectrum of **27**

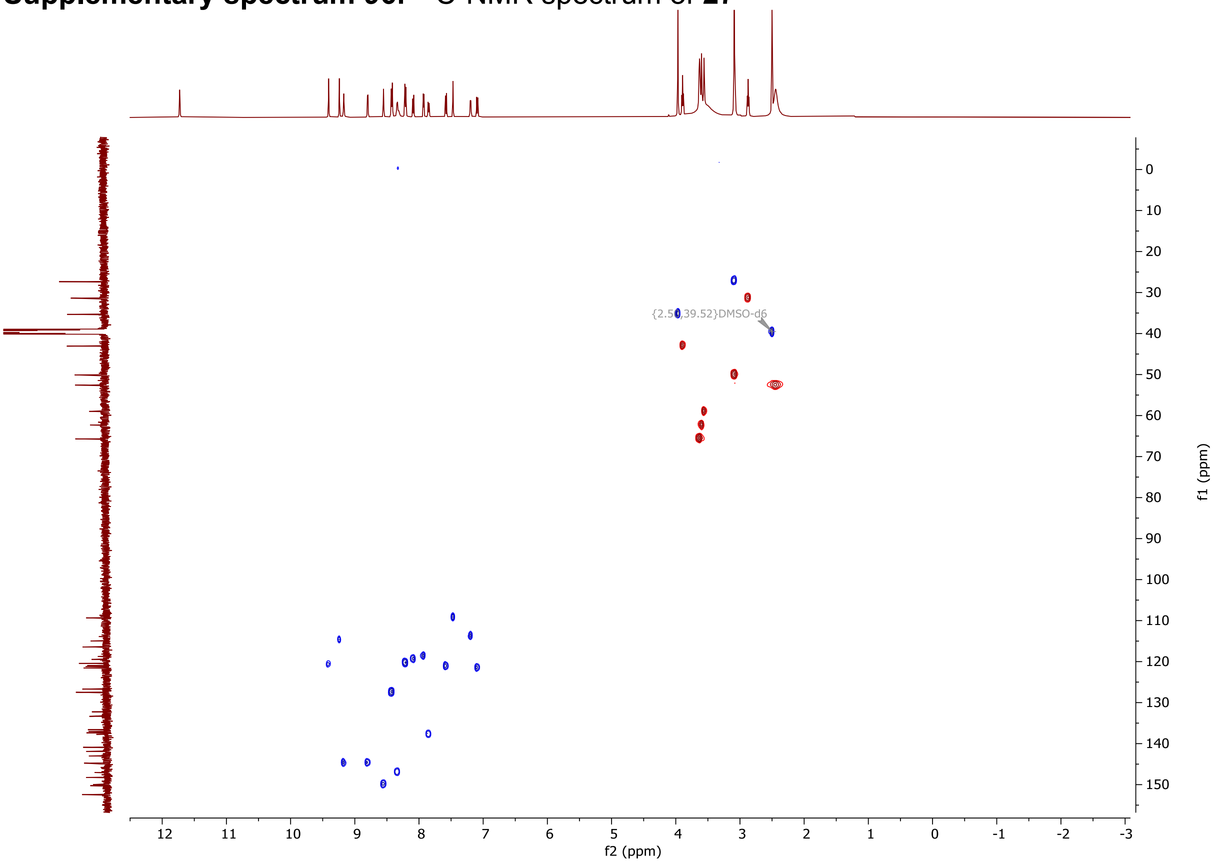

Supplementary spectrum 97: HSQC-NMR spectrum of **27**

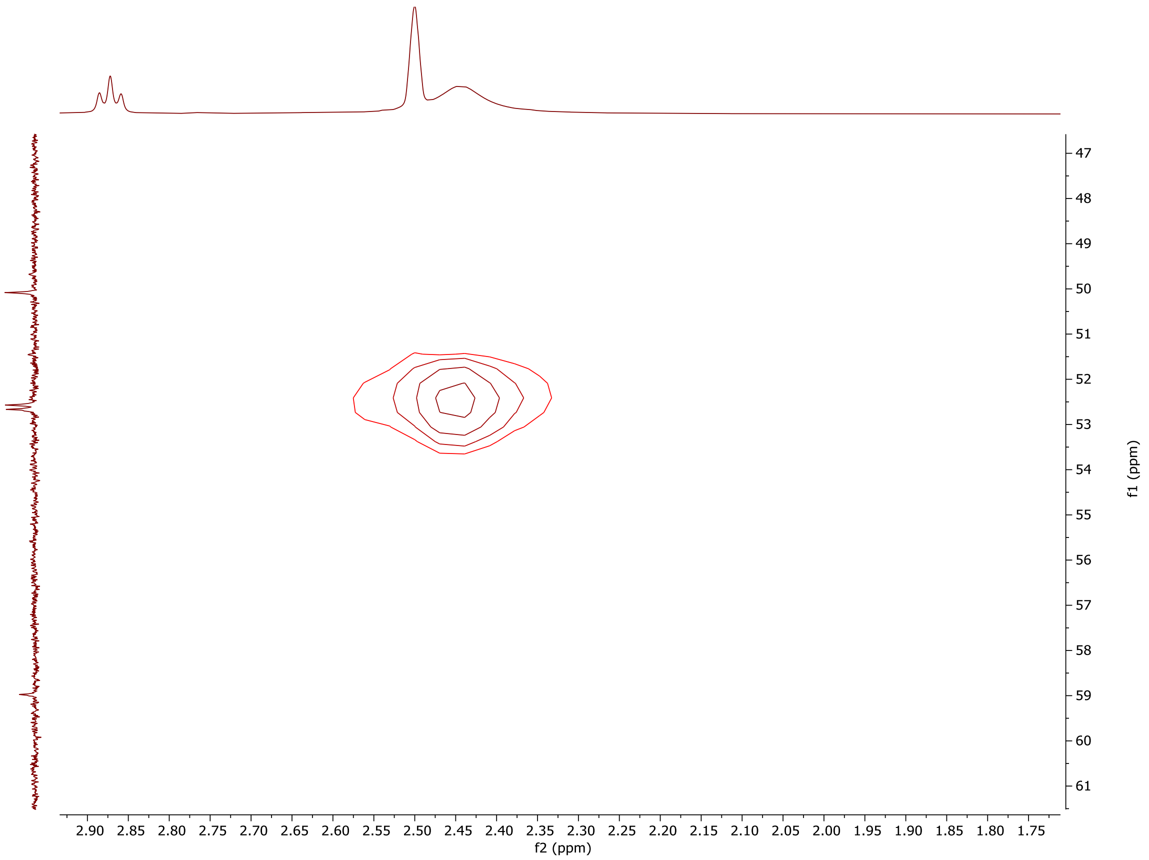

Supplementary spectrum 98: Zoomed in HSQC-NMR spectrum of **27**

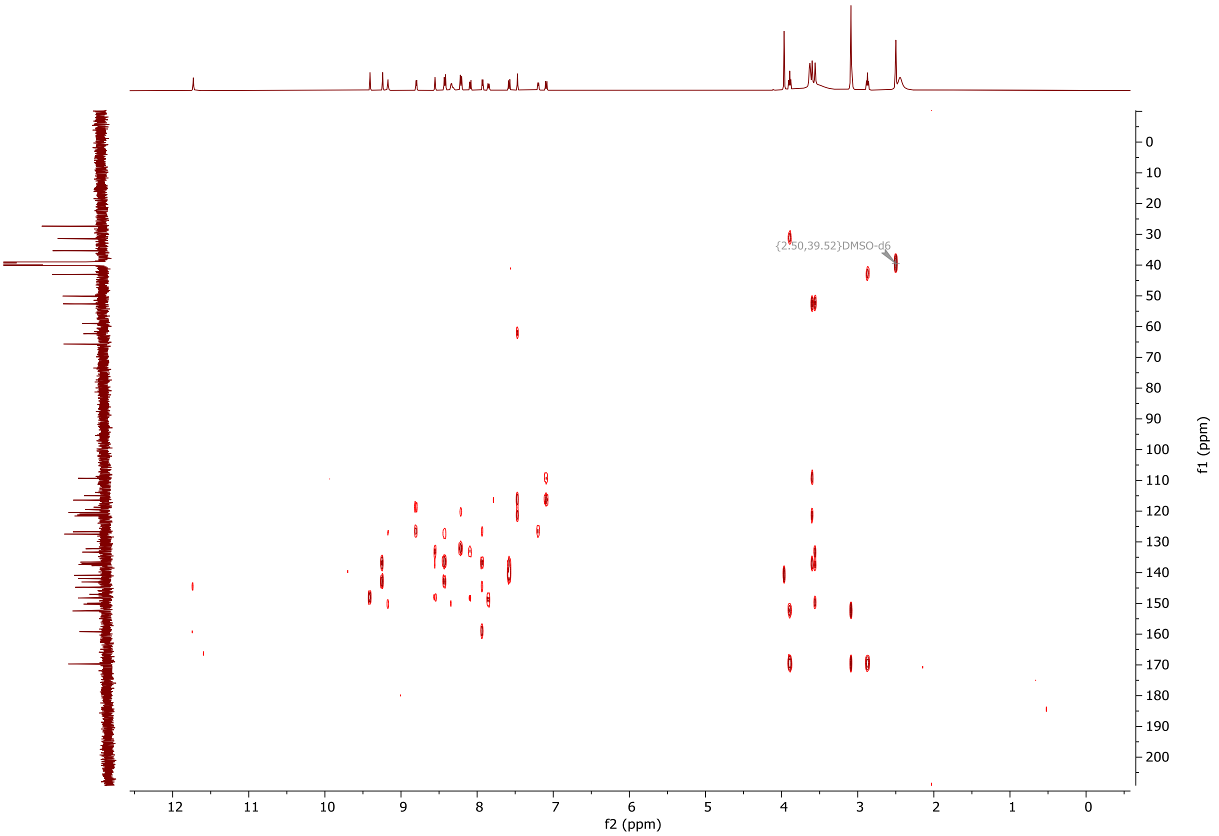

Supplementary spectrum 99: HMBC-NMR spectrum of **27**

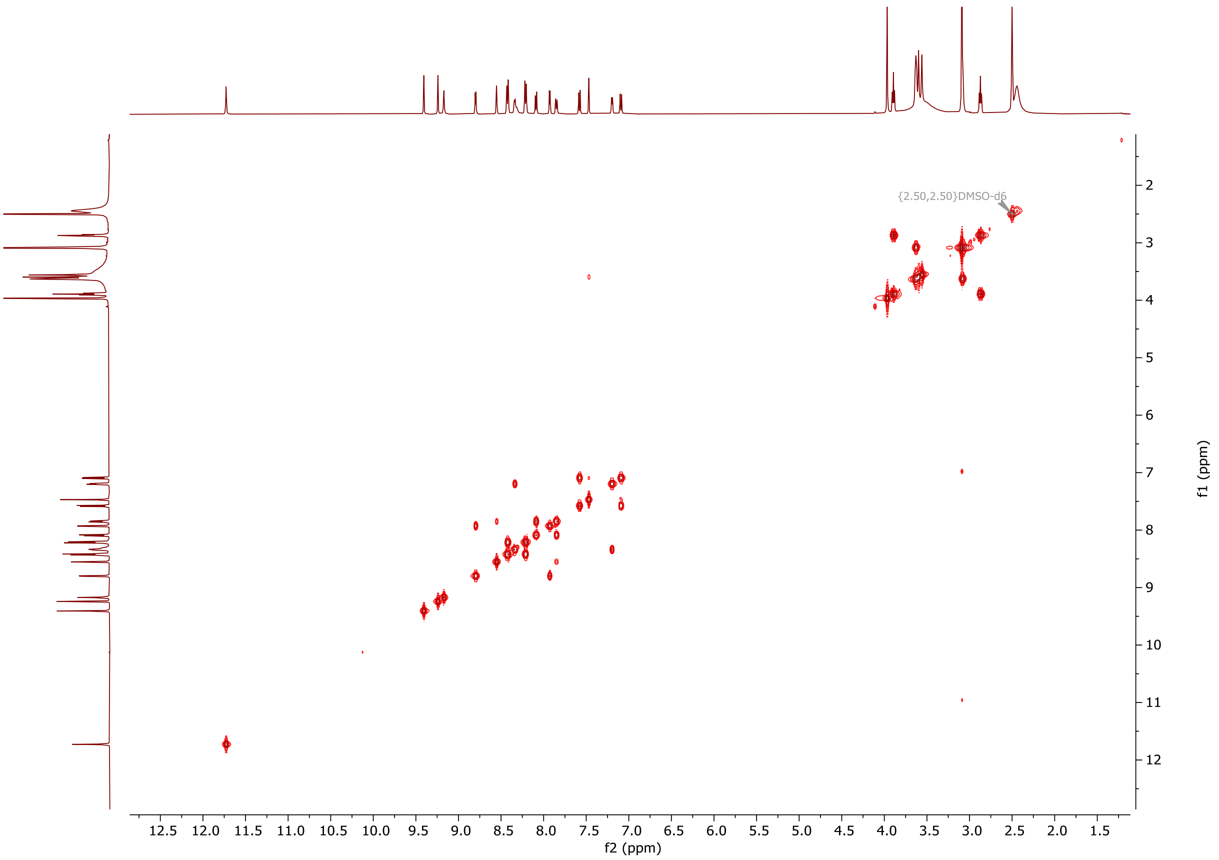

Supplementary spectrum 100: COSY-NMR spectrum of **27**

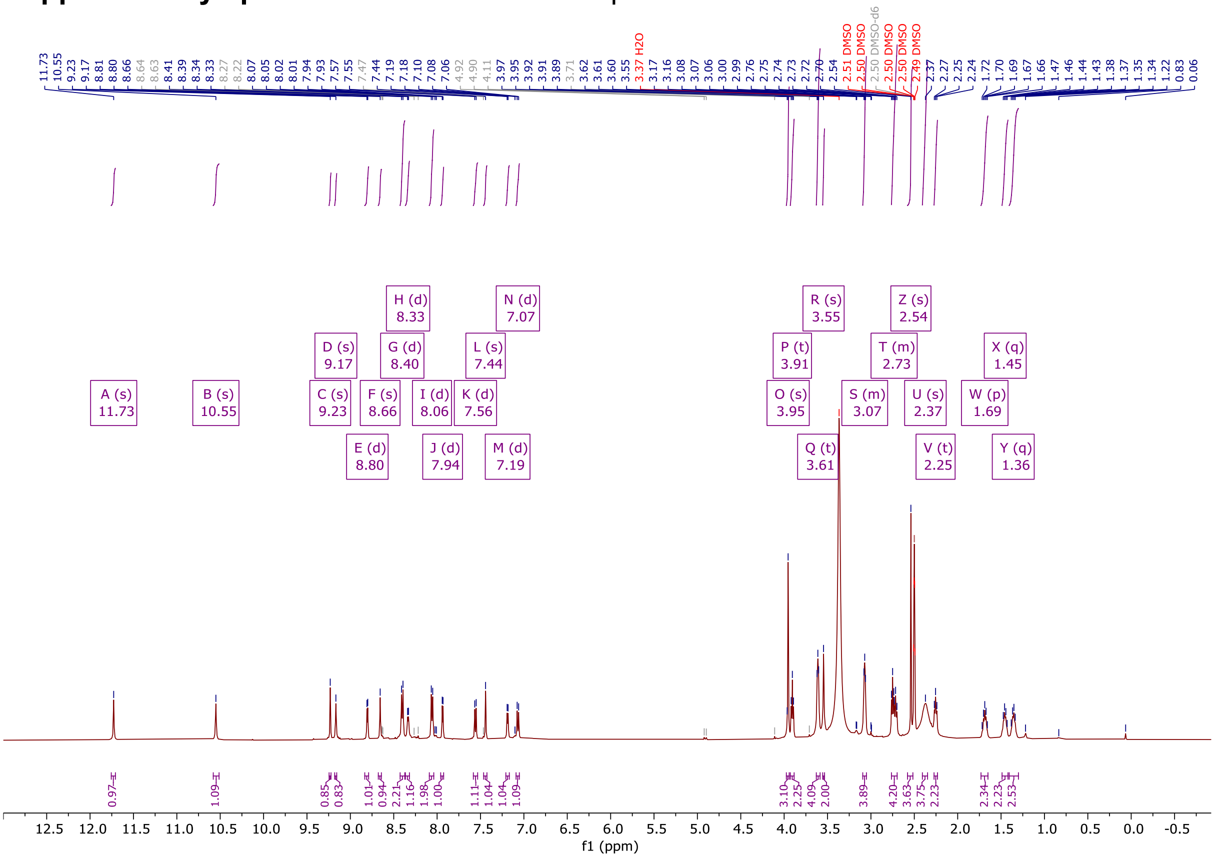

Supplementary spectrum 101: ^{1}H-NMR spectrum of **25**

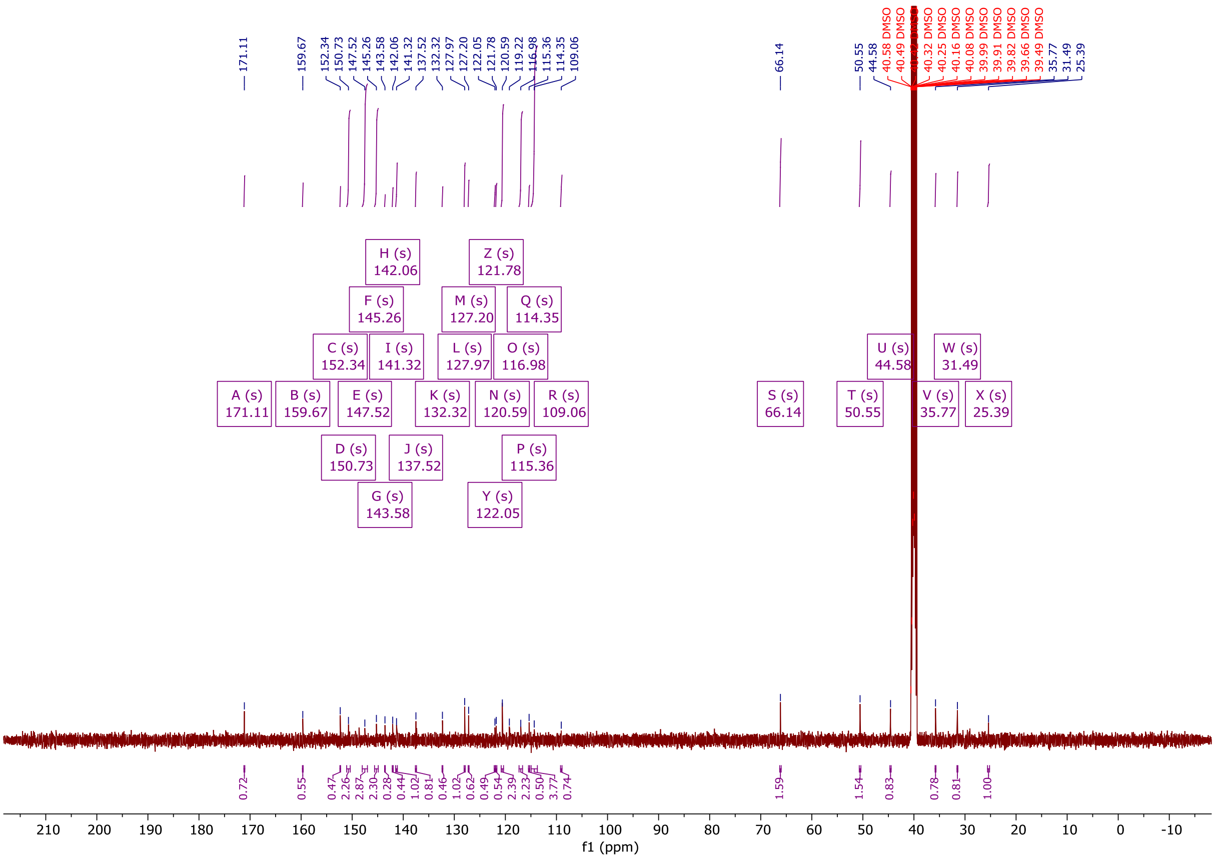

Supplementary spectrum 102: ^{13}C-NMR spectrum of **25**

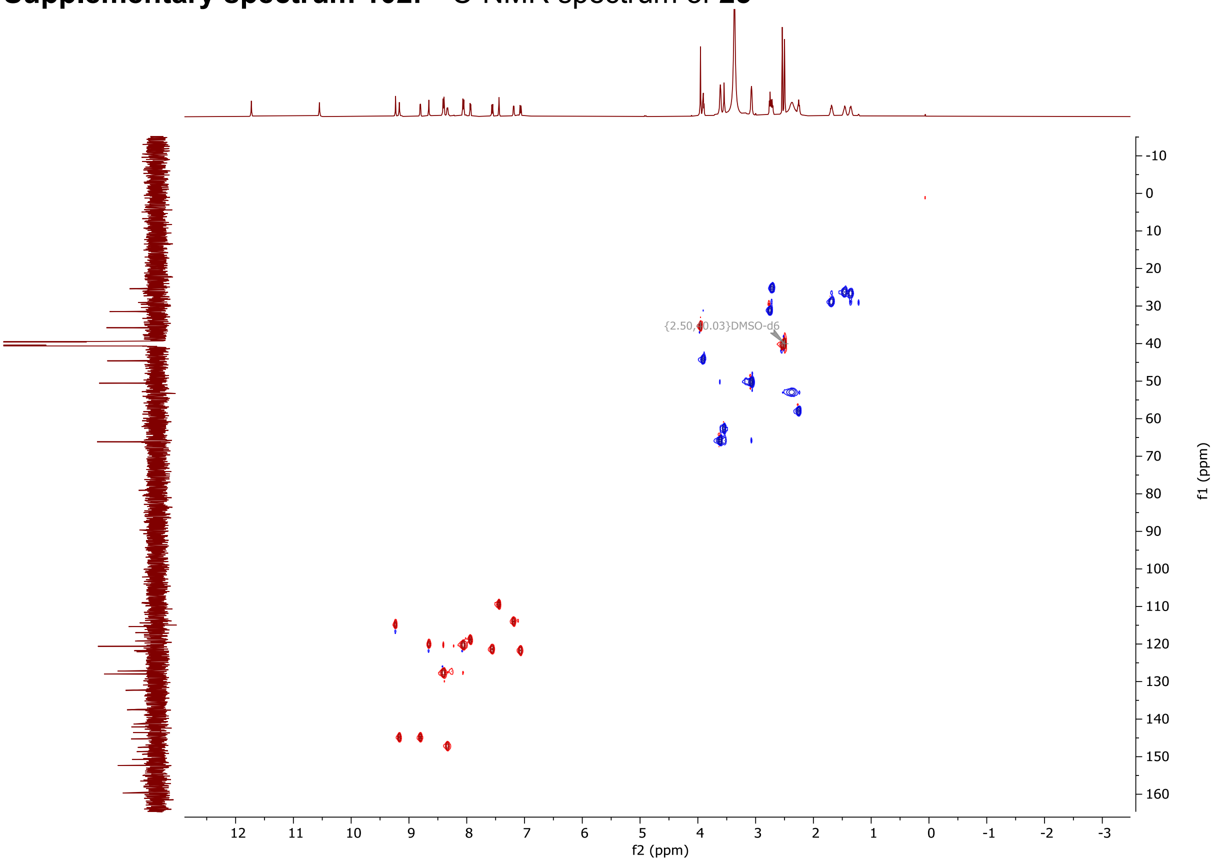

Supplementary spectrum 103: HSQC-NMR spectrum of **25**

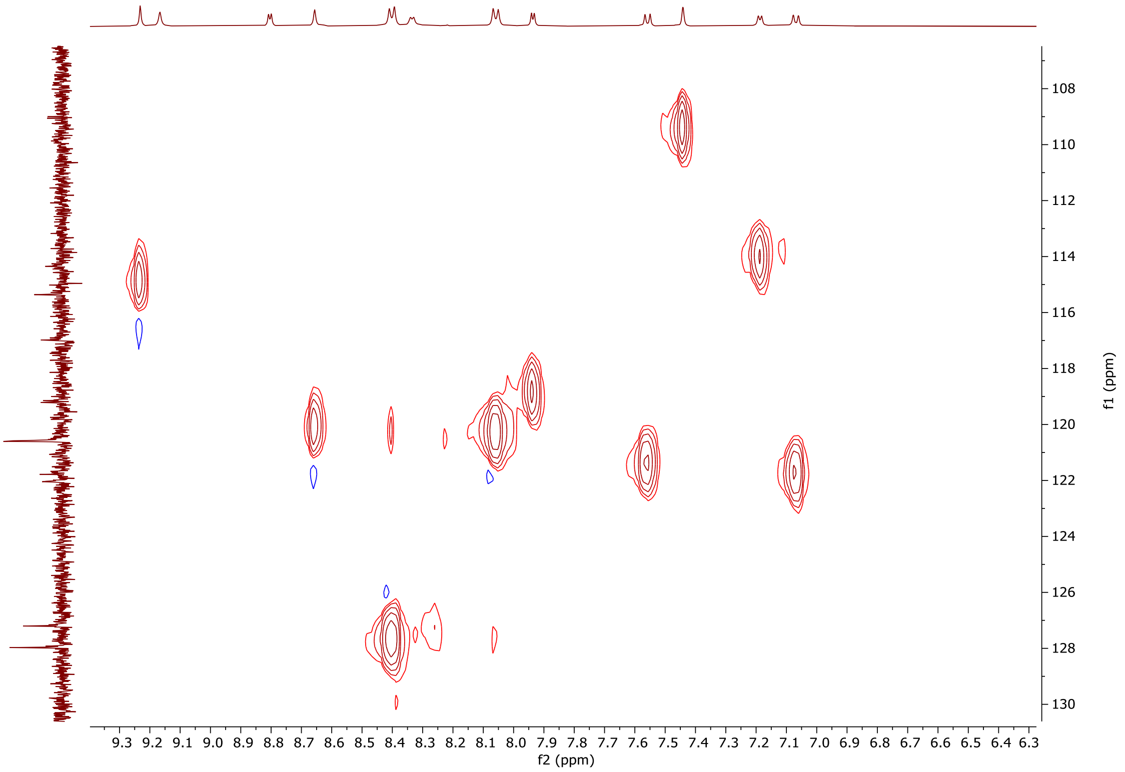

Supplementary spectrum 104: Zoomed in HSQC-NMR spectrum of **25**

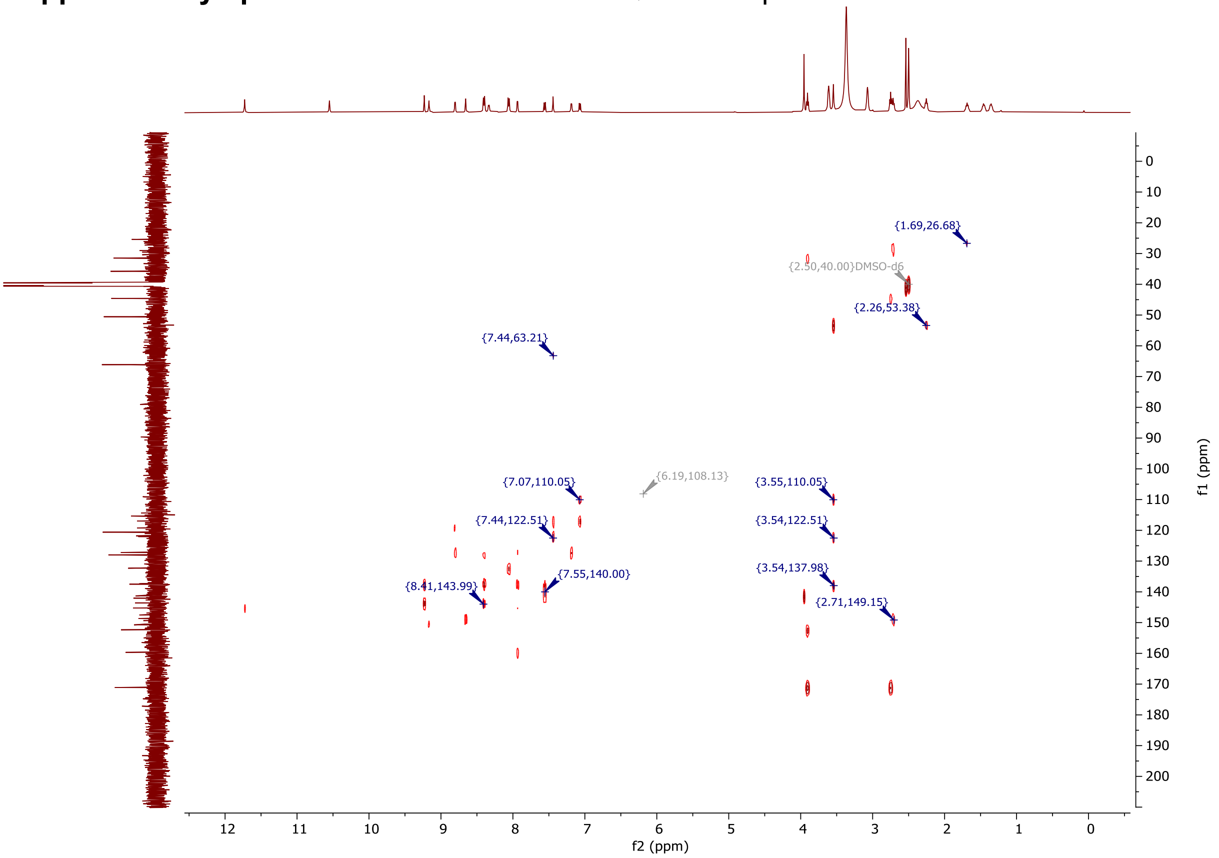

Supplementary spectrum 105: HMBC-NMR spectrum of **25**

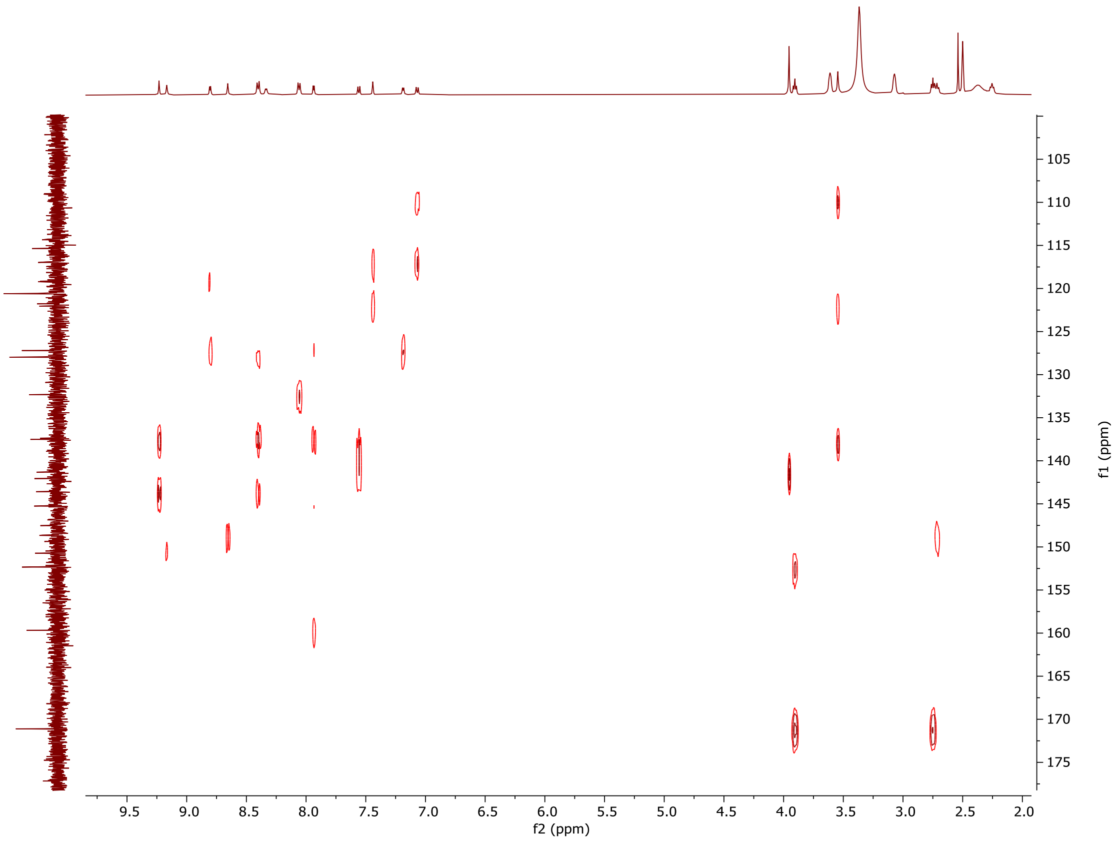

Supplementary spectrum 106: Zoomed in HMBC-NMR spectrum of **25**

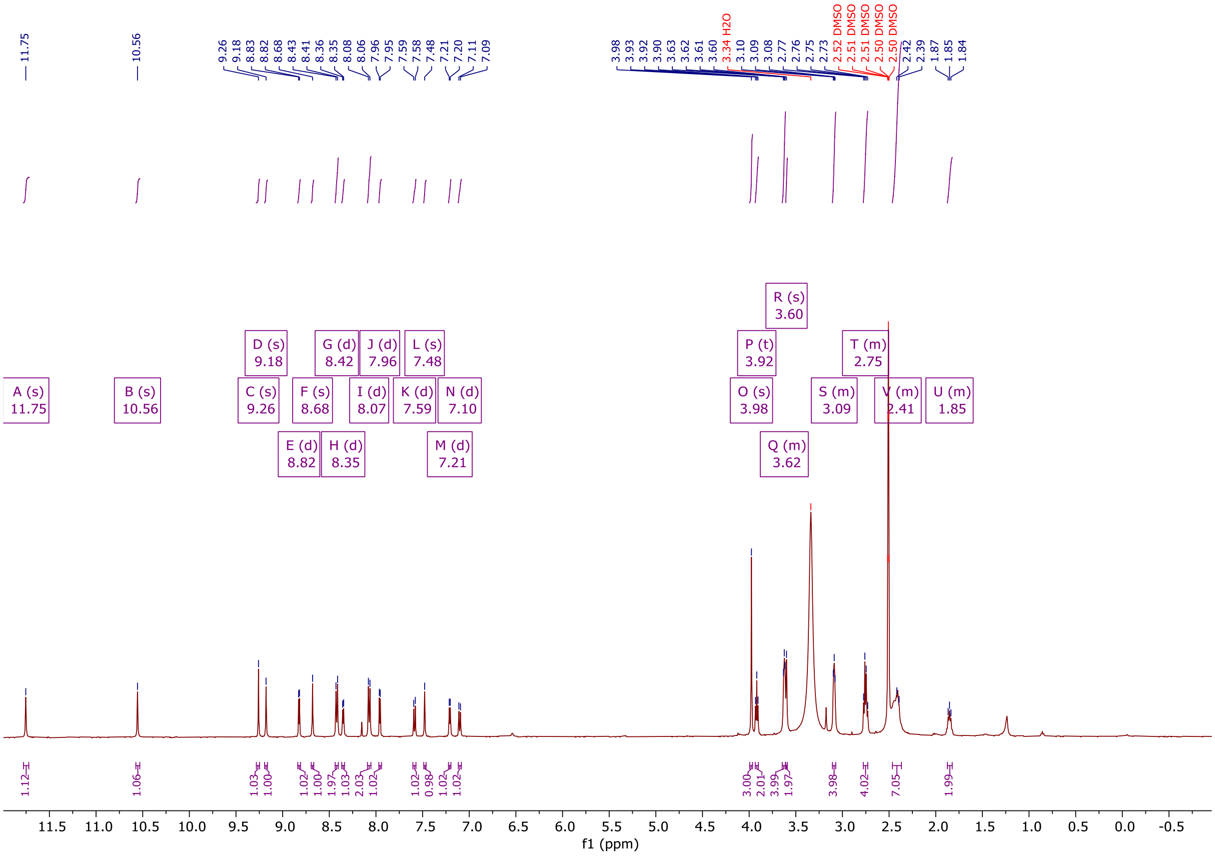

Supplementary spectrum 107: ^{1}H-NMR spectrum of KH2 (**26**)

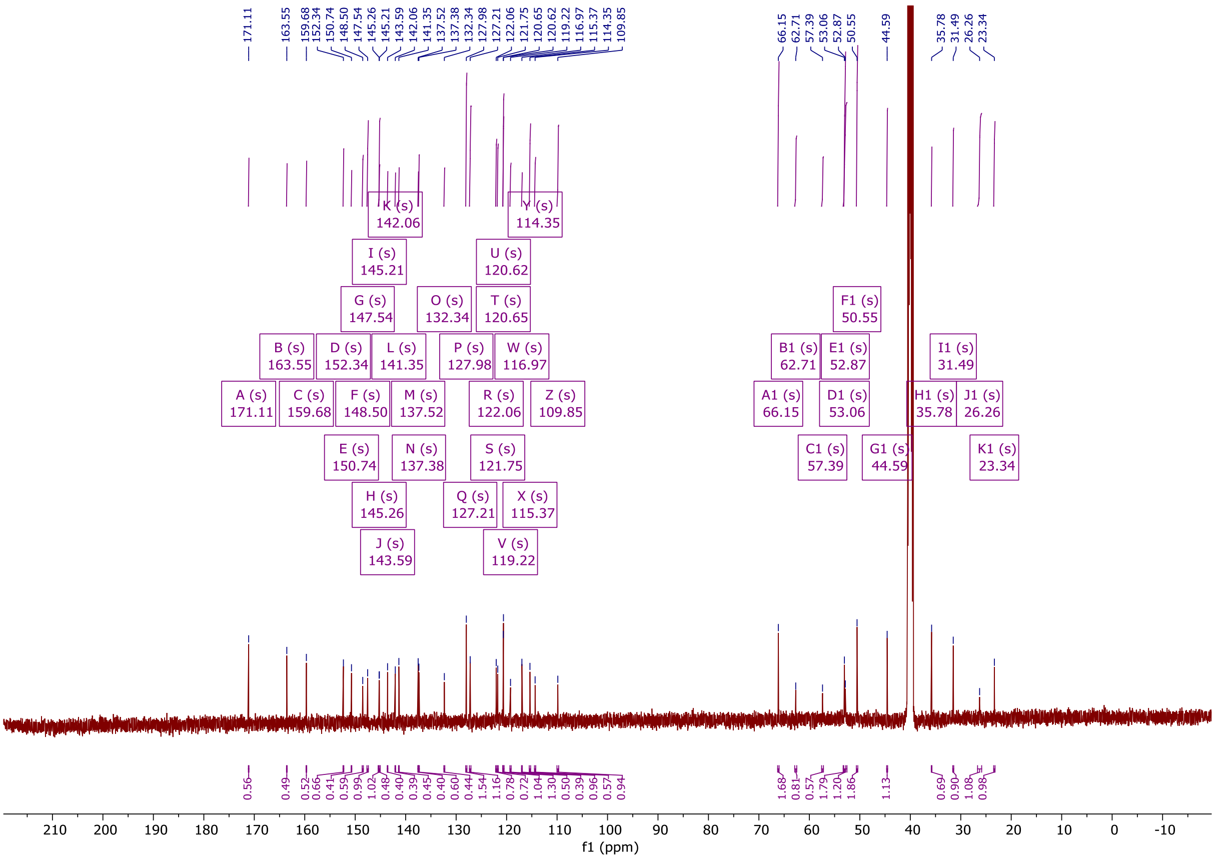

Supplementary spectrum 108: ^{13}C-NMR spectrum of KH2 (**26**)

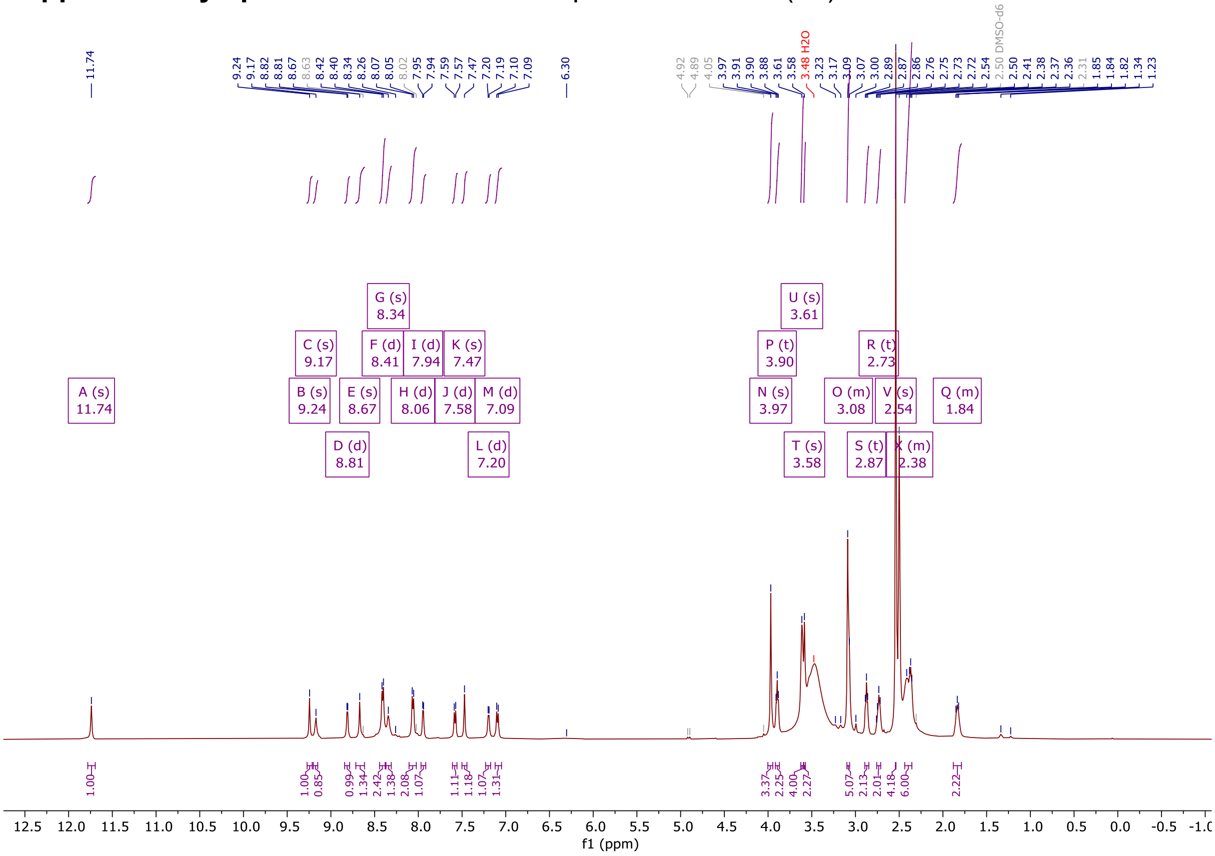

Supplementary spectrum 109: ^{1}H-NMR spectrum of **28**

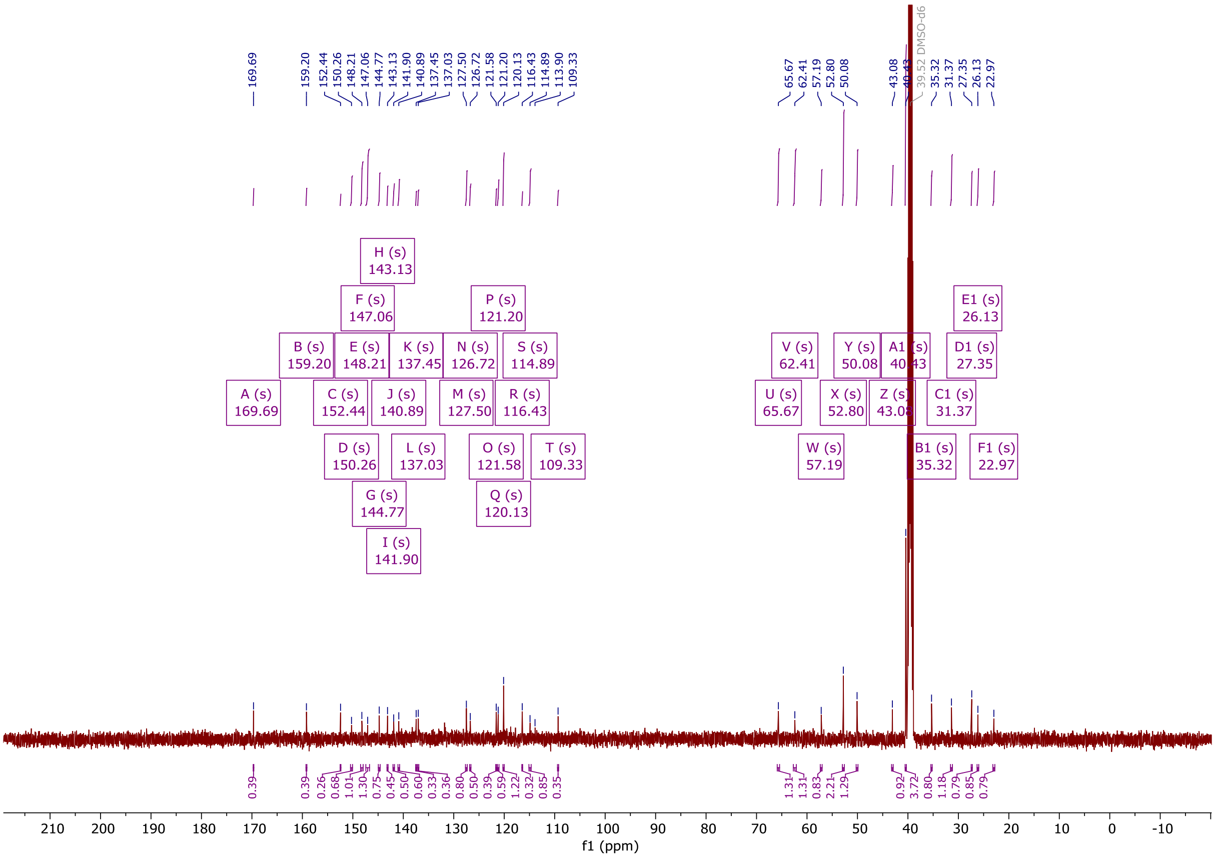

Supplementary spectrum 110: 13C-NMR spectrum of **28**

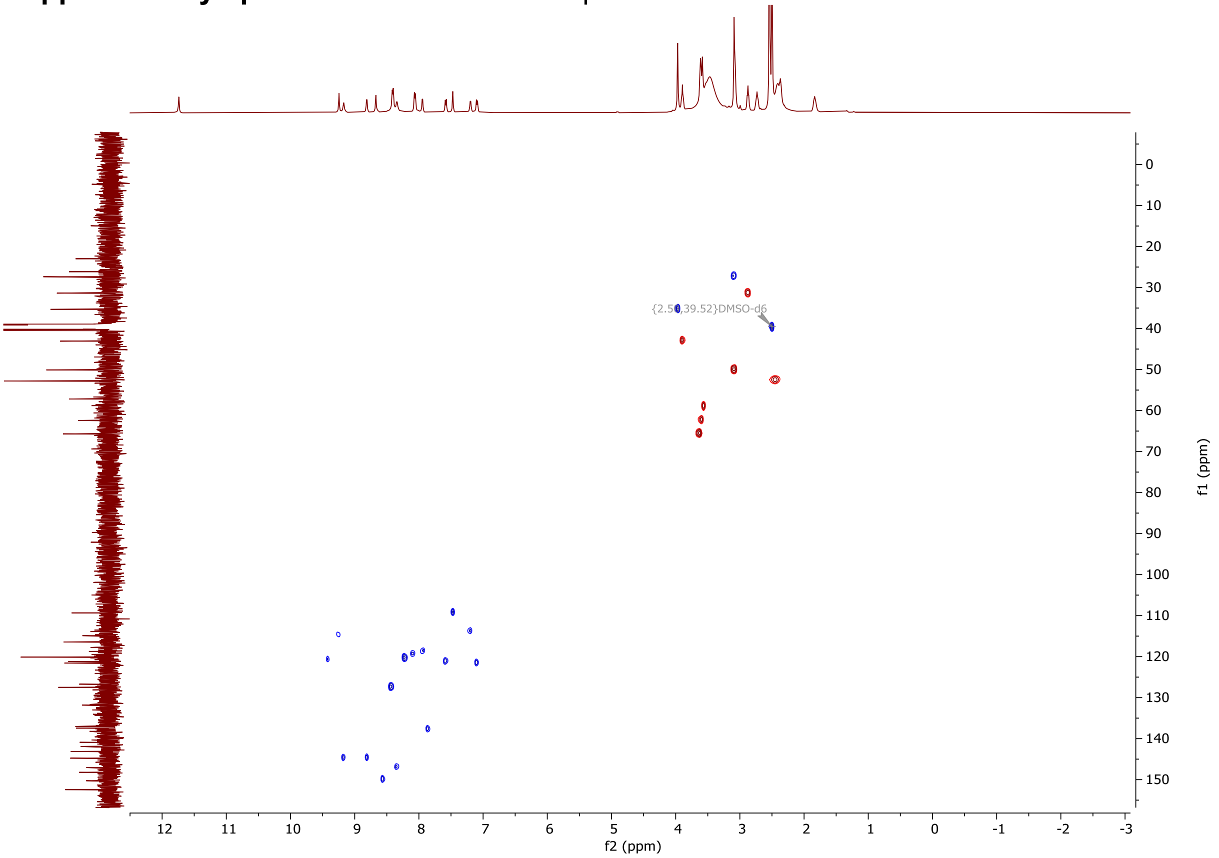

Supplementary spectrum 111: HSQC-NMR spectrum of **28**

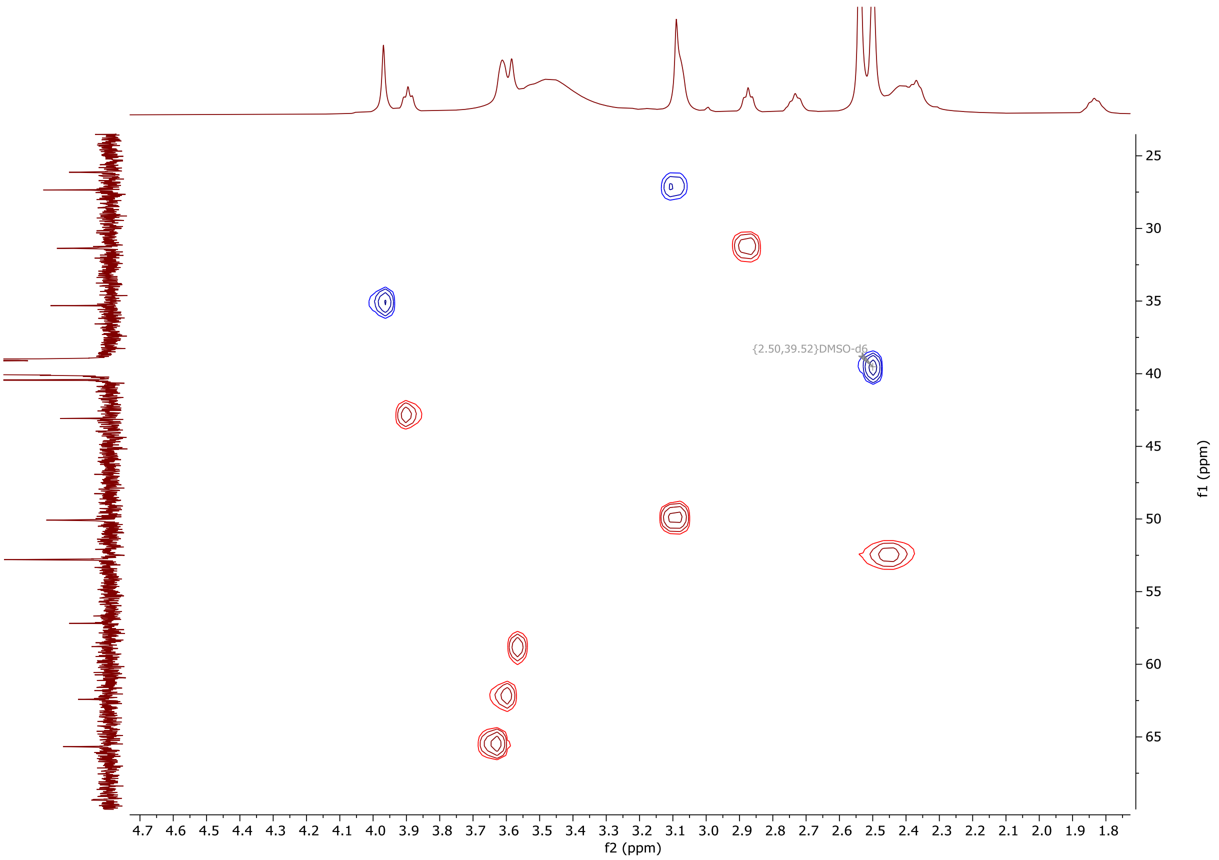

Supplementary spectrum 112: Zoomed in HSQC-NMR spectrum of **28**

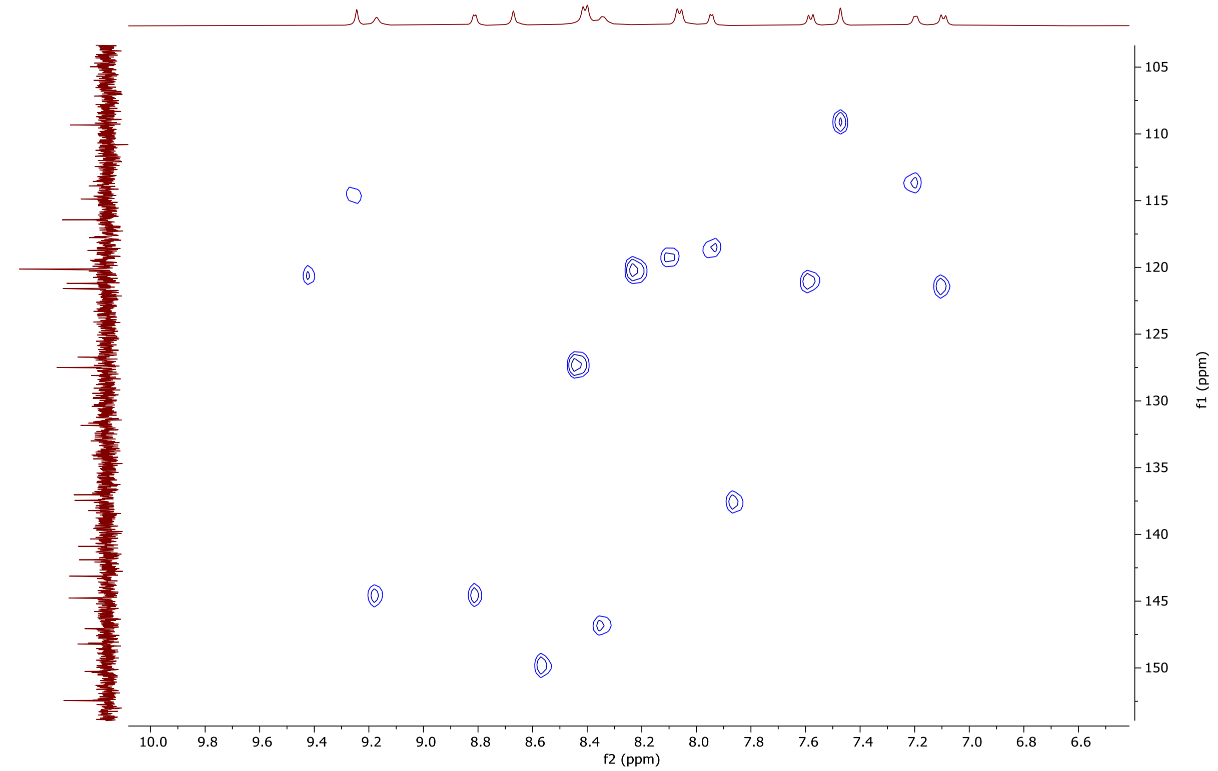

Supplementary spectrum 113: Zoomed in HSQC-NMR spectrum of **28**

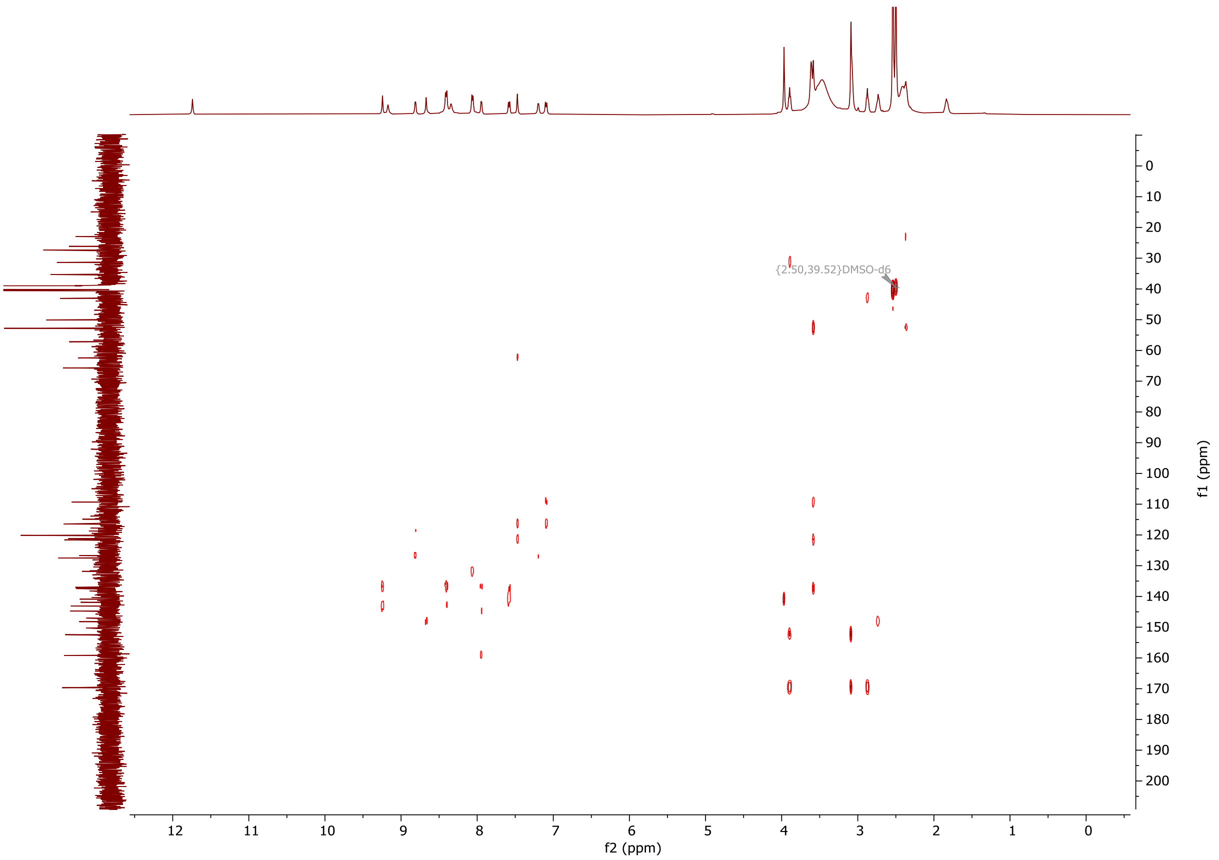

Supplementary spectrum 114: HMBC-NMR spectrum of **28**

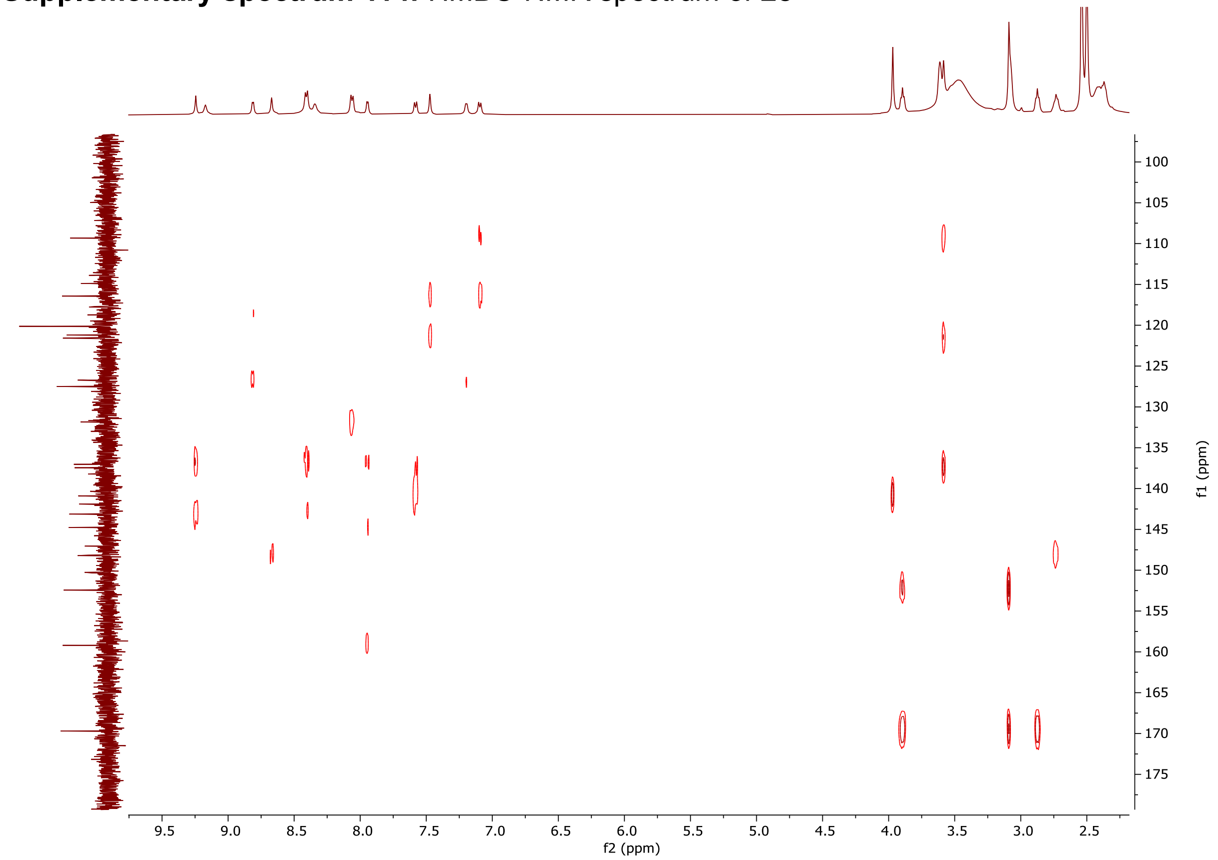

Supplementary spectrum 115: Zoomed in HMBC-NMR spectrum of **28**

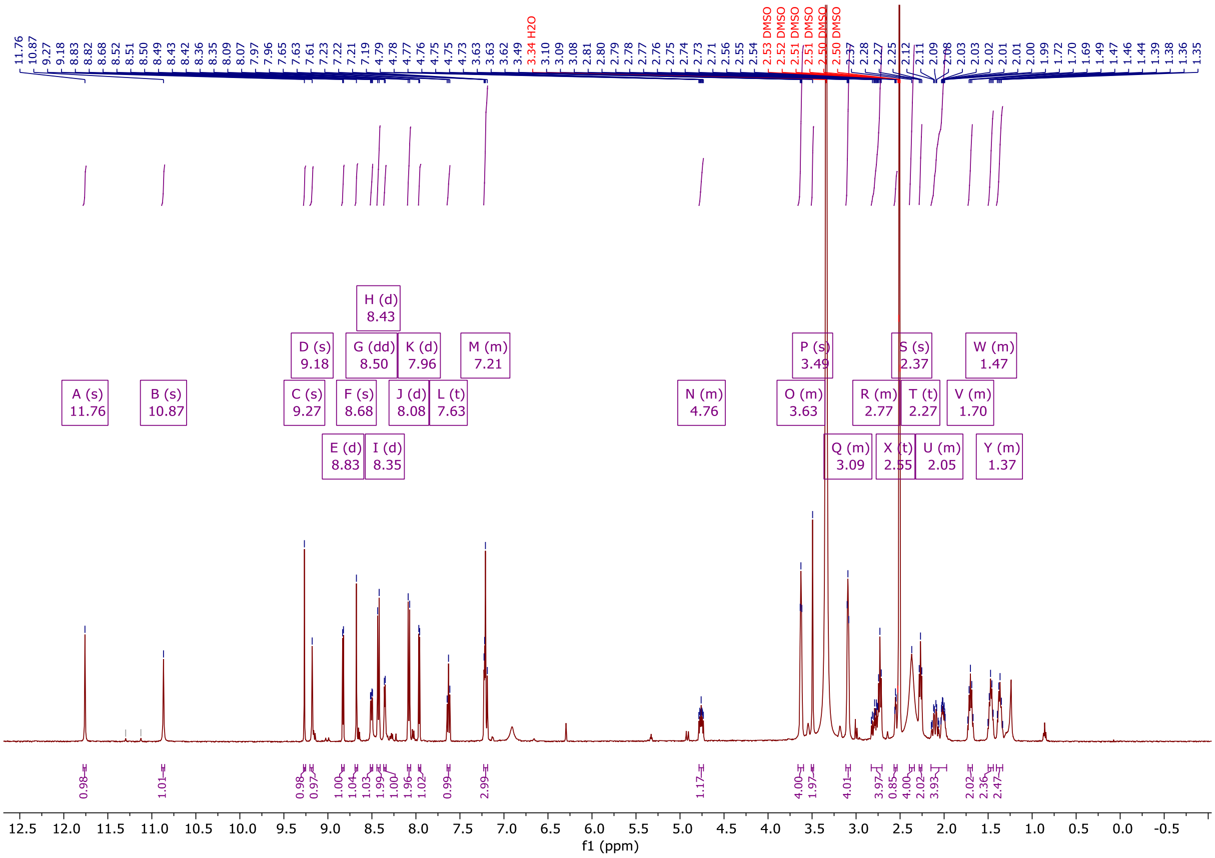

Supplementary spectrum 116: ^{1}H-NMR spectrum of **21**

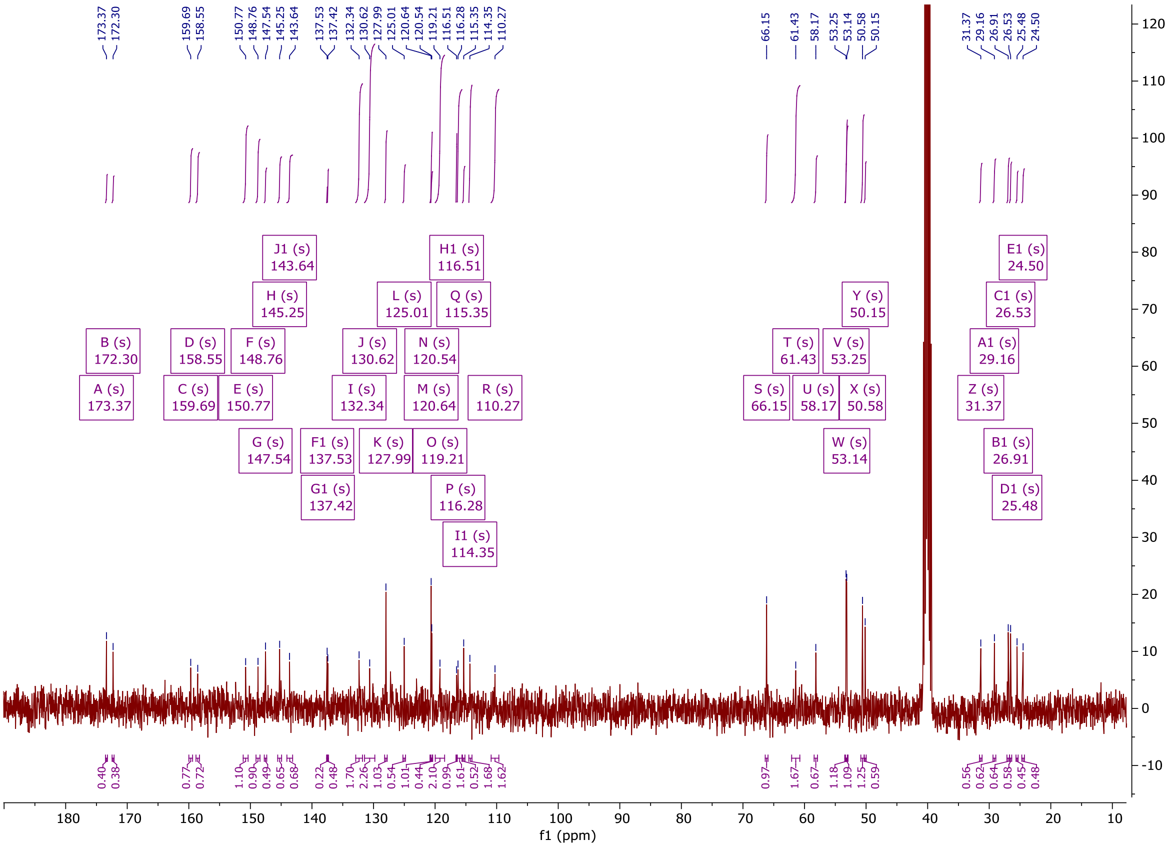

Supplementary spectrum 117: ^{13}C-NMR spectrum of **21**

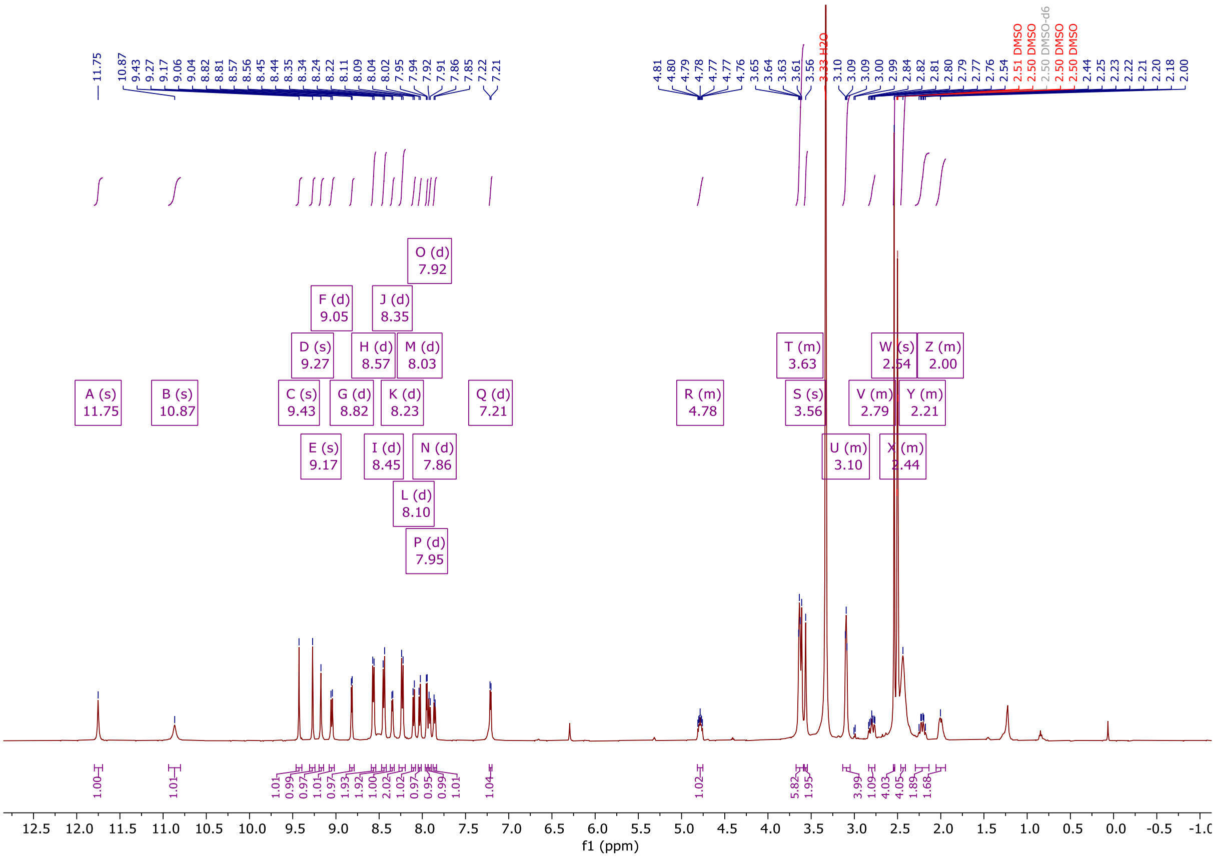

Supplementary spectrum 118: ^{1}H-NMR spectrum of **22**

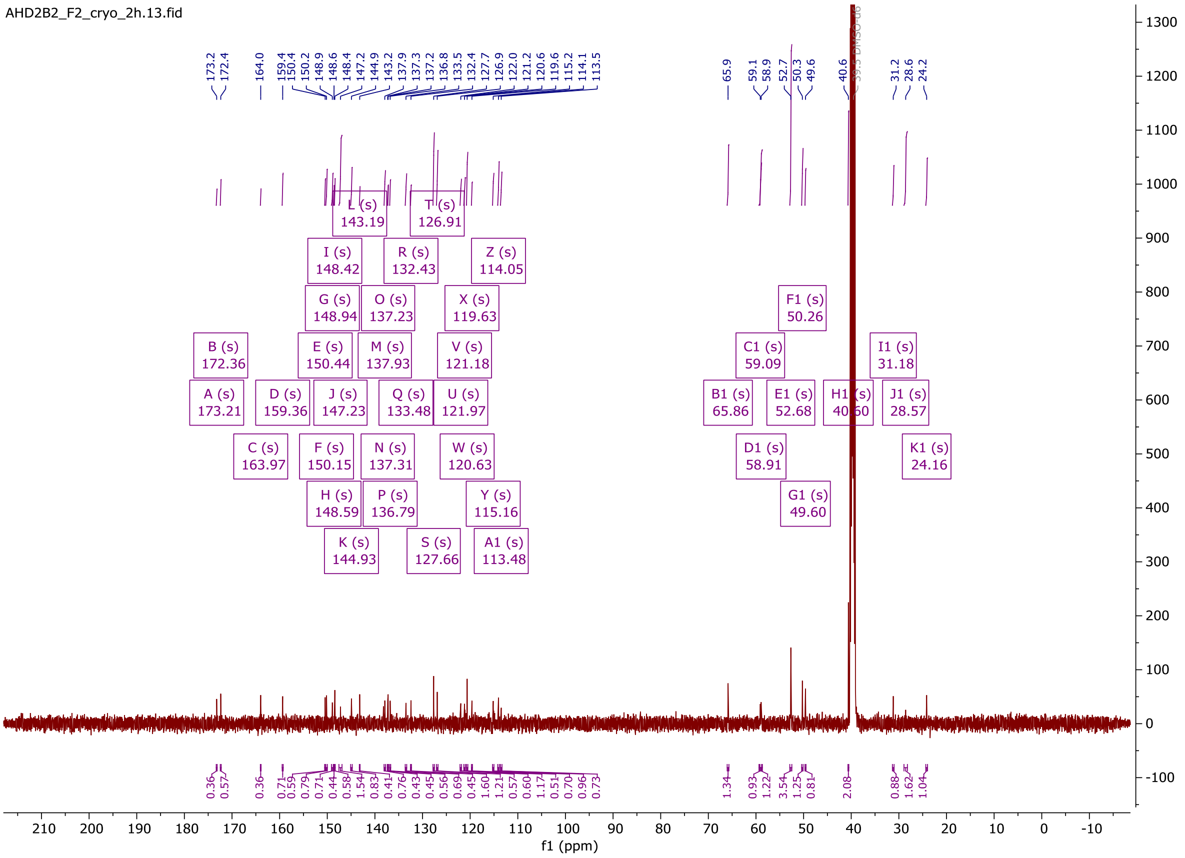

Supplementary spectrum 119: ^{13}C-NMR spectrum of **22**

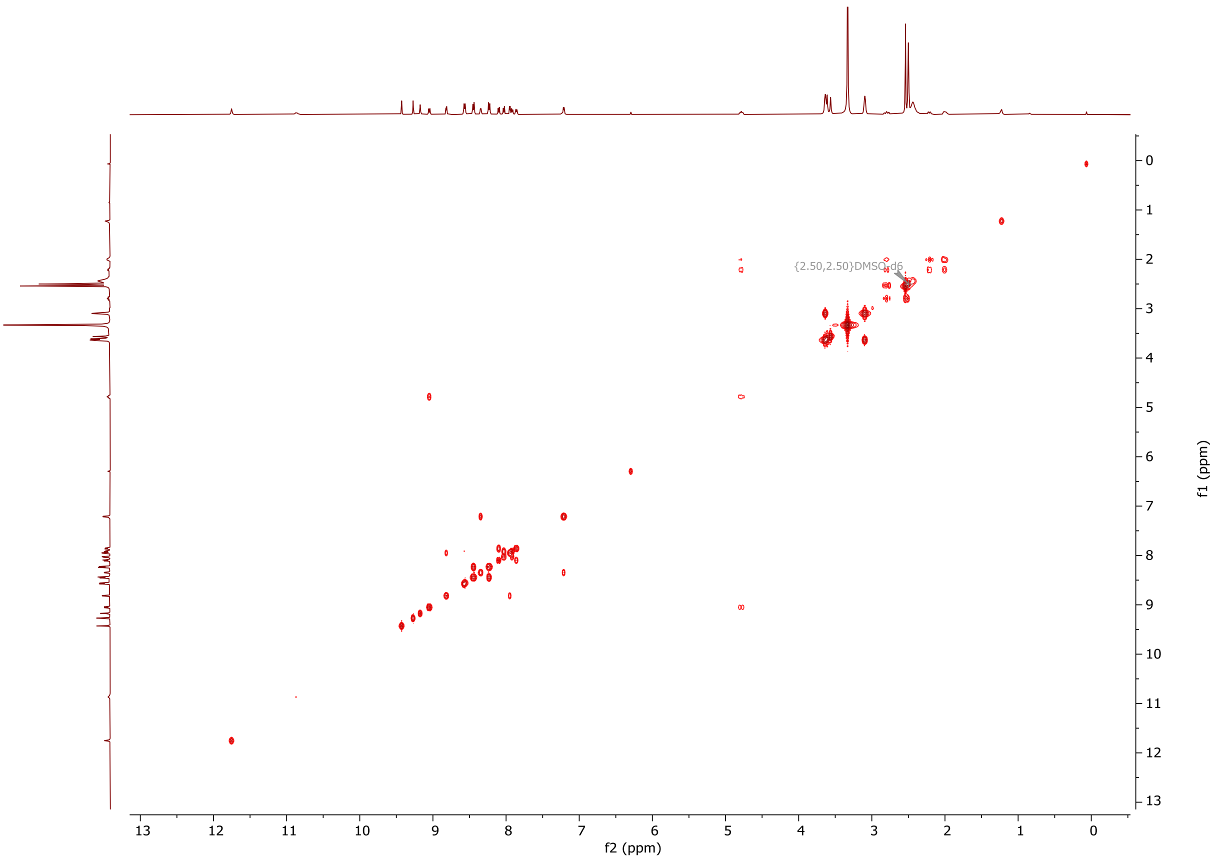

Supplementary spectrum 120: COSY-NMR spectrum of **22**

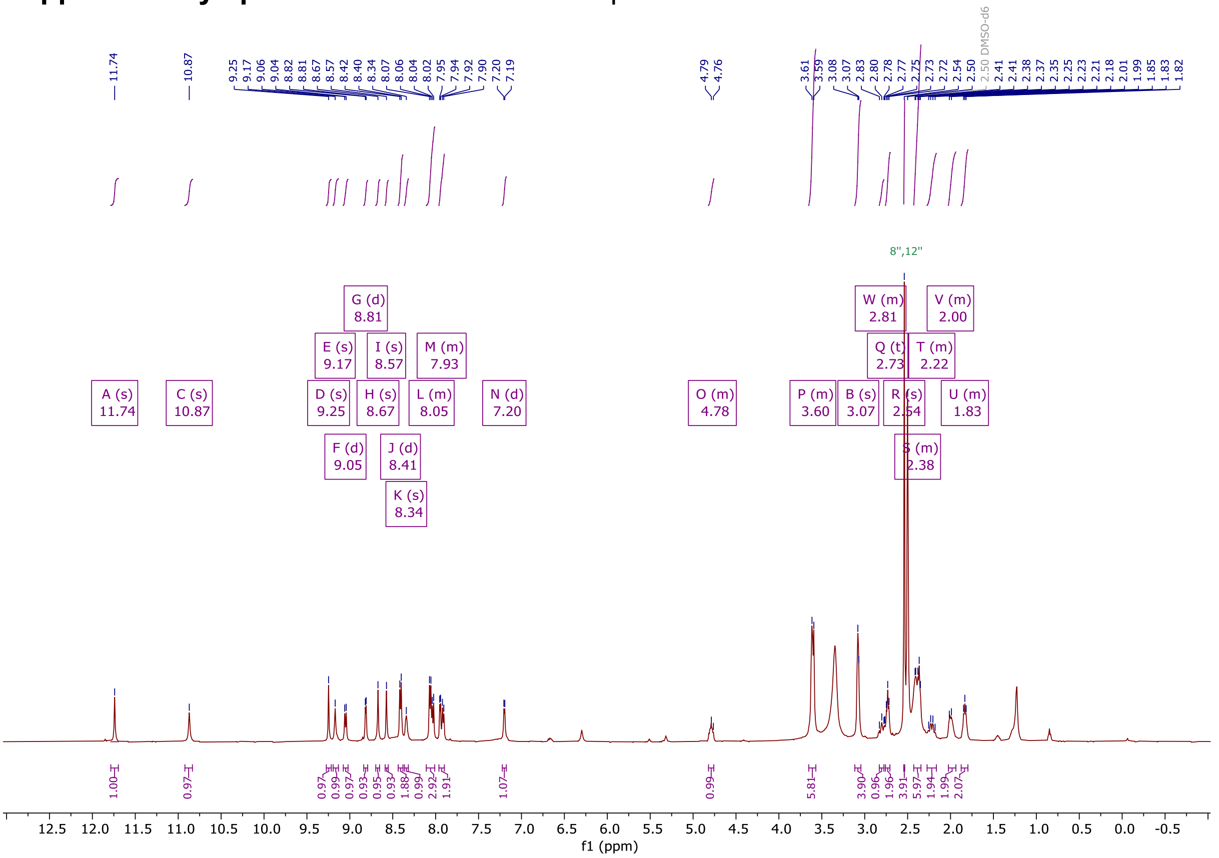

Supplementary spectrum 121: ^{1}H-NMR spectrum of **23**

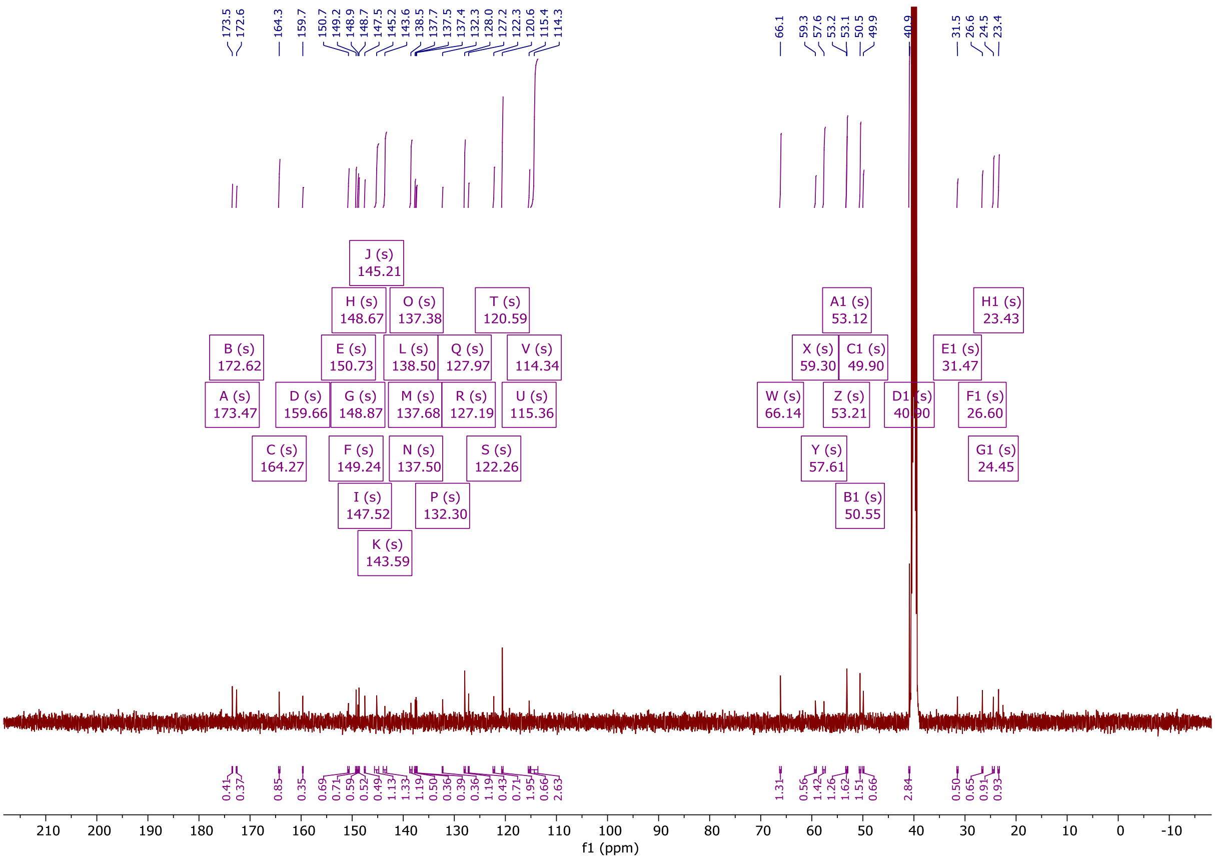

Supplementary spectrum 122: ^{13}C-NMR spectrum of **23**

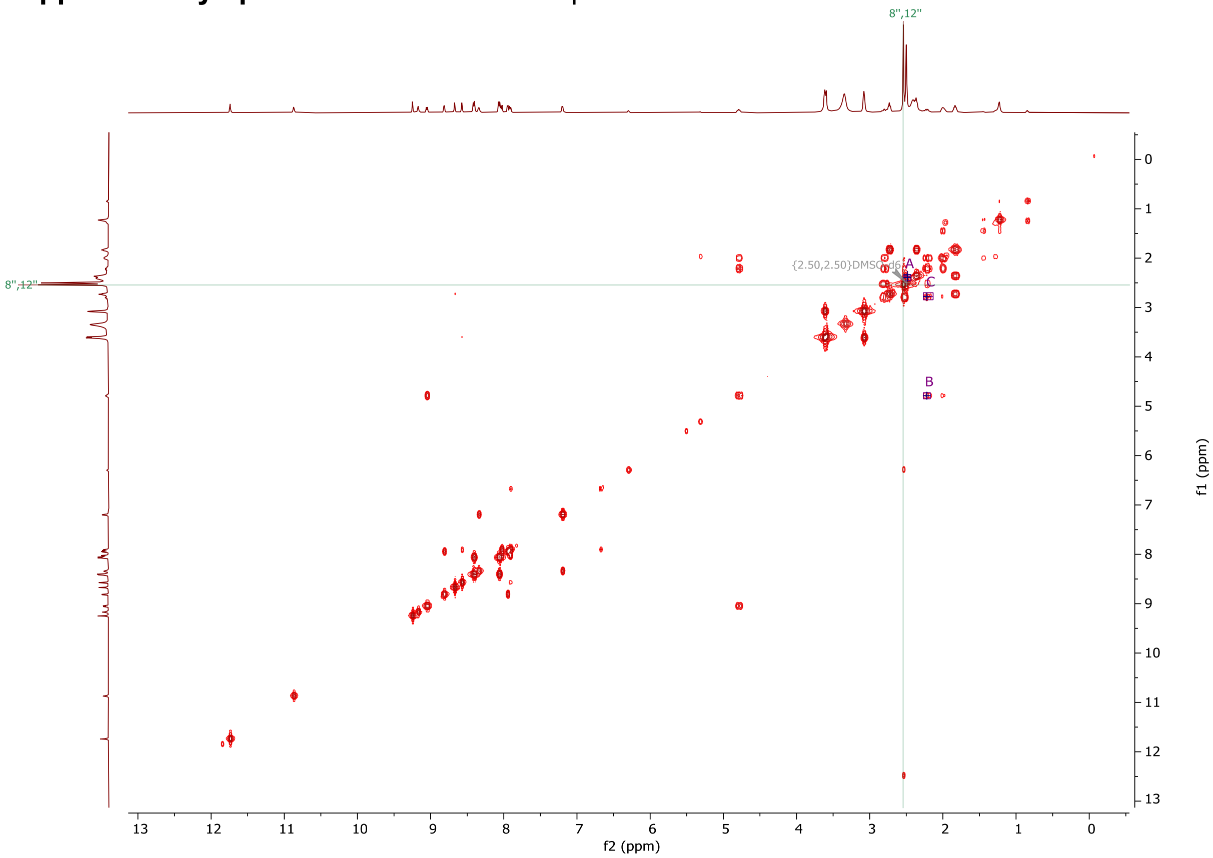

Supplementary spectrum 123: COSY-NMR spectrum of **23**
