# Supplementary material for: Discovery of a CNS active GSK3 degrader using orthogonally reactive linker screening
Source: Nat Commun. 2025 Oct 6;16:8857. doi: 10.1038/s41467-025-63928-8 (PMC12501029; doi:10.1038/s41467-025-63928-8)
Supplement: Supplementary file 2 — Description of Additional Supplementary Information [file 41467_2025_63928_MOESM2_ESM.pdf]

Discovery of a CNS active GSK3 degrader using orthogonally reactive linker screening

Andreas Holmqvist [1$], Nur Mehpare Kocaturk[1$], Christina Duncan[2], Jennifer Riley[2], Steven Baginski[2], Graham Marsh[3], Joel Cresser-Brown[3], Hannah Maple[3], Kristiina Juvonen[1], Gajanan Sathe[1], Nicola Morrice[4], Calum Sutherland[5], Kevin D. Read[2] and William Farnaby[1*]

1. Centre for Targeted Protein Degradation, School of Life Sciences, University of Dundee, Dundee, DD1 5JJ, U.K.
2. Wellcome Centre for Anti-Infective Research, Drug Discovery Unit, Division of Biological Chemistry and Drug Discovery, School of Life Sciences, University of Dundee, DD1 5EH, U.K.
3. Bio-Techne (Tocris), The Watkins Building, Atlantic Road, Avonmouth, Bristol BS11 9QD, U.K.
4. Division of Neuroscience, School of Medicine, IMS/ WTB Complex, University of Dundee, Dundee, DD1 5EH, UK
5. School of Medicine, University of Dundee, Ninewells Hospital & Medical School DD1 9SY, U.K.

[$]These authors contributed equally

[*]Corresponding author – for enquiries please contact w.farnaby@dundee.ac.uk

Description of additional supplementary data

Supplementary Data 1. Total Proteomics for KH1 with 2 hours treatment time in HEK293 cells.

Supplementary Data 2. Total Proteomics for KH2 with 2 hours treatment time in HEK293 cells.

Supplementary Data 3. Total Proteomics for KH1 with 4 hours treatment time in HEK293 cells.

Supplementary Data 4. Total Proteomics for KH1 with 24 hours treatment time in HEK293 cells.

Supplementary Data 5. Phosphoproteomics for KH1 with 2 hours treatment time in HEK293 cells.

Supplementary Data 6. Phosphoproteomics for KH1 with 4 hours treatment time in HEK293 cells.

Supplementary Data 7. Phosphoproteomics for KH1 with 24 hours treatment time in HEK293 cells.

Supplementary Data 8. Total Proteomics for KH1 from liver tissue samples.

Supplementary Data 9. Phosphoproteomics for KH1 from liver tissue samples.

Supplementary Data 10. Certification of cell line authentication tests.
