# Supplementary material for: Discovery of a CNS active GSK3 degrader using orthogonally reactive linker screening
Source: Nat Commun. 2025 Oct 6;16:8857. doi: 10.1038/s41467-025-63928-8 (PMC12501029; doi:10.1038/s41467-025-63928-8)
Supplement: Supplementary file 12 — Supplementary Dataset 10 [file 41467_2025_63928_MOESM12_ESM.pdf]

Eurofins Genomics Europe Food/Environment/White Biotech Products & Services GmbH, Anzinger Str. 7 a, D-85560 Ebersberg

Dr. Nur Kocaturk
University of Dundee, School of Lifesciences
1 James Lindsay Place
DD1 5JJ Dundee
Great Britain

eurofins | Genomics

SOP_APG_Zelllinienauthentizität_A04_3

Person in charge:	Dr. Torsten Brendel
Report date:	14.04.2025
Sample received on:	07.04.2025
Start / End of Analysis:	07.04.2025/14.04.2025

Analytical Report:
Cell Line Authentication Test
Order ID: 11109312168

Method:
DNA isolation was carried out from cell pellet (cell layer).
Genetic characteristics were determined by PCR-single-locus-technology.
16 independent PCR-systems D8S1179, D21S11, D7S820, CSF1PO, D3S1358, TH01, D13S317, D16S539, D2S1338, AMEL, D5S818, FGA, D19S433, vWA, TPOX and D18S51 were investigated.
(ASN-0002 core markers are colored grey, Thermo Fisher, AmpFlSTR® Identifiler® Plus PCR Amplification Kit)
In parallel, positive and negative controls were carried out yielding correct results.
Method details are given ir**SOP_APG_Zelllinienauthentizität_3.(**_15.04.2024

Result:

**Client Sample Name**	GSK3b-HiBiTcl.F1 HEK293
**Sample Code**	CL00022624
D8S1179	12,14
D21S11	28,30.2
D7S820	11,12
CSF1PO	12,12
D3S1358	15,17
TH01	9.3,9.3
D13S317	12,14
D16S539	9,13
D2S1338	19,19
D19S433	15,18
vWA	16,19
TPOX	11,11
D18S51	17,18
AMEL	X,X
D5S818	8,8
FGA	23,23
**Database Name**	94 % identity: Several HEK cell lines were identified

The table shows the result of the cell line analysis and the comparison with the online database of the DSMZ (http://www.dsmz.de/de/service/services-human-and-animal-cell) and the Cellosaurus database (https://web.expasy.org/cellosaurus). Please note that only the PCR-systems according to ANSI/ATCC standard ASN-0002 were aligned (D5S818, D13S317, D7S820, D16S539, VWA, TH01, TPOX, CSF1PO, AMEL - colored grey).

This report was created automatically and is therefore valid without a signature.

The laboratory is accredited acc. to **DIN EN ISO/IEC 17025:2018**. All analyses have been carried out with greatest care and on the basis of state of the art scientific knowledge. The results refer solely to the analysed samples, as received. The duplication and publication also in parts requires a written authorization by this laboratory. Our General Terms and Conditions apply exclusively and are available under eurofinsgenomics.com

Eurofins Genomics Europe Food/Environment/White Biotech Products & Services GmbH, Anzinger Str. 7 a, D-85560 Ebersberg

eurofins | Genomics

SOP_APG_Zelllinienauthentizität_A04_3

Dr. Nur Kocaturk
University of Dundee, School of Lifesciences
1 James Lindsay Place
DD1 5JJ Dundee
Great Britain

Person in charge: Dr. Torsten Brendel
Report date: 14.04.2025
Sample received on: 07.04.2025
Start / End of Analysis: 07.04.2025/14.04.2025

Analytical Report:
Cell Line Authentication Test
Order ID: 11109312168

Method:
DNA isolation was carried out from cell pellet (cell layer).
Genetic characteristics were determined by PCR-single-locus-technology.
16 independent PCR-systems D8S1179, D21S11, D7S820, CSF1PO, D3S1358, TH01, D13S317, D16S539, D2S1338, AMEL, D5S818, FGA, D19S433, vWA, TPOX and D18S51 were investigated.
(ASN-0002 core markers are colored grey, Thermo Fisher, AmpFlSTR® Identifiler® Plus PCR Amplification Kit)
In parallel, positive and negative controls were carried out yielding correct results.
Method details are given ir **SOP_APG_Zelllinienauthentizität_3.(**_15.04.2024

Result:

**Client Sample Name**	HAP1 CRBN KO
**Sample Code**	CL00022622
D8S1179	8,8
D21S11	30,30
D7S820	9,9
CSF1PO	10,10
D3S1358	15,15
TH01	7,7
D13S317	11,11
D16S539	11,11
D2S1338	25,25
D19S433	15,15
vWA	18,18
TPOX	8,8
D18S51	18,18
AMEL	X,X
D5S818	12,12
FGA	21,21
**Database Name**	HAP1

The table shows the result of the cell line analysis and the comparison with the online database of the DSMZ (http://www.dsmz.de/de/service/services-human-and-animal-cell) and the Cellosaurus database (https://web.expasy.org/cellosaurus). Please note that only the PCR-systems according to ANSI/ATCC standard ASN-0002 were aligned (D5S818, D13S317, D7S820, D16S539, VWA, TH01, TPOX, CSF1PO, AMEL - colored grey).

This report was created automatically and is therefore valid without a signature.

The laboratory is accredited acc. to **DIN EN ISO/IEC 17025:2018**. All analyses have been carried out with greatest care and on the basis of state of the art scientific knowledge. The results refer solely to the analysed samples, as received. The duplication and publication also in parts requires a written authorization by this laboratory. Our General Terms and Conditions apply exclusively and are available under eurofinsgenomics.com

DAkkS Deutsche Akkreditierungsstelle D-PL-13372-01-00

Eurofins Genomics Europe Food/Environment/White Biotech Products & Services GmbH, Anzinger Str. 7 a, D-85560 Ebersberg

Dr. Nur Kocaturk
University of Dundee, School of Lifesciences
1 James Lindsay Place
DD1 5JJ Dundee
Great Britain

eurofins | Genomics

SOP_APG_Zelllinienauthentizität_A04_3.(

Person in charge: Dr. Torsten Brendel
Report date: 14.04.2025
Sample received on: 07.04.2025
Start / End of Analysis: 07.04.2025/14.04.2025

Analytical Report:
Cell Line Authentication Test
Order ID: 11109312168

Method:
DNA isolation was carried out from cell pellet (cell layer).
Genetic characteristics were determined by PCR-single-locus-technology.
16 independent PCR-systems D8S1179, D21S11, D7S820, CSF1PO, D3S1358, TH01, D13S317, D16S539, D2S1338, AMEL, D5S818, FGA, D19S433, vWA, TPOX and D18S51 were investigated.
(ASN-0002 core markers are colored grey, Thermo Fisher, AmpFlSTR® Identifiler® Plus PCR Amplification Kit)
In parallel, positive and negative controls were carried out yielding correct results.
Method details are given in **SOP_APG_Zelllinienauthentizität_3.(**_15.04.2024

Result:

**Client Sample Name**	HAP1 WT
**Sample Code**	CL00022627
D8S1179	8,8
D21S11	30,30
D7S820	9,9
CSF1PO	10,10
D3S1358	15,15
TH01	7,7
D13S317	11,11
D16S539	11,11
D2S1338	25,25
D19S433	15,15
vWA	18,18
TPOX	8,8
D18S51	18,18
AMEL	X,X
D5S818	12,12
FGA	21,21
**Database Name**	HAP1

The table shows the result of the cell line analysis and the comparison with the online database of the DSMZ (http://www.dsmz.de/de/service/services-human-and-animal-cell) and the Cellosaurus database (https://web.expasy.org/cellosaurus). Please note that only the PCR-systems according to ANSI/ATCC standard ASN-0002 were aligned (D5S818, D13S317, D7S820, D16S539, VWA, TH01, TPOX, CSF1PO, AMEL - colored grey).

This report was created automatically and is therefore valid without a signature.

The laboratory is accredited acc. to DIN EN ISO/IEC 17025:2018. All analyses have been carried out with greatest care and on the basis of state of the art scientific knowledge. The results refer solely to the analysed samples, as received. The duplication and publication also in parts requires a written authorization by this laboratory. Our General Terms and Conditions apply exclusively and are available under eurofinsgenomics.com

Eurofins Genomics Europe Food/Environment/White Biotech Products & Services GmbH, Anzinger Str. 7 a, D-85560 Ebersberg

Dr. Nur Kocaturk
University of Dundee, School of Lifesciences
1 James Lindsay Place
DD1 5JJ Dundee
Great Britain

eurofins | Genomics

SOP_APG_Zelllinienauthentizität_A04_3

Person in charge: Dr. Torsten Brendel
Report date: 14.04.2025
Sample received on: 07.04.2025
Start / End of Analysis: 07.04.2025/14.04.2025

Analytical Report:
Cell Line Authentication Test
Order ID: 11109312168

Method:
DNA isolation was carried out from cell pellet (cell layer).
Genetic characteristics were determined by PCR-single-locus-technology.
16 independent PCR-systems D8S1179, D21S11, D7S820, CSF1PO, D3S1358, TH01, D13S317, D16S539, D2S1338, AMEL, D5S818, FGA, D19S433, vWA, TPOX and D18S51 were investigated.
(ASN-0002 core markers are colored grey, Thermo Fisher, AmpFlSTR® Identifiler® Plus PCR Amplification Kit)
In parallel, positive and negative controls were carried out yielding correct results.
Method details are given ir**SOP_APG_Zelllinienauthentizität_3.(**_15.04.2024

Result:

**Client Sample Name**	HEK293
**Sample Code**	CL00022623
D8S1179	12,14
D21S11	28,30.2
D7S820	11,12
CSF1PO	12,12
D3S1358	15,17
TH01	7,9.3
D13S317	12,14
D16S539	9,13
D2S1338	19,19
D19S433	15,18
vWA	16,19
TPOX	11,11
D18S51	17,18
AMEL	X,X
D5S818	8,8
FGA	23,23
**Database Name**	Several HEK cell lines were identified

The table shows the result of the cell line analysis and the comparison with the online database of the DSMZ (http://www.dsmz.de/de/service/services-human-and-animal-cell) and the Cellosaurus database (https://web.expasy.org/cellosaurus). Please note that only the PCR-systems according to ANSI/ATCC standard ASN-0002 were aligned (D5S818, D13S317, D7S820, D16S539, VWA, TH01, TPOX, CSF1PO, AMEL - colored grey).

This report was created automatically and is therefore valid without a signature.

The laboratory is accredited acc. to DIN EN ISO/IEC 17025:2018. All analyses have been carried out with greatest care and on the basis of state of the art scientific knowledge. The results refer solely to the analysed samples, as received. The duplication and publication also in parts requires a written authorization by this laboratory. Our General Terms and Conditions apply exclusively and are available under eurofinsgenomics.com

DAkkS Deutsche Akkreditierungsstelle D-PL-13372-01-00

Eurofins Genomics Europe Food/Environment/White Biotech Products & Services GmbH, Anzinger Str. 7 a, D-85560 Ebersberg

Nur Kocaturk
University of Dundee, CeTPD
1 James Lindsay place
DD1 5JJ Dundee

United Kingdom

Person in charge: Dr. Torsten Brendel
Report date: 11.04.2025

Analytical Report: Mouse Cell Line Authentication

Received on: 07.04.2025
Start / End of Analysis: 07.04.2025 / 11.04.2025

By order of Nur Kocaturk, we were requested to perform a cell line authentication test for murine cell lines. The analysis was carried out with genomic DNA. The customer provided the sampling material. Following samples were examined:

Our sample number	Client sample name
25_ZE_000325	MEF_WT

Method:

DNA isolation was carried out from cell pellet (cell layer) separately from the samples.
Profiling of mouse cell line was done using highly-polymorphic short tandem repeat loci (STRs) published in Almeida et al. (2014) Mouse cell line authentication. Cytotechnology 66: 133-147. Fragment analysis was done on an ABI3130 (Life Technologies) and the resulting data were analysed with GeneMapper Software (Applied Biosystems).
Method details are given in: **SOP_APG_Zelllinienauthentizität_3.0**

All analyses have been carried out with greatest care and on the basis of state of the art scientific knowledge. The results refer solely to the analysed samples, as received. The duplication and publication also in parts requires a written authorization by this laboratory. Our General Terms and Conditions apply exclusively and are available under eurofinsgenomics.com

Results:

Marker / Sample	25_ZE_000325
**18-3**	15/15
**4-2**	19.3/20.3/21.3
**6-7**	14/15/16
**9-2**	19/19
**15-3**	22.3/22.3
**6-4**	18/18
**12-1**	17/17
**5-5**	17/18
**X-1**	27/27

Profile Comparison to References:

Not possible due to missing reference database for murine cell lines.

This report was created automatically and is therefore valid without a signature.

All analyses have been carried out with greatest care and on the basis of state of the art scientific knowledge. The results refer solely to the analysed samples, as received. The duplication and publication also in parts requires a written authorization by this laboratory. Our General Terms and Conditions apply exclusively and are available under eurofinsgenomics.com

Eurofins Genomics Europe Food/Environment/White Biotech Products & Services GmbH, Anzinger Str. 7 a, D-85560 Ebersberg

Dr. Nur Kocaturk
University of Dundee, School of Lifesciences
1 James Lindsay Place
DD1 5JJ Dundee
Great Britain

eurofins | Genomics

SOP_APG_Zelllinienauthentizität_A04_3

Person in charge: Dr. Torsten Brendel
Report date: 14.04.2025
Sample received on: 07.04.2025
Start / End of Analysis: 07.04.2025/14.04.2025

Analytical Report:
Cell Line Authentication Test
Order ID: 11109312168

Method:
DNA isolation was carried out from cell pellet (cell layer).
Genetic characteristics were determined by PCR-single-locus-technology.
16 independent PCR-systems D8S1179, D21S11, D7S820, CSF1PO, D3S1358, TH01, D13S317, D16S539, D2S1338, AMEL, D5S818, FGA, D19S433, vWA, TPOX and D18S51 were investigated.
(ASN-0002 core markers are colored grey, Thermo Fisher, AmpFlSTR® Identifiler® Plus PCR Amplification Kit)
In parallel, positive and negative controls were carried out yielding correct results.
Method details are given ir**SOP_APG_Zelllinienauthentizität_3.(**_15.04.2024

Result:

**Client Sample Name**	SHSY-5Y - CS
**Sample Code**	CL00022621
D8S1179	15,15
D21S11	31,31.2
D7S820	7,10
CSF1PO	11,11
D3S1358	15,16
TH01	7,10
D13S317	11,11
D16S539	8,13
D2S1338	17,19
D19S433	13,14
vWA	14,18
TPOX	8,11
D18S51	13,16
AMEL	X,X
D5S818	12,12
FGA	23.2,24
**Database Name**	SH-SY5Y

The table shows the result of the cell line analysis and the comparison with the online database of the DSMZ (http://www.dsmz.de/de/service/services-human-and-animal-cell) and the Cellosaurus database (https://web.expasy.org/cellosaurus). Please note that only the PCR-systems according to ANSI/ATCC standard ASN-0002 were aligned (D5S818, D13S317, D7S820, D16S539, VWA, TH01, TPOX, CSF1PO, AMEL - colored grey).

This report was created automatically and is therefore valid without a signature.

The laboratory is accredited acc. to **DIN EN ISO/IEC 17025:2018**. All analyses have been carried out with greatest care and on the basis of state of the art scientific knowledge. The results refer solely to the analysed samples, as received. The duplication and publication also in parts requires a written authorization by this laboratory. Our General Terms and Conditions apply exclusively and are available under eurofinsgenomics.com

Eurofins Genomics Europe Food/Environment/White Biotech Products & Services GmbH
Anzinger Str. 7a
85560 Ebersberg
Deutschland

Phone: +49 7531 816068
Email: support-eu@genomics.eurofinseu.com
www.eurofinsgenomics.com

Managing Director: Dr. Mashal Alawi
Register Court Munich HRB 207710
VAT ID: DE815473648

HypoVereinsbank
IBAN: DE23 2073 0017 7000 0006 50
SWIFT: HYVEDEMME17
