# Supplementary material for: Discovery of a CNS active GSK3 degrader using orthogonally reactive linker screening
Source: Nat Commun. 2025 Oct 6;16:8857. doi: 10.1038/s41467-025-63928-8 (PMC12501029; doi:10.1038/s41467-025-63928-8)
Supplement: Supplementary file 13 — Reporting Summary [file 41467_2025_63928_MOESM13_ESM.pdf]

nature portfolio

Corresponding author(s): *Double-anonymous peer review submissions: write DAPR and your manuscript number here instead of author names.*

Last updated by author(s): *YYYY-MM-DD*

# Reporting Summary

Nature Portfolio wishes to improve the reproducibility of the work that we publish. This form provides structure for consistency and transparency in reporting. For further information on Nature Portfolio policies, see our Editorial Policies and the Editorial Policy Checklist.

## Statistics

For all statistical analyses, confirm that the following items are present in the figure legend, table legend, main text, or Methods section.

| n/a | Confirmed | |
|---|---|---|
| ☐ | ☒ | The exact sample size (*n*) for each experimental group/condition, given as a discrete number and unit of measurement |
| ☐ | ☒ | A statement on whether measurements were taken from distinct samples or whether the same sample was measured repeatedly |
| ☐ | ☒ | The statistical test(s) used AND whether they are one- or two-sided<br>*Only common tests should be described solely by name; describe more complex techniques in the Methods section.* |
| ☒ | ☐ | A description of all covariates tested |
| ☐ | ☒ | A description of any assumptions or corrections, such as tests of normality and adjustment for multiple comparisons |
| ☐ | ☒ | A full description of the statistical parameters including central tendency (e.g. means) or other basic estimates (e.g. regression coefficient) AND variation (e.g. standard deviation) or associated estimates of uncertainty (e.g. confidence intervals) |
| ☐ | ☒ | For null hypothesis testing, the test statistic (e.g. *F*, *t*, *r*) with confidence intervals, effect sizes, degrees of freedom and *P* value noted<br>*Give P values as exact values whenever suitable.* |
| ☒ | ☐ | For Bayesian analysis, information on the choice of priors and Markov chain Monte Carlo settings |
| ☒ | ☐ | For hierarchical and complex designs, identification of the appropriate level for tests and full reporting of outcomes |
| ☒ | ☐ | Estimates of effect sizes (e.g. Cohen's *d*, Pearson's *r*), indicating how they were calculated |

*Our web collection on statistics for biologists contains articles on many of the points above.*

## Software and code

Policy information about availability of computer code

| | |
|---|---|
| Data collection | n/a |
| Data analysis | all software used for data analysis is described in methods.<br>Statistical analyses of assay data were performed using GraphPad Prism Software (v10.1.2).<br>FASTQ files further analysed on CRISPResso2 software.<br>For immunoblot analysis, images are captured by ChemiDoc MP Imaging system (Biorad) or LiCOR Odyssey and band intensities were quantified using Image Lab software (Bio-Rad, v6.1) or LiCOR imageStudio software (v95.2 or v6.0) respectively. Microsoft Excel and Graphpad Prism software (v10.1.2) used for further processing and statistical analysis.<br>Statistical analyses of proteomics data were performed with Perseus software (v1.6.15.0)<br>The proteomics raw data were searched using SEQUEST HT search engines with Proteome Discoverer 3.0 (Thermo Fisher Scientific). |

For manuscripts utilizing custom algorithms or software that are central to the research but not yet described in published literature, software must be made available to editors and reviewers. We strongly encourage code deposition in a community repository (e.g. GitHub). See the Nature Portfolio guidelines for submitting code & software for further information.

## Data

Policy information about availability of data

All manuscripts must include a data availability statement. This statement should provide the following information, where applicable:

- Accession codes, unique identifiers, or web links for publicly available datasets
- A description of any restrictions on data availability
- For clinical datasets or third party data, please ensure that the statement adheres to our policy

All data generated and analysed during this study are included in this published article and its supplementary information or are available from the corresponding authors upon reasonable request. A source data file is also provided. All proteomics data has been uploaded to the PRIDE repository with the following accession codes: PXD065662, PXD065692, PXD065722, PXD065750, PXD065740, PXD065724, PXD065731, PXD065760.

## Research involving human participants, their data, or biological material

Policy information about studies with human participants or human data. See also policy information about sex, gender (identity/presentation), and sexual orientation and race, ethnicity and racism.

| | |
|---|---|
| Reporting on sex and gender | *Use the terms sex (biological attribute) and gender (shaped by social and cultural circumstances) carefully in order to avoid confusing both terms. Indicate if findings apply to only one sex or gender; describe whether sex and gender were considered in study design; whether sex and/or gender was determined based on self-reporting or assigned and methods used. Provide in the source data disaggregated sex and gender data, where this information has been collected, and if consent has been obtained for sharing of individual-level data; provide overall numbers in this Reporting Summary. Please state if this information has not been collected. Report sex- and gender-based analyses where performed, justify reasons for lack of sex- and gender-based analysis.* |
| Reporting on race, ethnicity, or other socially relevant groupings | *Please specify the socially constructed or socially relevant categorization variable(s) used in your manuscript and explain why they were used. Please note that such variables should not be used as proxies for other socially constructed/relevant variables (for example, race or ethnicity should not be used as a proxy for socioeconomic status). Provide clear definitions of the relevant terms used, how they were provided (by the participants/respondents, the researchers, or third parties), and the method(s) used to classify people into the different categories (e.g. self-report, census or administrative data, social media data, etc.) Please provide details about how you controlled for confounding variables in your analyses.* |
| Population characteristics | *Describe the covariate-relevant population characteristics of the human research participants (e.g. age, genotypic information, past and current diagnosis and treatment categories). If you filled out the behavioural & social sciences study design questions and have nothing to add here, write "See above."* |
| Recruitment | *Describe how participants were recruited. Outline any potential self-selection bias or other biases that may be present and how these are likely to impact results.* |
| Ethics oversight | *Identify the organization(s) that approved the study protocol.* |

Note that full information on the approval of the study protocol must also be provided in the manuscript.

# Field-specific reporting

Please select the one below that is the best fit for your research. If you are not sure, read the appropriate sections before making your selection.

☒ Life sciences ☐ Behavioural & social sciences ☐ Ecological, evolutionary & environmental sciences

For a reference copy of the document with all sections, see nature.com/documents/nr-reporting-summary-flat.pdf

# Life sciences study design

All studies must disclose on these points even when the disclosure is negative.

| | |
|---|---|
| Sample size | Cellular screens were performed in biological duplicate within each of which there was a technical duplicate, sufficient for guiding decisions on hit identification. All subsequent cellular data was produced based on a minimum of 3 biological replicates. Pharmacokinetic (PK) studies typically use 3 animals for each dose route or dose level to be studied – this is because the use of only one or two animals carries a risk that variability between individual animals could result in differences in drug concentration which make interpretation of results difficult. Some studies included an additional brain penetration study group, also consisting of 3 animals, as such tissues could not be collected from the PK animals while retaining a full pharmacokinetic profile due to the terminal nature of the brain tissue collection. |
| Data exclusions | in cellular degradation experiments data points resulting in 'hooking' were removed prior to determination of kinetic and DC50 parameters, however data points are still included in figures. |
| Replication | all attempts at replication were successful. |

| | |
|---|---|
| Randomization | All animals are randomised on arrival. |
| Blinding | Animals are not blinded within each study. This is because pharmacokinetic studies consist of a very low number of animals, all of which receive the same test substance. Additionally, for practical reasons (for example dose administration and the first blood collections being only a few minutes apart), these procedures are typically performed by the same licensees, so blinding is not possible. As the results of the study are based on objective drug concentrations in the blood and brain, the effect of bias from the lack of blinding is limited. |

# Reporting for specific materials, systems and methods

We require information from authors about some types of materials, experimental systems and methods used in many studies. Here, indicate whether each material, system or method listed is relevant to your study. If you are not sure if a list item applies to your research, read the appropriate section before selecting a response.

## Materials & experimental systems

| n/a | Involved in the study |
|---|---|
| ☐ | ☒ Antibodies |
| ☐ | ☒ Eukaryotic cell lines |
| ☒ | ☐ Palaeontology and archaeology |
| ☐ | ☒ Animals and other organisms |
| ☒ | ☐ Clinical data |
| ☒ | ☐ Dual use research of concern |
| ☒ | ☐ Plants |

## Methods

| n/a | Involved in the study |
|---|---|
| ☒ | ☐ ChIP-seq |
| ☒ | ☐ Flow cytometry |
| ☒ | ☐ MRI-based neuroimaging |

## Antibodies

| | |
|---|---|
| Antibodies used | Anti-GSK3β (Abcam, ab93926, 1:1000 ), anti-GSK3α+ β (Abcam, ab185141, 1:1000), anti-GSK3α (Abcam, ab40870, 1:1000), anti-Cul2 (Abcam, ab166917, 1:1000), anti-UB (P4D1) (Enzo Life Sciences, BML-PW093001, 1:1000), anti-HIBIT (Promega, N7200 1:500), anti-c-Myc (Cell Signalling Technologies, 5605S, 1:1000), anti-GSK3 (Cell Signalling Technologies, 5676, lot 7, 1:1000), anti-β-actin (Sigma-Aldrich, A3853, 1:2000), anti-phospho (Thr 509/514) CRMP2 (MRC-PPU Reagents and Services, 1:1000), anti-β-catenin (Cell Signalling Technologies, 9562, 1:1000), anti-mouse IRDye 680RD (LiCOR, 926-68070, 1:5,000), anti-rabbit IRDye 800CW (LiCOR, 926-32211, 1:5,000), anti-mouse StarBright blue 520 (Bio-Rad, 12005866, Bio-Rad, 1:5,000), anti-Sheep IgG (H+L) Cross-Adsorbed Secondary Antibody, DyLight™ 800(Thermo Fisheer Scientific, SA5-10060, 1:5000), anti-rabbit (LiCOR, 926-32211, 1:10000), anti-mouse (Invitrogen, A21057, 1:10000), HRP-linked anti-sheep (Invitrogen, 31480, 1:5000), HRP-liked goat anti-rabbit (Invitrogen, 31460, 1:5000). For loading control, hFAB™ Rhodamine Anti-GAPDH Primary Antibody (Bio-Rad, 12004167, 1:10000), hFAB Rhodamine Anti-Actin Primary Antibody (Bio-Rad, 12004164, 1:10000) or Anti-actin (Monoclonal Antibody (BA3R), DyLight™, 1:10000) were used. In addition anti-phospho Ser9 GSK3β (CST, 5558T ,1:1000), anti-phospho Tyr216 GSK3β (Proteintech, 29125-1-AP ,1:1000), anti-CRBN (CST, were used. |
| Validation | Details of antibody validation by commercial vendors are provided at the links included here. Additional validation performed as part od this study, where carried out, are also specified. anti-GSK3β (Abcam, ab93926 ), anti-GSK3α+ β (Abcam, ab185141) and anti-GSK3α (Abcam, ab40870) were validated with siRNA experiments. anti-phospho Ser9 GSK3β (CST, 5558T , https://www.cellsignal.com/products/primary-antibodies/phospho-gsk-3b-ser9-d85e12-xp-rabbit-mab/5558), anti-phospho Tyr216 GSK3β (Proteintech, 29125-1-AP , https://www.ptglab.com/products/Phospho-GSK3B-Tyr216-Antibody-29125-1-AP.htm) and anti-phospho (Thr 509/514) CRMP2 (MRC-PPU Reagents and Services, https://mrcppureagents.dundee.ac.uk/reagents-view-antibodies/614300) also validated as part of this study with inhibitor controls (figure 4g). anti-HIBIT (Promega, N7200, https://www.promega.co.uk/products/protein-detection/protein-quantification/anti-hibit-antibody/?catNum=N7200#specifications) , also validated via western blotting by comparison with WT lysates in this study. Anti-β-catenin (Cell Signalling Technologies, 9562, https://www.cellsignal.com/products/primary-antibodies/b-catenin-antibody/9562). anti-Cul2 (Abcam, ab166917, https://www.abcam.com/en-us/products/primary-antibodies/cullin-2-cul-2-antibody-epr31042-ab166917) also validated by here by MLN4924 treatment in this study. Anti-UB (P4D1) (Enzo Life Sciences, BML-PW093001, https://www.enzo.com/product/ubiquitin-monoclonal-antibody-p4d1/) also validated by MG132 proteasomal inhibitor treatment in this study. anti-CRBN (CST 71810, https://www.cellsignal.com/products/primary-antibodies/crbn-d8h3s-rabbit-mab/71810) was also validated with KO cell lines in this study. anti-c-Myc (Cell Signalling Technologies, 5605S, https://www.cellsignal.com/products/primary-antibodies/c-myc-d84c12-rabbit-mab/5605). |

## Eukaryotic cell lines

Policy information about cell lines and Sex and Gender in Research

| | |
|---|---|
| Cell line source(s) | HEK293 was isolated from the kidney of a human embryo. HAP1 is a near-haploid human cell line derived from the KBM-7 chronic myelogenous leukemia (CML) cell line. KBM-7 cells were derived from a male patient but lack a Y chromosome, thus containing a single X chromosome when in the haploid state. MEF are mouse embryonic fibroblast cells. The SH-SY5Y cell line is a thrice cloned subline of the neuroblastoma cell line SK-N-SH, originating from a metastatic bone tumor from a 4-year-old female patient. |
| Authentication | All cell lines have been independently authenticated and STR data files are provided for all cell lines used. |
| Mycoplasma contamination | All cell lines were routinely tested for mycoplasma contamination. |

| | |
|---|---|
| Commonly misidentified lines (See ICLAC register) | *Name any commonly misidentified cell lines used in the study and provide a rationale for their use.* |

## Animals and other research organisms

Policy information about studies involving animals; ARRIVE guidelines recommended for reporting animal research, and Sex and Gender in Research

| | |
|---|---|
| Laboratory animals | Mice of the Balb/c strain (BALB/cAnNCrl) were obtained from Charles River Laboratories, UK. All animals were 7-9 weeks old at the start of the study. |
| Wild animals | This study did not involve wild animals. |
| Reporting on sex | Initial pharmacokinetic and brain penetration studies were performed on female animals only. These first-in-vivo studies use very low animal numbers, and so to investigate the effect on both sexes would increase variability and require an increase in total animal numbers. While sex has some potential to impact the degree of metabolism of a compound, it was determined unlikely to impact the eventual findings as to whether a compound is considered bioavailable and brain-penetrant. As such, during this early stage it was determined that the use of additional animals was not justified. Both male and female animals shall be used if further investigation is performed for these compounds. As all data has been generated in female mice, no sex- and gender-based analyses have been performed. We have specified in the abstract that in vivo data was obtained in female mice. |
| Field-collected samples | This study did not involve samples collected from the field |
| Ethics oversight | Overall ethical oversight was provided by the University of Dundee's local AWERB committee. Individual study plans were also inspected and approved by the Named Veterinary Surgeon prior to the start of work. |

Note that full information on the approval of the study protocol must also be provided in the manuscript.

## Plants

| | |
|---|---|
| Seed stocks | n/a |
| Novel plant genotypes | n/a |
| Authentication | n/a |
